# Electrochemical synthesis of biaryls by reductive extrusion from *N,N’*-diarylureas

**DOI:** 10.1038/s41467-023-40237-6

**Published:** 2023-07-28

**Authors:** Ellie Stammers, Chris D. Parsons, Jonathan Clayden, Alastair J. J. Lennox

**Affiliations:** 1 School of Chemistry, University of Bristol, Cantock’s Close, Bristol, BS8 1TS UK; 2 Early Chemical Development, Pharmaceutical Sciences, R&D, AstraZeneca, Macclesfield, SK10 2NA UK

**Keywords:** Synthetic chemistry methodology, Chemical synthesis

## Abstract

The synthesis of biaryl compounds by the transition-metal free coupling of arenes is an important contemporary challenge, aiming to avoid the toxicity and cost profiles associated with the metal catalysts commonly used in the synthesis of these pharmaceutically relevant motifs. In this paper, we describe an electrochemical approach to the synthesis of biaryls in which aniline derivatives are coupled through the formation and reduction of a temporary urea linkage. The conformational alignment of the arenes in the *N,N’*-diaryl urea intermediates promotes C-C bond formation following single-electron reduction. Our optimized conditions are suitable for the synthesis of a variety of biaryls, including sterically hindered examples carrying ortho-substituents, representing complementary reactivity to most metal catalysed methods.

## Abstract (web summary)

The synthesis of biaryls by transition metal-free coupling is an important challenge. Here, the authors describe an electrochemical approach to the synthesis of biaryls, in which aniline derivatives are coupled through the temporary formation and reduction of ureas.

## Introduction

The biaryl motif is a feature found in many compound classes, including natural products, pharmaceuticals, agrochemicals, organic electronic devices, and ligands for metals^1–3^. The limited conformational flexibility provided by the biaryl linkage makes it a ‘privileged’ moiety in bioactive molecules, prevalent in drugs across a range of therapeutic areas^4^.

**Fig. 1:**
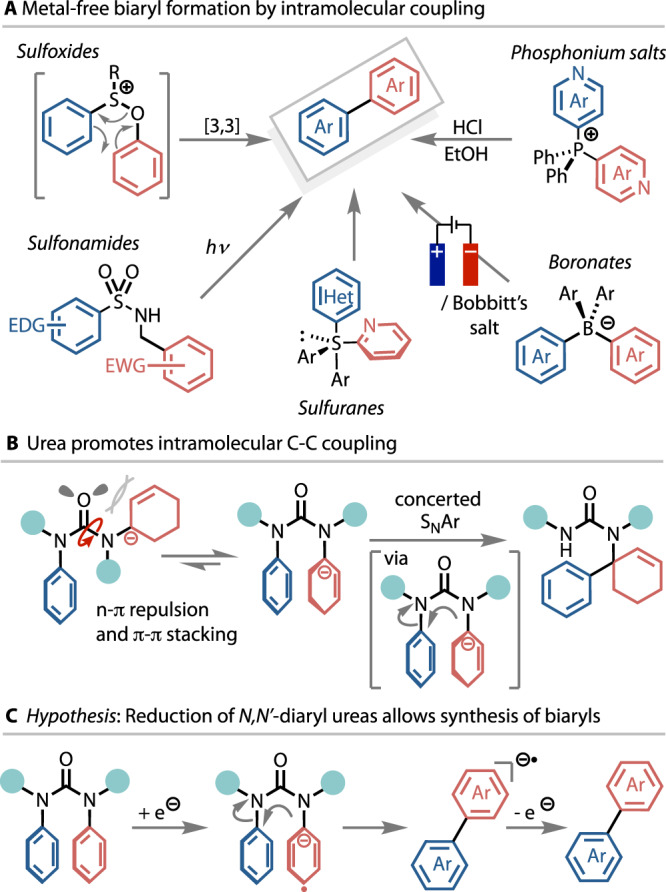
Previous intramolecular coupling strategies and project hypothesis. **A** Overview of transition metal-free biaryl coupling involving an intramolecular C–C bond-forming step. **B** Urea facilitates unconventional intramolecular C–C coupling from cis conformation. **C** Reductive extrusion of biaryls from reduced diaryl ureas.

Transition metal-catalysed cross-coupling methods provide the most common synthetic approaches to biaryls^5–7^, especially within medicinal chemistry^8^. These methods have the advantage of broad scope and versatility but set against this, the continuity of supply, the cost, and the toxicity of the transition metal catalysts have prompted significant exploration of alternative, metal-free, cross-coupling strategies^9^. Challenges associated with this class of reaction include regioselectivity^10^, functional group tolerance^11^, very specific coupling partners^12–18^, or a requirement for a high excess of coupling partner, often up to solvent level^19–25^. Many of these properties may be alleviated by an alternative intramolecular strategy in which both arenes are contained within the same species. Prominent examples of such precursors include phosphonium salts that allow the coupling of two heteroaromatic rings^26–30^, as well as sulfoxides^31,32^, sulfuranes^33–37^, sulphonamides^38–41^ and boron^42–46^ complexes (Fig. 1A).

Intramolecular reactivity is enhanced by structural features that favour reactive conformations, classically formulated as the Thorpe-Ingold effect. Thus, intramolecular nucleophilic aromatic substitution reactions of *N*-aryl ureas carrying a nucleophilic *N’*-substituent are accelerated by their conformational preference for the *N*-aryl ring to lie trans to the urea carbonyl group, and hence cis to the nucleophile (Fig. 1B). In this way, even electron-rich aniline derivatives exhibit electrophilic reactivity towards anionic nucleophiles, providing versatile methods for C(sp^3^)-arylation^47^.

Stevenson and co-workers reported in 2003 that reduction of *N*,*N*’-dimethyl-*N*,*N*’-diphenylurea using sodium in HMPA generates a biaryl radical anion, observable by EPR^48,49^. This spectroscopic observation suggested that reductive formation of a radical anion from an *N*-aryl urea could be used as a general trigger for Ar–Ar bond formation, with the return of aromaticity leading to C–N bond cleavage and extruding biaryl as an isolable product (Fig. 1C).

In this work, we show the development of a preparative method for the synthesis of biaryls by reductive cross-coupling based on this hypothesis. We show that the well-established conformational preference of *N*,*N*’-dialkyl-*N,N’*-diarylureas^50–54^ can be used to promote a transition-metal-free aryl-aryl cross-coupling reaction, which is complementary to existing biaryl forming strategies.

## Results and discussion

Lithium metal, in combination with di-*tert*-butylbiphenyl (DBB), has proved successful for the reduction of arenes under ammonia-free Birch conditions^55^ and is more practical than sodium in HMPA. Using this method for reduction of a model unsymmetrical urea substrate **1a**, variation of solvents, concentration, and reagent quantity ratios gave **2a** (entry 1), but in a yield of only 10%. Other substrates were surveyed, most of which gave similarly low yields, except for the electron-deficient *N,N’*-bis(4-cyanophenyl)urea **1b** (see SI).

Despite promising indications that reductive coupling was possible, the poor yield with LiDBB led us to explore alternatives. Among these, electrochemical reduction offers the advantage of tuneable redox potential and is inherently sustainable^56–59^, avoiding the use of stoichiometric metal. We thus turned to the electrochemical reduction of **2a**, optimising applied current, electrode material, solvent, and concentration. We noted an instant improvement with the cathodic reduction of the urea, giving a 51% yield of **2a**. After optimisation of other variables, including the addition of LiCl as an additive, an improved yield of 86% was achieved. The reaction outcome was particularly sensitive to the solvent, the amount of LiCl, and the electrode materials (Table 1)^60^. Sacrificial anodes such as aluminium worked well, but we chose to avoid the production of stoichiometric metal waste through the oxidation of bromide (present in the electrolyte as Bu_4_NBr) to tribromide. We propose that this method works particularly well because of the very slow migration of anionic tribromide product to the cathodic chamber and avoiding competitive reduction^61^.

**Table 1:** Reaction optimisation


	Reduction conditions^a^ {−/+}	LiCl eq.	1a/%^b^	2a/%^a^
1	Li, DBB	0	0	10
2	−6 mA {Gr/Gr}	0	27	51
3	−6 mA {Gr/Gr}	5	0	86
4	−6 mA {Gr/Gr}	10	0	77
5	−6 mA {Pt/Gr}	5	0	54
6	−6 mA {GC/Gr}	5	10	54
7	−6 mA {Gr/Pt}	5	0	79
8	−6 mA {Gr/CC}	5	13	68
9	−6 mA {Gr/Gr} in DMSO	5	0	67
10	−6 mA {Gr/Gr} in MeCN	5	18	44

^a^Electrochemical reactions conducted at constant current with {cathode/anode}. *Gr* graphite, *CC* carbon cloth.

^b^^1^H NMR yields.

**Fig. 2:**
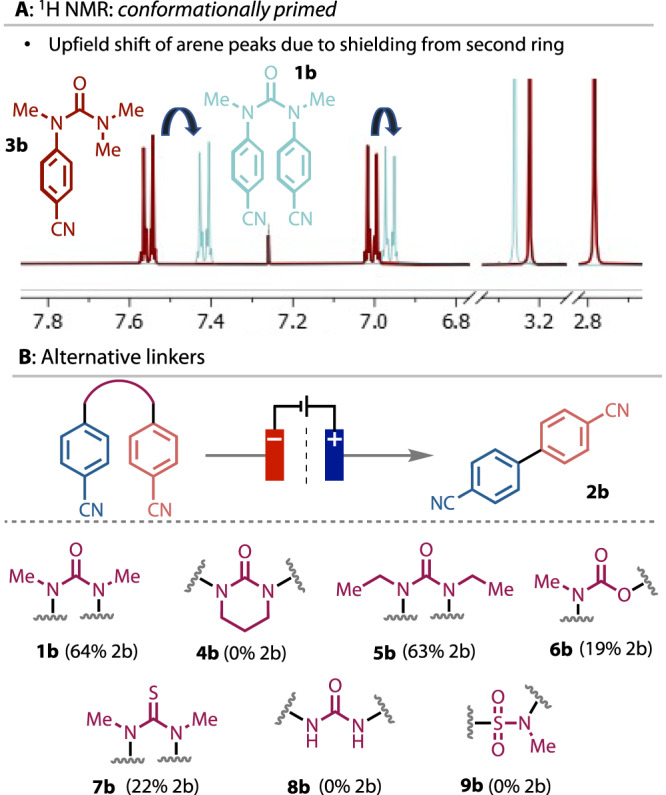
Conformation of diarylureas and variation of alternative linkers. **A** ^1^H NMR of **1b** and **3b**, which demonstrates the shielding effect of the second ring in **1b** due to the cis conformation. **B** A selection of alternative linkers that were tested in the biaryl coupling reaction.

The conformational preference imposed by the dimethylurea linker, which we propose brings the coupling partners into proximity, is evident in the ^1^H NMR spectra of **3b** and **1b**. An upfield shift of the aromatic ^1^H NMR signals on moving from monoaryl urea **3b** to diarylurea **1b** (Fig. 2A) suggests shielding of the proximal p-system. The role of this conformational preference on reactivity was tested by comparing the reduction of the dimethyl urea **1b** with the corresponding cyclic analogue **4b**, in which the aryl substituents necessarily point away from each other (Fig. 2B). None of the coupled **2b** was observed, consistent with our proposal. Additional evidence for an intramolecular, as opposed to intermolecular, coupling was provided by the lack of homo-coupled products from unsymmetrical ureas (see below) and by the absence of product when monoaryl urea **3b** was subjected to the reaction conditions. Molecules containing other linker units were also prepared and tested in order to gauge their reactivity relative to dimethylurea. Ethyl substitution (**5b**) was tolerated, but any further changes were detrimental to the reaction outcome. The carbamate (**6b**) and thiourea (**7b**) gave low yields, and the unmethylated urea (**8b**) and sulphonamide (**9b**) afforded no product. With substrates other than ureas, significant cleavage of the linker was observed under the reaction conditions (see SI for more details).

**Fig. 3:**
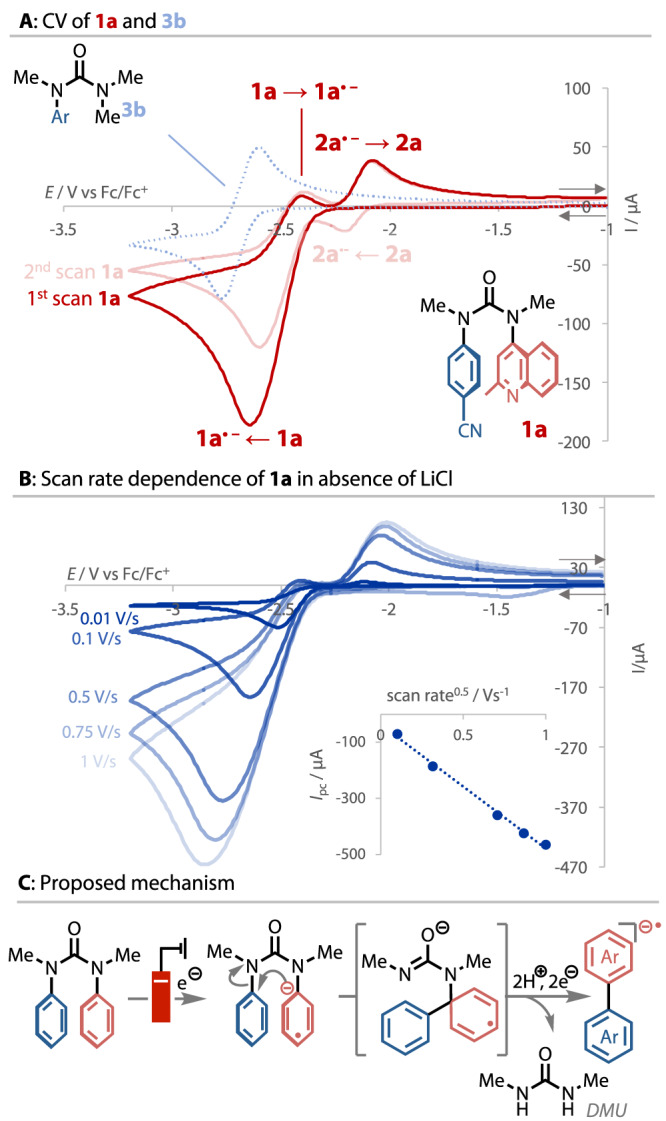
Mechanistic studies. **A** CV of **1a** with 1 repeat scan, co-plotted with a CV of **3b**. Glassy carbon (−)|Pt wire (+), Ag/AgNO_3_ (ref), 0.1 V s^−1^, 0.025 mmol substrate, 0.1 M TBAPF_6_, 0.05 M DMF; **B** scan rate dependence of **1a**, inset: cathodic peak current vs scan rate^0.5^; **C** proposed overall mechanism, DMU dimethylurea.

Rapid biaryl formation on the cyclic voltammetry (CV) timescale was evident from CV analysis of **1a**, Fig. 3A. The CV trace of **1a** was irreversible, indicating that the radical anion formed on the electrode surface must react to give further products. Indeed, the reverse sweep shows an oxidation peak for product **2a**, and the second scan shows a new reduction peak for product **2a** formed in the first scan. In contrast, the monoaryl urea **3b** gave a reversible CV trace, indicating that, without a second arene coupling partner, no onward reaction takes place from the reduced species. Product **2a** was formed on the CV timescale at scan rates up to 1 V/s, the fastest tested (Fig. 1B). The observed linear relationship between the *I*_pc_ and the square root of the scan rate reveals, according to the Randles-Sevcik equation, freely diffusing redox species. However, the reduction peak shape changed when LiCl was added. When LiCl was titrated, the peak adopted a progressively more symmetrical shape, with no evidence for the formation of **2a**. A loss of current was observed in subsequent scans when the electrode was not polished. Although this CV analysis could not fully reveal the role of LiCl, an electron-transfer process of a surface-bound LiCl–urea adduct could account for this evidence (see SI for details)^62,63^.

In light of these results, we propose the reaction mechanism illustrated in Fig. 3C, in which the key step is the intramolecular C–C coupling of the conformationally preorganised reduced urea. Considering the electronic promiscuity of the reaction (see below), it is likely that a concerted S_N_Ar reaction occurs, as proposed in related reactions of *N*-aryl ureas^64–66^. A time-course experiment showed that dimethyl urea (DMU) is the sole by-product of the reaction and is formed in direct parity with product **2b** (see SI). Several possibilities exist for the liberation of the biaryl from the rearranged intermediate, including C-N bond homolysis, a second reduction, or the intermediate formation of a diaziridinone^48,67^. The biaryl appears to persist in a reduced state (a radical anion or dianion) until work-up, at which point it undergoes aerobic oxidation. Indeed, we noted the decomposition of these reduced species if the reaction mixture was left for prolonged periods without workup.

**Fig. 4:**
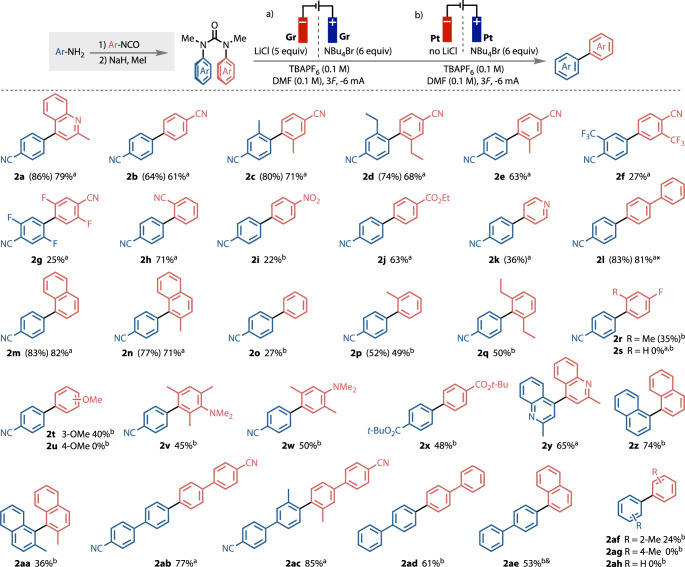
Substrate scope. Substrate scope, Yields = isolated yields (NMR yields); Gr = graphite rod. *Gr (−) flaked during reaction, ^&^ 2*F* applied.

Figure 4 details the range of biaryl products that have been formed by electrochemical reduction of *N,N*’-diarylureas. Using the same conditions (conditions a) as model substrates **2a** and **2b,** *ortho* alkyl substitution was well tolerated in reactions giving biaryls **2c–e**, suggesting that steric hindrance was not detrimental to the coupling reaction.

Trifluoromethyl and fluoro substituents were also tolerated (**2f,** **2g**), though these rings coupled in lower yield. Unsymmetrical biaryls were similarly formed when a *p*-cyanophenyl ring was paired with other electron-deficient rings, yielding products **2h**–**2k**, and the coupling of nitrile or ester-substituted rings (**2h,** **2j**) were the highest yielding, with nitro substituted **2i** the lowest yielding presumably due to enhanced stability of the reduced form. Coupling *p*-cyanophenyl with biphenyl (**2l**) and naphthalene (**2m, 2n**) partners having more extended, and therefore reducible and electrophilic, π-systems was also especially effective.

Coupling with more electron-rich partners required a change of reaction conditions (conditions b). Platinum electrodes were used, as the graphite electrodes were unstable at very deep electrode potentials, and LiCl was removed to avoid competitive reduction of lithium cations. These modifications enabled coupling of the *p*-cyanophenyl ring to phenyl (**2o**) and ortho-substituted phenyl (**2p,** **2q**) and fluorophenyl (**2r**) rings, as well as with methoxy and amino-substituted partners (**2t,** **2v** and **2w**). Limitations to the tolerance of less electron-withdrawing and halogen substituents were indicated by the failure of the 4-methoxyphenyl coupling (**2u,** **2s**), in contrast to the successful 3-methoxyphenyl coupling **2t**. Such substituents are nonetheless tolerated in the 4-position (**2r,** **2w**) provided they are accompanied by a 2-alkyl substituent, an observation that highlights a beneficial effect from 2-alkyl substituents that is also apparent when comparing the yields of **2o** with **2p** and **2q**, and in the formation of **2af**. *Ortho* substituents may help to increase the population of the reactive conformer by favouring face-to-face rather than edge-to-face p-p interactions. This effect contrasts with established cross-coupling methods, in which more hindered partners require more reactive and specialised catalyst systems^68–71^.

Successful coupling with more electron-deficient and conjugated p-systems was extended further to ureas in which neither ring carried a *p*-cyano group. The biphenyl-4,4’-diester **2x**, biquinolyl **2y**, binaphthyl **2z** and even the hindered atropisomeric tetra-ortho*-*substituted 2,2’binaphthyl **2aa** all formed successfully. Biphenyl substrates coupled exceptionally well, giving tetraphenyls **2ab,** **2ac** and **2ad**, and naphthylbiphenyl **2ae**. Biphenyl itself could not be formed from *N,N’*-diphenylurea, but 2,2’-dimethylbiphenyl **2af** was formed in low yield.

In summary, we report a new electrochemical reductive method for the formation of biaryls. This metal-free approach uses readily available anilines as coupling partners, tethering them through a urea linkage whose conformational preference enforces proximity between the arene rings. Electrochemical reduction forms the biaryl product, extruding dimethylurea as a by-product. The reaction scope is complementary to more established transition-metal catalysed coupling methods, being especially amenable to electron-deficient and sterically hindered ortho-substituted biaryl products.

## Methods

The Supplementary Information provides full details of methods for the synthesis of all urea starting materials, their spectroscopic characterisation and their reduction to biaryls. For the electrochemical reduction of urea to a biaryl: a solution of the urea (1 eq), LiCl (5 eq), TBAPF_6_ (0.1 M) in DMF on the cathodic side of a divided cell and TBAPF_6_ (0.1 M) and TBAB (6 eq) in DMF on the anodic side were electrolysed (3*F*, −6 mA) with graphite electrodes under an atmosphere of N_2_.

## Supplementary information

Supplementary information

Peer Review File

## Data Availability

The data that support the findings of this study are available in the supplementary material of this article.

## References

[1] Simonetti M, Cannas DM, Larrosa I (2017). Biaryl Synthesis via C–H Bond Activation: Strategies and Methods. Adv. Organomet. Chem..

[2] Triggvi J (2010). Top 200 Pharmaceuticals. J. Chem. Ed..

[3] Xin H (2009). Efficient Solar Cells Based on a New Phthalimide-Based Donor-Acceptor Copolymer Semiconductor: Morphology, Charge-Transport, and Photovoltaic Properties. J. Mater. Chem..

[4] Horton DA, Bourne GT, Smythe ML (2003). The Combinatorial Synthesis of Bicyclic Privileged Structures or Privileged Substructures. Chem. Rev..

[5] Oh-e T, Miyaura N, Suzuki A (1993). Palladium-Catalysed Cross-Coupling Reaction of Organoboron Compounds with Organic Triflates. Angew. Chem. Int. Ed..

[6] Kotha S, Lahiri K, Kashinath D (2002). Recent Applications of the Suzuki-Miyaura Cross-Coupling Reaction in Organic Synthesis. Tetrahedron.

[7] Lennox AJJ, Lloyd-Jones GC (2014). Selection of Boron Reagents for Suzuki-Miyaura Coupling. Chem. Soc. Rev..

[8] Brown DG, Boström J (2016). Analysis of Past and Present Synthetic Methodologies on Medicinal Chemistry: Where Have All the New Reactions Gone?. J. Med. Chem..

[9] Sun CL, Shi ZJ (2014). Transition-Metal-Free Coupling Reactions. Chem. Rev..

[10] Castro S, Fernández JJ, Vicente R, Fañanás FJ, Rodríguez F (2012). Base- and Metal-Free C–H Direct Arylations of Naphthalene and Other Unbiased Arenes with Diaryliodonium Salts. Chem. Commun..

[11] Shirakawa E (2012). Cross-Coupling of Aryl Grignard Reagents with Aryl Iodides and Bromides through S_RN_1 Pathway. Angew. Chem. Int. Ed..

[12] Ackermann L, Dellacqua M, Fenner S, Vicente R, Sandmann R (2011). Metal-Free Direct Arylations of Indoles and Pyrroles with Diaryliodonium Salts. Org. Lett..

[13] Kita Y (2009). Metal-Free Oxidative Cross-Coupling of Unfunctionalized Aromatic Compounds. J. Am. Chem. Soc..

[14] Becht JM, Ngouela S, Wagner A, Mioskowski C (2004). Straightforward Anionic Coupling for the Synthesis of Ortho-Bromobiaryls. Tetrahedron.

[15] Becht JM, Gissot A, Wagner A, Mioskowski C (2004). An Efficient Synthesis of Biaryls via Noncatalysed Anionic Coupling of an Arylsodium with Haloarenes. Tetrahedron Lett..

[16] Elsler B (2015). Source of Selectivity in Oxidative Cross-Coupling of Aryls by Solvent Effect of 1,1,1,3,3,3-Hexafluoropropan-2-ol. Eur. J. Chem..

[17] Kirste A, Schnakenburg G, Stecker F, Fischer A, Waldvogel SR (2010). Anodic Phenol-Arene Cross-Coupling Reaction on Boron-Doped Diamond Electrodes. Angew. Chem. Int. Ed..

[18] Jurrat M, Maggi L, Lewis W, Ball LT (2020). Modular Bismacycles for the Selective C–H Arylation of Phenols and Naphthols. Nat. Chem..

[19] Liu W, Tian F, Wang X, Yu H, Bi Y (2013). Simple Alcohols Promoted Direct C-H Arylation of Unactivated Arenes with Aryl Halides. Chem. Commun..

[20] Tanimoro K, Ueno M, Takeda K, Kirihata M, Tanimori S (2012). Proline Catalyzes Direct C-H Arylations of Unactivated Arenes. J. Org. Chem..

[21] Qiu Y (2011). New Ligands That Promote Cross-Coupling Reactions between Aryl Halides and Unactivated Arenes. Org. Lett..

[22] Yanagisawa S, Ueda K, Taniguchi T, Itami K (2008). Potassium T-Butoxide Alone Can Promote the Biaryl Coupling of Electron-Deficient Nitrogen Heterocycles and Haloarenes. Org. Lett..

[23] Lee J, Hong B, Lee A (2019). Visible-Light-Promoted, Catalyst-Free Gomberg-Bachmann Reaction: Synthesis of Biaryls. J. Org. Chem..

[24] Crisóstomo FP, Martín T, Carrillo R (2014). Ascorbic Acid as an Initiator for the Direct C-H Arylation of (Hetero)Arenes with Anilines Nitrosated in Situ. Angew. Chem. Int. Ed..

[25] Hari DP, Schroll P, König B (2012). Visible-Light-Mediated Direct C-H Arylation of Heteroarenes with Aryl Diazonium Salts. J. Am. Chem. Soc..

[26] Mann FG, Watson J (1948). Conditions of Salt Formation in Polyamines and Kindred Compounds. Salt Formation in the Tertiary 2-Pyridylamines, Phosphines and Arsines. J. Org. Chem..

[27] Newkome G, Hager DC (1978). A New Contractive Coupling Procedure. Convenient Phosphorous Expulsion Reaction. J. Am. Chem. Soc..

[28] Uchida Y, Kozawa H (1989). Formation of 2,2’-Bipyridyl by Ligand Coupling on the Phosphorus Atom. Tetrahedron Lett..

[29] Hilton MC (2018). Heterobiaryl Synthesis by Contractive C-C Coupling via P(V) Intermediates. Science.

[30] Boyle BT, Hilton MC, McNally A (2019). Nonsymmetrical Bis-Azine Biaryls from Chloroazines: A Strategy Using Phosphorus Ligand-Coupling. J. Am. Chem. Soc..

[31] Yanagi T (2016). Metal-Free Approach to Biaryls from Phenols and Aryl Sulfoxides by Temporarily Sulfur-Tethered Regioselective C-H/C-H Coupling. Am. Chem. Soc..

[32] Yanagi T (2020). Construction of Biaryls from Aryl Sulfoxides and Anilines by Means of a Sigmatropic Rearrangement. Eur. J. Chem..

[33] Franzen V, Joschek HI, Mertz C (1962). Reaction of Benzene with Thioethers. Justus Liebigs Ann. Chem..

[34] Larochelle R, Trost BM (1971). Reactions of Organolithiums with Arylsulfonium Salts. J. Am. Chem. Soc..

[35] Oae S, Ishihara H, Yoshihara M (1995). Reaction of Triphenylsulfonium Salt with Organolithium Reagents. Chem. Heterocycl. Compd..

[36] Duong VK, Horan AM, McGarrigle EM (2020). Synthesis of Pyridylsulfonium Salts and Their Application in the Formation of Functionalized Bipyridines. Org. Lett..

[37] Horan AM, Duong VK, McGarrigle EM (2021). Synthesis of Bis-Heteroaryls Using Grignard Reagents and Pyridylsulfonium Salts. Org. Lett..

[38] Motherwell WB, Pennell AMK (1991). A Novel Route to Biaryls via Intramolecular Free Radical Ipso Substitution Reactions. J. Chem. Soc. Chem. Commun..

[39] da Mata ML, Motherwell WB, Ujjainwalla F (1997). Steric and Electronic Effects in the Synthesis of Biaryls and Their Heterocyclic Congeners Using Intramolecular Free Radical [1,5] Ipso Substitution Reactions. Tetrahedron Lett..

[40] Holden CM, Sohel SMA, Greaney MF (2016). Metal Free Bi(hetero)aryl Synthesis: A Benzyne Truce–Smiles Rearrangement. Angew. Chem. Int. Ed..

[41] Kloss F, Neuwirth T, Haensch VG, Hertweck C (2018). Metal-Free Synthesis of Pharmaceutically Important Biaryls by Photosplicing. Angew. Chem. Int. Ed..

[42] Geske DH (1959). The Electrooxidation of the Tetraphenylboarte Ion; An Example of a Secondary Chemical Reaction Following the Primary Electrode Process. J. Phys. Chem.

[43] Music A (2020). Electrochemical Synthesis of Biaryls via Oxidative Intramolecular Coupling of Tetra(Hetero)Arylborates. J. Am. Chem. Soc..

[44] Music A (2021). Photocatalyzed Transition-Metal-Free Oxidative Cross-Coupling Reactions of Tetraorganoborates. Eur. J. Chem..

[45] Paul S (2020). Transition-Metal-Free Synthesis of Heterobiaryls through 1,2-Migration of Boronate Complex. Eur. J. Chem..

[46] Gerleve C, Studer A (2020). Transition-Metal-Free Oxidative Cross-Coupling of Tetraarylborates to Biaryls Using Organic Oxidants. Angew. Chem. Int. Ed..

[47] Tait MB, Ottersbach PA, Tetlow DJ, Clayden J (2014). Synthesis of 1-Arylcycloalkenamines by Intramolecular Arylation of Lithiated Ureas. Org. Process Res. Dev..

[48] Kurth TL, Lewis FD, Hattan CM, Reiter RC, Stevenson CD (2003). N,N′-Dimethyl-N,N′-Diarylurea Anion Radicals: An Intramolecular Reductive Elimination. J. Am. Chem. Soc..

[49] Lewis FD, Kurth TL, Hattan CM, Reiter RC, Stevenson CD (2004). Polyaryl Anion Radicals via Alkali Metal Reduction of Arylurea Oligomers. Org. Lett..

[50] Lepore G, Migdal S, Blagdon D, Goodman M (1973). Conformations of Substituted Aryl Ureas in Solution. J. Org. Chem..

[51] Lepore U, Lepore G, Ganis P (1976). Conformation of Substituted Aryl Ureas. J. Org. Chem..

[52] Clayden J (2008). Conformation and Stereodynamics of 2,2′-Disubstituted N,N′-Diaryl Ureas. Org. Biomol. Chem..

[53] Clayden J, Hennecke U, Vincent MA, Hillier IH, Helliwell M (2010). The Origin of the Conformational Preference of N,N′-Diaryl-N,N′-Dimethyl Ureas. Phys. Chem. Chem. Phys..

[54] Tanatani A (1997). Aromatic Urea and Guanidine. Tetrahedron Lett..

[55] Donohoe TJ, House D (2002). Ammonia Free Partial Reduction of Aromatic Compounds Using Lithium Di-Tert-Butylbiphenyl (LiDBB). J. Org. Chem..

[56] Yan M, Kawamata Y, Baran PS (2017). Synthetic Organic Electrochemical Methods since 2000: On the Verge of a Renaissance. Chem. Rev..

[57] Wiebe A (2018). Electrifying Organic Synthesis. Angew. Chem. Int. Ed..

[58] Möhle S (2018). Modern Electrochemical Aspects for the Synthesis of Value-Added Products. Angew. Chem. Int. Ed..

[59] Pollok D, Waldvogel SR (2020). Electro-Organic Synthesis-a 21^st^ century Technique. Chem. Sci..

[60] Heard DM, Lennox AJJ (2020). Electrode Materials in Modern Organic Electrochemistry. Angew. Chem. Int. Ed..

[61] Box JR, Atkins AP, Lennox AJ (2021). Direct Electrochemical Hydrodefluorination of Trifluoromethylketones Enabled by Non-Protic Conditions. J. Chem. Sci..

[62] Grujicic D, Pesic B (2002). Electrodeposition of Copper: The Nucleation Mechanisms. Electrochim. Acta.

[63] Juma JA (2021). The Effect of Organic Additives in Electrodeposition of Co from Deep Eutectic Solvents. Arab. J. Chem..

[64] Kwan EE, Zeng Y, Besser HA, Jacobsen EN (2018). Concerted Nucleophilic Aromatic Substitutions. Nat. Chem..

[65] Meisenheimer Complexes in SNAr Reactions: Intermediates or Transition States? *Int. Ed*. **130**, 14898–14900 (2018). 10.1002/anie.201809606 30320484

[66] Leonard DJ, Ward JW, Clayden J (2018). Asymmetric α-Arylation of Amino Acids. Nature.

[67] To gauge some insight into this, we synthesized the *t*-Bu substituted diaziridinone, which is more easily prepared than the dimethyl diaziridinone. Subjecting it to the reaction conditions did indeed lead to the corresponding di-*tert*-butylurea, suggesting the diaziridinone can be readily reduced electrochemically.

[68] Schaarschmidt D, Grumbt M, Hildebrandt A, Lang HA (2014). A Planar-Chiral Phosphino(Alkenyl)Ferrocene for Suzuki-Miyaura C-C Coupling Reactions. Eur. J. Chem..

[69] Raders SM (2013). Trineopentylphosphine: A Conformationally Flexible Ligand for the Coupling of Sterically Demanding Substrates in the Buchwald-Hartwig Amination and Suzuki-Miyaura Reaction. J. Org. Chem..

[70] To SC, Kwong FY (2011). Highly Efficient Carbazolyl-Derived Phosphine Ligands: Application to Sterically Hindered Biaryl Couplings. Chem. Commun..

[71] Zhao Q, Li C, Senanayake CH, Tang W (2013). An Efficient Method for Sterically Demanding Suzuki-Miyaura Coupling Reactions. Eur. J. Chem..

