# Supplementary material for: Electrochemical synthesis of biaryls by reductive extrusion from N,N’-diarylureas
Source: Nat Commun. 2023 Jul 28;14:4561. doi: 10.1038/s41467-023-40237-6 (PMC10382484; doi:10.1038/s41467-023-40237-6)
Supplement: Supplementary file 1 — Supplementary information [file 41467_2023_40237_MOESM1_ESM.pdf]

Supplementary Information

Electrochemical Synthesis of Biaryls by Reductive Extrusion from *N*,*N*'-diarylureas

Ellie Stammers,[a] Chris D. Parsons,[b] Jonathan Clayden*[a] and Alastair J. J. Lennox,*[a]

[a] School of Chemistry, University of Bristol, Cantock's Close, Bristol BS8 1TS, UK; [b] Early Chemical Development, Pharmaceutical Sciences, R&D, AstraZeneca, Macclesfield, SK10 2NA, UK.
*a.lennox@bristol.ac.uk and j.clayden@bristol.ac.uk

Table of Contents

1. Supplementary methods 3

1.1 General experimental 3

1.2 General Procedures 3

GP1a – Symmetrical urea synthesis from *N*-methylated substituted anilines 3

GP1b – Symmetrical urea synthesis from substituted anilines 3

GP2a – Unsymmetrical urea synthesis from isocyanate and aniline 4

GP2b – Unsymmetrical urea synthesis from *N*-methylated substituted aniline and carbamoyl chloride 4

GP2c – Unsymmetrical ureas synthesis from reaction of substituted anilines with CDI 4

GP3 – *N*-methylation of *N,N'*-diaryl ureas 4

GP4a – LiDBB reduction of ureas 4

GP4b – LiDBB reduction of ureas in the presence of LiCl 4

GP5a – Electrochemical reduction of ureas, Gr(+) Gr(-), 5 eq LiCl 5

GP5b – Electrochemical reduction of ureas, Pt(+) Pt(-) 5

1.3 Synthesis of Ureas 5

1.4 Biaryl Products 26

1.4 Other linkers 38

1.5 Synthetic intermediates 41

1.6 Compounds synthesised for mechanistic investigation 46

2. LiDBB conditions 48

2.1 Optimisation of LiDBB conditions 48

2.2 Scope of biaryl formation using LiDBB as reductant 49

2.3. Unsuccessful substrates using LiDBB conditions 49

3. Investigation of different linkers 50

4. Electrochemical optimisation 50

5. Unsuccessful substrates in electrochemical reduction 51

6. Electrochemical set up 51
7. Diaziridinone CV and electrochemical reaction 52
8. Comparison of reduction potentials to reaction conditions 53
9. CV of urea 1a (3 scans) overlaid with CV of biaryl product 2a 54
10. Effect of LiCl on CV of urea 1a 55
11. CVs of ureas 56
12. NMR spectra 60
  12.1 NMR spectra of ureas 60
  12.1 NMR spectra of biaryl products 99
  12.3 NMR spectra of other linkers 129
  12.4 NMR spectra of synthetic intermediates 135
  12.5 NMR spectra of compounds synthesised for mechanistic investigation 143
13. Supplementary References 146

1. Supplementary methods

1.1 General experimental

Syntheses requiring inert conditions were carried out under a nitrogen atmosphere and glassware was flame-dried prior to use. All commercial reagents and non-anhydrous solvents were purchased at the highest quality and used as supplied. Reaction mixtures were stirred magnetically. Air- and moisture-sensitive liquids and solutions were transferred via syringe into the reaction vessels through rubber septa. Anhydrous solvents were obtained using the Anhydrous Engineering Ltd. double alumina and alumina-copper catalysed drying columns and stored in a Young's flask.

Flash column chromatography was performed on an automated Biotage IsoleraTM Spektra Four using gradient elution on pre-packed silica gel Biotage® ZIP columns, or manually using silica gel (Aldrich 40-63 µm, 230-400 mesh). TLC was performed on aluminium backed silica plates (0.2 mm, 60 F254) which were visualised with UV fluorescence (254 & 366 nm).

All NMR spectra were recorded at 25 °C on a Bruker 400, Varian 400, Jeol ECZ400 or Jeol ECS400 spectrometer and processed using MestReNova 14.0.1. Chemical shifts (δ) are quoted in parts per million (ppm) and referenced to the appropriate NMR solvent peak ($CDCl_3$ = 7.26 (^{1}H), 77.16 (^{13}C), d_6-DMSO = 2.50 (^{1}H), 39.52 (^{13}C)). ^{1}H NMR coupling constants are reported in Hz and refer to apparent multiplicities. ^{1}H coupling constants are reported to the nearest 0.1 Hz and where coupling did not match as a result of digitisation are reported as rounded. NMR data is reported in the following format, chemical shift (integration, multiplicity (s = singlet, d = doublet, t = triplet, q = quartet, m = multiplet), *J* coupling constant, assignment). COSY, HSQC, and HMBC techniques were routinely utilised to definitively assign the signals of ^{1}H and ^{13}C NMR spectra. Mass spectrometry (ESI and APCI) was carried out by the Bristol Mass Spectrometry Service.

All IR spectra were recorded on a Perkin Elmer FTIR spectrometer and are quoted as ν in cm^{-1}, and assigned strong (s), medium (m), weak (w) or broad (br). Melting points were measured on a Kofler hotstage melting point apparatus.

All chronopotentiometric measurements or electrochemical reactions were performed at room temperature using a PalmSens4 or TENMA PLH250 power pack

1.2 General Procedures

GP1a – Symmetrical urea synthesis from *N*-methylated substituted anilines

By the method of Clayden[1] the *N*-methyl aniline (1 eq) with Et_3N (2.2 eq) was dissolved in toluene (0.1 M) and cooled to 0 °C. Triphosgene (0.16 eq) was added portionwise and the reaction refluxed overnight. Upon cooling the reaction mixture was diluted with water and extracted with 3 x EtOAc. The organic layers were dried with anhydrous $MgSO_4$, filtered and concentrated under reduced pressure to afford the crude product. Purification by silica column chromatography afforded the desired product.

GP1b – Symmetrical urea synthesis from substituted anilines

By the method of Clayden[1] the substituted aniline (1 eq) with Et_3N (2.2 eq) was dissolved in CH_2Cl_2 (0.2 M) and cooled to 0 °C. Triphosgene (0.16 eq) was added portion wise and the reaction mixture stirred at rt overnight. Water was added to the reaction mixture and the precipitate filtered out, and further washed with water. Where no significant precipitate formed, product extracted from the aqueous layer with CH_2Cl_2, dried with anhydrous $MgSO_4$ and concentrated under reduced pressure to give the desired urea in good purity.

GP2a – Unsymmetrical urea synthesis from isocyanate and aniline

By the method of Clayden[1] the substituted aniline (1 eq) was dissolved in CH_2Cl_2 (0.2 M). The isocyanate (1 eq) was added portion wise and the reaction mixture stirred overnight at rt. The resulting precipitate was filtered out to give the desired urea in good purity.

GP2b – Unsymmetrical urea synthesis from *N*-methylated substituted aniline and carbamoyl chloride

By the method of Clayden[2] the *N*-methyl aniline (1 eq) was dissolved in THF (1 M) and cooled to -78 °C. KHMDS (1M in THF, 1.15 eq) was added dropwise and the reaction mixture stirred at -78 °C for 1 hr. A solution of the desired carbamoyl chloride (1 eq) in THF (1 M) was added and the reaction mixture warmed to room temperature overnight. The reaction mixture was quenched with MeOH, diluted with EtOAc and washed with water and brine. The organic layer was dried with anhydrous $MgSO_4$, filtered and concentrated under reduced pressure to afford the crude product. Purification by silica column chromatography afforded the desired product.

GP2c – Unsymmetrical ureas synthesis from reaction of substituted anilines with CDI

By the method of DeForest[3] the first aniline (1 eq) was added portionwise to a solution of CDI (1 eq) in MeCN (0.3 M) over 15 mins. After stirring overnight MsOH (2 eq) was added dropwise and the reaction mixture left to stirred for 2-3 hours. The second aniline (1 eq) was then added and the reaction mixture stirred overnight. Water was added and the resulting precipitate filtered out affording the desired product.

GP3 – *N*-methylation of *N,N'*-diaryl ureas

By the method of Clayden[1] the *N,N'*-diaryl urea (1 eq) was dissolved in THF (0.05 M) and cooled to 0 °C. NaH (60% dispersion in mineral oil, 3 eq) was added portion wise and the reaction mixture warmed to rt over 1 hr. MeI (4 eq) was added and the reaction stirred overnight at rt. The solvent was removed under reduced pressure and the resulting solid diluted with water and extracted with EtOAc. The organic layers were dried with anhydrous $MgSO_4$, filtered and concentrated under reduced pressure to afford the crude product. Purification by silica column chromatography afforded the desired product.

GP4a – LiDBB reduction of ureas

Small pieces of Li wire (10 eq) were added to a solution of DBB (1 eq) in THF (0.04 M) under an atmosphere of N_2 (or Ar). Using a glass rod, inserted through a rubber septum, the pieces of Li wire were pressed again the side of the Schlenk tube until a colour change to a dark blue solution was observed (~10 mins). The solution was stirred for an additional 10 minutes and a solution of urea (1 eq) in THF (0.1 M) was added. Upon completion of the reaction the reaction mixture was cooled to 0 °C and quenched with water. The organic layer was extracted with 3 x Et_2O or CH_2Cl_2, dried with anhydrous $MgSO_4$, filtered and concentrated under reduced pressure to afford the crude product. Purification by silica column chromatography afforded the desired product.

GP4b – LiDBB reduction of ureas in the presence of LiCl

Small pieces of Li wire (10 eq) were added to a solution of DBB (1 eq) and LiCl (5 eq) in THF (0.04 M) under an atmosphere of N_2 (or Ar). Using a glass rod, inserted through a rubber septum, the pieces of Li wire were pressed again the side of the Schlenk tube until a colour change to a dark blue solution was observed (~10 mins). The solution was stirred for an additional 10 minutes and a solution of urea (1 eq) in THF (0.1 M) was added. Upon completion of the reaction the reaction mixture was cooled to 0 °C and quenched with water. The organic layer was extracted with 3 x Et_2O or CH_2Cl_2, dried with anhydrous $MgSO_4$, filtered and concentrated under reduced

pressure to afford the crude product. Purification by silica column chromatography afforded the desired product.

GP5a – Electrochemical reduction of ureas, Gr(+) Gr(-), 5 eq LiCl

To the cathodic half of an oven dried H-cell under an atmosphere of N_2, the urea (1 eq), LiCl (5 eq), $TBAPF_6$ (0.1 M) was added. In the anodic half $TBAPF_6$ (0.1 M) and TBAB (6 eq) were added. To each side DMF (0.1 M) was added. A graphite rod electrode was inserted through the rubber septum of each side of the H cell. The reaction was subjected to electrolysis (3 F mol^{-1}, -6 mA unless stated otherwise). Both sides of the reaction mixture were then combined, diluted with water and extracted with 3 x CH_2Cl_2. The organic layers were dried with anhydrous $MgSO_4$, filtered and concentrated under reduced pressure to afford the crude product. Purification by silica column chromatography afforded the desired product.

GP5b – Electrochemical reduction of ureas, Pt(+) Pt(-)

To the cathodic half of an oven dried H-cell under an atmosphere of N_2, the urea (1 eq) and $TBAPF_6$ (0.1 M) was added. In the anodic half $TBAPF_6$ (0.1 M) and TBAB (6 eq) were added. To each side DMF (0.1 M) was added. A platinum wire electrode was inserted through the rubber septum of each side of the H cell. The reaction was subjected to electrolysis (3 F mol^{-1}, -6 mA unless stated otherwise). Both sides of the reaction mixture were then combined, diluted with water and extracted with 3 x CH_2Cl_2. The organic layers were dried with anhydrous $MgSO_4$, filtered and concentrated under reduced pressure to afford the crude product. Purification by silica column chromatography afforded the desired product.

1.3 Synthesis of Ureas

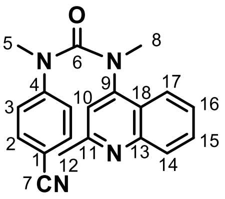

N-(4-Cyanophenyl)-N,N'-dimethyl-N'-(2-methylquinolin-4-yl)urea **(1a)** was synthesised by procedure **GP2a** using 4-cyanophenyl isocyanate (721 mg, 5.0 mmol, 1 eq) and 2-methylquinolin-4-amine (791 mg, 5.0 mmol, 1 eq) with a reaction time of 20 hours. The urea formed was carried forward and methylated by procedure **GP3** without characterisation. Purification by silica column chromatography (50-100% EtOAc in petroleum ether) afforded the desired product (426 mg, 26% over 2 steps) as a white solid.

Alternatively ***N-(4-cyanophenyl)-N,N'-dimethyl-N'-(2-methylquinolin-4-yl)urea*** **(1a)** was synthesised following **GP2b** using *N*,2-dimethylquinolin-4-amine (344 mg, 2.0 mmol, 1 eq) and (4-cyanophenyl)(methyl)carbamic chloride (389 mg, 2.0 mmol, 1 eq) with a reaction time of 92 hours. Purification by silica column chromatography (0-100% EtOAc in petroleum ether) afforded the desired product (240 mg, 36%) as a white solid.

Alternatively ***N-(4-cyanophenyl)-N,N'-dimethyl-N'-(2-methylquinolin-4-yl)urea*** **(1a)** was synthesised by procedure **GP2c** using 2-methylquinolin-4-amine (1582 mg, 10 mmol, 1 eq) followed by 4-aminobenzonitrile (1181 mg, 10 mmol, 1 eq) as the second aniline with a total reaction time of 122 hours. The urea formed was carried forward and methylated by procedure **GP3**, without characterisation. Purification by silica column chromatography (50-80% EtOAc in petroleum ether) afforded the desired product (711 mg, 22% over 2 steps) as a white solid.

^{1}H NMR (400 MHz, $CDCl_3$) δ; 7.91 (1H, d, *J* = 8.4, H17), 7.59-7.67 (2H, m, H14&15), 7.44 (1H, ddd, *J* = 8.4, 6.7, 1.2, H16), 7.14 (2H, d, *J* = 8.6, H2), 6.60-6.65 (3H, m, H3&10), 3.38 (3H, s, H8), 3.15 (3H, s, H5), 2.54 (3H, s, H12)

^{13}C NMR (101 MHz, $CDCl_3$) δ; 160.3 (C6), 159.2 (C11), 149.7 (C9), 149.1 (C13), 148.6 (C4), 132.7 (C2), 130.1 (C15), 129.4 (C17), 126.1 (C16), 125.9 (C3), 122.9 (C18), 122.3 (C14), 119.8 (C10), 118.1 (C7), 108.4 (C1), 39.6 (C8), 38.8 (C5), 25.1 (C12)

IR n$_{max}$ (ATR)/ cm^{-1} 2971 (w), 2225 (m, CN), 1739 (m), 1644 (s, CO), 1599 (s), 1353 (s), 762 (s)

HRMS-ESI (m/z): $[M + H]^+$ calcd for $C_{20}H_{19}N_4O$, 331.1553; found 331.1550

TLC R$_f$ 0.24 (EtOAc) [UV]

Mp = 98 °C

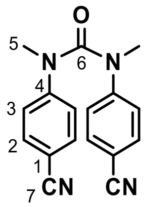

N,N'-Bis(4-cyanophenyl)-N,N'-dimethylurea **(1b)** was synthesised by procedure **GP1b** using 4-aminobenzonitrile (6000 mg, 50.8 mmol, 1 eq) with a reaction time of 20 hrs. The urea formed was carried forward and methylated by procedure **GP3** without characterisation. Purification by silica column chromatography (20-70% EtOAc in petroleum ether) afforded the desired product (4431 mg, 60% over 2 steps) as a white powder.

^{1}H NMR (400 MHz, $CDCl_3$) δ; 7.42 (4H, d, *J* = 8.8, H2), 6.96 (4H, d, *J* = 8.8, H3), 3.26 (9H, s, H5)

^{13}C NMR (101 MHz, $CDCl_3$) δ; 159.3 (C6), 148.7 (C4), 133.1 (C2), 124.4 (C3), 118.3 (C7), 108.2 (C1), 38.4 (C5)

IR n$_{max}$ (ATR)/ cm^{-1} 2938 (w), 2224 (m, CN), 1651 (s, CO), 1601 (s), 1506 (s), 1329 (s), 841 (s)

HRMS-ESI (m/z): $[M + H]^+$ calcd for $C_{17}H_{15}N_4O$, 291.1246; found, 291.1231

TLC R$_f$ 0.26 (50:50 EtOAc/petroleum ether) [UV]

Mp = 188-190 °C

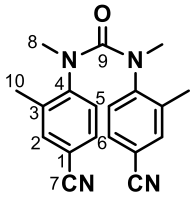

N,N'-Bis(4-cyano-2-methylphenyl)-N,N'-dimethylurea **(1c)** was synthesised by procedure **GP1b** using 4-amino-3-methylbenzonitrile (528 mg, 4 mmol, 1 eq) with a reaction time of 96 hrs. The urea formed was carried forward and methylated by procedure **GP3**, without characterisation. Purification by silica column chromatography (0-50% EtOAc in petroleum ether) afforded the desired product (299 mg, 47% over 2 steps) as a pale yellow solid.

^{1}H NMR (400 MHz, $CDCl_3$) δ; 7.26 (2H, d, *J* = 2.0 Hz, H2), 7.16 (2H, dd, *J* = 8.3, 2.0 Hz, H6), 6.65 (2H, d, *J* = 8.3 Hz, H5), 3.06 (6H, s, H8), 1.99 (6H, s, H10)

^{13}C NMR (101 MHz, $CDCl_3$) δ; 160.7 (C9), 147.8 (C4), 136.8 (C3), 134.7 (C2), 130.5 (C6), 128.3 (C5), 118.1 (C1), 110.4 (C7), 38.5 (C8), 17.3 (C10)

IR υ_{max} (ATR)/ cm^{-1} 2924 (w), 2227 (m, CN), 1651 (s, CO), 1497 (m), 1344 (s), 1095 (m), 602 (s)

HRMS-ESI (m/z): $[M + Na]^+$ calcd for $C_{19}H_{18}N_4NaO$, 341.1373; found, 341.1381

TLC R_f 0.18 (50:50 EtOAc/petroleum ether) [UV]

Mp = 181-182 °C

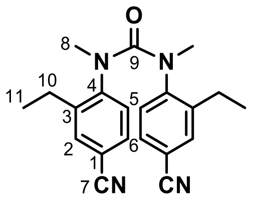

***N,N'-Bis(4-cyano-2-ethylphenyl)-N,N'-dimethylurea* (1d)** was synthesised by procedure **GP1b** using 4-amino-3-ethylbenzonitrile (1742 mg, 10 mmol, 1 eq) with a reaction time of 96 hrs followed by addition of a further 0.08 eq triphoshene and a further 24 hrs. The urea formed was carried forward and methylated by procedure **GP3**, without characterisation. Purification by silica column chromatography (0-60% EtOAc in petroleum ether) afforded the desired product (895 mg, 52% over 2 steps) as a white solid.

^{1}H NMR (400 MHz, $CDCl_3$) δ; 7.33 (2H, d, *J* = 1.9 Hz, H2), 7.17 (2H, dd, *J* = 8.2, 1.9 Hz, H6), 6.63 (2H, d, *J* = 8.2 Hz, H5), 3.07 (6H, s, H8), 2.31 (4H, q, *J* = 7.5 Hz, H10), 1.12 (6H, t, *J* = 7.5 Hz, H11)

^{13}C NMR (101 MHz, $CDCl_3$) δ; 160.9 (C9), 147.3 (C4), 142.4 (C3), 132.7 (C2), 130.3 (C6), 128.5 (C5), 118.3 (C1), 110.3 (C7), 39.2 (C8), 22.9 (C10), 13.6 (C11)

IR υ_{max} (ATR)/ cm^{-1} 2972 (m), 2226 (m, CN), 1643 (s, CO), 1495 (m), 1422 (m), 1325 (s), 1099 (m)

HRMS-ESI (m/z): $[M + Na]^+$ calcd for $C_{21}H_{22}N_4NaO$, 369.1686; found, 369.1701

TLC R_f 0.26 (50:50 EtOAc/petroleum ether) [UV]

Mp = 177-178 °C

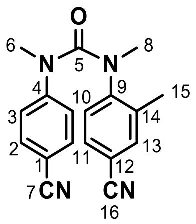

***N*-(4-Cyano-2-methylphenyl)-*N'*-(4-cyanophenyl)-*N,N'*-dimethylurea (1e)** was synthesised following **GP2b** using 3-methyl-4-(methylamino)benzonitrile (292 mg, 2.0 mmol, 1 eq) and (4-cyanophenyl)(methyl)carbamic chloride (311 mg, 1.6 mmol, 0.8 eq) with a reaction time of 48 hours. Purification by silica column chromatography (0-50% EtOAc in petroleum ether) afforded the desired product (209 mg, 43%) as a white solid.

^{1}H NMR (400 MHz, $CDCl_3$) δ; 7.39 (2H, d, *J* = 8.6 Hz, H2), 7.28 (1H, d, *J* = 2.0 Hz, H13), 7.22 (1H, dd, *J* = 8.2, 2.0 Hz, H11), 6.87 (2H, d, *J* = 8.6 Hz, H3), 6.80 (1H, d, *J* = 8.2 Hz, H10), 3.17 (3H, s, H6), 3.14 (3H, s, H8), 2.08 (3H, s, H15)

^{13}**C NMR** (101 MHz, $CDCl_3$) δ; 160.1 (C5), 149.2 (C4), 147.5 (C9), 136.3 (C14), 134.9 (C13), 133.0 (C2), 130.5 (C11), 128.5 (C10), 126.2 (C3), 118.3 (C7), 118.2 (C16), 110.4 (C12), 108.7 (C1), 39.2 (C6), 38.4 (C8), 17.7 (C15)

IR υ$_{max}$ (ATR)/ cm^{-1} 2923 (w), 2225 (m, CN), 1651 (s, CO), 1599 (s), 1345 (s), 1098 (m), 849 (m)

HRMS-ESI (m/z): [M + Na]$^+$ calcd for $C_{18}H_{16}N_4NaO$, 327.1216; found, 327.1227

TLC R$_f$ 0.32 (EtOAc) [UV]

Mp = 140-143 °C

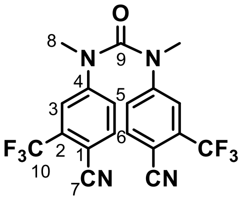

N,N'-Bis(4-cyano-3-(trifluoromethyl)phenyl)-N,N'-dimethylurea **(1f)** was synthesised by procedure **GP1b** using 4-amino-2-(trifluoromethyl)benzonitrile (3723 mg, 20 mmol, 1 eq) with a reaction time of 96 hrs followed by addition of a further 0.08 eq triphoshene and a further 24 hrs. The urea formed was carried forward and methylated by procedure **GP3**, without characterisation. Purification by silica column chromatography (40-80% EtOAc in petroleum ether) afforded a mix of the desired product and singularly methylated product, and so the mixed material was dissolved in chloroform, filtered and the filtrate concentrated under reduced pressure to afford the desired product (1328 mg, 31% over 2 steps) as a pale yellow solid.

^{1}**H NMR** (400 MHz, $CDCl_3$) δ; 7.62, (2H, d, *J* = 8.4 Hz, H6), 7.27 (2H, d, *J* = 2.4 Hz, H3), 7.16 (2H, dd, *J* = 8.4, 2.4 Hz, H5), 3.33 (6H, s, H8)

^{13}**C NMR** (101 MHz, $CDCl_3$) δ; 158.2 (C9), 148.2 (C4), 135.8 (C6), 134.1 (q, *J* = 33.1 Hz, C2), 126.1 (C5), 121.8 (q, *J* = 274.3 Hz, C10), 121.5 (q, J = 5.3 Hz, C3), 114.7 (C1), 105.6 (C7), 38.3 (C8)

^{19}**F NMR** (282 MHz, $CDCl_3$) δ; -62.4

IR υ$_{max}$ (ATR)/ cm^{-1} 3056 (w), 2232 (m, CN), 1669 (s, CO), 1430 (m), 1266 (s), 1174 (s), 1134 (s)

HRMS-ESI (m/z): [M + Na]$^+$ calcd for $C_{19}H_{12}F_6N_4NaO$, 449.0808; found, 449.0833

TLC R$_f$ 0.14 (50:50 EtOAc/petroleum ether) [UV]

Mp = 201-203 °C

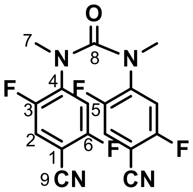

N,N'-Bis(4-cyano-2,5-difluorophenyl)-N,N'-dimethylurea **(1g)** was synthesised by procedure **GP1b** using 4-amino-2,5-difluorobenzonitrile (2312 mg, 4 mmol, 1 eq) with a reaction time of 45 hrs. The urea formed was carried forward and methylated by procedure **GP3**, using DMF as the solvent, without characterisation. Filtration from CH_2Cl_2 afforded the desired product (1155 mg, 43% over 2 steps) as a pale yellow solid.

^{1}H NMR (400 MHz, $CDCl_3$) δ; 7.22 (2H, dd, *J* = 9.3, 5.7 Hz, H2), 6.90 (2H, dd, *J* = 8.9, 6.2 Hz, H5), 3.21 (6H, s, H7)

^{13}C NMR (101 MHz, $CDCl_3$) δ; 159.3 (d, *J* = 262.3, C3), 158.0 (C8), 152.4 (d, *J* = 252.2, C6), 138.2 (m, C4), 120.4 (d, *J* = 26.1 Hz, C2), 115.1 (d, *J* = 22.8 Hz, C5) , 112.1 (C9), 99.5 (m, C1), 38.1 (C7)

^{19}F NMR (282 MHz, $CDCl_3$) δ; -108.8, -122.1

IR n$_{max}$ (ATR)/ cm^{-1} 2955 (w), 2240 (m), 1684 (s), 1626 (m), 1508 (s), 1354 (s), 1181 (w)

HRMS-EI (m/z): [M]$^+$ calcd for $C_{17}H_1F_4N_4O$, 362.0785 ; found, 362.0784

TLC R$_f$ 0.36 (50:50 EtOAc/petroleum ether) [UV]

Mp = 180-182 °C

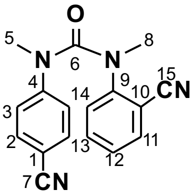

N-(2-Cyanophenyl)-N'-(4-cyanophenyl)-N,N'-dimethylurea **(1h)** was synthesised by procedure **GP2b** using 2-(methylamino)benzonitrile (264 mg, 2.0 mmol, 1.25 eq) and (4-cyanophenyl)(methyl)carbamic chloride (311 mg, 1.6 mmol, 1.0 eq) with a reaction time of 19 hours. Purification by silica column chromatography (0-60% EtOAc in petroleum ether) afforded the desired product (281 mg, 61%) as a cream solid.

^{1}H NMR (400 MHz, $CDCl_3$) δ; 7.49 – 7.23 (4H, m, H2 & H11 & H13/H14), 7.11 – 7.03 (1H, m, H12), 6.98 – 6.81 (3H, m, H3 & H13/14), 3.28 (3H, s, H5/8), 3.25 (3H, s, H5/8)

^{13}C NMR (101 MHz, $CDCl_3$) δ; 159.3 (C6), 148.8 (H4/9), 147.7 (H4/9), 133.5 (ArCH), 133.1 (ArCH), 127.7(ArCH), 126.5 (ArCH), 125.3 (ArCH), 118.4 (C7/15), 116.5 (C7/15), 110.9 (C10), 108.2 (C1), 39.1 (C5/8), 38.9 (C5/8)

IR n$_{max}$ (ATR)/ cm^{-1} 2944 (w), 2224 (s, CN), 1660 (s, CO), 1599 (m), 1342 (s), 847 (s), 763 (s)

HRMS-ESI (m/z): [M + H]$^+$ calcd for $C_{17}H_{14}N_4O$, 291.1240 ; found, 291.1246

TLC R$_f$ 0.17 (50:50 EtOAc/petroleum ether) [UV]

Mp = 126-128 °C

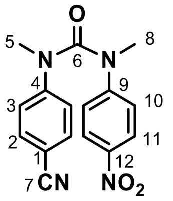

N-(4-Cyanophenyl)-N,N'-dimethyl-N'-(4-nitrophenyl)urea **(1i)** was synthesised by procedure **GP2a** using 4-nitrophenyl isocyanate (820 mg, 5.0 mmol, 1 eq) and 4-aminobenzonitrile (591 mg, 5.0 mmol, 1 eq) with a reaction time of 18 hours. The urea formed was carried forward and methylated by procedure **GP3** without characterisation. Purification by silica column chromatography (50-70% EtOAc in petroleum ether), followed by additional silica column chromatography (0-5% acetone in CH_2Cl_2) afforded the desired product (167 mg, 11% over 2 steps) as a pale yellow solid.

Alternatively ***N-(4-Cyanophenyl)-N,N'-dimethyl-N'-(4-nitrophenyl)urea*** **(1i)** was synthesised following **GP2b** using *N*-methyl-4-nitroaniline (1217 mg, 8.0 mmol, 1.8 eq) and (4-cyanophenyl)(methyl)carbamic chloride (877 mg, 4.5 mmol, 0.1 eq) with a reaction time of 24 hours. Purification by silica column chromatography (0-60% EtOAc in petroleum ether) afforded the desired product (939 mg, 67%) as a pale yellow solid.

^{1}H NMR (400 MHz, $CDCl_3$) δ; 8.01 (2H, d, *J* = 9.1 Hz, H11), 7.43 (2H, d, *J* = 8.7 Hz, H2), 7.01 (d, *J* = 9.1 Hz, H10), 7.01 (2H, d, *J* = 8.7 Hz, H3), 3.30 (3H, s, H5), 3.28 (3H, s, H8)

^{13}C NMR (101 MHz, $CDCl_3$) δ; 159.1 (C6), 150.3 (C9), 148.4 (C4), 143.8 (C12), 133.1 (C2), 124.7 (C11), 124.3 (C3), 123.2 (C10), 118.1 (C7), 108.4 (C1), 38.4 (C5), 38.3 (C8)

IR υmax (ATR)/ cm^{-1} 2937 (w), 2227 (m, CN), 1663 (s, CO), 1592 (s), 1506 (s), 1326 (s), 1099 (s)

HRMS-ESI (m/z): $[M + Na]^+$ calcd for $C_{16}H_{14}N_4O_3Na$, 333.0958; found, 333.0962

TLC R_f 0.28 (50:50 EtOAc/petroleum ether) [UV]

Mp = 144-145 °C

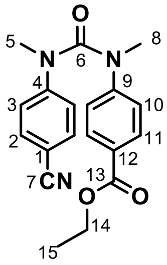

Ethyl 4-(3-(4-cyanophenyl)-1,3-dimethylureido)benzoate **(1j)** was synthesised by procedure **GP2a** using ethyl 4-isocyanatobenzoate (956 mg, 5.0 mmol, 1 eq) and 4-aminobenzonitrile (591 mg, 5.0 mmol, 1 eq) with a reaction time of 40 hours. The urea formed was carried forward and methylated by procedure **GP3** without characterisation. Purification by silica column chromatography (40-80% EtOAc in petroleum ether) afforded the desired product (883 mg, 52% over 2 steps) as a yellow solid.

^{1}H NMR (400 MHz, $CDCl_3$) δ; 7.78 (2H, d, *J* = 8.7, H11), 7.36 (2H, d, *J* = 8.7, H2), 6.95 (2H, d, *J* = 8.7, H3), 6.89 (2H, d, *J* = 8.7, H10), 4.32 (2H, q, *J* = 7.1, H14), 3.28 (3H, s, H5/8), 3.21 (3H, s, H5/8), 1.36 (3H, t, *J* = 7.1, H15)

^{13}C NMR (101 MHz, $CDCl_3$) δ; 165.8 (C13), 159.7 (C6), 148.9 (C4/9), 148.7 (C4/9), 132.9 (C2), 130.6 (C11), 127.1, 124.1 (C2), 124.0 (C3), 118.7 (C7), 107.5 (C1), 61.2 (C14), 38.7 (C5/8), 38.3 (C5/8), 14.4 (C15)

IR n_{max} (ATR)/ cm^{-1} 2971 (w), 2221 (m, CN), 1707 (s, CO ester), 1649 (s, CO urea), 1597 (s), 1274 (s), 1104 (s)

HRMS-ESI (m/z): $[M + H]^+$ calcd for $C_{19}H_{20}N_3O_3$, 338.1499; found 338.1509

TLC R_f 0.37 (50:50 EtOAc/petroleum ether) [UV]

Mp = 169-170 °C

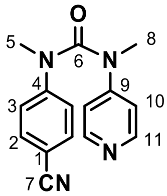

N-(4-Cyanophenyl)-N,N'-dimethyl-N'-(pyridin-4-yl)urea **(1k)** was synthesised following **GP2b** using N-methylpyridin-4-amine (108 mg, 1 mmol, 1 eq) and (4-cyanophenyl)(methyl)carbamic chloride (252 mg, 1.3 mmol, 1.3 eq) with a reaction time of 45 hours. Purification by silica column chromatography (10-40% acetone in CH_2Cl_2) afforded the desired product (210 mg, 79%) as a pale brown solid.

^{1}H NMR (400 MHz, $CDCl_3$) δ; 8.30 (2H, d, *J* = 6.4 Hz, H11), 7.43 (2H, d, *J* = 8.8 Hz, H2), 7.02 (2H, d, *J* = 8.8 Hz, H3), 6.80 (2H, d, *J* = 6.4 Hz, H10), 3.30 (3H, s, H5), 3.21 (3H, s, H8)

^{13}C NMR (101 MHz, $CDCl_3$) δ; 158.9 (C6), 151.3 (C9), 150.5 (C11), 148.4 (C4), 133.2 (C2), 124.2 (C3), 118.3 (C7), 116.6 (C10), 108.4 (C1), 38.3 (C5), 37.3 (C8)

IR υ_{max} (ATR)/ cm^{-1} 2986 (w), 2228 (m, CN), 1668 (s), 1590 (s), 1352 (s), 1266 (s), 701 (s)

HRMS-ESI (m/z): $[M + H]^+$ calcd for $C_{15}H_{15}N_4O$, 267.1249; found, 267.1249

TLC R$_f$ 0.13 (20:80 acetone/CH_2Cl_2) [UV]

Mp = 102-104 °C

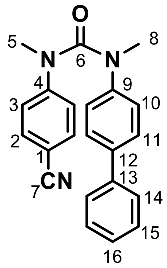

N-([1,1'-Biphenyl]-4-yl)-N'-(4-cyanophenyl)-N,N'-dimethylurea **(1l)** was synthesised by procedure **GP2a** using 4-cyanophenyl isocyanate (432 mg, 3 mmol, 1 eq) and [1,1'-biphenyl]-4-amine (553 mg, 3.3 mmol, 1.1 eq) with a reaction time of 18 hours. The urea formed was carried forward and methylated by procedure **GP3** without characterisation. Purification by silica column chromatography (0-40% EtOAc in petroleum ether) afforded the desired product (767 mg, 75% over 2 steps) as a cream solid.

Alternatively ***N-([1,1'-Biphenyl]-4-yl)-N'-(4-cyanophenyl)-N,N'-dimethylurea*** **(1q)** was synthesised by procedure **GP2c** using 4-aminobiphenyl (846 mg, 5 mmol, 1 eq) followed by 4-aminobenzonitrile (591 mg, 5 mmol, 1 eq) as the second aniline with a total reaction time of 122 hours. The urea formed was carried forward and methylated by procedure **GP3**, without characterisation. Purification by silica column chromatography (0-40% EtOAc in petroleum ether) afforded the desired product (934 mg, 67% over 2 steps) as a cream solid.

^{1}H NMR (400 MHz, $CDCl_3$) δ; 7.43 (4H, m, H10 & H14), 7.33 (5H, m, H2 & H15 & H16), 6.95 (2H, d, *J* = 8.7 Hz, H3), 6.89 (2H, d, *J* = 8.6 Hz, H11), 3.29 (3H, s, H9), 3.18 (3H, s, H5)

^{13}C NMR (101 MHz, $CDCl_3$) δ; 160.2 (C6), 149.4 (C4), 143.9 (C9), 140.5 (C12/C13), 138.7 (C12/C13), 132.8 (C2), 129.0 (C10/C14), 127.7 (C15), 127.6 (C16), 126.9 (C10/C14), 125.8 (C11), 123.9 (C3), 118.8 (C1), 107.0 (C7), 39.2 (C9), 38.2 (C5)

IR ʋmax (ATR)/ cm-1 2918 (w), 2221 (m, CN), 1639 (s, CO), 1599 (s), 1339 (s), 1110 (m), 837 (s)

HRMS-ESI (m/z): [M + Na]+ calcd for $C_{22}H_{19}N_3NaO$, 364.1420; found, 364.1434

TLC Rf 0.18 (50:50 EtOAc/petroleum ether) [UV]

Mp = 117-118 °C

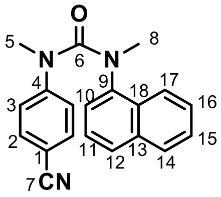

N-(4-Cyanophenyl)-N,N'-dimethyl-N'-(naphthalen-1-yl)urea **(1m)** was synthesised by procedure **GP2a** using 4-cyanophenyl isocyanate (1000 mg, 6.9 mmol, 1 eq) and 1-aminonaphthalene (993 mg, 6.9 mmol, 1 eq) with a reaction time of 26 hours. The urea formed was carried forward and methylated by procedure **GP3** without characterisation. Purification by silica column chromatography (20-50% EtOAc in petroleum ether) afforded the desired product (1813 mg, 83% over 2 steps) as a white powder.

^{1}H NMR (400 MHz, $CDCl_3$) δ; 7.70-7.76 (1H, m), 7.62-7.67 (1H, m), 7.56 (1H, d, *J* = 8.7), 7.41-7.47 (2H, m), 7.18 (1H, dd, *J* = 8.2, 7.3), 7.07 (2H, d, *J* = 8.8, H2), 6.93 (1H, dd, 7.3, 1.1), 6.66 (2H, d, J = 8.8, H3), 3.36 (3H, s, H5/H8), 3.10 (3H, s, H5/8)

^{13}C NMR (101 MHz, $CDCl_3$) δ; 161.1 (C6), 148.9 (C4), 141.3, 134.3, 129.3, 128.5, 127.3, 126.4, 126.3, 125.4 (C3), 125.3, 125.2, 122.5, 118.4 (C7), 107.3 (C1), 39.8 (C5/8), 38.9 (C5/8)

IR nmax (ATR)/ cm-1 3059 (w), 2225 (m, CN), 1644 (s, CO), 1599 (m), 1358 (m), 1112 (m), 773 (s)

HRMS-ESI (m/z): [M + H]+ calcd for $C_{20}H_{18}N_3O$, 316.14499; found 316.1440

TLC Rf 0.44 (70:30 EtOAc/petroleum ether) [UV]

Mp = 142-144 °C

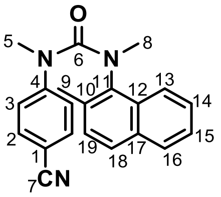

N-(4-Cyanophenyl)-N,N'-dimethyl-N'-(2-methylnaphthalen-1-yl)urea **(1n)** was synthesised by procedure **GP2b** using *N*,2-dimethylnaphthalen-1-amine (684 mg, 4.0 mmol, 1 eq) and (4-cyanophenyl)(methyl)carbamic chloride (946 mg, 4.8 mmol, 1.2 eq) with a reaction time of 88 hours. Purification by silica column chromatography (0-60% EtOAc in petroleum ether) afforded the desired product (925 mg, 70%) as a white solid.

^{1}H NMR (400 MHz, $CDCl_3$) δ; 7.65 (1H, m), 7.54 (1H, m), 7.48 (1H, d, *J* = 8.4 Hz), 7.39 (2H, m), 7.05 (1H, d, *J* = 8.4 Hz), 6.96 (2H, d, *J* = 8.1 Hz, H2), 6.56 (2H, *J* = 8.1 Hz, H3), 3.29 (3H, s, H5 or H8), 3.11 (3H, s, H5 or H8), 2.22 (3H, s, H9)

^{13}C NMR (101 MHz, $CDCl_3$) δ; 160.8 (C6), 148.3 (C4), 137.8 (ArC), 133.4 (ArC), 132.9 (ArC), 132.2 (C2), 129.9 (ArC), 128.6 (ArCH), 128.3 (ArCH), 127.6 (ArCH), 126.5 (ArCH), 125.8 (C3), 125.5 (ArCH), 122.8 (ArC), 118.4 (C7), 108.0 (C1)

IR n$_{max}$ (ATR)/ cm^{-1} 2939 (w), 2224 (m, CN), 1635 (s, CO), 1601 (s), 1356 (s), 1312 (m), 816 (s)

HRMS-ESI (m/z): $[M + H]^+$ calcd for $C_{21}H_{20}N_3O$, 330.1601; found, 330.1599

TLC R$_f$ 0.20 (50:50 EtOAc/petroleum ether) [UV]

Mp = 140-142°C

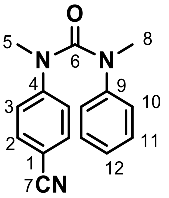

N-(4-Cyanophenyl)-N,N'-dimethyl-N'-phenylurea **(1o)** was synthesised by procedure **GP2a** using 4-cyanophenyl isocyanate (1000 mg, 6.9 mmol, 1 eq) and aniline (646 mg, 6.9 mmol, 1 eq) with a reaction time of 18 hours. The urea formed was carried forward and methylated by procedure **GP3** without characterisation. Purification by silica column chromatography (20-70% EtOAc in petroleum ether) afforded the desired product (1445 mg, 79% over 2 steps) as a white solid.

^{1}H NMR (400 MHz, $CDCl_3$) δ; 7.33 (2H, d, *J* = 8.8, H2), 7.06-7.12 (2H, m, H11), 6.98 (1H, tt, *J* = 7.4, 1.2, H12), 6.91 (2H, d, *J* = 8.8, H3), 6.80-6.84 (2H, m, H10), 3.26 (3H, s, H8), 3.16 (3H, s, H5)

^{13}C NMR (101 MHz, $CDCl_3$) δ; 160.2 (C6), 149.4 (C4), 144.8 (C9), 132.7 (C2), 129.1 (C11), 125.7 (C12), 125.6 (C10), 123.9 (C3), 118.9 (C7), 108.9 (C1)

HRMS-ESI (m/z): $[M + H]^+$ calcd for $C_{16}H_{15}N_3O_3$, 266.1288; found, 266.1291

Data consistent with previous reports[4]

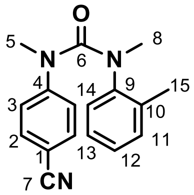

N-(4-Cyanophenyl)-N,N'-dimethyl-N'-(o-tolyl)urea **(1p)** was synthesised by procedure **GP2b** using *N*,2-dimethylaniline (364 mg, 0.371 mL, 3.0 mmol, 1 eq) and (4-cyanophenyl)(methyl)carbamic chloride (759 mg, 3.9 mmol, 1.3 eq) with a reaction time of 44 hours. Purification by silica column chromatography (0-40% EtOAc in petroleum ether) afforded the desired product (550 mg, 66%) as a pale yellow solid.

^{1}H NMR (400 MHz, $CDCl_3$) δ; 7.32 (2H, d, *J* = 8.5 Hz, H2), 6.94 (2H, m, H11/12/13/14), 6.82-6.91 (3H, m, H3 & H11/12/13/14), 6.65 (1H, d, *J* = 7.2 Hz, H11/14), 3.13 (3H, s, H5/8), 3.12 (3H, s, H5/8), 2.03 (3H, s, H15)

^{13}C NMR (101 MHz, $CDCl_3$) δ; 160.7 (C6), 149.6 (C4), 143.1 (C9), 134.7 (C10), 132.7 (C2), 131.1 (C11/12/13/14), 127.8 (C11/12/13/14), 126.8 (C11/12/13/14), 126.7 (C11/12/13/14), 125.0 (C3), 118.8 (C7), 107.7 (C1), 39.0 (C5/8), 38.6 (C5/8), 17.7 (C15)

IR n$_{max}$ (ATR)/ cm^{-1} 2930 (w), 2228 (m, CN), 1656 (s, CO), 1602 (s), 1493 (s), 1342 (s), 1098 (m)

HRMS-ESI (m/z): $[M + Na]^+$ calcd for $C_{17}H_{17}N_3ONa$, 302.1264; found, 302.1279

TLC R_f 0.16 (40:60 EtOAc/petroleum ether) [UV]

Mp = 99-100 °C

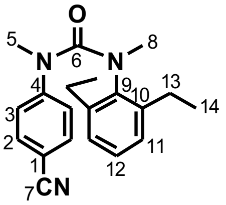

N-(4-Cyanophenyl)-N'-(2,6-diethylphenyl)-N,N'-dimethylurea **(1q)** was synthesised by procedure **GP2a** using 4-cyanophenyl isocyanate (1009 mg, 7 mmol, 1 eq) and 2,6-diethylaniline (0.78 mL, 788 mg, 7.35 mmol, 1.05 eq) with a reaction time of 27 hours. The urea formed was carried forward and methylated by procedure **GP3** without characterisation. Purification by silica column chromatography (0-50% EtOAc in petroleum ether) afforded the desired product (1127 mg, 50% over 2 steps) as a white solid.

^{1}H NMR (400 MHz, $CDCl_3$) δ; 7.29 (2H, d, *J* = 8.5 Hz, H2), 7.01 (1H, t, *J* = 7.6 Hz, H12), 6.84 (2H, d, *J* = 7.6 Hz, H11), 6.76 (2H, d, *J* = 8.5 Hz, H3), 3.13 (3H, s, H8), 3.04 (3H, s, H5), 2.39 (2H, dq, *J* = 15.0, 7.5 Hz, H13a), 2.21 (2H, dq, *J* = 15.0, 7.5 Hz, H13b), 1.13 (6H, t, *J* = 7.5 Hz, H14)

^{13}C NMR (101 MHz, $CDCl_3$) δ; 160.7 (C6), 149.3 (C4), 141.3 (C10), 140.2 (C9), 132.7 (C2), 127.7 (C12), 126.2 (C11), 125.9 (C3), 118.7 (C7), 108.5 (C1), 40.1 (C5), 38.6 (C8), 23.7 (C13), 14.4 (C14)

IR n$_{max}$ (ATR)/ cm^{-1} 2967 (m), 2221 (m, CN), 1651 (s, CO), 1605 (m), 1455 (s), 1346 (s), 1097 (s)

HRMS-ESI (m/z): $[M + H]^+$ calcd for $C_{20}H_{23}N_3O$, 322.1914 ; found, 322.1919

TLC R_f 0.43 (50:50 EtOAc/petroleum ether) [UV]

Mp = 131-132 °C

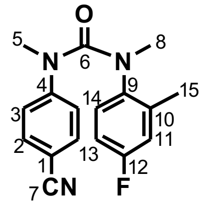

N-(4-Cyanophenyl)-N'-(4-fluoro-2-methylphenyl)-N,N'-dimethylurea **(1r)** was synthesised by procedure **GP2a** using 4-cyanophenyl isocyanate (1009 mg, 7 mmol, 1 eq) and 4-fluoro-2-methylaniline (1.09 mL, 1505 mg, 8.4 mmol, 1.2 eq) with a reaction time of 27 hours. The urea formed was carried forward and methylated by procedure **GP3** without characterisation. Purification by silica column chromatography (0-50% EtOAc in petroleum ether) afforded the desired product (1699 mg, 82% over 2 steps) as a cream solid.

^{1}H NMR (400 MHz, $CDCl_3$) δ; 7.36 (2H, d, *J* = 8.7 Hz, H2), 6.85 (2H, d, *J* = 8.7 Hz, H3), 6.65 (1H, ddt, *J* = 9.2, 2.7, 0.7 Hz, HX), 6.60 – 6.49 (2H, m, HX), 3.11 (3H, s, H5), 3.09 (3H, s, H8), 2.00 (3H, s, H15)

^{13}C NMR (101 MHz, $CDCl_3$) δ; 160.8 (d, *J* = 247.0 Hz, C12), 160.7 (C6), 149.7 (C4), 139.1 (d, *J* = 3.2 Hz, C9), 137.2 (d, *J* = 8.3 Hz, C10), 132.9 (C2), 129.3 (d, *J* = 8.9 Hz, C14), 125.1 (C3), 118.6 (C7), 117.3 (d, *J* = 22.1 Hz, C11), 113.6 (d, J = 22.4 Hz, C13), 108.1 (C1), 39.2 (C5), 38.8 (C8), 17.8 (C15)

^{19}F NMR (282 MHz, $CDCl_3$) δ; -115.0

IR n$_{max}$ (ATR)/ cm^{-1} 2989 (s), 2225 (w, CN), 1658 (m, CO), 1499 (m), 1394 (m), 1066 (s), 869 (w)

HRMS-ESI (m/z): [M + H]$^+$ calcd for $C_{17}H_{16}FN_3O$, 298.1350 ; found, 298.1341

TLC R$_f$ 0.21 (50:50 EtOAc/petroleum ether) [UV]

Mp = 118-120 °C

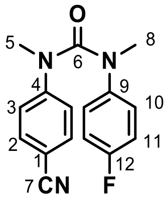

N-(4-Cyanophenyl)-N'-(4-fluorophenyl)-N,N'-dimethylurea **(1s)** was synthesised by procedure **GP2a** using 4-cyanophenyl isocyanate (1000 mg, 6.9 mmol, 1 eq) and 4-fluoroaniline (771 mg, 6.9 mmol, 1 eq) with a reaction time of 4 hours. The urea formed was carried forward and methylated by procedure **GP3** without characterisation. Purification by silica column chromatography (30-50% EtOAc in petroleum ether) afforded the desired product (1254 mg, 64% over 2 steps) as a cream solid.

^{1}H NMR (400 MHz, $CDCl_3$) δ; 7.37 (2H, d, *J* = 8.7, H2), 6.90 (2H, d, J = 8.7, H3), 6.73-6.81 (4H, m, H10&11), 3.21 (3H, s, H8), 3.16 (3H, s, H5)

^{13}C NMR (101 MHz, $CDCl_3$) δ; 160.3 (d, $^1J_{CF}$ = 246.7, C12), 160.2 (C6), 149.4 (C4) 140.9 (d, $^4J_{CF}$ = 3.3, C9), 132.9 (C2), 127.4 (d, $^3J_{CF}$ = 8.5, C10), 124.2 (C3), 118.7 (C7), 115.9 (d, $^2J_{CF}$ = 22.8, C11), 107.3 (C1), 39.5 (C8), 38.4 (C5)

^{19}F NMR (282 MHz, $CDCl_3$) δ; -115.4

IR n$_{max}$ (ATR)/ cm^{-1} 2974 (w), 2229 (m, CN), 1644 (s, CO), 1598 (s), 1504 (s), 1360 (s), 1208 (s)

HRMS-ESI (m/z): [M + H]$^+$ calcd for $C_{16}H_{15}FN_3O$, 284.1199; found 284.1197

TLC R$_f$ 0.37 (70:30 EtOAc/petroleum ether) [UV]

Mp = 100-101 °C

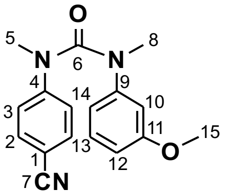

N-(4-Cyanophenyl)-N'-(3-methoxyphenyl)-N,N'-dimethylurea **(1t)** was synthesised by procedure **GP2a** using 4-cyanophenyl isocyanate (1009 mg, 7.0 mmol, 1 eq) and 3-methoxyaniline (0.78 mL, 862 mg, 7.0 mmol, 1.0 eq) with a reaction time of 23 hours. The urea formed was carried forward and methylated by procedure **GP3** without characterisation. Purification by silica column chromatography (0-60% EtOAc in petroleum ether) afforded the desired product (1515 mg, 73% over 2 steps) as a yellow oil.

^{1}H NMR (400 MHz, $CDCl_3$) δ; 7.34 (2H, d, *J* = 8.9 Hz, H2), 7.01 – 6.96 (1H, m, H13), 6.93 (2H, d, *J* = 8.9 Hz, H3), 6.52 (ddd, *J* = 8.4, 2.5, 0.9 Hz, H14), 6.41 (ddd, *J* = 7.9, 2.1, 0.9 Hz, H12), 6.33 (1H, t, *J* = 2.3 Hz, H10), 3.65 (3H, s, H15), 3.23 (3H, s, H8), 3.15 (3H, s, H5).

^{13}**C NMR** (101 MHz, $CDCl_3$) δ; 160.1 (C11), 160.0 (C6), 149.3 (C4), 145.85 (C9), 132.7 (C2), 129.8 (C13), 123.93 (C3), 118.9 (C7), 117.84 (C12), 111.8 (C10), 110.9 (C14), 106.9 (C1), 55.4 (C15), 39.2 (C8), 38.1 (C5)

IR n$_{max}$ (ATR)/ cm^{-1} 3012 (w), 2220 (m, CN), 1652 (s, CO), 1597 (s), 1488 (m), 1347 (m), 837 (m)

HRMS-ESI (m/z): $[M + H]^+$ calcd for $C_{17}H_{17}N_3O_2$, 296.1394 ; found, 296.1399

TLC R$_f$ 0.20 (50:50 EtOAc/petroleum ether) [UV]

Mp = 78-80 °C

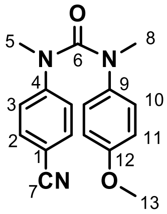

N-(4-Cyanophenyl)-N'-(4-methoxyphenyl)-N,N'-dimethylurea **(1u)** was synthesised by procedure **GP2a** using 4-cyanophenyl isocyanate (1000 mg, 6.9 mmol, 1 eq) and 4-methoxyaniline (854 mg, 6.9 mmol, 1 eq) with a reaction time of 18 hours. The urea formed was carried forward and methylated by procedure **GP3** without characterisation. Purification by silica column chromatography (20-60% EtOAc in petroleum ether) afforded the desired product (1715 mg, 84% over 2 steps) as a pale orange solid.

^{1}**H NMR** (400 MHz, $CDCl_3$) δ; 7.37 (2H, d, *J* = 8.5, H2), 6.92 (2H, d, *J* = 8.5, H3), 6.73 (2H, d, *J* = 8.9, H11), 6.62 (2H, d, *J* = 8.9, H10), 3.71 (3H, s, H13), 3.22 (3H, s, H8), 3.13 (3H, s, H5)

^{13}**C NMR** (101 MHz, $CDCl_3$) δ; 160.5 (C6), 157.5 (C12), 149.6 (C4), 137.7 (C9), 132.8 (C2), 128.0 (C11), 123.7 (C3), 119.0 (C7), 113.9 (C10), 106.7 (C1), 55.6 (C13), 39.7 (C8), 37.8 (C5)

IR n$_{max}$ (ATR)/ cm^{-1} 2934 (w), 2227 (m, CN), 1648 (s, CO), 1599 (s), 1505, (s), 1246 (s), 836 (s)

HRMS-ESI (m/z): $[M + H]^+$ calcd for $C_{17}H_{18}N_3O_2$, 296.1394; found, 296.1399

TLC R$_f$ 0.35 (70:30 EtOAc/petroleum ether) [UV]

Mp = 79-81 °C

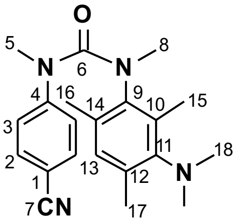

N-(4-Cyanophenyl)-N'-(3-(dimethylamino)-2,4,6-trimethylphenyl)-N,N'-dimethylurea **(1v)** 4-cyanophenyl isocyanate (2883 mg, 20 mmol, 2 eq) was added portion wise to a solution of 2,4,6-trimethylbenzene-1,3-diamine (1502 mg, 10 mmol, 1 eq) in DCM (80 mL), and the reaction mixture stirred overnight at rt. The resulting precipitate was filtered and carried forward and methylated by procedure **GP3**, (6 eq NaH, 8 eq MeI), using DMF as the solvent, without characterisation. Purification by silica column chromatography (0-40% EtOAc in petroleum ether) afforded the desired product (895 mg, 26% over 2 steps) as a pale yellow solid.

^{1}H NMR (400 MHz, $CDCl_3$) δ; 7.29 (2H, d, *J* = 8.6 Hz, H2), 6.80 (2H, d, *J* = 8.6 Hz, H3), 6.58 (1H, s, H13), 3.09 (3H, s, H5), 3.05 (3H, s, H8), 2.63 (6H, br s, H18), 2.10 (3H, s, H17), 1.97 (3H, s, H16), 1.83 (3H, s, H15)

^{13}C NMR (101 MHz, $CDCl_3$) δ; 160.4 (C6), 149.3 (C4), 148.2 (C11), 139.8 (C9), 136.1 (C12), 134.7 (C10), 132.4 (C2), 131.9 (C14), 130.6 (C13), 125.8 (C3), 118.6 (C7), 108.5 (C1), 42.3 (C18), 40.0 (C5), 37.4 (C8), 18.8 (C17), 17.8 (C16), 14.4 (C15)

IR n$_{max}$ (ATR)/ cm^{-1} 2903 (w), 2223 (m, CN), 1637 (s, CO), 1508 (m), 1352 (s), 1069 (m), 850 (m)

HRMS-ESI (m/z): [M + H]$^+$ calcd for $C_{21}H_{26}N_4O$, 351.2179 ; found, 351.2174

TLC R$_f$ 0.28 (50:50 EtOAc/petroleum ether) [UV]

Mp = 111-113 °C

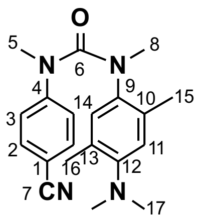

N-(4-Cyanophenyl)-N'-(4-(dimethylamino)-2,5-dimethylphenyl)-N,N'-dimethylurea **(1w)** 4-cyanophenyl isocyanate (2883 mg, 20 mmol, 2 eq) was added portion wise to a solution of 2,5-dimethylbenzene-1,4-diamine (1362 mg, 10 mmol, 1 eq) in DCM (80 mL), and the reaction mixture stirred overnight at rt. The resulting precipitate was filtered and carried forward and methylated by procedure **GP3**, (6 eq NaH, 8 eq MeI), using DMF as the solvent, without characterisation. After removal of solids by filtration from EtOAc, purification by silica column chromatography (0-50% EtOAc in petroleum ether) afforded the desired product (642 mg, 19% over 2 steps) as an orange solid.

^{1}H NMR (400 MHz, $CDCl_3$) δ; 7.30 (2H, d, *J* = 8.7 Hz, H2), 6.82 (2H, d, *J* = 8.7 Hz, H3), 6.52 (1H, s, H11/14), 6.32 (1H, s, H 11/14), 3.12 (3H, s, H5/8), 3.11 (3H, s, H5/8), 2.56 (6H, s, H17), 2.02 (3H, s, H15/16), 1.98 (3H, s, H15/16)

^{13}C NMR (101 MHz, $CDCl_3$) δ; 160.6 (C6), 151.3 (C12), 149.9 (C4), 137.1 (C9), 132.5 (C2), 130.6 (C11/14), 130.3 (C10/13), 125.3 (C3), 124.7 (C10/13), 120.1 (C11/14), 118.7 (C7), 107.5 (C1), 44.1 (C17), 39.1 (C5/8), 38.7 (C5/8), 17.7 (C15/16), 17.5 (C15/16)

IR n$_{max}$ (ATR)/ cm^{-1} 2777 (w), 2226 (m, CN), 1647 (s, CO), 1505 (s), 1349 (s), 1132 (m), 596 (m)

HRMS-ESI (m/z): [M + H]$^+$ calcd for $C_{20}H_{24}N_4O$, 337.2023 ; found, 337.2018

TLC R$_f$ 0.25 (50:50 EtOAc/petroleum ether) [UV]

Mp = 118-121 °C

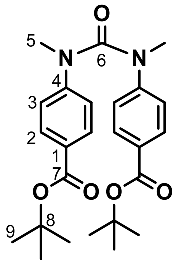

Di-tert-butyl 4,4'-(carbonylbis(methylazanediyl))dibenzoate **(1x)** was synthesised by procedure **GP1b** using *tert*-butyl 4-aminobenzoate (966 mg, 5.0 mmol, 1 eq) with a reaction time of 40 hrs. The urea formed was carried forward and methylated by procedure **GP3** without characterisation. Purification by silica column chromatography (10-50% EtOAc in petroleum ether) afforded the desired product (428 mg, 39% over 2 steps) as a white solid.

^{1}H NMR (400 MHz, $CDCl_3$) δ; 7.71 (4H, d, *J* = 8.7, H2), 6.90 (4H, d, *J* = 8.7, H3), 3.22 (6H, s, H5), 1.54 (18H, s, H9)

^{13}C NMR (101 MHz, $CDCl_3$) δ; 165.1 (C7), 160.2, (C6), 148.7 (C4), 130.2 (C2), 127.9 (C1), 123.1 (C3), 81.0 (C8), 38.5 (C5), 28.2 (C9)

IR n$_{max}$ (ATR)/ cm^{-1} 2971 (w), 1703 (s, CO ester), 1657 (s, CO urea), 1603 (s), 1281 (s), 1106 (s), 702 (m)

HRMS-ESI (m/z): [M + H]$^+$ calcd for $C_{25}H_{33}N_2O_5$, 441.2384; found, 441.2387

TLC R$_f$ 0.27 (30:70 EtOAc/petroleum ether) [UV]

Mp = 158 °C

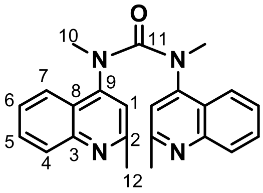

N,N'-Dimethyl-N,N'-bis(2-methylquinolin-4-yl)urea **(1y)** was synthesised by procedure **GP1b** using 2-methylquinolin-4-amine (3164 mg, 20 mmol, 1 eq) with a reaction time of 22 hrs. The urea formed was carried forward and methylated by procedure **GP3** without characterisation. Purification by silica column chromatography (0-50% EtOAc in petroleum ether) afforded the desired product (286 mg, 8% over 2 steps) as a yellow solid.

^{1}H NMR (400 MHz, $CDCl_3$) δ; 7.75 (2H, dd, *J* = 8.5, 1.3 Hz, H7), 7.54 (2H, ddd, *J* = 8.5, 6.8, 1.3 Hz, H6), 7.40 (2H, app. d, *J* = 8.0 Hz, H4), 7.33 (2H, ddd, *J* = 8.0, 6.8, 1.3 Hz, H5), 6.23 (2H, s, H1), 3.30 (3H, s, H10), 2.26 (3H, s, H12)

^{13}C NMR (101 MHz, $CDCl_3$) δ; 160.6 (C11), 158.9 (C2), 148.9 (C3), 148.7 (C9), 129.6 (C6), 129.2 (C7), 125.8 (C5), 123.0 (C8), 121.7 (C4), 120.2 (C1), 39.4 (C10), 24.9 (C12)

IR n$_{max}$ (ATR)/ cm^{-1} 2915 (w), 1616 (m), 1547 (s), 1408 (s), 1328 (s), 1269 (s), 1069 (m)

HRMS-ESI (m/z): [M + H]$^+$ calcd for $C_{23}H_{22}N_4O$, 371.1866; found, 371.1868

TLC R$_f$ 0.21 (5:95 MeOH/CH_2Cl_2) [UV]

Mp = 156-157 °C

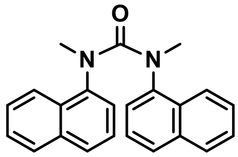

N,N'-Dimethyl- N,N'-di(naphthalen-1-yl)urea **(1z)** was synthesised by procedure **GP1b** using 1-aminonaphthalene (1500 mg, 10.5 mmol, 1 eq) with a reaction time of 4 hrs. The urea formed was carried forward and methylated by procedure **GP3**, using DMF as the solvent, without characterisation. Purification by silica column chromatography (10-30% EtOAc in petroleum ether) afforded the desired product (1182 mg, 66% over 2 steps) as an off white solid.

^{1}H NMR (400 MHz, $CDCl_3$) δ; 7.50 (2H, d, *J* = 8.2, NpH), 6.91-7.46 (8H, br m, NpH), 6.78 (2H, br s, NpH), 6.62 (2H, br s, NpH), 3.29 (6H, s, N-CH_3)

^{13}C NMR (101 MHz, $CDCl_3$) δ; 162.8 (CO), 141.2, 134.2, 129.6, 128.0, 126.3, 125.8, 125.6, 125.3, 125.1, 122.4, 40.0 (N-CH_3)

Data consistent with previous reports[5]

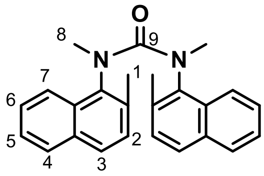

N,N'-Dimethyl-N,N'-bis(2-methylnaphthalen-1-yl)urea **(1aa)** was synthesised by procedure **GP1b** using 2-methylnaphthalen-1-amine (1572 mg, 0.94 mL, 10 mmol, 1 eq) with a reaction time of 96 hrs. The urea formed was carried forward and methylated by procedure **GP3**, without characterisation. Purification by silica column chromatography (0-40% EtOAc in petroleum ether) afforded the desired product (587 mg, 32% over 2 steps) as a cream solid.

^{1}H NMR (400 MHz, $CDCl_3$) δ; 7.66 (0.4x2H, dd, J = 8.3, 1.5 Hz, H4$_A$/7$_\mathbf{A}$), 7.54 (0.4x2H, dd, J = 7.2, 1.6 Hz, H4$_A$/7$_\mathbf{A}$), 7.42 (0.6x2H, d, *J* = 8.1 Hz, H4$_B$/7$_B$), 7.32 (0.4x4H, m, H5$_\mathbf{A}$ & H6$_\mathbf{A}$), 7.20 (0.6x2H, m, H5$_B$/6$_B$), 7.11 (0.4x2H & 0.6x2H, m, H3$_\mathbf{A}$ & H3$_\mathbf{B}$), 6.97 (0.6x4H, m, H4$_B$/7$_\mathbf{B}$ & H5/6$_\mathbf{B}$), 6.77 (0.6x2H, d, *J* = 8.4 Hz, H2$_\mathbf{B}$), 6.60 (0.4x2H, d, *J* = 8.4 Hz, H2$_\mathbf{A}$), 3.21 (0.4x6H, s, H8$_\mathbf{A}$), 3.20 (0.6x6H, s, H8$_\mathbf{B}$), 2.10 (0.6x6H, s, H1$_\mathbf{B}$), 1.52 (0.4x6H, s, H1$_\mathbf{A}$)

^{13}C NMR (101 MHz, $CDCl_3$) δ; 163.3 (C9$_\mathbf{B}$), 163.1 (C9$_\mathbf{A}$), 138.0 (Cquat$_\mathbf{B}$), 137.8 (Cquat$_\mathbf{A}$), 133.2 (Cquat$_\mathbf{A}$), 133.1 (Cquat$_\mathbf{B}$), 132.9(Cquat$_\mathbf{B}$), 132.8(Cquat$_\mathbf{A}$), 130.4 (Cquat$_\mathbf{A}$), 130.3 (Cquat$_\mathbf{B}$), 129.0 (C2$_\mathbf{A}$), 128.4 (C2$_\mathbf{B}$), 127.9 (C4$_A$/7$_\mathbf{A}$), 127.7 (C4$_B$/7$_\mathbf{B}$), 126.3 (C3$_\mathbf{B}$), 126.1 (C3$_\mathbf{A}$), 126.0 (C5/6$_\mathbf{A}$), 125.5 (C4$_B$/5$_B$/6$_B$/7$_\mathbf{B}$), 125.0 (C5$_A$/6$_\mathbf{A}$), 124.7 (C5$_A$/6$_\mathbf{B}$), 122.7 (C4$_A$/7$_\mathbf{A}$), 122.0 (C4$_B$/5$_B$/6$_B$/7$_\mathbf{B}$), 38.3 (C8$_\mathbf{A}$), 38.2 (C8$_\mathbf{B}$), 18.1 (C1$_\mathbf{B}$), 17.1 (C1$_\mathbf{A}$)

IR υ_{max} (ATR)/ cm^{-1} 2962 (w), 1638 (s, CO), 1347 (s), 811 (m), 786 (m), 744 (s)

HRMS-ESI (m/z): $[M + Na]^+$ calcd for $C_{25}H_{24}N_2NaO$, 391.1781; found, 391.1788

TLC R_f 0.35 (30:70 EtOAc/petroleum ether) [UV]

Mp = 142-143 °C

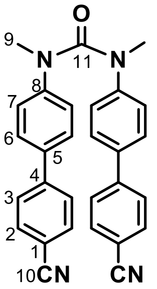

N,N'-Bis(4'-cyano-[1,1'-biphenyl]-4-yl)-N,N'-dimethylurea **(1ab)** was synthesised by procedure **GP1b** using 4'-amino-[1,1'-biphenyl]-4-carbonitrile (777 mg, 4 mmol, 1 eq) with a reaction time of 65 hrs. The urea formed was carried forward and methylated by procedure **GP3**, without characterisation. Filtration from EtOAc afforded the desired product (634 mg, 72% over 2 steps) as a white solid.

^{1}H NMR (400 MHz, $CDCl_3$) δ; 7.67 (4H, d, *J* = 8.4 Hz, H2), 7.52 (4H, d, *J* = 8.4 Hz, H3), 7.31 (4H, d, *J* = 8.5 Hz, H6), 7.00 (4H, d, *J* = 8.5 Hz, H7), 3.26 (6H, s, H9)

^{13}C NMR (101 MHz, $CDCl_3$) δ; 160.7 (C11), 145.9 (C8), 144.7 (C4), 135.6 (C5), 132.7 (C2), 127.6 (C6), 127.4 (C3), 125.8 (C7), 118.9 (C10), 111.0 (C1), 39.1 (C9)

IR n$_{max}$ (ATR)/ cm^{-1} 2226 (m, CN), 1644 (s, CO), 1494 (m), 1355 (m), 1291 (m), 821 (s), 557 (m)

HRMS-ESI (m/z): $[M + H]^+$ calcd for $C_{29}H_{22}N_4O$, 443.1866 ; found, 443.1852

TLC R$_f$ 0.28 (70:30 EtOAc/petroleum ether) [UV]

Mp = 226-227°C

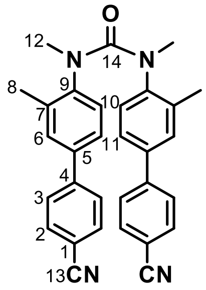

N,N'-Bis(4'-cyano-3-methyl-[1,1'-biphenyl]-4-yl)-N,N'-dimethylurea **(1ac)** was synthesised by procedure **GP1b** using 4'-amino-3'-methyl-[1,1'-biphenyl]-4-carbonitrile (1145 mg, 5.5 mmol, 1 eq) with a reaction time of 39 hrs. The urea formed was carried forward and methylated by procedure **GP3**, without characterisation. Filtration from CH_2Cl_2 afforded the desired product (1193 mg, 92% over 2 steps) as a cream solid.

^{1}H NMR (400 MHz, $CDCl_3$) δ; 7.67 (4H, d, *J* = 8.7 Hz, H2), 7.49 (4H, d, *J* = 8.7 Hz, H3), 7.15 (2H, d, *J* = 2.6 Hz, H6), 7.03 (2H, dd, *J* = 8.2, 2.6 Hz, H11), 6.72 (2H, d, *J* = 8.2 Hz, H10), 3.14 (6H, s, H12), 2.08 (6H, s, H8)

^{13}C NMR (101 MHz, $CDCl_3$) δ; 161.6 (C14), 144.9 (C4), 144.5 (C9), 137.3 (C5), 136.2 (C7), 132.7 (C2), 129.4 (C6), 128.4 (C10), 127.5 (C3), 125.3 (C11), 118.9 (C13), 111.1 (C1), 38.8 (C12), 17.7 (C8)

IR n$_{max}$ (ATR)/ cm^{-1} 2969 (w), 2226 (m, CN), 1630 (s, CO), 1491 (s), 1357 (s), 822 (s), 556 (m)

HRMS-ESI (m/z): $[M + H]^+$ calcd for $C_{31}H_{27}N_4O$, 471.2179 ; found, 471.2172

TLC R$_f$ 0.36 (70:30 EtOAc/petroleum ether) [UV]

Mp = decomposed 250 °C

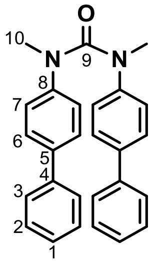

N,N'-Di([1,1'-biphenyl]-4-yl)-N,N'-dimethylurea **(1ad)** was synthesised by procedure **GP1b** using 4-aminobiphenyl (406.1 mg, 2.4 mmol, 1 eq) with a reaction time of 18 hrs. The urea formed was carried forward and methylated by procedure **GP3** without characterisation. Purification by silica column chromatography (0-50% EtOAc in petroleum ether) afforded the desired product (305 mg, 65% over 2 steps) as an orange solid.

^{1}H NMR (400 MHz, $CDCl_3$) δ; 7.27-7.38 (8H, m, H2&3), 7.15-7.26 (6H, m, H6/7&1), 6.82 (4H, d, *J* = 8.5, H6/7), 3.17 (6H, s, H10)

^{13}C NMR (101 MHz, $CDCl_3$) δ; 161.2 (C10), 145.4 (C8), 140.1 (C4), 138.0 (C5), 128.9 (C2/3/6/7), 127.4 (C2/3/6/7), 127.3 (C1), 127.0 (C2/3/6/7), 126.1 (C2/3/6/7), 39.4 (C10)

IR n$_{max}$ (ATR)/ cm^{-1} 3034 (w), 2927 (w), 1738 (m), 1653 (s, CO), 1356 (s), 1107 (m), 733 (s)

HRMS-ESI (m/z): [M + Na]$^+$ calcd for $C_{27}H_{24}N_2ONa$, 415.1781; found, 415.1799

TLC R$_f$ 0.31 (50:50 EtOAc/petroleum ether) [UV]

Mp = 115-116 °C

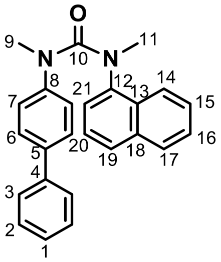

N-([1,1'-Biphenyl]-4-yl)-N,N'-dimethyl-N'-(naphthalen-1-yl)urea **(1ae)** was synthesised by procedure **GP2c** using 4-aminobiphenyl (1354 mg, 8 mmol, 1 eq) followed by naphthalen-1-amine (1146 mg, 8 mmol, 1 eq) as the second aniline with a total reaction time of 50 hours. The urea formed was carried forward and methylated by procedure **GP3**, without characterisation. Purification by silica column chromatography (0-40% EtOAc in petroleum ether) afforded the desired product (2006 mg, 68% over 2 steps) as a pale orange oil.

^{1}H NMR (400 MHz, $CDCl_3$) δ; 7.68 (1H, d, *J* = 7.5 Hz, ArH), 7.64 (1H, d, *J* = 7.2 Hz, ArH), 7.50 (d, *J* = 8.3 Hz, ArH), 7.42-7.25 (5H, m, ArH), 7.20 (2H, m, ArH), 7.13 (1H, dd, *J* = 8.2, 7.3 Hz, ArH), 6.94 (1H, dd, *J* = 7.4, 1.1 Hz, ArH), 6.91 (2H, d, *J* = 8.5 Hz, ArH), 6.54 (2H, d, *J* = 8.5 Hz, ArH), 3.33 (3H, s, H9/11), 3.16 (3H, s, H9/11)

^{13}C NMR (101 MHz, $CDCl_3$) δ; 162.1 (C10), 144.2 (ArC), 142.1 (ArC), 141.1 (ArC), 138.1 (ArC), 134.4 (ArC), 130.2 (ArC), 128.6 (ArCH), 128.2 (ArCH), 127.3 (ArCH), 127.1 (ArCH), 127.1 (ArCH), 126.8 (ArCH), 126.7 (ArCH), 125.9 (ArCH), 125.9 (ArCH), 125.5 (ArCH), 125.3 (ArCH), 123.2 (ArCH), 39.9 (C9/11), 39.8 (C9/11)

IR υ_{max} (ATR)/ cm^{-1}) 2965 (w), 1736 (w), 1648 (s, CO), 1486 (m), 1354 (s), 1115 (w), 1007 (w)

HRMS-ESI (m/z): $[M + H]^+$ calcd for $C_{25}H_{23}N_2O$, 367.1805; found, 367.1801

TLC R_f 0.38 (50:50 EtOAc/petroleum ether) [UV]

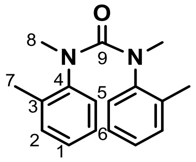

N,N'-Dimethyl-N,N'-di-o-tolylurea **(1af)** was synthesised by procedure **GP1a** using *N*,2-dimethylaniline (1212 mg, 10 mmol, 1 eq) with a reaction time of 17 hrs. Purification by silica column chromatography (0-40% Et_2O in petroleum ether) afforded the desired product (524 mg, 39%) as a white solid.

^{1}H NMR (400 MHz, $CDCl_3$) δ; 6.90 (4H, m, H1 & H2), 6.81 (2H, m, H6), 6.55 (2H, dt, *J* = 7.8, 2.0 Hz, H5), 3.07 (6H, s, H8), 1.95 (6H, s, H7)

^{13}C NMR (101 MHz, $CDCl_3$) δ; 162.2 (C9), 144.0 (C4), 135.3 (C3), 130.2 (C2), 127.6 (C5), 126.6 (C6), 126.3 (C1), 38.8 (C8), 17.3 (C7)

HRMS-ESI (m/z): $[M + Na]^+$ calcd for $C_{17}H_{20}N_2NaO$, 291.1468; found, 291.1482

Data consistent with previous reports[1]

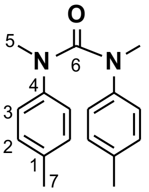

N,N'-Dimethyl-N,N'-di-p-tolylurea **(1ag)** was synthesised by procedure **GP1a** using N,4-dimethylaniline (848 mg, 0.89 mL, 7 mmol, 1 eq) with a reaction time of 66 hrs. Purification by silica column chromatography (0-30% EtOAc in petroleum ether) afforded the title compound (755 mg, 80%) as an off white solid.

^{1}H NMR (400 MHz, $CDCl_3$) δ; 1H NMR (400 MHz, Chloroform-d) δ 6.84 (4H, d, *J* = 8.5 Hz, H2), 6.67 (4H, d, *J* = 8.5 Hz, H3), 3.12 (6H, s, H5), 2.20 (6H, s, H7)

^{13}C NMR (101 MHz, $CDCl_3$) δ; 161.7 (C6), 143.3 (C4), 134.5 (C1), 129.2 (C2), 125.6 (C3), 39.6 (C5), 20.9 (C7)

IR n_{max} (ATR)/ cm^{-1} 2928 (w), 1639 (s), 1510 (s), 1348 (s), 1107 (s), 823 (s), 535 (m)

HRMS-ESI (m/z): $[M + H]^+$ calcd for $C_{17}H_{20}N_2O$, 269.1648 ; found, 269.1653

TLC R_f 0.40 (50:50 EtOAc/petroleum ether) [UV]

Mp = 128-129 °C

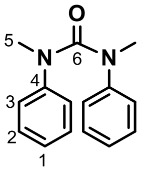

N,N'-Dimethyl-N,N'-diphenylurea **(1ah)** was synthesised by procedure **GP1a** using *N*-methylaniline (1000 mg, 9.3 mmol, 1 eq) with a reaction time of 24 hrs. Purification by silica column chromatography (10-30% EtOAc in petroleum ether) afforded the title compound (750.2 mg, 67%) as an off-white solid.

^{1}H NMR (400 MHz, $CDCl_3$) δ; 7.03 (4H, dd, *J* = 8.4, 7.1, H2), 6.92 (2H, tt, *J* = 7.1, 1.2, H1), 6.79 (4H, dd, *J* = 8.4, 1.2, H3), 3.18 (3H, s, H5)

^{13}C NMR (101 MHz, $CDCl_3$) δ; 161.3 (C6), 145.7 (C4), 128.7 (C2), 125.8 (C3), 124.9 (C1), 39.4 (C5)

HRMS-ESI (m/z): $[M + H]^+$ calcd for $C_{15}H_{17}N_2O$, 241.1341; found, 241.1327

Data consistent with previous reports[6]

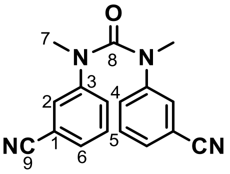

N,N'-Bis(3-cyanophenyl)-N,N'-dimethylurea **(1ai)** was synthesised by procedure **GP1b** using 3-aminobenzonitrile (1500 mg, 12.7 mmol, 1 eq) with a reaction time of 40 hrs. The urea formed was carried forward and methylated by procedure **GP3** without characterisation. Purification by silica column chromatography (20-50% EtOAc in petroleum ether) afforded the desired product (953 mg, 52% over 2 steps) as a white powder.

^{1}H NMR (400 MHz, $CDCl_3$) δ; 7.21-7.25 (2H, m, H4/6), 7.15-7.21 (2H, m, H5), 7.05-7.07 (2H, m, H2), 7.02-7.05 (2H, m, H4/6), 3.19 (6H, s, H7)

^{13}C NMR (101 MHz, $CDCl_3$) δ; 159.6 (C8), 145.8 (C3), 130.0 (C5), 129.7 (C4/6), 128.7 (C4/6), 128.6 (C2), 117.7 (C9), 113.1 (C1), 39.0 (C7)

IR n_{max} (ATR)/ cm^{-1} 2942 (w), 2229 (m, CN), 1647 (s, CO), 1577 (s), 1485 (m), 1349 (s), 799 (s)

HRMS-ESI (m/z): $[M + H]^+$ calcd for $C_{11}H_{15}N_4O$, 291.3335; found 291.1240

TLC R_f 0.33 (70:30 EtOAc/petroleum ether) [UV]

Mp = 134-137 °C

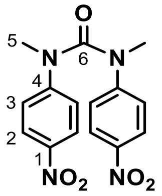

***N,N'*-Dimethyl-*N,N'*-bis(4-nitrophenyl)urea (1aj)** was synthesised by procedure **GP1b** using 4-nitroaniline (1500 mg, 12.7 mmol, 1 eq) with a reaction time of 18 hrs. The urea formed was carried forward and methylated by procedure **GP3** without characterisation. Purification by silica column chromatography (20-70% EtOAc in Petrol ether) afforded the desired product (514 mg, 29% over 2 steps) as a pale yellow solid.

^{1}H NMR (400 MHz, $CDCl_3$) δ; 8.03 (4H, d, *J* = 9.1, H2), 7.05 (4H, d, *J* = 9.1, H3), 3.32 (9H, s, H5)

^{13}C NMR (101 MHz, $CDCl_3$) δ; 159.0 (C6), 150.1 (C4), 143.9 (C1), 124.7 (C2), 123.2 (C3), 38.3 (C5)

HRMS-ESI (m/z): $[M + H]^+$ calcd for $C_{15}H_{15}N_4O_5$, 331.1042; found, 331.1028

Data consistent with previous reports[6]

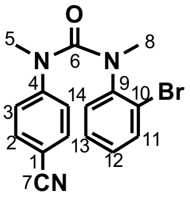

***N-(2-bromophenyl)-N'-(4-cyanophenyl)-N,N'-dimethylurea* (1ak)** was synthesised by procedure **GP2a** using 1-bromo-2-isocyanatobenzene (0.86 mL, 1382 mg, 7.0 mmol, 1 eq) and 4-aminobenzonitrile (868 mg, 7.4 mmol, 1.05 eq) with a reaction time of 24 hours. The urea formed was carried forward and methylated by procedure **GP3** without characterisation. Purification by silica column chromatography (0-50% EtOAc in petroleum ether) afforded the desired product (1287 mg, 53% over 2 steps) as a white solid.

^{1}H NMR (400 MHz, $CDCl_3$) δ; 7.36 – 7.29 (3H, m, H2 & H11/14), 6.99 (1H, ddd, *J* = 7.8, 7.4, 1.5 Hz, H12/13), 6.93 – 6.83 (3H, m, H3 & H12/13), 6.77 (1H, dd, *J* = 7.9, 1.7 Hz, H11/14), 3.18 (3H, s, H5/8), 3.17 (3H, s, H5/8)

^{13}C NMR (101 MHz, $CDCl_3$) δ; 159.8 (C6), 149.2 (C4), 143.2 (C9), 133.6 (C11/14), 132.9 (C2), 129.7 (C11/14), 128.2 (C12/13), 128.0 (C12/13), 125.1 (C3), 122.4, 118.7 (C7), 107.8 (C1), 39.1 (C5/8), 38.5 (C5/8)

IR n$_{max}$ (ATR)/ cm^{-1} 2928 (w), 2224 (m, CN), 1651 (s, CO), 1432 (s), 1354 (s), 1111 (m), 850 (s)

HRMS-ESI (m/z): $[M + H]^+$ calcd for $C_{16}H_{14}N_3OBr$, 344.0393; found 344.0399

TLC R$_f$ 0.31 (50:50 EtOAc/petroleum ether) [UV]

Mp = 144-147 °C

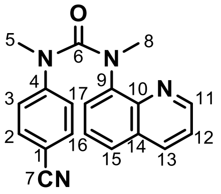

N-(4-Cyanophenyl)-N,N'-dimethyl-N'-(quinolin-8-yl) **(1al)** was synthesised by procedure **GP2c** using 4-aminobenzonitrile (1181 mg, 10 mmol, 1 eq) followed by quinolin-8-amine (1442 mg, 10 mmol, 1 eq) as the second aniline with a total reaction time of 72 hours. The urea formed was carried forward and methylated by procedure **GP3**, without characterisation. Purification by silica column chromatography (0-70% EtOAc in petroleum ether) afforded the desired product (422 mg, 13% over 2 steps) as an orange solid.

^{1}H NMR (400 MHz, $CDCl_3$) δ; 8.86 (1H, dd, *J* = 4.2, 1.7 Hz, H11), 8.02 (1H, dd, *J* = 8.3, 1.7 Hz, H13), 7.49 (1H, dd, *J* = 8.1, 1.4 Hz, H15), 7.36 (1H, dd, *J* = 8.3, 4.2 Hz, H12), 7.24 (1H, dd, *J* = 8.1, 7.4 Hz, H16), 7.16 (1H, dd, *J* = 7.4, 1.4 Hz, H17), 7.08 (1H, d, *J* = 8.6 Hz, H2), 6.74 (1H, d, *J* = 8.6 Hz, H3), 3.42 (3H, s, H8), 3.10 (3H, s, H5)

^{13}C NMR (101 MHz, $CDCl_3$) δ; 161.2 (C6), 150.0 (C11), 149.0 (C4), 143.4 (C10), 142.2 (C9), 136.3 (C13), 132.2 (C2), 129.0 (C14), 127.5 (C17), 126.7 (C15), 126.2 (C16), 123.7 (C3), 121.7 (C12), 118.9 (C7), 106.0 (C1), 39.8 (C8), 37.9 (C5)

IR n$_{max}$ (ATR)/ cm^{-1} 2939 (w), 2215 (m, CN), 1670 (s, CO), 1501 (s), 1331 (s), 1103 (s), 800 (m)

HRMS-ESI (m/z): [M + H]$^+$ calcd for $C_{19}H_{16}N_4O$, 317.1397; found 317.1387

TLC R$_f$ 0.12 (60:40 EtOAc/petroleum ether) [UV]

Mp = 122-123 °C

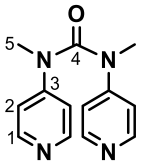

N,N'-Dimethyl-N,N'-di(pyridin-4-yl)urea **(1am)** was synthesised by procedure **GP1a** using *N*-methylpyridin-4-amine (540 mg, 5.0 mmol, 1 equiv) with a reaction time of 24 hrs. Purification by silica column chromatography (0-10% MeOH in CH_2Cl_2) afforded the title compound (282 mg, 48%) as a brown solid.

^{1}H NMR (400 MHz, $CDCl_3$) δ; 8.35 (4H, d, *J* = 6.4, H1), 6.87 (4H, d, *J* = 6.4, H2), 3.26 (6H, s, H5)

^{13}C NMR (101 MHz, $CDCl_3$) δ; 158.8 (C4), 151.1 (C3), 150.8 (C1), 116.5 (C2), 37.3 (C5)

IR n$_{max}$ (ATR)/ cm^{-1} 3028 (w), 1736 (w), 1665 (s, CO), 1580 (s), 1351 (s), 1115 (m), 818 (s)

HRMS-ESI (m/z): [M + H]$^+$ calcd for $C_{13}H_{15}N_4O$, 243.1246; found, 243.1252

TLC R$_f$ 0.31 (10:90 MeOH/CH_2Cl_2) [UV]

R$_f$ = 0.31 in 10% MeOH in dichloromethane

Mp = 113-114 °C

1.4 Biaryl Products

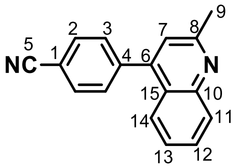

4-(2-Methylquinolin-4-yl)benzonitrile **(2a)** was synthesised by procedure **GP4a** under a nitrogen atmosphere using *N*-(4-cyanophenyl)-*N,N'*-dimethyl-*N'*-(2-methylquinolin-4-yl)urea (50 mg, 0.56 mmol, 1 eq) and the reaction left for 6 hours at RT. Purification by silica column chromatography (0-40% EtOAc in petroleum ether) afforded the desired product (15 mg, 10%) as a pale yellow solid.

Alternatively ***4-(2-methylquinolin-4-yl)benzonitrile*** **(2a)** was synthesised by procedure **GP5a** using *N*-(4-cyanophenyl)-*N,N'*-dimethyl-*N'*-(2-methylquinolin-4-yl)urea (99 mg, 0.30 mmol, 1 eq). Purification by silica column chromatography (0-40% EtOAc in petroleum ether) afforded the desired product (58 mg, 79%) as a pale yellow solid.

^{1}**H NMR** (400 MHz, $CDCl_3$) δ; 8.10 (1H, dt, *J* = 8.3, 1.1, H11/14), 7.82 (2H, d, *J* = 8.6, H2), 7.68-7.76 (2H, m, H11/14 & 12/13), 7.62 (2H, d, *J* = 7.9, H3), 7.47 (1H, ddd, *J* = 8.3, 6.8, 1.3, H12/13), 7.21 (1H, s, H7), 2.78 (3H, s, H9)

^{13}**C NMR** (101 MHz, $CDCl_3$) δ; 158.6 (C8), 148.4 (C10), 146.5 (C6), 143.0 (C4), 132.4 (C2), 130.4 (C3), 129.8 (C12/13), 129.4 (C11/14), 126.4 (C12/13), 124.9 (C11/14), 124.3 (C15), 122.6 (C7), 118.6 (C5), 112.4 (C1), 25.4 (C9)

MS-APCI (m/z): $[M + H]^+$ calcd for $C_{17}H_{13}N_2$, 245.1; found 245.1

Data consistent with previous reports[7]

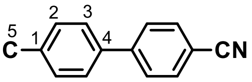

[1,1'-Biphenyl]-4,4'-dicarbonitrile **(2b)** was synthesised by procedure **GP4a** under a nitrogen atmosphere using *N,N'*-bis(4-cyanophenyl)-*N,N'*-dimethylurea (200 mg, 0.69 mmol, 1 eq) and the reaction left at overnight at RT. Purification by silica column chromatography (10-50% EtOAc in petroleum ether) afforded the desired product (99 mg, 70%) as a white solid.

Alternatively ***[1,1'-biphenyl]-4,4'-dicarbonitrile*** **(2b)** was synthesised by procedure **GP4b** under an argon atmosphere using *N,N'*-bis(4-cyanophenyl)-*N,N'*-dimethylurea (145 mg, 0.50 mmol, 1 eq) and the reaction left for 5 hours at RT. Purification by silica column chromatography (10-50% EtOAc in petroleum ether) afforded the desired product (90 mg, 88%) as a white solid.

Alternatively ***[1,1'-biphenyl]-4,4'-dicarbonitrile*** **(2b)** was synthesised by procedure **GP5a** using *N,N'*-bis(4-cyanophenyl)-*N,N'*-dimethylurea (87 mg, 0.30 mmol, 1 eq). Purification by silica column chromatography (10-50% EtOAc in petroleum ether) afforded the desired product (37 mg, 61%) as a white solid.

^{1}**H NMR** (400 MHz, $CDCl_3$) δ; 7.78 (4H, d, *J* = 8.4, H2), 7.69 (4H, d, *J* = 8.4, H3)

^{13}**C NMR** (101 MHz, $CDCl_3$) δ; 143.6 (C4), 133.0 (C2), 128.1 (C3), 118.5 (C5), 112.6 (C1)

MS-APCI (m/z): $[M + H]^+$ calcd for $C_{14}H_9N_2$, 205.1; found 205.1

Data consistent with previous reports[8]

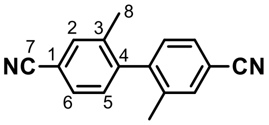

2,2'-Dimethyl-[1,1'-biphenyl]-4,4'-dicarbonitrile **(2c)** was synthesised by procedure **GP5a**, using *N,N'*-bis(4-cyano-2-methylphenyl)-*N,N'*-dimethylurea (96 mg, 0.3 mmol, 1 eq). Purification by silica column chromatography (0-20% EtOAc in petroleum ether) afforded the desired product (49 mg, 71%) as a white solid.

^{1}H NMR (400 MHz, $CDCl_3$) δ; 7.60 (2H, s, H2), 7.56 (2H, dd, *J* = 7.8, 1.7 Hz, H6), 7.17 (2H, d, *J* = 7.8 Hz, H5), 2.07 (6H, s, H8)

^{13}C NMR (101 MHz, $CDCl_3$) δ; 144.6 (C4), 137.1 (C3), 133.8 (C2), 129.8 (C6), 129.6 (C5), 118.7 (C7), 112.2 (C1), 19.7 (C8)

MS-ESI (m/z): $[M + Na]^+$ calcd for $C_{16}H_{12}N_2Na$, 255.1; found 255.0

Data consistent with previous reports[9]

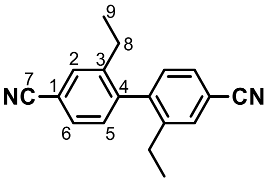

2,2'-Diethyl-[1,1'-biphenyl]-4,4'-dicarbonitrile **(2d)** was synthesised by procedure **GP5a**, using *N,N'*-bis(4-cyano-2-ethylphenyl)-*N,N'*-dimethylurea (104 mg, 0.3 mmol, 1 eq). Purification by silica column chromatography (0-15% EtOAc in petroleum ether) afforded the desired product (53 mg, 68%) as a white solid.

^{1}H NMR (400 MHz, $CDCl_3$) δ; 7.61 (2H, dd, *J* = 1.7, 0.6 Hz, H2), 7.54 (dd, *J* = 7.8, 1.4 Hz, H6), 7.17 (2H, d, *J* = 7.8 Hz, H5), 2.33 (4H, ddt, *J* = 25.3, 14.8, 7.4 Hz, H8a & H8b), 1.05, (6H, t, *J* = 7.4 Hz, H9)

^{13}C NMR (101 MHz, $CDCl_3$) δ; 143.9 (C4), 143.1 (C3), 132.3 (C2), 130.0 (C5), 129.4 (C6), 118.4 (C7), 112.4 (C1), 26.0 C8), 14.6 (C9)

IR υ_{max} (ATR)/ cm^{-1} 2967 (m), 1872 (w), 2228 (s, CN), 1600 (m), 1480 (m), 1062 (m), 844 (s)

HRMS-ESI (m/z): $[M + Na]^+$ calcd for $[M + Na]^+$ calcd for $C_{18}H_{16}N_2Na$, 283.1206; found 283.1213

TLC R_f 0.17 (10:90 EtOAc/petroleum ether) [UV]

Mp = 127-128 °C

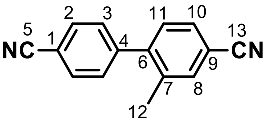

2-Methyl-[1,1'-biphenyl]-4,4'-dicarbonitrile **(2e)** was synthesised by procedure **GP5a**, using *N*-(4-cyano-2-methylphenyl)-*N'*-(4-cyanophenyl)-*N,N'*-dimethylurea (91 mg, 0.3 mmol, 1 eq). Purification by silica column chromatography (0-20% EtOAc in petroleum ether) afforded the desired product (42 mg, 63%) as a cream solid.

^{1}**H NMR** (400 MHz, $CDCl_3$) δ; 7.75 (2H, d, *J* = 8.4 Hz, H2), 7.59 (1H, d, *J* = 1.8 Hz, H8), 7.56 (1H, dd, *J* = 8.0, 1.8 Hz, H10), 7.42 (2H, d, *J* = 8.4 Hz, H3), 7.29 (1H,, *J* = 8.0 Hz, H11), 2.28 (3H, s, H12)

^{13}**C NMR** (101 MHz, $CDCl_3$) δ; 144.8 (C4/6/7), 144.6 (C4/6/7), 136.8 (C4/6/7), 134.2 (C8), 132.4 (C2), 130.3 (C11), 129.9 (C10), 129.7 (C3), 118.7 (C13), 118.6 (C5), 112.3 (C9), 112.0 (C1), 20.3 (C12)

MS-EI (m/z): $[M]^+$ calcd for $C_{15}H_{10}N_2$, 218.1; found 218.1

Data consistent with previous reports[10]

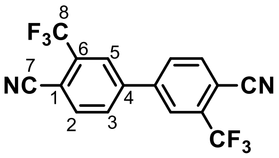

3,3'-Bis(trifluoromethyl)-[1,1'-biphenyl]-4,4'-dicarbonitrile **(2f)** was synthesised by procedure **GP5a**, using *N*,*N'*-bis(4-cyano-3-(trifluoromethyl)phenyl)-*N*,*N'*-dimethylurea (128 mg, 0.3 mmol, 1 eq). Purification by silica column chromatography (0-30% EtOAc in petroleum ether) afforded the desired product (28 mg, 27%) as a pale yellow solid.

^{1}**H NMR** (400 MHz, $CDCl_3$) δ; 8.01 (2H, d, *J* = 8.1 Hz, H2), 8.00 – 7.99 (2H, m, H5), 7.91 (2H, dd, *J* = 8.1, 2.0 Hz, H3)

^{13}**C NMR** (126 MHz, $CDCl_3$) δ; 142.5 (C4), 135.8 (C2), 134.1 (q, *J* = 33.0 Hz, C6), 130.9 (C3), 125.6 (q, *J* = 4.7 Hz, C5), 122.0 (q, *J* = 274.3 Hz, C8), 114.8 (C7), 110.9 (q, *J* = 2.1 Hz, C1)

^{19}**F NMR** (282 MHz, $CDCl_3$) δ; -61.88

MS-EI (m/z): $[M]^+$ calcd for $C_{16}H_6F_6N_2$, 340.0; found 340.0

Data consistent with previous reports[11]

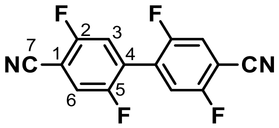

2,2',5,5'-Tetrafluoro-[1,1'-biphenyl]-4,4'-dicarbonitrile **(2g)** was synthesised by procedure **GP5a**, using *N*,*N'*-bis(4-cyano-2,5-difluorophenyl)-*N*,*N'*-dimethylurea (109 mg, 0.3 mmol, 1 eq). Purification by silica column chromatography (0-10% EtOAc in petroleum ether) afforded the desired product (21 mg, 25%) as a white solid.

^{1}**H NMR** (400 MHz, CD_2Cl_2) δ; 7.54 – 7.47 (2H, m, H3/6), 7.34 – 7.28 (2H, m, H3/6)

Unable to obtain carbon data due to poor solubility

^{19}**F NMR** (282 MHz, $CDCl_3$) δ; -110.3, -116.2

IR n$_{max}$ (ATR)/ cm^{-1} 3072 (m), 2245 (m, CN), 1482 (s), 1386 (s), 1178 (s), 899 (s), 788 (s)

HRMS-EI (m/z): $[M]^+$ calcd for $C_{14}H_4F_4N_2$, 276.0305; found 276.0303

TLC R$_f$ 0.34 (20:80 EtOAc/petroleum ether) [UV]

Mp = 231-235 °C

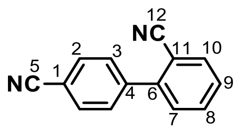

[1,1'-Biphenyl]-2,4'-dicarbonitrile **(2h)** was synthesised by procedure **GP5a**, using *N*-(2-cyanophenyl)- *N'*-(4-cyanophenyl)- *N,N'*-dimethylurea (87 mg, 0.3 mmol, 1 eq). Purification by silica column chromatography (0-30% EtOAc in petroleum ether) afforded the desired product (43 mg, 71%) as a white solid.

^{1}H NMR (400 MHz, $CDCl_3$) δ; 7.82 – 7.75 (3H, m, H2 & H7/8/9/10), 7.72 – 7.63 (3H, m, H3 & H7/8/9/10), 7.55 – 7.47 (2H, m, 2 x H7/8/9/10)

^{13}C NMR (101 MHz, $CDCl_3$) δ; 143.4 (C4/6), 142.6 (C4/6), 134.1 (C7/8/9/10) , 133.3 (C7/8/9/10), 132.6 (C2), 130.0 (C7/8/9/10), 129.7 (C3), 128.9 (C7/8/9/10), 118.5 (C5/12), 118.1 (C5/12), 112.8 (C1/11), 111.4 (C1/11)

MS-EI (m/z): $[M]^+$ calcd for $C_{14}H_8N_2$, 204.1 ; found 204.1

Data consistent with previous reports[12]

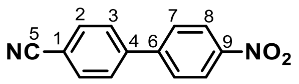

4'-Nitro-[1,1'-biphenyl]-4-carbonitrile **(2i)** was synthesised by procedure **GP5b**, using *N*-(4-cyanophenyl)-*N,N'*-dimethyl-*N'*-(4-nitrophenyl)urea (93 mg, 0.3 mmol, 1 eq). Purification by silica column chromatography (0-20% EtOAc in petroleum ether) afforded the desired product (15 mg, 22%) as an orange solid.

^{1}H NMR (400 MHz, $CDCl_3$) δ; 8.33 (2H, d, *J* = 9.1 Hz, H8), 7.78 (2H, d, *J* = 8.5 Hz, H2), 7.74 (2H, d, *J* = 9.1 Hz, H7), 7.71 (2H, d, *J* = 8.5 Hz, H3)

^{13}C NMR (101 MHz, $CDCl_3$) δ; 148.0 (C9), 145.5 (C6), 143.2 (C4), 133.0 (C2), 128.3 (C3/7), 128.2 (C3/7), 124.5 (C8), 118.4 (C5), 112.8 (C1)

MS-EI (m/z): $[M]^+$ calcd for $C_{13}H_8N_2O_2$, 224.1; found 224.1

Data consistent with previous reports[13]

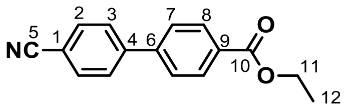

Ethyl 4'-cyano-[1,1'-biphenyl]-4-carboxylate **(2j)** was synthesised by procedure **GP5a**, using ethyl 4-(3-(4-cyanophenyl)-1,3-dimethylureido)benzoate (101 mg, 0.3 mmol, 1 eq). Purification by silica column chromatography (0-30% EtOAc in petroleum ether) afforded the desired product (48 mg, 63%) as a cream solid.

^{1}H NMR (400 MHz, $CDCl_3$) δ; 8.13 (2H, d, *J* = 8.7 Hz, H8), 7.74 (2H, d, *J* = 8.7 Hz, H2), 7.70 (2H, d, *J* = 8.7 Hz, H3), 7.64 (2H, d, *J* = 8.7 Hz, H7), 4.40 (2H, q, *J* = 7.1 Hz, H11), 1.41 (3H, t, *J* = 7.1 Hz, H12)

^{13}C NMR (101 MHz, $CDCl_3$) δ; 166.2 (C10), 144.6 (C4), 143.4 (C6), 132.8 (C2), 130.7 (C9), 130.4 (C8), 128.0 (C3), 127.3 (C7), 118.8 (C5), 111.9 (C1), 61.3 (C11), 14.4 (C12)

MS-EI (m/z): $[M]^+$ calcd for $C_{16}H_{14}NO_2$, 252.1; found 252.1

Data consistent with previous reports[14]

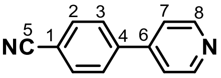

4-(Pyridin-4-yl)benzonitrile **(2k)** was synthesised by procedure **GP5a**, using *N*-(4-cyanophenyl)-*N*,*N'*-dimethyl-*N'*-(pyridin-4-yl)urea (80 mg, 0.3 mmol, 1 eq). An NMR yield of 36% was determined.

^{1}H NMR (400 MHz, $CDCl_3$) δ; 8.72 (2H, d, *J* = 6.2 Hz, H8), 7.79 (2H, d, *J* = 8.7 Hz, H2), 7.74 (2H, d, *J* = 8.7 Hz, H3), 7.51 (2H, d, J = 6.2 Hz, H7)

^{13}C NMR (101 MHz, $CDCl_3$) δ; 150.7 (C8), 146.4 (C6), 142.7 (C4), 133.0 (C2), 127.9 (C3), 121.7 (C7), 118.5 (C5), 112.9 (C1)

MS-EI (m/z): $[M]^+$ calcd for $C_{12}H_8N_2$, 180.1; found 180.1

Data consistent with previous reports[15]

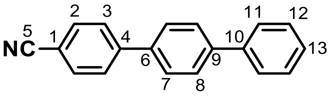

[1,1':4',1''-Terphenyl]-4-carbonitrile **(2l)** was synthesised by procedure **GP5a**, using *N*-([1,1'-biphenyl]-4-yl)-*N'*-(4-cyanophenyl)-*N*,*N'*-dimethylurea (102 mg, 0.3 mmol, 1 eq). Purification by silica column chromatography (0-50% EtOAc in petroleum ether) afforded the desired product (61 mg, 81%) as a pale yellow solid.

^{1}H NMR (400 MHz, $CDCl_3$) δ; 7.74 – 7.61 (10H, m, H2 & H3 & H7 & H8 & H11), 7.49 – 7.43 (2H, m, H12), 7.40 – 7.35 (1H, m, H13)

^{13}C NMR (101 MHz, $CDCl_3$) δ; 145.3 (ArC), 141.7 (ArC), 140.3 (ArC), 138.1 (ArC), 132.8 (ArCH), 129.0 (ArCH), 127.9 (ArCH), 127.8 (C13), 127.7 (ArCH), 127.7 (ArCH), 127.2 (ArCH), 119.0 (C1), 111.0 (C2)

HRMS-ESI (m/z): $[M + H]^+$ calcd for $C_{19}H_{13}N$, 255.1043; found 255.1041

Data consistent with previous reports[16]

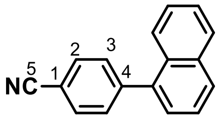

4-(Naphthalen-1-yl)benzonitrile **(2m)** was synthesised by procedure **GP4b** under an argon atmosphere using *N*-(4-cyanophenyl)-*N*,*N'*-dimethyl-*N'*-(naphthalen-1-yl)urea (158 mg, 0.5 mmol, 1 eq) and the reaction left for 1 hour at RT. Purification by silica column chromatography (0-20% EtOAc in petroleum ether) afforded the desired product (30 mg, 26%) as a yellow oil.

Alternatively ***4-(naphthalen-1-yl)benzonitrile*** **(2m)** was synthesised by procedure **GP5a** using *N*-(4-cyanophenyl)-*N*,*N'*-dimethyl-*N'*-(naphthalen-1-yl)urea (95 mg, 0.3 mmol, 1 eq). Purification by silica column chromatography (0-10% EtOAc in petroleum ether) afforded the desired product (56 mg, 82%) as a clear oil.

^{1}H NMR (400 MHz, $CDCl_3$) δ; 7.93 (2H, m, 2xNpH), 7.78 (3H, m, H2 & NpH), 7.62 (2H, m, H3), 7.54 (2H, m, 2xNpH), 7.47 (1H, ddd, *J* = 8.3, 6.8, 1.4 Hz, NpH), 7.40 (1H, dd, *J* = 7.1, 1.2 Hz, NpH)

^{13}**C NMR** (101 MHz, $CDCl_3$) δ; 145.7 (C4), 138.2 (NpC), 133.8 (NpC), 132.2 (C2), 131.0 (NpC), 130.8 (C3), 128.8 (NpCH), 127.1 (NpCH), 126.7 (NpCH), 126.2 (NpCH), 125.4 (NpCH), 125.2 (NpCH), 118.9 (C5), 111.2 (C1)

MS-ESI (m/z): $[M+Na]^+$ calcd for $C_{17}H_{11}NNa$, 252.1; found 252.1

Data consistent with previous reports[17]

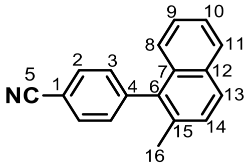

4-(2-Methylnaphthalen-1-yl)benzonitrile **(2n)** was synthesised by procedure **GP5a**, using *N*-(4-cyanophenyl)-*N,N'*-dimethyl-*N'*-(2-methylnaphthalen-1-yl) (99 mg, 0.3 mmol, 1 eq). Purification by silica column chromatography (0-10% EtOAc in petroleum ether) afforded the desired product (52 mg, 71%) as a white solid.

^{1}**H NMR** (400 MHz, $CDCl_3$) δ; 7.86 (1H, d, *J* = 8.5 Hz, NpH), 7.82 (1H, d, *J* = 8.4 Hz, NpH), 7.80 (2H, d, *J* = 8.5 Hz, H2), 7.46 – 7.38 (4H, m, H3 & 2xNpH), 7.35 (1H, ddd, *J* = 8.2, 6.7, 1.4 Hz, NpH), 7.25 (1H, d, *J* = 8.4 Hz, HNpH), 2.21 (3H, s, H16)

^{13}**C NMR** (101 MHz, $CDCl_3$) δ; 145.2 (ArC), 136.2 (ArC), 133.0 (ArC), 132.4 (C2), 132.3 (ArC), 132.0 (ArC), 131.3 (C3), 128.7 (NpH), 128.3 (NpH), 128.1 (NpH), 126.5 (NpH), 125.5 (NpH), 125.2 (NpH), 119.1 (C5), 111.3 (C1), 20.8 (C16)

IR $\mathbf{n_{max}}$ (ATR)/ cm^{-1} 2988 (m), 2227 (s, CN), 1605 (m), 1510 (m), 1379 (m), 909 (m), 811 (s)

HRMS-EI (m/z): $[M]^+$ for $C_{18}H_{13}N$, 243.1043; found 243.1041

TLC $\mathbf{R_f}$ 0.40 (20:80 EtOAc/petroleum ether) [UV]

Mp = 107-119 °C

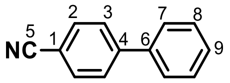

[1,1'-Biphenyl]-4-carbonitrile **(2o)** was synthesised by procedure **GP4a** under a nitrogen atmosphere using *N*-(4-cyanophenyl)-*N,N'*-dimethyl-*N'*-phenylurea (150 mg, 0.56 mmol, 1 eq) and the reaction left at overnight at RT. Purification by silica column chromatography (0-20% EtOAc in petroleum ether) afforded the desired product (10 mg, 10%) as a white solid.

[1,1'-Biphenyl]-4-carbonitrile **(2o)** was synthesised by procedure **GP5b**, using *N*-(4-cyanophenyl)-*N,N'*-dimethyl-*N'*-phenylurea (80 mg, 0.3 mmol, 1 eq). Purification by silica column chromatography (0-10% EtOAc in petroleum ether) afforded the desired product (15 mg, 27%) as a white solid.

^{1}**H NMR** (400 MHz, $CDCl_3$) δ; 7.73 (2H, d, *J* = 8.7, H2), 7.69 (2H, d, *J* = 8.7, H3), 7.57-7.62 (2H, m, H7), 7.40-7.52 (3H, m, H8&9)

^{13}**C NMR** (101 MHz, $CDCl_3$) δ; 145.8 (C4), 139.3 (C6), 133.6 (C2), 129.2 (C8), 128.8 (C9), 127.8 (C3), 127.3 (C7), 119.1 (C5), 111.0 (C1)

MS-APCI (m/z): $[M + H]^+$ calcd for $C_{13}H_{10}N$, 180.1; found 180.1

Data consistent with previous reports[17]

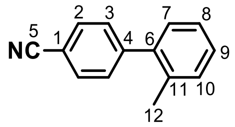

2'-Methyl-[1,1'-biphenyl]-4-carbonitrile **(2p)** was synthesised by procedure **GP5b**, using *N*-(4-cyanophenyl)-*N,N'*-dimethyl-*N'*-(o-tolyl)urea (84 mg, 0.3 mmol, 1 eq). Purification by silica column chromatography (0-10% EtOAc in petroleum ether) afforded the desired product (28 mg, 49%) as a pale orange oil solid.

^{1}H NMR (400 MHz, $CDCl_3$) δ; 7.70 (2H, d, *J* = 8.4 Hz, H2), 7.43 (2H, d, *J* = 8.4 Hz, H3), 7.34 – 7.22 (3H, m, 2 x H7/8/9 & H10), 7.18 (1H, dt, *J* = 7.2, 1.2 Hz, H7/8/9), 2.25 (3H, s, H12)

^{13}C NMR (101 MHz, $CDCl_3$) δ; 146.9 (C4), 140.1 (C6), 135.1 (C11), 132.1 (C2), 130.8 (C10), 130.1 (C3), 129.5 (C7/8/9), 128.4 (7/8/9), 126.2 (C7/8/9), 119.1 (C5), 110.8 (C1), 20.4 (C12)

MS-EI (m/z): $[M+H]^+$ calcd for $C_{14}H_{12}N$, 194.1 ; found 194.1

Data consistent with previous reports[18]

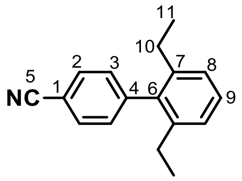

2',6'-Diethyl-[1,1'-biphenyl]-4-carbonitrile **(2q)** was synthesised by procedure **GP5b**, using *N*-(4-cyanophenyl)-*N'*-(2,6-diethylphenyl)-*N,N'*-dimethylurea (96 mg, 0.3 mmol, 1 eq). Purification by silica column chromatography (0-5% EtOAc in petroleum ether) afforded the desired product (35 mg, 50%) as a pale yellow solid.

^{1}H NMR (400 MHz, $CDCl_3$) δ; 7.83 – 7.63 (2H, m, H2), 7.35 – 7.28 (3H, m, H8 & H9), 7.17 (2H, d, J = 7.7 Hz, H3), 2.28 (4H, q, *J* = 7.5 Hz, H10), 1.02 (6H, t, *J* = 7.5 Hz, H11)

^{13}C NMR (101 MHz, $CDCl_3$) δ; 145.9 (C4), 141.7 (C7), 138.9 (C6), 132.1 (C2), 130.6 (C8), 128.5 (C9), 126.0 (C3), 119.0 (C5), 110.9 (C1), 26.8 (C10), 15.6 (C11)

IR n_{max} (ATR)/ cm^{-1} 2966 (s), 2227 (s, CN), 1607 (m), 1453 (s), 1005 (m), 836 (s), 576 (m)

HRMS-EI (m/z): $[M]^+$ calcd for $C_{17}H_{17}N$, 235.1356; found, 235.1357

TLC R_f 0.54 (20:80 EtOAc/petroleum ether) [UV]

Mp = 67-69 °C

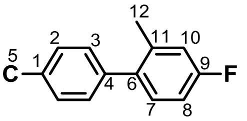

4'-fluoro-2'-methyl-[1,1'-biphenyl]-4-carbonitrile **(2r)** was synthesised by procedure **GP5b**, using *N*-(4-cyanophenyl)-*N'*-(4-fluoro-2-methylphenyl)-*N,N'*-dimethylurea (89 mg, 0.3 mmol, 1 eq). An NMR yield of 35% was determined.

^{1}H NMR (400 MHz, $CDCl_3$) δ; 7.69 (2H, d, *J* = 8.6 Hz, H2), 7.38 (2H, d, *J* = 8.6 Hz, H3), 7.14 (1H, dd, *J* = 8.4, 5.8 Hz, H7), 7.01 – 6.91 (2H, m, H8 & H10), 2.23 (3H, s, H12).

^{13}C NMR (101 MHz, $CDCl_3$) δ; 162.6 (d, *J* = 246.9 Hz, C9), 145.9 (C4), 137.6 (d, *J* = 8.0 Hz, C11), 136.1 (app s, C6), 132.1 (C2), 131.1 (d, *J* = 8.4 Hz, C7), 130.2 (C3), 118.9 (C5), 117.3 (d, *J* = 21.2 Hz, C10), 113.2 (d, *J* = 21.3 Hz, C8), 111.0 (C1), 20.5 (C12)

MS-EI (m/z): $[M]^+$ calcd for $C_{14}H_{10}FN$, 211.1; found 211.1

Data consistent with previous reports[19]

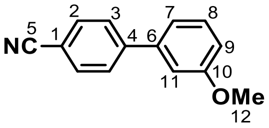

3'-Methoxy-[1,1'-biphenyl]-4-carbonitrile **(2t)** was synthesised by procedure **GP5b**, using *N*-(4-cyanophenyl)-*N'*-(3-methoxyphenyl)-*N,N'*-dimethylurea (89 mg, 0.3 mmol, 1 eq). Purification by silica column chromatography (0-20% EtOAc in petroleum ether) afforded the desired product (25 mg, 40%) as an off white solid.

^{1}H NMR (400 MHz, $CDCl_3$) δ; 7.70 (2H, d, *J* = 8.8 Hz, H2), 7.66 (2H, d, *J* = 8.8 Hz, H3), 7.38 (1H, dd, *J* = 8.2, 7.7 Hz, H8), 7.15 (1H, ddd, *J* = 7.6, 1.7, 0.9 Hz, H7), 7.09 (1H, dd, *J* = 2.5, 1.7 Hz, H11), 6.95 (1H, ddd, *J* = 8.2, 2.6, 0.9 Hz, H9), 3.86 (3H, s, H12)

^{13}C NMR (101 MHz, $CDCl_3$) δ; 160.2 (C10), 145.6 (C4), 140.7 (C6), 132.7 (C2), 130.3 (C8), 127.9 (C3), 119.8 (C7), 119.0 (C5), 114.0 (C9), 113.2 (C12), 111.1 (C1), 55.5 (C12)

MS-EI (m/z): $[M]^+$ calcd for $C_{14}H_{11}NO$, 209.1; found 209.1

Data consistent with previous reports[20]

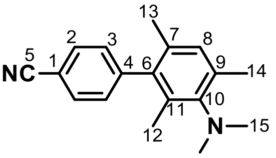

3'-(Dimethylamino)-2',4',6'-trimethyl-[1,1'-biphenyl]-4-carbonitrile **(2v)** was synthesised by procedure **GP5b**, using *N*-(4-cyanophenyl)-*N'*-(3-(dimethylamino)-2,4,6-trimethylphenyl)-*N,N'*-dimethylurea (105 mg, 0.3 mmol, 1 eq). Purification by silica column chromatography (0-10% EtOAc in petroleum ether) afforded the desired product (36 mg, 45%) as a yellow oil.

^{1}H NMR (400 MHz, $CDCl_3$) δ; 7.71 (2H, d, *J* = 8.3 Hz, H2), 7.25 (2H, d, *J* = 8.3 Hz, H3), 6.92 (1H, s, H8), 2.82 (6H, s, H15), 2.31 (3H, s, H13), 1.91 (3H, s, H12/14), 1.90 (3H, s, H12/14)

^{13}C NMR (101 MHz, $CDCl_3$) δ; 147.8 (C10), 147.1 (C4), 138.9 (C6/7/9/11), 137.0 (C6/7/9/11), 135.0 (C6/7/9/11), 132.4 (C2), 131.8 (C6/7/9/11), 130.4 (C3), 130.3 (C8), 119.2 (C5), 110.6 (C1), 42.6 (C18), 20.4 (C12/14), 19.2 (C13), 16.7 (C12/14)

IR $\mathbf{n_{max}}$ (ATR)/ cm^{-1} 2918 (m), 2228 (m, CN), 1679 (m), 1605 (m), 1449 (m), 1112 (m), 842 (s)

HRMS-EI (m/z): $[M]^+$ calcd for $C_{18}H_{20}N_2$, 265.1699; found 265.1689

TLC $\mathbf{R_f}$ 0.47 (petroleum ether) [UV]

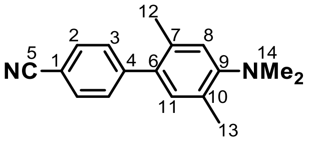

4'-(Dimethylamino)-2',5'-dimethyl-[1,1'-biphenyl]-4-carbonitrile **(2w)** was synthesised by procedure **GP5b**, using *N*-(4-cyanophenyl)-*N'*-(4-(dimethylamino)-2,5-dimethylphenyl)-*N*,*N'*-dimethylurea (101 mg, 0.3 mmol, 1 eq). Purification by silica column chromatography (0-10% EtOAc in petroleum ether) afforded the desired product (38 mg, 50%) as an orange solid.

^{1}H NMR (400 MHz, $CDCl_3$) δ; 7.67 (2H, d, *J* = 8.3 Hz, H2), 7.42 (2H, d, *J* = 8.3 Hz, H3), 7.00 (1H, s, H11), 6.91 (1H, s, H8), 2.74 (6H, s, H14), 2.32 (3H, s, H13), 2.24 (3H, s, H12)

^{13}C NMR (101 MHz, $CDCl_3$) δ; 152.8 (C9), 147.0 (C4), 133.9 (C6), 132.5 (C11), 132.0 (C2), 130.2 (C3), 129.5 (C7), 120.5 (C8), 119.3 (C5), 110.2 (C1), 44.2 (C14), 20.3 (C12), 18.2 (C13)

IR n_{max} (ATR)/ cm^{-1} 2938 (w), 2226 (m, CN), 1605 (s), 1492 (s), 1451 (m), 1107 (s), 842 (s)

HRMS-EI (m/z): $[M]^+$ calcd for $C_{17}H_{18}N_2$, 250.1465; found 240.1464

TLC R_f 0.39 (10:90 EtOAc/petroleum ether) [UV]

Mp = 68-70 °C

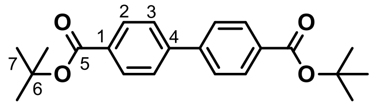

Di-tert-butyl [1,1'-biphenyl]-4,4'-dicarboxylate **(2x)** was synthesised by procedure **GP5b**, using di-tert-butyl 4,4'-(carbonylbis(methylazanediyl))dibenzoate (132 mg, 0.3 mmol, 1 eq). Purification by silica column chromatography (0-30% EtOAc in petroleum ether) afforded the desired product (50 mg, 48%) as a cream solid.

^{1}H NMR (400 MHz, $CDCl_3$) δ; 8.06 (4H, d, *J* = 8.7 Hz, H2), 7.64 (4H, d, *J* = 8.7 Hz, H3), 1.60 (18H, s, H7)

^{13}C NMR (101 MHz, $CDCl_3$) δ; δ 165.6 (C5), 144.1 (C4), 131.6 (C1), 130.1 (C2), 127.2 (C3), 81.3 (C6), 28.3 (C7)

IR n_{max} (ATR)/ cm^{-1} 2977 (m), 1707 (s, CO), 1367 (m), 1289 (s), 1161 (s), 1110 (s), 847 (m)

MS-EI (m/z): $[M]^+$ calcd for $C_{22}H_{26}O_4$, 354.1826; found 354.1829

TLC R_f 0.37 (10:90 EtOAc/petroleum ether) [UV]

Mp = 111-114 °C

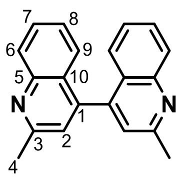

2,2'-Dimethyl-4,4'-biquinoline **(2y)** was synthesised by procedure **GP5a**, using *N*,*N'*-dimethyl-*N*,*N'*-bis(2-methylquinolin-4-yl)urea (111 mg, 0.3 mmol, 1 eq). Purification by silica column chromatography (0-55% EtOAc in petroleum ether) afforded the desired product (55 mg, 65%) as a pale yellow solid.

^{1}H NMR (400 MHz, $CDCl_3$) δ; 8.10 (2H, d, *J* = 8.5 Hz, H6), 7.65 (2H, m, H7), 7.28 (4H, m, H8 & H9), 7.25 (2H, s, H2), 2.78 (6H, s, H4)

^{13}C NMR (101 MHz, $CDCl_3$) δ; 158.6 (C3), 148.1 (C5), 144.7 (C1), 129.9 (C7), 129.2 (C6), 126.3 (C8/9), 125.7 (C8/9), 125.4 (C10), 122.7 (C2), 25.5 (C4)

MS-ESI (m/z): $[M + Na]^+$ calcd for for $C_{20}H_{17}N_2$, 285.1; found 285.1

Data consistent with previous reports[21]

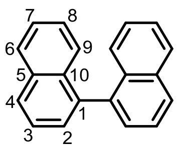

***1,1'-Binaphthalene* (2z)** was synthesised by procedure **GP4b** under an argon atmosphere using *N*,*N'*-dimethyl-*N*,*N'*-di(naphthalen-1-yl)urea (170 mg, 0.5 mmol, 1 eq) and the reaction left for 1 hour at RT. Purification by silica column chromatography (100% petroleum ether) afforded the desired product (48 mg, 38%) as a pale brown solid.

Alternatively ***1,1'-binaphthalene* (2z)** was synthesised by procedure **GP5a** using *N*,*N'*-dimethyl-*N*,*N'*-di(naphthalen-1-yl)urea (102 mg, 0.3 mmol, 1 eq). Purification by silica column chromatography (0-10% EtOAc in petroleum ether) afforded the desired product (56 mg, 74%) as a pale yellow solid.

^{1}H NMR (400 MHz, $CDCl_3$) δ; 7.95 (2H, d, *J* = 8.3 Hz, H4), 7.94 (2H, d *J* = 8.3 Hx, H6), 7.61 (2H, dd, *J* = 8.3, 7.0 Hz, H3), 7.49 (4H, m, H2 & H7), 7.40 (2H, d, *J* = 8.4 Hz, H9), 7.29 (2H, ddd, *J* = 8.4, 6.7, 1.3 Hz, H8)

^{13}C NMR (101 MHz, $CDCl_3$) δ; 138.6 (C1), 133.7 (C5), 133.0 (C10), 128.3 (C4/6), 128.0 (C4/6), 127.9 (C2), 126.7 (C9), 126.1 (C8), 125.9 (C7), 125.5 (C3)

MS-EI (m/z): $[M]^+$ calcd for $C_{20}H_{14}$, 254.1090; found 254.1079

Data consistent with previous reports[8]

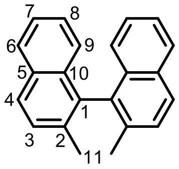

***2,2'-Dimethyl-1,1'-binaphthalene* (2aa)** was synthesised by an adapted procedure **GP5b**, using *N*,*N'*-dimethyl-*N*,*N'*-bis(2-methylnaphthalen-1-yl)urea (111 mg, 0.3 mmol, 1 eq), with LiCl (5 eq) in MeCN. Purification by silica column chromatography (100% petroleum ether) afforded the desired product (30 mg, 36%) as a pale orange oil.

^{1}H NMR (400 MHz, $CDCl_3$) δ; 7.92 (2H, d, *J* = 8.4 Hz, H9), 7.91 (2H, d, *J* = 8.6 Hz, H3/4), 7.53 (2H, d, *J* = 8.6 Hz, H3/4), 7.41 (2H, ddd, *J* = 8.4, 6.8, 1.2 Hz, H8), 7.22 (2H, ddd, *J* = 8.4, 6.8, 1.3 Hz, H7), 7.07 (2H, app. d, *J* = 8.4 Hz, H6), 2.06 (6H, s, H11)

^{13}C NMR (101 MHz, $CDCl_3$) δ; 135.2 (C1/2), 134.4 (C1/2), 132.9 (C5/10), 132.3 (C5/10), 128.9 (C3/4), 128.1 (C9), 127.6 (C3/4), 126.2 (C7), 125.8 (C6), 125.0 (C8), 20.2 (C11)

MS-EI (m/z): $[M]^+$ calcd for $C_{22}H_{18}$, 282.1; found 282.1

Data consistent with previous reports[22]

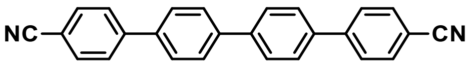

[1,1':4',1'':4'',1'''-Quaterphenyl]-4,4'''-dicarbonitrile **(2ab)** was synthesised by procedure **GP5a**, using *N*,*N'*-bis(4'-cyano-[1,1'-biphenyl]-4-yl)-*N*,*N'*-dimethylurea (133 mg, 0.3 mmol, 1 eq). Filtration of the crude reaction mixture afforded the desired product (82 mg, 77%) as an off-white solid.

^{1}**H NMR** (400 MHz, $CDCl_3$) δ; 7.74 (12H, m, ArH), 7.70 (4H, d, *J* = 8.6 Hz, ArH)

Unable to obtain carbon data due to poor solubility

HRMS-ESI (m/z): $[M + H]^+$ calcd for $C_{26}H_{16}N_2$, 357.1386; found, 357.1389

Data consistent with previous reports[23]

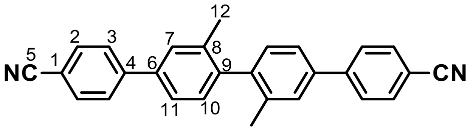

2'',3'-Dimethyl-[1,1':4',1'':4'',1'''-quaterphenyl]-4,4'''-dicarbonitrile **(2ac)** was synthesised by procedure **GP5a**, using *N*,*N'*-bis(4'-cyano-3-methyl-[1,1'-biphenyl]-4-yl)-*N*,*N'*-dimethylurea (141 mg, 0.3 mmol, 1 eq). Purification by silica column chromatography (0-50% EtOAc in petroleum ether) afforded the desired product (98 mg, 85%) as an off white solid.

^{1}**H NMR** (400 MHz, $CDCl_3$) δ; 7.74 (8H, app s, H2 & H3), 7.53 (2H, d, *J* = 1.9 Hz, H7), 7.48 (2H, dd, *J* = 7.8, 2.0 Hz, H11), 7.24 (2H, d, *J* = 7.8 Hz, H10), 2.18 (6H, s, H12)

^{13}**C NMR** (101 MHz, $CDCl_3$) δ; δ 145.5 (C4), 141.5 (C9), 138.4 (C6), 136.9 (C8), 132.7 (C2), 130.2 (C10), 128.9 (H7), 127.8 (C3), 124.7 (C11), 119.1 (C5), 111.0 (C1), 20.2 (C12)

IR $\mathbf{n_{max}}$ (ATR)/ cm^{-1} 2921 (m), 2219 (m, CN), 1601 (m), 1386 (m), 1006 (m), 818 (s), 558 (s)

HRMS-MALDI (m/z): $[M + H]^+$ calcd for $C_{28}H_{20}N_2$, 385.1699; found, 385.1692

TLC $\mathbf{R_f}$ 0.29 (20:80 EtOAc/petroleum ether) [UV]

Mp = 220-222 °C

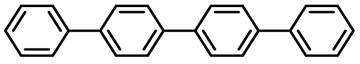

1,1':4',1'':4'',1'''-Quaterphenyl **(2ad)** was synthesised by procedure **GP5b**, using *N*,*N'*-di([1,1'-biphenyl]-4-yl)-*N*,*N'*-dimethylurea (118 mg, 0.3 mmol, 1 eq). Filtration of the crude reaction mixture afforded the desired product (56 mg, 61%) as a pale pink solid.

^{1}**H NMR** (400 MHz, $CDCl_3$) δ; 7.73 (4H, d, *J* = 8.6 Hz, ArH), 7.69 (4H, d, *J* = 8.6 Hz, ArH), 7.67 – 7.63 (4H, m, ArH), 7.46 (4H, t, *J* = 7.6 Hz, ArH), 7.39 – 7.33 (2H, m, ArH)

Unable to obtain carbon data due to poor solubility

MS-ES (m/z): $[M]^+$ calcd for $C_{24}H_{18}$, 306.1; found, 306.1

Data consistent with previous reports[24]

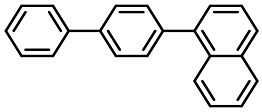

1-([1,1'-Biphenyl]-4-yl)naphthalene **(2ae)** was synthesised by an adapted procedure **GP5b**, using *N*-([1,1'-biphenyl]-4-yl)-*N,N'*-dimethyl-*N'*-(naphthalen-1-yl)urea (110 mg, 0.3 mmol, 1 eq), for 2 F mol^{-1}. Purification by silica column chromatography (0-5% EtOAc in petroleum ether) afforded the desired product (45 mg, 53%) as a white solid.

^{1}H NMR (400 MHz, $CDCl_3$) δ; 8.00 (1h, d, *J* = 8.3 Hz, ArH), 7.93 (1H, d, *J* = 7.4 Hz, ArH), 7.89 (1H, d, *J* = 9.2 Hz, ArH), 7.76 – 7.68 (4H, m, ArH), 7.62 – 7.57 (2H, m, ArH), 7.57 – 7.43 (6H, m, ArH), 7.42 – 7.36 (1H, m, ArH).

^{13}C NMR (101 MHz, $CDCl_3$) δ; 141.0 (ArC), 140.2 (ArC), 140.0 (ArC), 139.9 (ArC), 134.0 (ArC), 131.7 (ArC), 130.6 (ArCH), 129.0 (ArCH), 128.4 (ArCH), 127.8 (ArCH), 127.5 (ArCH), 127.3 (ArCH), 127.1 (ArCH), 127.1 (ArCH), 126.2 (ArCH), 126.1 (ArCH), 125.9 (ArCH), 125.5 (ArCH)

MS-EI (m/z): $[M]^+$ calcd $C_{22}H_{16}$, 280.1 ; found 280.1

Data consistent with previous reports[25]

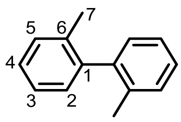

2,2'-Dimethyl-1,1'-biphenyl **(2af)** was synthesised by procedure **GP5b**, using *N,N'*-dimethyl-*N,N'*-di-o-tolylurea (81 mg, 0.3 mmol, 1 eq). Purification by silica column chromatography (0-5% EtOAc in petroleum ether) afforded the desired product (13 mg, 24%) as a clear oil.

^{1}H NMR (400 MHz, $CDCl_3$) δ; 7.28 – 7.24 (4H, m, 2 x H2/3/4), 7.24 – 7.19 (2H, m, H2/3/4), 7.10 (2H, m, H5), 2.05 (6H, s, H7)

^{13}C NMR (101 MHz, $CDCl_3$) δ; 141.7 (C1), 135.9 (C6), 129.9 (C5), 129.4 (C2/3/4), 127.2 (C2/3/4), 125.6 (C2/3/4), 19.9 (C7)

MS-EI (m/z): $[M]^+$ calcd for $C_{14}H_{14}$, 182.1; found 182.1

Data consistent with previous reports[8]

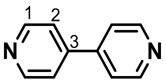

4,4'-Bipyridine **(2am)** was synthesised by procedure **GP4a** under a nitrogen atmosphere using *N,N'*-dimethyl-*N,N'*-di(pyridin-4-yl)urea (169 mg, 0.7 mmol, 1 equiv) and the reaction left at overnight at RT. Purification by silica column chromatography (0-10% MeOH in CH_2Cl_2) afforded the desired product (23 mg, 20%) as a white solid.

Alternatively ***4,4'-bipyridine*** **(2am)** was synthesised by procedure **GP4b** under an argon atmosphere using *N,N'*-dimethyl-*N,N'*-di(pyridin-4-yl)urea (121 mg, 0.50 mmol, 1 equiv) and the reaction left overnight at RT. Purification by silica column chromatography (0-10% MeOH in CH_2Cl_2) afforded the desired product (78 mg, 64%) as a white solid.

^{1}H NMR (400 MHz, $CDCl_3$) δ; 8.73 (4H, d, *J* = 6.2, H1), 7.52 (4H, d, *J* = 6.2, H2)

MS-APCI (m/z): $[M + H]^+$ calcd for $C_{10}H_9N_2$, 157.1; found 157.1

Data consistent with previous reports[8]

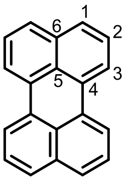

***Perylene* (2an)** was synthesised by procedure **GP4a** under a nitrogen atmosphere using *N,N'*-dimethyl-*N,N'*-di(naphthalen-1-yl)urea (200 mg, 0.58 mmol, 1 equiv) and the reaction left overnight at RT. Purification by silica column chromatography (0-40% EtOAc in petroleum ether) afforded the desired product (3 mg, 4%) as a white solid.

^{1}H NMR (400 MHz, $CDCl_3$) δ; 8.18 (4H, dd, *J* = 7.5, 1.0, H3), 7.68 (4H, dd, *J* = 8.2, 1.0, H1), 7.47 (4H, dd, *J* = 8.2, 7.5, H2)

^{13}C NMR (101 MHz, $CDCl_3$) δ; 134.9 (C6), 131.4 (C4), 129.0 (C5), 128.0 (C2), 126.7 (C1), 120.4 (C3)

Data consistent with previous reports[26]

1.4 Other linkers

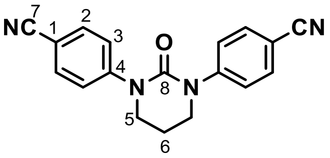

***4,4'-(2-Oxodihydropyrimidine-1,3(2H,4H)-diyl)dibenzonitrile* (4b)** By the method of Tschierske[27] tetrahydropyrimidin-2(1H)-one (400 mg, 4.0 mmol, 2 eq), 4-bromobenzonitrile (364 mg, 2 mmol, 1 eq), caesium carbonate (977 mg, 3 mmol, 1.5 eq), $Pd(dba)_3$ (9.2 mg, 0.01 mmol, 0.5 mol%) and Xantphos (17.4 mg, 0.03 mmol, 1.5 mol%) were refluxed in dioxane (11.1 mL) for 19 hours. Sat NH_4Cl was added and the mixture extracted with CH_2Cl_2. The combined organic layers were dried with anhydrous $MgSO_4$, filtered and concentrated under reduced pressure to afford the crude product. Purification by silica column chromatography (20-100% EtOAc in petroleum ether) afforded the desired product (110 mg, 37%) as a pale yellow solid.

^{1}H NMR (400 MHz, $CDCl_3$) δ; 7.64 (4H, d, *J* = 8.8 Hz, H2), 7.48 (4H, d, *J* = 8.8 Hz, H3), 3.88 (4H, t, *J* = 5.9 Hz, H5), 2.35 (2H, p, *J* = 5.9 Hz, H6)

^{13}C NMR (101 MHz, $CDCl_3$) δ; 153.3 (C8), 147.3 (C4), 132.8 (C2), 125.6 (C3), 118.8 (C7), 108.8 (C1), 48.5 (C5), 22.8 (C6)

IR n_{max} (ATR)/ cm^{-1} 2956 (w), 2218 (m, CN), 1651 (s, CO), 1600 (m), 1477 (s), 1405 (s), 1180 (s)

HRMS-ESI (m/z): $[M + H]^+$ calcd for $C_{18}H_{14}N_4O$, 303.1240; found, 303.1237

TLC R_f 0.26 (80:20 EtOAc/petroleum ether) [UV]

Mp = decomposed 258 °C

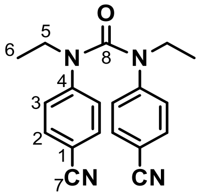

N,N'-Bis(4-cyanophenyl)-N,N'-diethylurea **(5b)** By the method of Clayden[Error! Reference source not found.] the 1,3-bis(4-cyanophenyl)urea (525 mg, 2 mmol, 1 eq) was dissolved in DMF (44 mL) and cooled to 0 °C. NaH (60% dispersion in mineral oil, 240 mg, 6 mmol, 3 eq) was added portion wise and the reaction mixture warmed to rt over 1 hr. EtI (0.64 mL, 8 mmol, 4 eq) was added and the reaction stirred overnight at rt. The reaction mixture was diluted with water and extracted with EtOAc. The organic layers were dried with anhydrous $MgSO_4$, filtered and concentrated under reduced pressure to afford the crude product. Purification by silica column chromatography (0-50% EtOAc in petroleum ether) afforded the title compound (323 mg, 51%) as a pale yellow solid.

^{1}H NMR (400 MHz, $CDCl_3$) δ; 7.34 (4H, d, *J* = 8.8 Hz, H2), 6.84 (4H, d, *J* = 8.8 Hz, H3), 3.69 (4H, q, *J* = 7.1 Hz, H5), 1.15 (6H, t, *J* = 7.1 Hz, H6)

^{13}C NMR (101 MHz, $CDCl_3$) δ; 158.6 (C8), 147.7 (C4), 132.8 (C2), 126.3 (C3), 118.3 (C7), 108.4 (C1), 46.3 (C5), 13.5 (C6)

IR n$_{max}$ (ATR)/ cm^{-1} 2968 (w), 2224 (m, CN), 1658 (s, CO), 1598 (s), 1506 (s), 1252 (s), 832 (s)

HRMS-ESI (m/z): $[M + H]^+$ calcd for $C_{19}H_{19}N_4O$, 319.1553; found, 319.1551

TLC R$_f$ 0.32 (80:20 Et_2O/petroleum ether) [UV]

Mp = 105-107°C

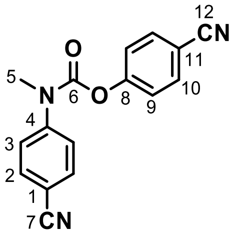

4-Cyanophenyl (4-cyanophenyl)(methyl)carbamate **(6b):** By the method of Snape[28] (4-cyanophenyl)(methyl)carbamic chloride (730 mg, 3.75 mmol, 1.5 eq) was added to a solution of 4-hydroxybenzonitrile (298 mg, 2.5 mmol, 1 eq) in CH_2Cl_2 (25 mL) at 0 °C and the reaction mixture was stirred for 10 mins. Et_3N (0.53 mL, 3.75 mmol, 1.5 eq) and DMAP (30.5 mg, 0.25 mmol, 0.1 eq) were added and the reaction mixture was stirred for 22 hours. The reaction mixture was washed with 1M HCl (3 x 20 mL) and then back extracted with CH_2Cl_2 (2 x 20 mL). The combined organic layers were dried with anhydrous $MgSO_4$, filtered and concentrated under reduced pressure to afford the crude product. The crude product was recrystalised from CH_2Cl_2 with petroleum ether affording the desired product (464 mg, 67%) as a white powder.

^{1}H NMR (400 MHz, $CDCl_3$) δ; 7.68 (2H, d, *J* = 8.8 Hz, H9), 7.66 (2H, d, *J* = 9.0 Hz, H2), 7.48 (2H, d, *J* = 8.8 Hz, H10), 7.25 (2H, d, *J* = 9.0 Hz, H3), 3.48 (3H, s, H5)

^{13}C NMR (101 MHz, $CDCl_3$) δ; 154.2 (C6), 152.2 (C8), 146.4 (C4), 133.7 (C2), 133.2 (C9), 125.8 (C10), 122.6 (C3), 118.3 (C1/C11), 118.2 (C1/C11), 110.2 (C12), 109.8 (C7) 37.9 (C5)

IR υ$_{max}$ (ATR)/ cm^{-1} 2958 (w), 2230 (m, CN), 1733 (s, CO), 1362 (s), 1221 (s), 1112 (s), 546 (s)

HRMS-EI (m/z): [M]+ calcd for $C_{16}H_{11}N_3O_2$, 277.0846; found, 277.0846

TLC R$_f$ 0.28 (50:50 EtOAc/petroleum ether) [UV]

Mp = 184-185 °C

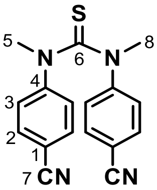

N,N'-Bis(4-cyanophenyl)-N,N'-dimethylthiourea **(7b)** By the method of Mukherjee[29] *N*,*N'*-bis(4-cyanophenyl)-*N*,*N'*-dimethylurea (290 mg, 1 mmol, 1 eq) and Lawessons reagent (769 mg, 1.9 mmol, 1.9 eq) was refluxed in *o*-xylene (4 mL) for 5 hours. The reaction mixture was concentrated under reduced pressure to afford the crude product. Purification by silica column chromatography (0-40% EtOAc in petroleum ether) afforded the desired product (243 mg, 79%) as a pale yellow solid.

^{1}H NMR (400 MHz, $CDCl_3$) δ; 7.39 (4H, d, *J* = 8.6 Hz, H2), 6.80 (2H, d, *J* = 8.6 Hz, H3), 3.53 (6H, s, H5).

^{13}C NMR (101 MHz, $CDCl_3$) δ; 190.6 (C6), 150.7 (C4), 133.1 (C2), 125.2 (C3), 118.1 (C7), 108.9 (c1), 44.3 (C5)

IR n$_{max}$ (ATR)/ cm^{-1} 2940 (w), 2228 (m, CN), 1599 (s), 1502 (s), 1339 (s), 1089 (s), 823 (s)

HRMS-ESI (m/z): [M + H]+ calcd for $C_{17}H_{14}N_4S$, 307.1012 ; found, 307.1015

TLC R$_f$ 0.43 (50:50 EtOAc/petroleum ether) [UV]

Mp = 190-192 °C

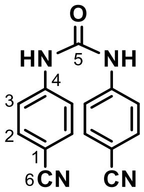

N,N'-Bis(4-cyanophenyl)urea **(8b)** was synthesised by procedure **GP1b** using 4-aminobenzonitrile (4000 mg, 33.9 mmol, 1 eq) with a reaction time of 48 hrs. Filtration of the crude reaction mixture afforded the desired product (4067 mg, 91%) a white solid.

^{1}H NMR (400 MHz, DMSO-d$_6$) δ; 9.42 (2H, s, NH), 7.70 (4H, d, *J* = 8.8 Hz, H2), 7.60 (4H, d, *J* = 8.9 Hz, H3)

^{13}C NMR (101 MHz, $CDCl_3$) δ; 152.3 (C5), 144.2 (C4), 133.9 (C2), 119.8 (C6), 118.9 (C3), 104.4 (C1)

IR n$_{max}$ (ATR)/ cm^{-1} 3378 (s), 2214 (m, CN), 1733 (s, CO), 1590 (s), 1528 (s), 1314 (m), 1175 (s)

HRMS-ESI (m/z): [M + H]+ calcd for $C_{15}H_{10}N_4O$, 263.0927; found 263.0934

TLC R$_f$ 0.20 (70:30 EtOAc/petroleum ether) [UV]

Mp = 294-296 °C

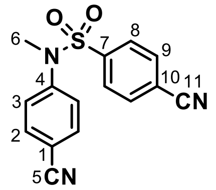

4-Cyano-N-(4-cyanophenyl)-N-methylbenzenesulfonamide (9b): By the method of Flygare[30] 1M LiHMDS in THF (0.85 mL, 0.85 mmol, 1.1 eq) was added to a solution of 4-Cyano-N-(4-cyanophenyl)benzenesulfinamide (220 mg, 0.78 mmol, 1 eq) in THF (7.7 mL). After stirring for 10 mins at room temperature MeI (0.12 mL, 1.9 mmol, 2.5 eq) was added and the reaction mixture stirred overnight. The reaction mixture was concentrated under reduced pressure to afford the crude product. Purification by silica column chromatography (10-40% EtOAc in petroleum ether) afforded the desired product (95 mg, 43%) as an off white solid.

^{1}H NMR (400 MHz, $CDCl_3$) δ; 7.78 (2H, d, *J* = 8.5 Hz, H9), 7.65 (2H, d, *J* = 8.5 Hz, H8), 7.64 (2H, d, *J* = 8.7 Hz, H2), 7.26 (2H, d, *J* = 8.7 Hz, H3), 3.23 (3H, s, H6)

^{13}C NMR (101 MHz, $CDCl_3$) δ; 144.8 (C4), 140.3 (C7), 133.2 (C2), 133.0 (C9), 128.3 (C8), 126.4 (C3), 118.0 (C5), 117.3 (C11), 117.1 (C10), 111.3 (C1), 37.8 (C6)

IR υ_{max} (ATR)/ cm^{-1} 2924 (w), 2229 (m, CN), 1668 (s, CO), 1602 (s), 1502 (s), 1354 (s) 839 (s)

HRMS-ESI (m/z): $[M + Na]^+$ calcd for $C_{15}H_{11}N_3O_2S$, 320.0464; found, 320.0460

TLC R_f 0.28 (30:70 EtOAc/petroleum ether) [UV]

Mp = 136-137 °C

1.5 Synthetic intermediates

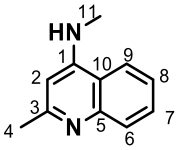

***N,2-Dimethylquinolin-4-amine* (10)**: To a solution of 2-methylquinolin-4-amine (3164 mg, 20 mmol, 1 eq) in CH_2Cl_2 (12 mL) at 0 °C. Triethylamine (2.8 mL, 20 mmol, 1 eq) and ethylchloroformate (2.1 mL, 22 mmol, 1.1 eq) were added and the reaction mixture stirred for 20 hours at room temperature. The reaction mixture was concentrated *in vacuo* and sat. aq. $NaHCO_3$ was added. The resulting precipitate was filtered out affording **ethyl (2-methylquinolin-4-yl)carbamate** (3688 mg) as a cream solid which was carried through to the next step without purification.

A solution of **ethyl (2-methylquinolin-4-yl)carbamate** (3688 mg, 16.0 mmol, 1 eq) in THF (25 mL) was added dropwise to a solution of $LiAlH_4$ (1826 mg, 48.1 mmol, 3 eq) in THF (175 mL) at 0 °C. The reaction mixture was heated to reflux for 4 hours. The reaction mixture was then cooled to 0 °C and quenched with 1M NaOH (12 mL) and water (6 mL) was added. The resulting precipitate was filtered out, chloroform was added and the remaining precipitate filtered out. The filtrate was concentrated *in vacuo* to yield the desired product (1034.9 mg, 30%) as a pale yellow solid.

^{1}**H NMR** (400 MHz, $CDCl_3$) δ; 7.91 (1H, dd, *J* = 8.4, 1.3 Hz, H9), 7.68 (1H, dd, *J* = 8.4, 1.3 Hz, H6), 7.58 (1H, ddd, *J* = 8.4, 6.9, 1.3 Hz, H8), 7.35 (1H, ddd, *J* = 8.4, 6.9, 1.3 Hz, H7), 6.30 (1H, s, H2), 5.23 (1H, br s, NH), 3.02 (3H, d, *J* = 4.9 Hz, H11), 2.62 (3H, s, H4)

^{13}**C NMR** (101 MHz, $CDCl_3$) δ; 159.5 (C3), 150.9 (C1), 147.8 (C5), 129.3 (C8), 128.8 (C9), 124.1 (C7), 119.3 (C6), 117.4 (C10), 98.8 (C2), 30.1 (C11), 25.6 (C4)

MS-ESI (m/z): $[M + H]^+$ calcd for $C_{11}H_{12}N_2$, 173.1; found, 173.1

Data consistent with previous reports[8]

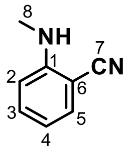

***2-(Methylamino)benzonitrile* (11)** By the method of Cruces[31] 2-aminobenzonitrile (591 mg, 5 mmol 1 eq), pyridine (2.35 mL, 17.5 mmol, 3.5 eq) and $Cu(OAc)_2$ (2270 mg, 12.5 mmol, 2.5 eq) was stirred for 15 mins in dioxane (60 mL). Methylboronic acid (748 mg, 12.5 mmol, 2.5 eq) was added and the reaction mixture refluxed for 24 hours. The reaction mixture was cooled, filtered through celite and concentrated under reduced pressure to afford the crude product. Purification by silica column chromatography (0-30% EtOAc in petroleum ether) afforded the desired product (284 mg, 43%) as a pale yellow solid.

^{1}**H NMR** (400 MHz, $CDCl_3$) δ; 7.43 – 7.31 (2H, m, H3 & H5), 6.71 – 6.59 (2H, m, H2 & H4), 4.61 (1H, br s, NH), 2.91 (3H, s, H8)

^{13}**C NMR** (101 MHz, $CDCl_3$) δ; 151.3 (C1), 134.4 (C3/5), 132.7 (C3/5), 118.1 (C7), 116.5 (C4), 110.2 (C2), 95.7 (C6), 30.1 (C8)

HRMS-ESI (m/z): $[M + H]^+$ calcd for $C_8H_8N_2$, 133.0760 ; found, 133.0758

Data consistent with previous reports[32]

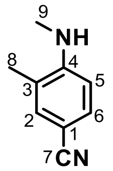

***3-Methyl-4-(methylamino)benzonitrile* (12)** By the method of Zhang[33] 4-amino-3-methylbenzonitrile (1983 mg, 15 mmol, 1 eq) was dissolved in DMF (50 mL) and borane trimethylamine (1204 mg, 16.5 mmol, 1.1 eq) and NaH (60% dispersion in mineral oil) (2520 mg, 63 mmol, 4.2 eq) were added and the reaction stirred at 80 °C for 72 hours. The reaction mixture was diluted with sat. $NaHCO_3$ and extracted with EtOAc (3 x 20 mL), and washed with brine (20 mL). The combined organic layers were dried with anhydrous $MgSO_4$, filtered and concentrated under reduced pressure to afford the crude product. Purification by silica column chromatography (0-30% EtOAc in petroleum ether) afforded the desired product (363 mg, 16%) as a white solid.

^{1}**H NMR** (400 MHz, $CDCl_3$) δ; 7.41 (1H, dd, *J* = 8.5, 2.0 Hz, H5), 7.26 (1H, dd, *J* = 2.0, 0.9 Hz, H2), 6.53 (1H, d, *J* = 8.4 Hz, H6), 4.13 (1H, br s, NH), 2.92 (3H, s, H9), 2.10 (3H, s, H8)

^{13}**C NMR** (101 MHz, $CDCl_3$) δ; 150.5 (C4), 133.2 (C2), 132.3 (C5), 121.9 (C3), 120.8 (C7), 108.6 (C6), 98.3 (C1), 30.3 (C9), 17.1 (C8)

IR n_{max} (ATR)/ cm^{-1} 3400 (m), 2937 (w), 2209 (s, CN), 1605 (s), 1534 (s), 1342 (s), 898 (s)

HRMS-ESI (m/z): $[M + H]^+$ calcd for $C_9H_{10}N_2$, 147.0917 ; found, 147.0914

TLC R_f 0.10 (50:50 EtOAc/petroleum ether) [UV]

Mp = 138-140 °C

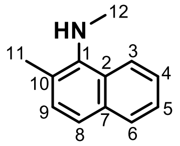

***N,2-Dimethylnaphthalen-1-amine* (13)** By the method of Zhang[33] 2-methylnaphthalen-1-amine (1.41 mL, 2358 mg, 15 mmol, 1 eq) was dissolved in DMF (50 mL) and borane trimethylamine (1204 mg, 16.5 mmol, 1.1 eq) and NaH (60% dispersion in mineral oil) (2520 mg, 63 mmol, 4.2 eq) were added and the reaction stirred at 80 °C for 20 hours. The reaction mixture was diluted with sat. $NaHCO_3$ and extracted with EtOAc (3 x 20 mL), and washed with brine (20 mL). The combined organic layers were dried with anhydrous $MgSO_4$, filtered and concentrated under reduced pressure to afford the crude product. Purification by silica column chromatography (0-10% EtOAc in petroleum ether) afforded the desired product (1264 mg, 49%) as an orange oil.

^{1}H NMR (400 MHz, $CDCl_3$) δ; 8.15 (1H, dd, *J* = 8.5, 1.1 Hz, H3), 7.85 (1H, d, *J* = 8.0 Hz, H6), 7.55 – 7.50 (2H, m, H4 & H8), 7.46 (1H, ddd, *J* = 8.0, 6.8, 1.3 Hz, H5), 7.34 (1H, d, *J* = 8.3 Hz, H9), 3.41 (1H, br s, NH), 2.99 (3H, s, H12), 2.50 (3H, s, H11)

^{13}C NMR (101 MHz, $CDCl_3$) δ; 144.2 (C1), 133.7 (C7), 129.5 (C9), 128.6 (C2), 128.5 (C6), 125.5 (C4/C8), 125.4 (C10), 125.0 (C5), 122.9 (C3), 122.7 (C4/C8), 37.0 (C12), 18.1 (C11)

HRMS-ESI (m/z): $[M + H]^+$ calcd for $C_{12}H_{13}N$, 172.1121 ; found, 172.1117

Data consistent with previous reports[34]

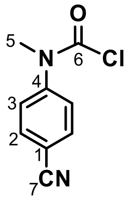

***(4-Cyanophenyl)(methyl)carbamic chloride* (14):** Triphosgene (4451 mg, 15 mmol, 0.5 eq) was added portionwise to CH_2Cl_2 (150 mL) at 0 °C. Pyridine (3.8 mL, 48 mmol, 1.6 eq) was added to the reaction mixture followed by slow addition of 4-(methylamino)benzonitrile (3965 mg, 30 mmol, 1 eq). The reaction mixture was quenched with 1M HCl (80 mL) after 2 hours. The organic layer was separated and washed with sat $NaHCO_3$ (60 mL). The organic layer was dried with anhydrous $MgSO_4$, filtered and concentrated under reduced pressure to afford the desired product (4569 mg, 78%) as a white solid.

^{1}H NMR (400 MHz, $CDCl_3$) δ; 7.73 (2H, d, *J* = 8.6 Hz, H2), 7.41 (2H, d, *J* = 8.6 Hz, H3), 3.43 (3H, s, H5)

^{13}C NMR (101 MHz, $CDCl_3$) δ; 149.1 (C6), 146.7 (C4), 134.0 (C2), 128.1 (C3), 118.0 (C7), 112.2 (C1), 40.6 (C5)

IR υ_{max} (ATR)/ cm^{-1} 2901 (w), 2230 (m, CN), 1730 (s, CO), 1505 (m), 1255 (s), 856 (s), 577 (s)

HRMS-ESI (m/z): $[M + Na]^+$ calcd for $C_9H_7N_2NaOCl$, 217.0139; found, 217.0142

TLC R$_f$ 0.38 (20:80 EtOAc/petroleum ether) [UV]

Mp = 112-113 °C

Data consistent with previous reports[35]

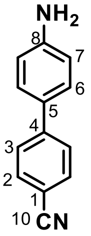

***4'-Amino-[1,1'-biphenyl]-4-carbonitrile* (15)** By the method of Yuan[36] 4-iodoaniline (3943 mg, 18 mmol, 1 eq), (4-cyanophenyl)boronic acid (3174 mg, 21.6 mmol, 1.2 eq), Na_2CO_3 (3816 mg, 36 mmol, 2 eq) and $PdCl_2(PPh_3)_2$ (632 mg, 0.9 mmol, 5 mol%) were refluxed in a 3:1 mixture of dioxane and water (180 mL) for 72 hours. The reaction mixture was cooled, diluted with sat NH_4Cl (50 mL) and extracted with EtOAc (3 x 100 mL). The combined organic layers were dried with anhydrous $MgSO_4$, filtered and concentrated under reduced pressure to afford the crude product. Purification by silica column chromatography (0-30% EtOAc in petroleum ether) afforded the desired product (542 mg, 16%) as an off white solid.

^{1}H NMR (400 MHz, $CDCl_3$) δ; 7.66 (2H, d, *J* = 8.6 Hz, H2), 7.61 (2H, d, J = 8.6 Hz, H3), 7.43 (2H, d, J = 8.5 Hz, H6), 6.77 (2H, d, J = 8.5 Hz, H7), 3.85 (2H, s, NH_2)

^{13}C NMR (101 MHz, $CDCl_3$) δ; 147.3 (C8), 145.6 (C4), 132.6 (C2), 129.1 (C5), 128.3 (C6), 126.7 (C3), 119.4 (C10), 115.5 (C7), 109.6 (C1)

IR n$_{max}$ (ATR)/ cm^{-1} 3360 (m), 2223 (m), 1630 (m, CN), 1592 (s), 1492 (s), 1276 (m), 1188 (m)

HRMS-ESI (m/z): $[M + H]^+$ calcd for $C_{13}H_{11}N_2$, 195.0917; found, 195.0915

TLC R$_f$ 0.30 (50:50 EtOAc/petroleum ether) [UV]

Mp = 172-175°C

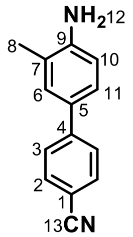

***4'-Amino-3'-methyl-[1,1'-biphenyl]-4-carbonitrile* (16)** By the method of Yuan[36] 4-iodo-2-methylaniline (4195 mg, 18 mmol, 1 eq), (4-cyanophenyl)boronic acid (3174 mg, 21.6 mmol, 1.2 eq), Na_2CO_3 (3816 mg, 36 mmol, 2 eq) and $PdCl_2(PPh_3)_2$ (632 mg, 0.9 mmol, 5 mol%) were refluxed in a 3:1 mixture of dioxane and water (180 mL) for 72 hours. The reaction mixture was cooled, diluted with sat NH_4Cl (50 mL) and extracted with EtOAc (3 x 100 mL). The combined organic layers were dried with anhydrous $MgSO_4$, filtered and concentrated under reduced pressure to afford the crude product. Purification by silica column chromatography (0-40% EtOAc in petroleum ether) afforded the desired product (2113 mg, 56%) as a pale orange solid.

^{1}**H NMR** (400 MHz, $CDCl_3$) δ; 7.65 (2H, d, *J* = 8.6 Hz, H2), 7.61 (2H, d, *J* = 8.6 Hz, H3), 7.31 (2H, m, H6 & H11), 6.76 (2H, d, *J* = 8.0 Hz, H10), 3.81 (2H, br s, H12), 2.24 (3H, s, H8)

^{13}**C NMR** (101 MHz, $CDCl_3$) δ; 145.8 (C4), 145.6 (C9), 132.6 (C2), 129.4 (C6), 129.1 (C5), 126.7 (C3), 126.0 (C11), 122.8 (C7), 119.5 (C13), 115.3 (C10), 109.4 (C1), 17.6 (C8)

IR n$_{max}$ (ATR)/ cm^{-1} 3215 (w), 2224 (m, CN), 1640 (w), 1592 (m), 1487 (m), 1293 (m), 813 (s)

HRMS-ESI (m/z): $[M + H]^+$ calcd for $C_{14}H_{13}N_2$, 209.1073 ; found, 209.1070

TLC R$_f$ 0.35 (50:50 EtOAc/petroleum ether) [UV]

Mp = 135-137°C

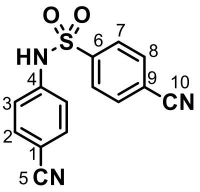

4-Cyano-N-(4-cyanophenyl)benzenesulfonamide **(17)**: By the method of Jang[37] 4-cyanobenzenesulfonyl chloride (444 mg, 2.2 mmol, 1.1 eq) was added to a solution of 4-aminobenzonitrile (236 mg, 2.0 mmol, 1 eq) in pyridine (10 mL) at 0 °C. The reaction mixture was stirred for 96 hours at room temperature. The reaction mixture was diluted with CH_2Cl_2 and washed with water (1 x 20 mL) and 1M HCl (3 x 20 mL). The combined organic layers were dried with anhydrous $MgSO_4$, filtered and concentrated under reduced pressure to afford the desired product (274 mg, 48%) as a cream solid.

^{1}**H NMR** (400 MHz, DMSO-d_6) δ; 11.27 (1H, br s, H6), 8.08 (2H, d, *J* = 8.7 Hz, H9), 7.99 (2H, d, *J* = 8.7 Hz, H8), 7.73 (2H, d, *J* = 8.8 Hz, H2), 7.25 (2H, d, *J* = 8.8 Hz, H3)

^{13}**C NMR** (101 MHz, DMSO-d_6) δ; 143.5 (C7), 142.0 (C4), 134.3 (C2), 134.2 (C9), 127.9 (C8), 119.5 (C3), 119.0 (C5), 117.9 (C11), 116.4 (C10), 106.6 (C1)

HRMS-APCI (m/z): $[M + H]^+$ calcd for $C_{14}H_9N_3O_2S$, 284.0488; found, 284.0493

1.6 Compounds synthesised for mechanistic investigation

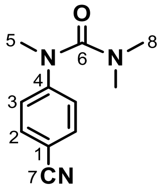

N-(4-Cyanophenyl)-N,N',N'-trimethylurea **(3b)** 4-cyanophenyl)(methyl)carbamic chloride (1557 mg, 8 mmol, 1.0 eq) was added to a solution of dimethylamine hydrochloride (979 mg, 12.0 mmol, 1.5 eq) and Et_3N (3.3 mL, 2396 mg, 23.7 mmol, 3 eq) in CH_2Cl_2 (80 mL) and the reaction mixture stirred for 19 hours. The reaction mixture was diluted with water and extracted with CH_2Cl_2. The combined organic layers were dried with anhydrous $MgSO_4$, filtered and concentrated under reduced pressure to afford the crude product. Purification by silica column chromatography (0-50% EtOAc in petroleum ether) afforded the desired product (1415 mg, 87%) as a white solid.

^{1}H NMR (400 MHz, $CDCl_3$) δ; 7.53 (2H, d, *J* = 8.2 Hz, H2), 6.99 (2H, d, *J* = 8.2 Hz, H3), 3.21 (3H, s, H5), 2.77 (6H, s, H8)

^{13}C NMR (101 MHz, $CDCl_3$) δ; 160.4 (C6), 150.0 (C4), 133.5 (C2), 120.2 (C3), 119.1 (C7), 105.1 (C1), 37.8 (C8), 37.7 (C5)

IR n_{max} (ATR)/ cm^{-1} 2946 (w), 2220 (m, CN), 1650 (s, CO), 1601 (s), 1492 (s), 1109 (s), 833 (s)

HRMS-ESI (m/z): $[M + H]^+$ calcd for $C_{11}H_{14}N_3O$, 204.1131; found, 204.1129

TLC R_f 0.20 (70:30 EtOAc/petroleum ether) [UV]

Mp = 84-86 °C

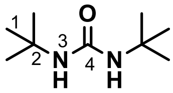

N,N'-Di-tert-butylurea By the method of Clayden[1] tert-butyl amine (1.05 mL, 731 mg, 10 mmol, 1 eq) and Et_3N (3.1 mL, 2251 mg, 2.2 eq) were dissolved in CH_2Cl_2 (50 mL) and the reaction mixture cooled to 0 °C. Triphosgene (475 mg, 1.6 mmol, 0.16 eq) was added portionwise and the reaction mixture was stirred for 24 hours at rt. The reaction mixture was diluted with water and extracted with CH_2Cl_2. The combined organic layers were dried with anhydrous $MgSO_4$, filtered and concentrated under reduced pressure to afford the desired product (581 mg, 67%) as a white solid.

^{1}H NMR (400 MHz, $CDCl_3$) δ; 4.03 (2H, br s, H3), 1.31 (18H, s, H1)

^{13}C NMR (101 MHz, $CDCl_3$) δ; 157.0 (C4), 50.4 (C2), 29.7 (C1)

MS-ESI (m/z): $[M + H]^+$ calcd for $C_9H_{21}N_2O$, 173.2 ; found, 173.2

Data consistent with previous reports[38]

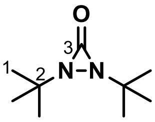

1,2-Di-tert-butyldiaziridin-3-one By the method of Shi[39] *N,N'*-di-tert-butylurea (345 mg, 2 mmol, 1 eq) was dissolved in Et_2O (2.4 mL) and tert-butyl hypochlorite (~70% purity, 0.34 mL, 316 mg, 1.02 eq) was added dropwise. After the solution went clear potassium *tert*-butoxide (20 wt% in THF, 1.29 mL, 258 mg, 1.15 eq) was added followed by addition of Et_3N (3 µL, 1.9 mg, 0.016 eq) and the reaction was stirred for 4 hours. The reaction mixture was diluted with pentane and washed with water and brine. The organic layer were dried with anhydrous $MgSO_4$, filtered and concentrated under reduced pressure to afford the desired product (281 mg, 83%) as a clear oil.

^{1}H NMR (400 MHz, $CDCl_3$) δ; 1.19 (18H, s, H1)

^{13}C NMR (101 MHz, $CDCl_3$) δ; 159.3 (C3), 59.3 (C2), 27.0 (C1)

IR n_{max} (ATR)/ cm^{-1} 2974 (m), 1932 (w), 1877 (s), 1858 (s), 1366 (s), 1192 (s), 1074 (s)

MS-ESI (m/z): $[M + H]^+$ calcd for $C_9H_{19}N_2O$, 171.1 ; found, 171.1

Data consistent with previous reports[39]

2. LiDBB conditions

2.1 Optimisation of LiDBB conditions

A more thorough investigation of the LiDBB conditions was carried out of urea 1b. Li source, reagent equivalents and reaction temperature were investigated (Supplementary Table 1). Additionally the addition of different additives were screened (Supplementary Table 2).

Supplementary Table 1. Initial optimisation of Li metal reduction

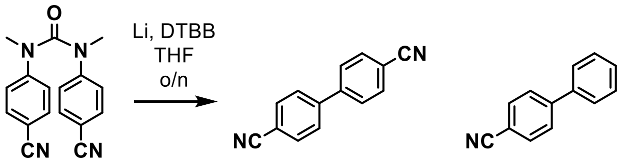

1b **2b** **2o**

Entry	Li source	Li /eq	DBB /eq	Temp /°C	1b/%	2b/%	20/%
**1**	Wire – sonicated	5	5	-78 to rt	73	**13**	-
**2**	Wire – pressed	5	1	-78 to rt	39	**45**	-
**3**	Wire – pressed	10	1	rt	-	**70**	11
**4***	Wire – pressed	10	1	rt	(88)	**(7)**	-
**5**	Powder	10	1	-78 to rt	-	**31**	43
**6**	Powder	10	1	rt	-	**43**	21

*Urea added at start; isolated yields (NMR yield)

Supplementary Table 2. Further optimisation of LiDBB reduction, addition of additives

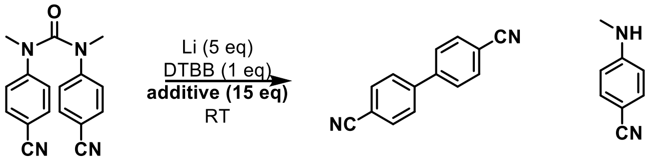

1b **2b** **20**

Additive	Rxn time /hr	1a /%	2b /%	20 /%
**none**	17	0	70	0
**TPPA**	4	0	40	38
**DMPU**	20	6	47	13
**LiCl**	5	0	88	0

Yields = isolated yields

2.2 Scope of biaryl formation using LiDBB as reductant

The scope of biaryl formation using LiDBB as the reductant was also investigated, however only biaryls 2b and 2an were produced in good yields.

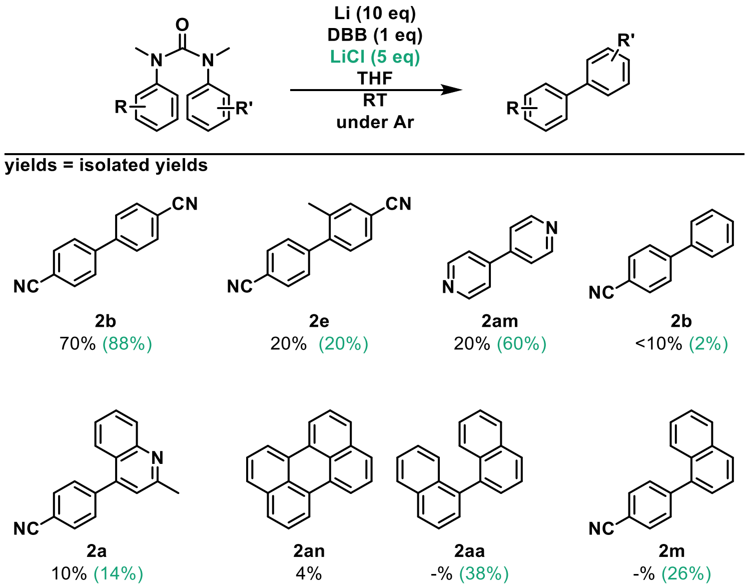

Supplementary Figure 1. Scope of LiDBB reduction, without LiCl (black), with LiCl (green)

2.3. Unsuccessful substrates using LiDBB conditions

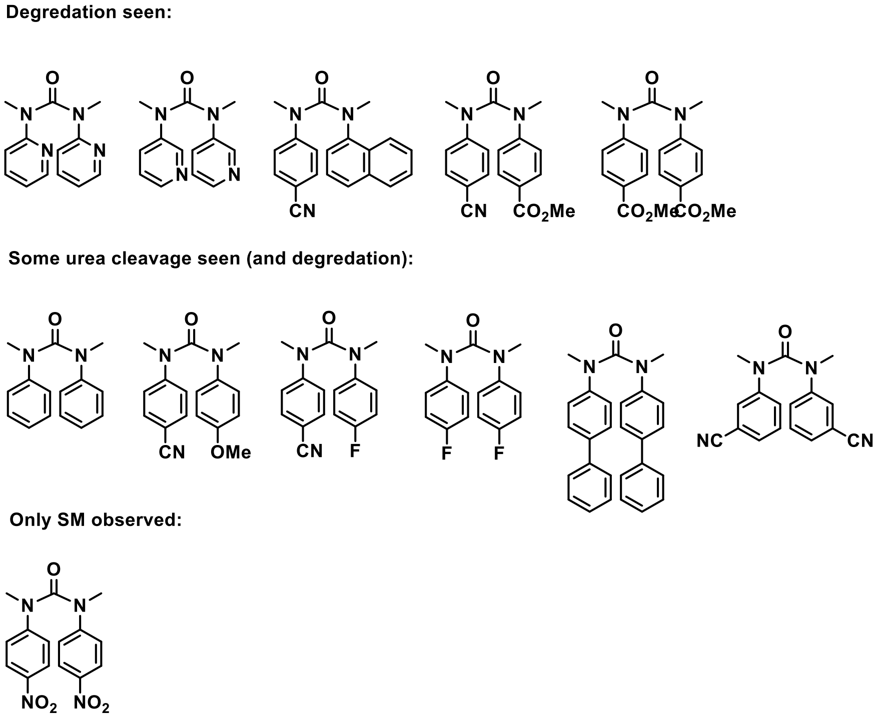

Supplementary Figure 2. Unsuccessful LiDBB (with LiCl) reductions

3. Investigation of different linkers

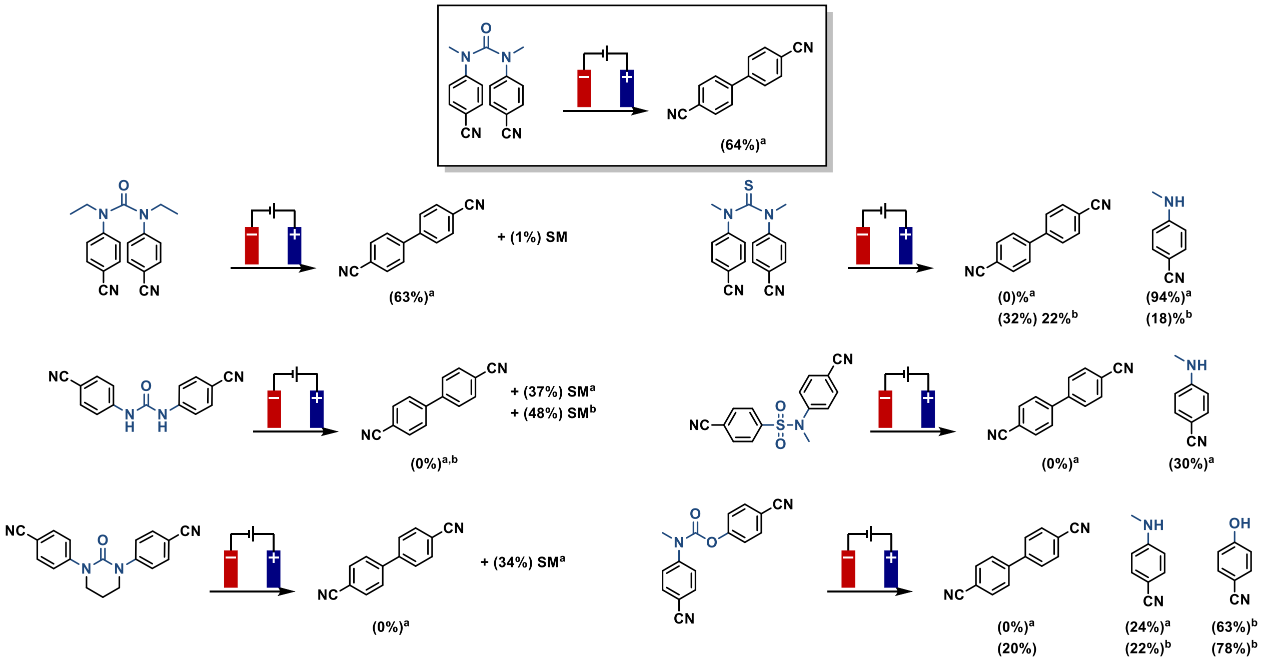

Supplementary Figure 3. Varying the linker, results of electrochemical reduction. (a) Gr(-)|Gr(+), LiCl (5 eq), $TBAPF_6$ (0.1 M), TBAB (6 eq), DMF (0.1 M), -6mA, 3 $Fmol^{-1}$.(b) Pt(-)|Pt(+), $TBAPF_6$ (0.1 M), TBAB (6 eq), DMF (0.1 M), -6mA, 3 $Fmol^{-1}$

4. Electrochemical optimisation

*Supplementary Table 3. Electrochemical optimisation of the reduction of urea **1a**, varying solvent, electrode material, concentration, current, LiCl eq and F mol^{-1}*

	Solvent	(-)	(+)	Conc /M	Current /mA	LiCl eq	F mol^{-1}	Pr /%	SM/%
1	DMF	Gr	Gr	0.1	-6	5	3	86	0
2	DMSO	Gr	Gr	0.1	-6	5	3	67	0
3	MeCN	Gr	Gr	0.1	-6	5	3	44	18
4	DMF	Pt	Gr	0.1	-6	5	3	54	0
5	DMF	GC	Gr	0.1	-6	5	3	54	10
6	DMF	Gr	Pt	0.1	-6	5	3	79	0
7	DMF	Gr	CC	0.1	-6	5	3	68	13
8	DMF	Gr	Gr	0.05	-6	5	3	62	5
9	DMF	Gr	Gr	0.2*	-6	5	3	71	0
10	DMF	Gr	Gr	0.1	-2	5	3	66	2
11	DMF	Gr	Gr	0.1	-4	5	3	72	1
12	DMF	Gr	Gr	0.1	-8	5	3	63	20
13	DMF	Gr	Gr	0.1	-6	0	3	51	27
14	DMF	Gr	Gr	0.1	-6	3.5	3	70	0
15	DMF	Gr	Gr	0.1	-6	7	3	82	0
16	DMF	Gr	Gr	0.1	-6	10	3	77	0
17	DMF	Gr	Gr	0.1	-6	5	1	34	64
18	DMF	Gr	Gr	0.1	-6	5	2	77	21

5. Unsuccessful substrates in electrochemical reduction

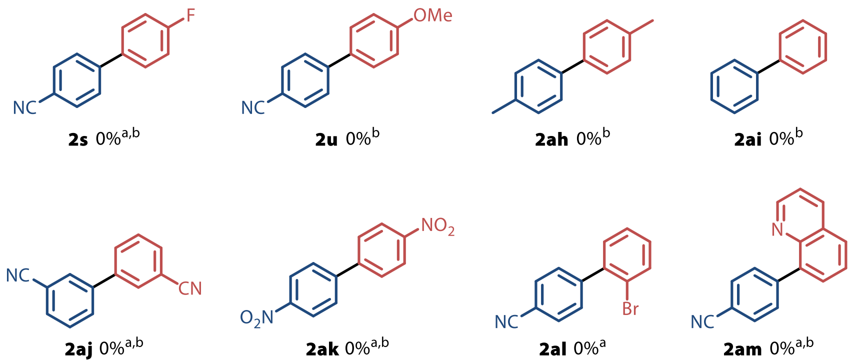

Supplementary Figure 4. Biaryls unable to form in electrochemical reduction of corresponding ureas

6. Electrochemical set up

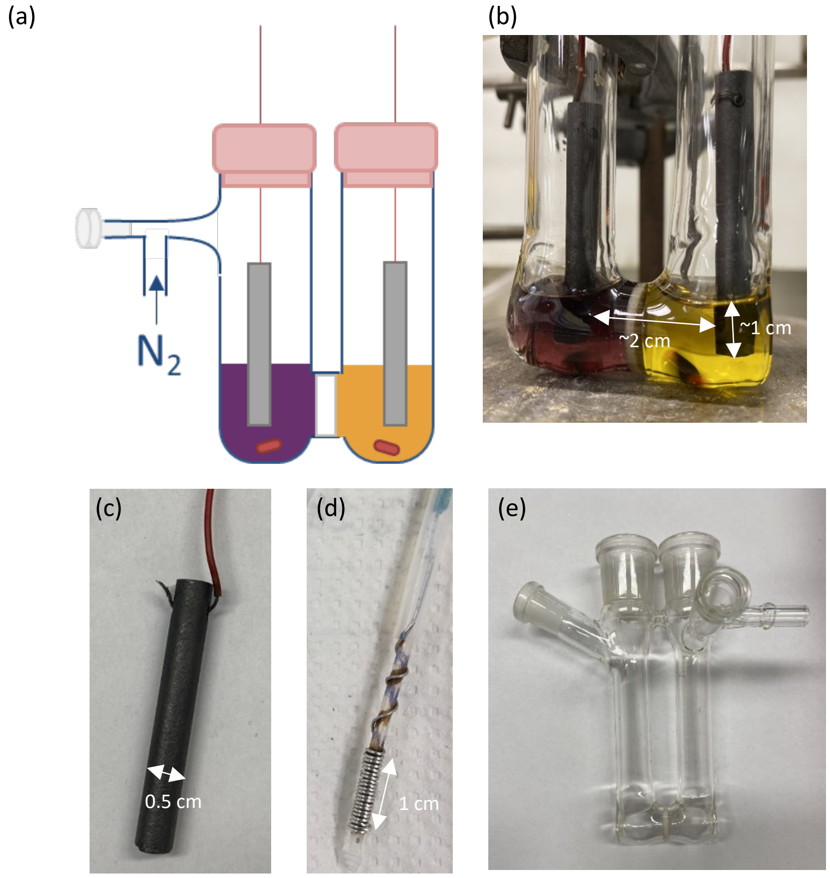

*Supplementary Figure 5. Electrochemical set up (a) Schematic of set up (b) Photo of the set up of the electrochemical reduction of urea **2a**, with electrode separation and electrode depth highlighted (c) Graphite rod electrode with diameter highlighted. A wire fed through a suba seal was connected to the graphite rod by feeding through a small hole at the top of the rod. (d) Platinum coil electrode. Platinum wire was wrapped around PTFE tubing creating a surface area approximately 1 cm^2, the platinum wire was then fed through PTFE tubing by creating a small hole on the side of the tubing. The wire was then spot welded to a copper wire, which was then fed through a suba seal. (e) H cell used in electrochemical reductions*

7. Diaziridinone CV and electrochemical reaction

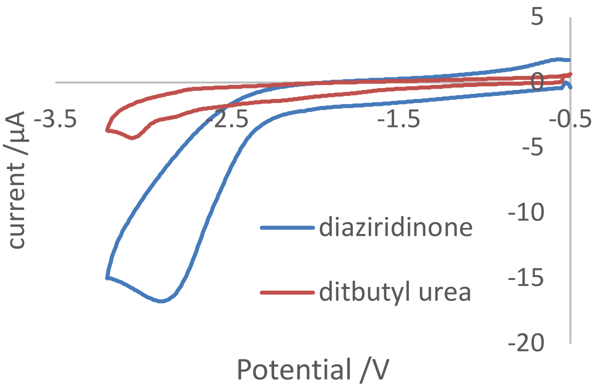

*Supplementary Figure 6. CV of tButyl diaziridinone (**19**) (blue) and tButyl urea (**20**) (red). Glassy carbon (-)|Pt wire (+), Ag/AgNO$_3$ (ref), 0.1 Vs^{-1}, 0.025 mmol substrate, 0.1 M TBAPF$_6$, 0.05 M DMF*

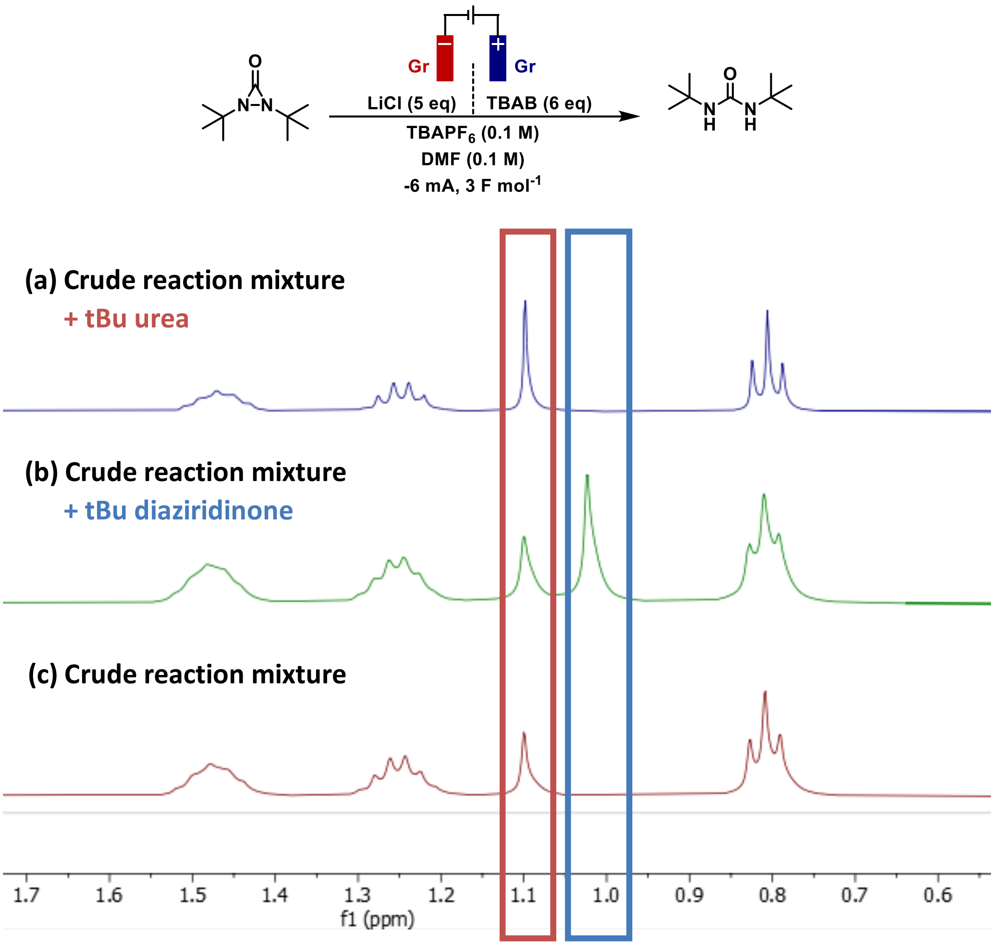

Supplementary Figure 7. Electrochemical reduction of tButyl diaziridinone. (a) Crude reaction mixture spiked with tBu urea (b) Crude reaction mixture spiked with tBu diaziridinone (c) Crude reaction mixture

8. Comparison of reduction potentials to reaction conditions

A plot of the reduction potential of each urea is shown (Supplementary Supplementary Figure 8). Ureas compatible with reaction conditions a (Gr(-),Gr(+) with LiCl) are coloured blue, and ureas requiring reaction conditions b (Pt(-), Pt(+), no LiCl) are coloured red. A clear divide at around -2.65 V is observed, allowing a prediction of required reaction conditions based on substrate reduction potential.

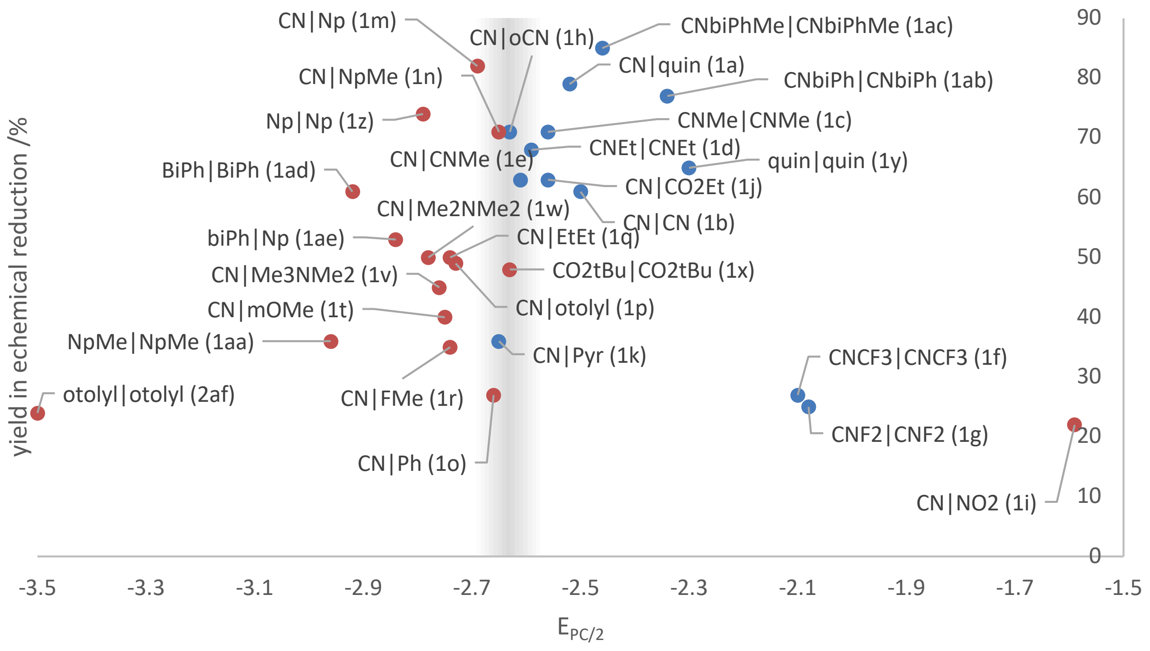

Supplementary Figure 8. Reduction potentials of ureas plotted against yield of electrochemical reduction. (blue) ureas reacted successfully under reaction conditions a. (red) ureas requiring reaction conditions b.

9. CV of urea 1a (3 scans) overlaid with CV of biaryl product 2a

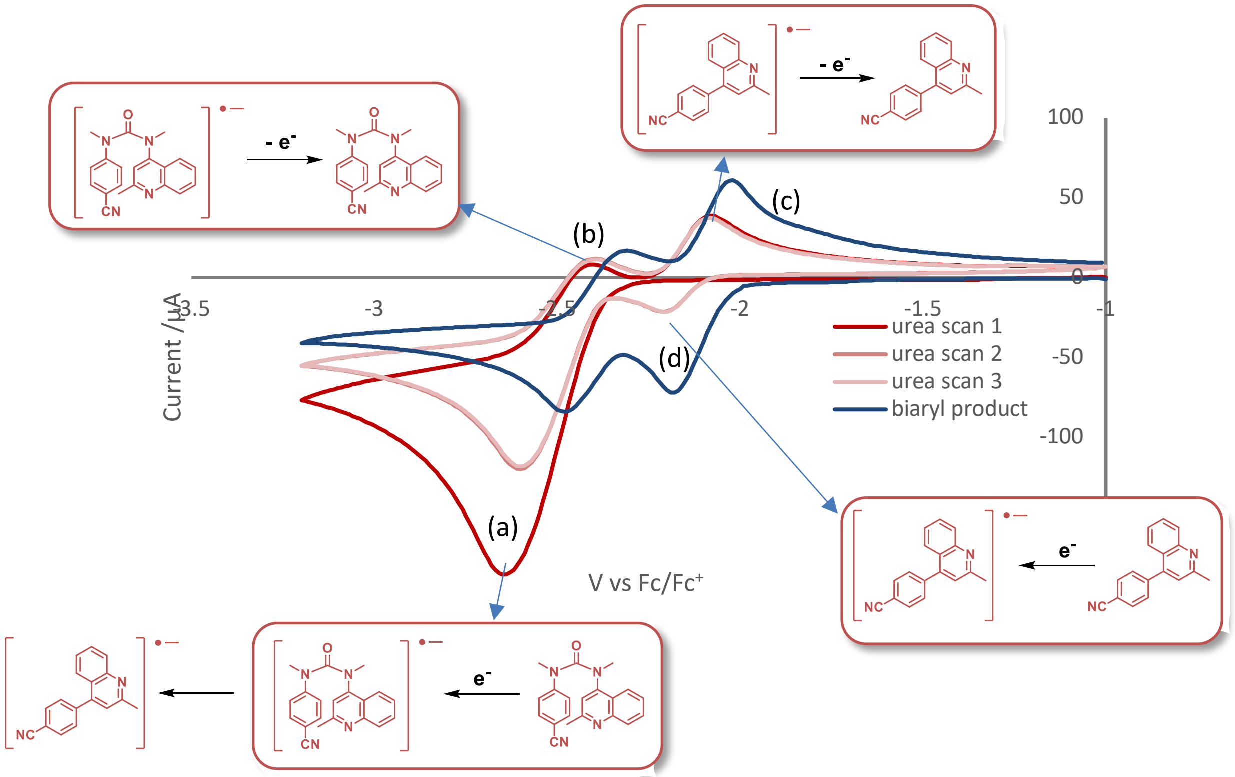

Supplementary Figure 9. CV of urea 1g (red -> pale red) overlaid with biaryl 2g (blue). (a) reduction of urea at -2.5 V. (b) small reverse oxidation peak. (c) proposed oxidation of the newly formed radical anion of biaryl product 2g. (d) reduction of newly formed neutral biaryl product 2g (only in second and subsequent scans). Glassy carbon (-)|Pt wire (+), Ag/AgNO$_3$ (ref), 0.1 Vs^{-1}, 0.025 mmol substrate, 0.1 M TBAPF$_6$, 0.05 M DMF

10. Effect of LiCl on CV of urea 1a

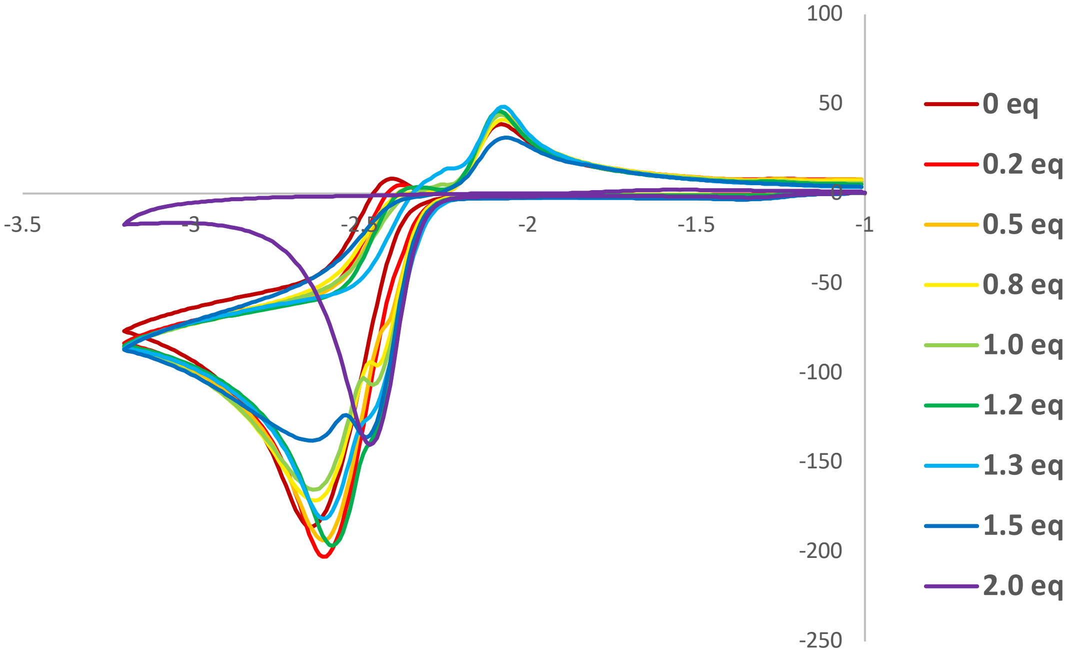

Supplementary Figure 10. CV of urea 1a with 0 - 2.0 eq LiCl added. Glassy carbon (-)|Pt wire (+), Ag/AgNO$_3$ (ref), 0.1 Vs^{-1}, 0.025 mmol substrate, 0.1 M TBAPF$_6$, 0.05 M DMF

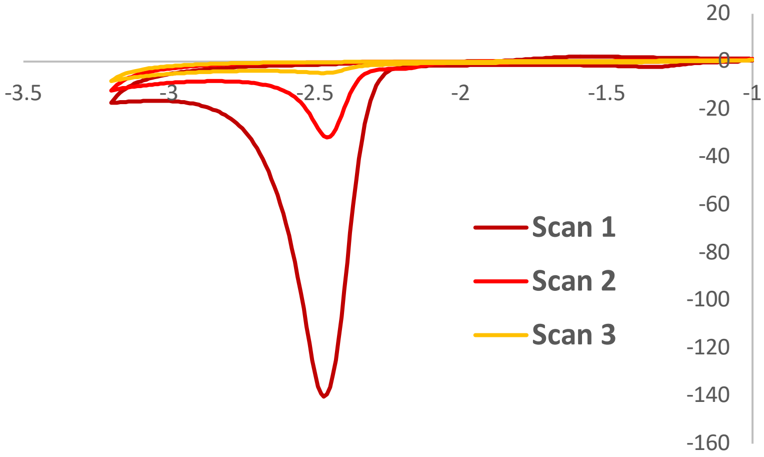

Supplementary Figure 11. CV of urea 1a + 2 eq LiCl, 3 scans. Glassy carbon (-)|Pt wire (+), Ag/AgNO$_3$ (ref), 0.1 Vs^{-1}, 0.025 mmol substrate, 0.1 M TBAPF$_6$, 0.05 M DMF

With increased LiCl added (Supplementary Figure 9) a slightly early reduction peak is observed in addition to the normal urea reduction peak. This suggests a small amount of some urea-LiCl adduct could be forming that is easier to reduce. Once at 2.0 eq of LiCl it appears this adduct is the only species present as the original urea reduction peak is no longer seen. This symmetrical peak could indicate a surface bound process, and interestingly upon cycling for multiple scans (Supplementary figure 10) the signal is quickly depleted suggesting further indicating formation of a surface bound species.

11. CVs of ureas

Supplementary Table 4. CVs of ureas 1a-1al. Glassy carbon (-)|Pt wire (+), Ag/AgNO3 (ref), 0.1 Vs-1, 0.025 mmol substrate, 0.1 M TBAPF6, 0.05 M DMF.

<table>
<tr><td>1a
CN|quin

→ $E_{PC/2}$ = -2.55 V</td><td>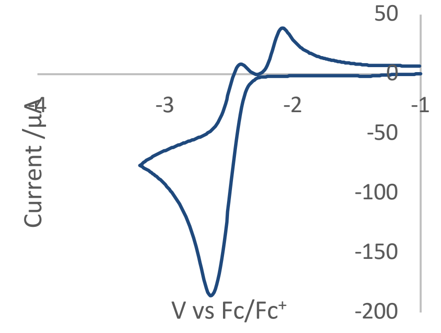</td><td>1b
CN|CN

→ $E_{PC/2}$ = -2.50 V</td><td>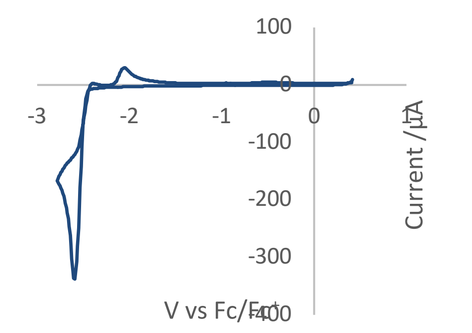</td></tr>
<tr><td>1c
CNMe|
CNMe

→$E_{PC/2}$ = -2.56</td><td>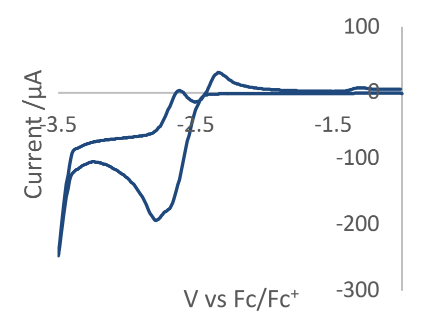</td><td>1d
CNEt|
CNEt

→ $E_{PC/2}$ = -2.59 V</td><td>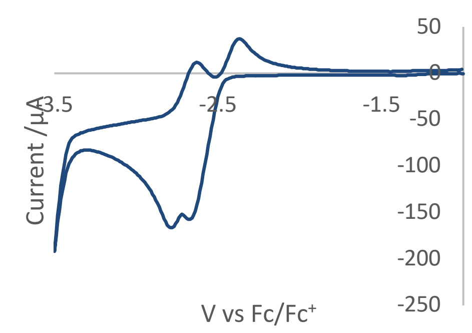</td></tr>
<tr><td>1e
CN|
CNMe

→ $E_{PC/2}$ = -2.61 V</td><td>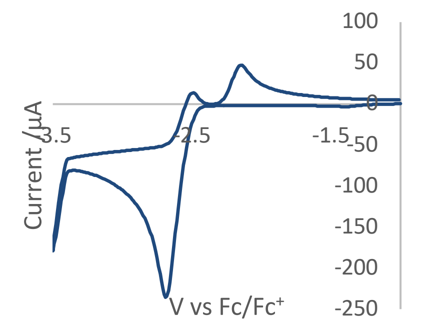</td><td>1f
CNCF3|
CNCF3

→ $E_{PC/2}$ = -2.10 V</td><td>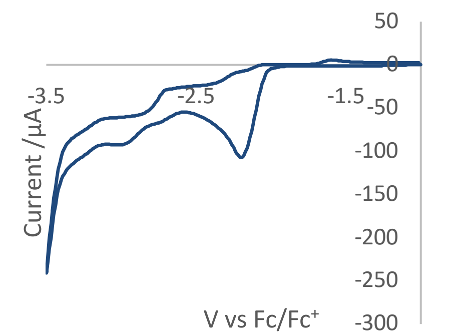</td></tr>
<tr><td>1g
CNF2|
CNF2

→ $E_{PC/2}$ = -2.08 V</td><td>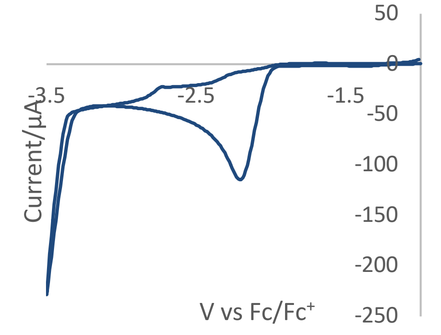</td><td>1h
pCN|oCN

→ $E_{PC/2}$ = -2.63 V</td><td>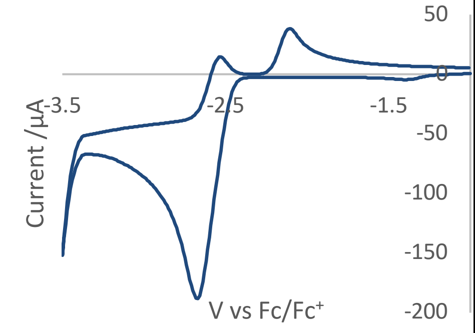</td></tr>
</table>

**1i**<br>CN|NO2<br><br>→ $E_{PC/2}$ = -1.59 V	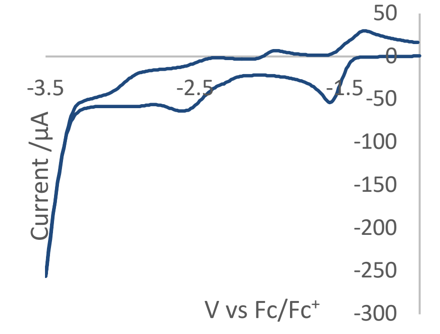	**1j**<br>CN| CO_2Et<br><br>→ $E_{PC/2}$ = -2.56 V	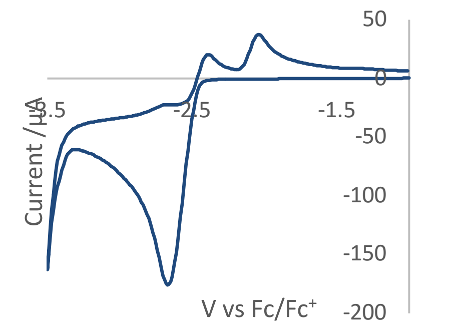
**1k**<br>CN|pyr<br><br>→ $E_{PC/2}$ = -2.65 V	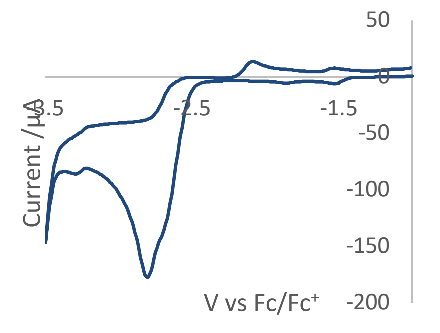	**1l**<br>CN|biPh<br><br>→ $E_{PC/2}$ = -2.74 V	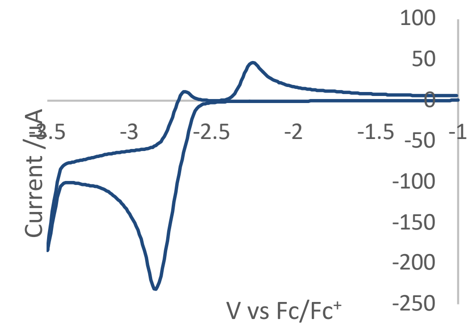
**1m**<br>CN|Np<br><br>→ $E_{PC/2}$ = -2.73	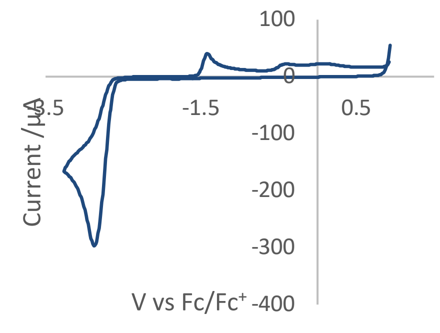	**1n**<br>CN|NpMe<br><br>→ $E_{PC/2}$ = -2.65 V	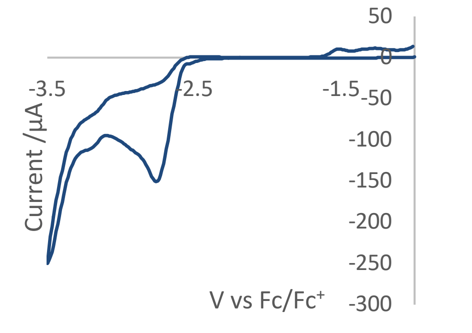
**1o**<br>CN|Ph<br><br>→ $E_{1/2}$ = -2.66 V	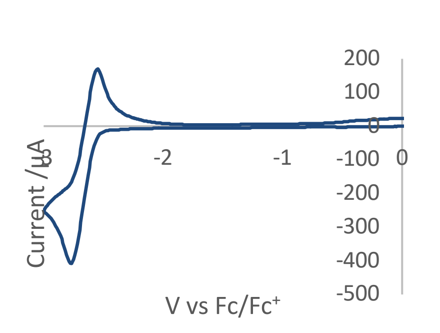	**1p**<br>CN| o-tolyl<br><br>→ $E_{1/2}$ = -2.69 V	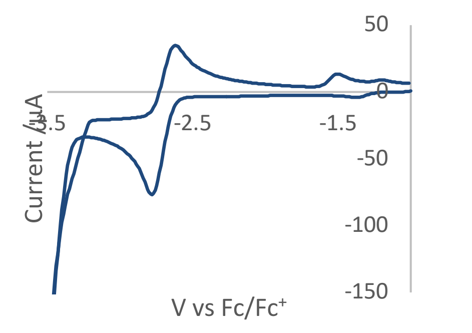
**1q**<br>CN| PhEtEt<br><br>→ $E_{1/2}$ = -2.74 V	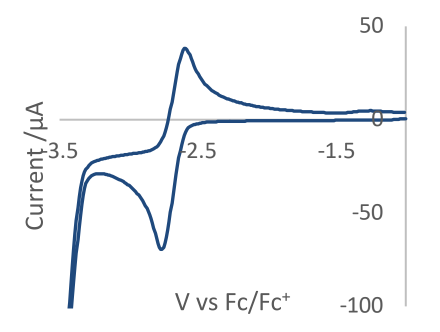	**1r**<br>CN|FMe<br><br>→ $E_{1/2}$ = -2.66 V	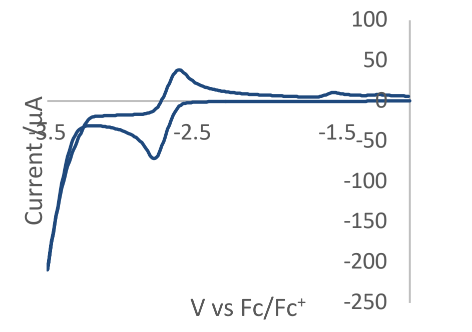

<table>
<tr><td>1s
CN|F

→ $E_{1/2}$ =
-2.72 V</td><td>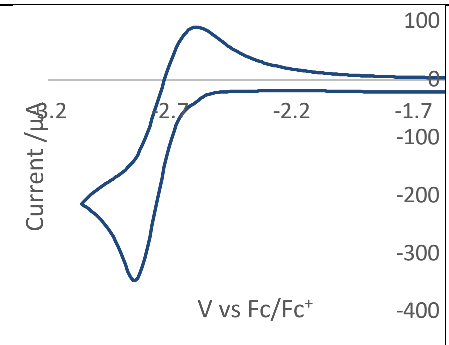
</td><td>1t
CN|
mOMe

→ $E_{PC/2}$ =
-2.75 V</td><td>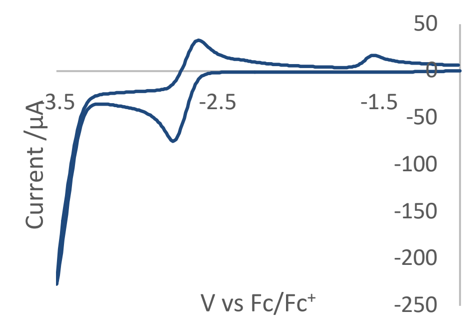
</td></tr>
<tr><td>1u
CN|
p-OMe

→ $E_{PC/2}$ =
-2.67 V</td><td>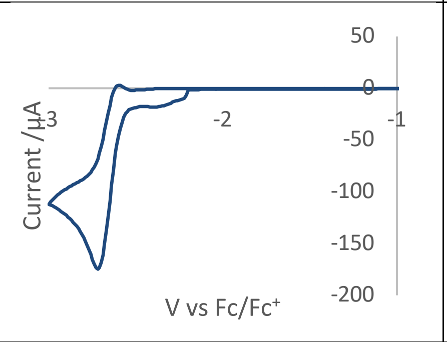
</td><td>1v
CN|
Me_3NMe_2

→ $E_{PC/2}$ =
-2.76 V</td><td>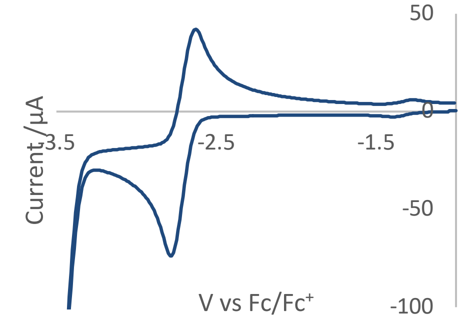
</td></tr>
<tr><td>1w
CN|
Me_2NMe_2

→ $E_{PC/2}$ =
-2.78 V</td><td>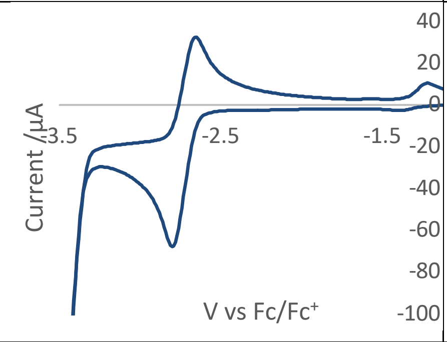
</td><td>1x
CO_2tBu|
CO_2tBu

→ $E_{PC/2}$ =
-2.63 V</td><td>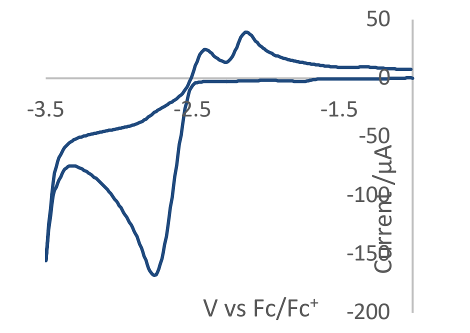
</td></tr>
<tr><td>1y
quin|qui
n

→ $E_{1/2}$ =
-2.27 V</td><td>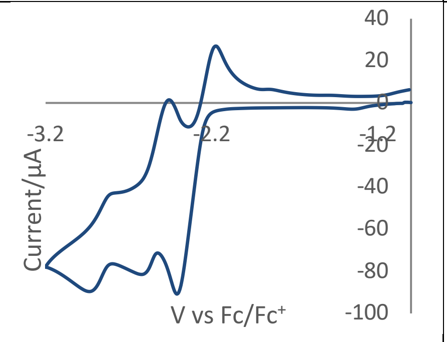
</td><td>1z
Np|Np

→ $E_{PC/2}$ =
-2.79 V</td><td>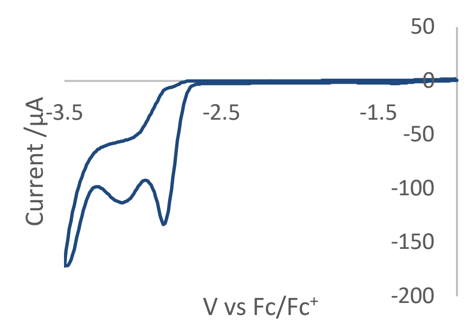
</td></tr>
<tr><td>1aa
NpMe|
NpMe

→ $E_{PC/2}$ =
-2.96 V</td><td>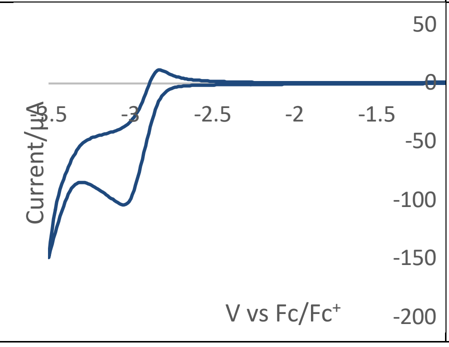
</td><td>1ab
CNbiPh|
CbiPh

→ $E_{PC/2}$ =
-2.34 V</td><td>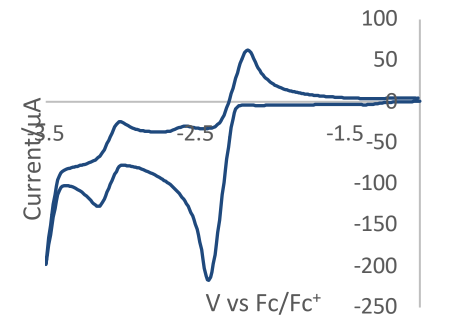
</td></tr>
</table>

**1ac**<br>CNbiPh Me| CNbiPh Me<br><br>→ $E_{PC/2}$ =<br>-2.46 V	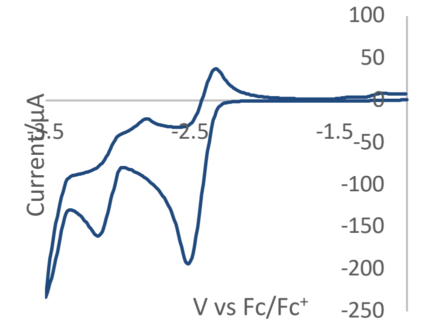	**1ad**<br>biPh| biPh<br><br>→ $E_{PC/2}$ =<br>-2.92 V	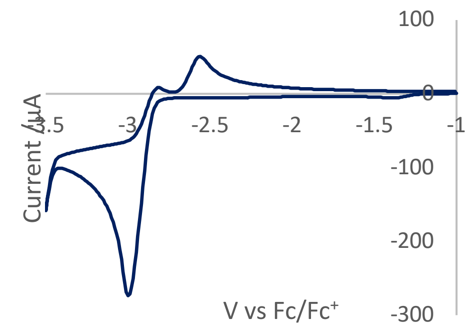
**1ae**<br>biPh|Np<br><br>→ $E_{PC/2}$ =<br>-2.84 V	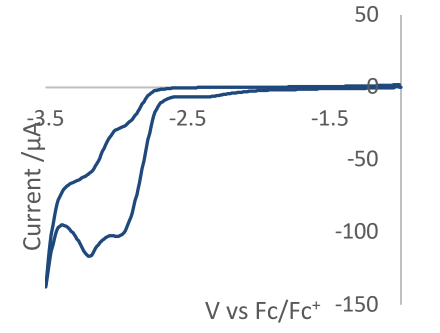	**1af**<br>o-tolyl| o-tolyl<br><br>→ $E_{PC/2}$ =<br><-3.5 V	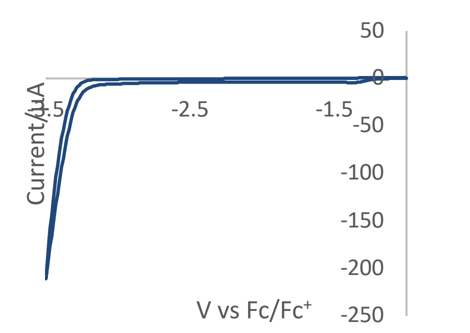
**1ag**<br>p-tolyl| p-tolyl<br><br>→ $E_{PC/2}$ =<br><-3.5 V	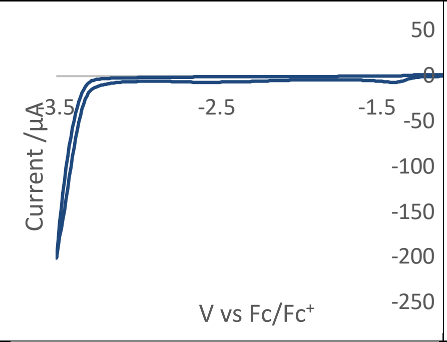	**1ah**<br>Ph|Ph<br><br>→ $E_{PC/2}$ =<br><-3.5 V	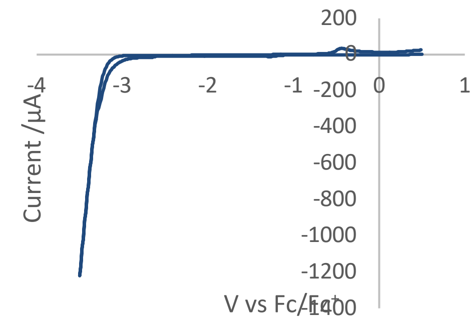
**1ai**<br>mCN| mCN<br><br>→ $E_{PC/2}$ =<br>-2.58 V	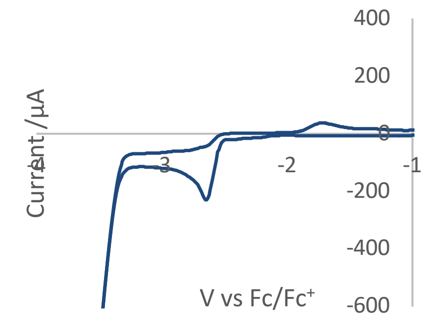	**1aj**<br>NO_2|NO_2<br><br>→ $E_{1/2}$ =<br>-1.52 V	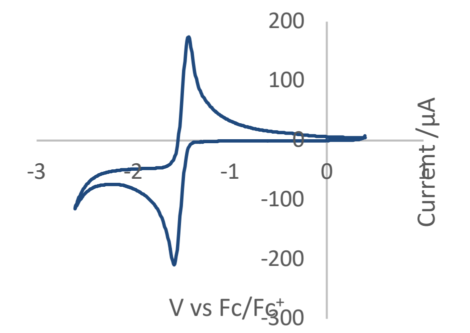
**1al**<br>CN|8quin<br><br>→ $E_{PC/2}$ =<br>-2.39 V	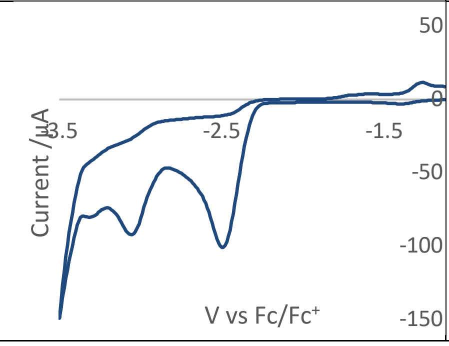		

12. NMR spectra

12.1 NMR spectra of ureas

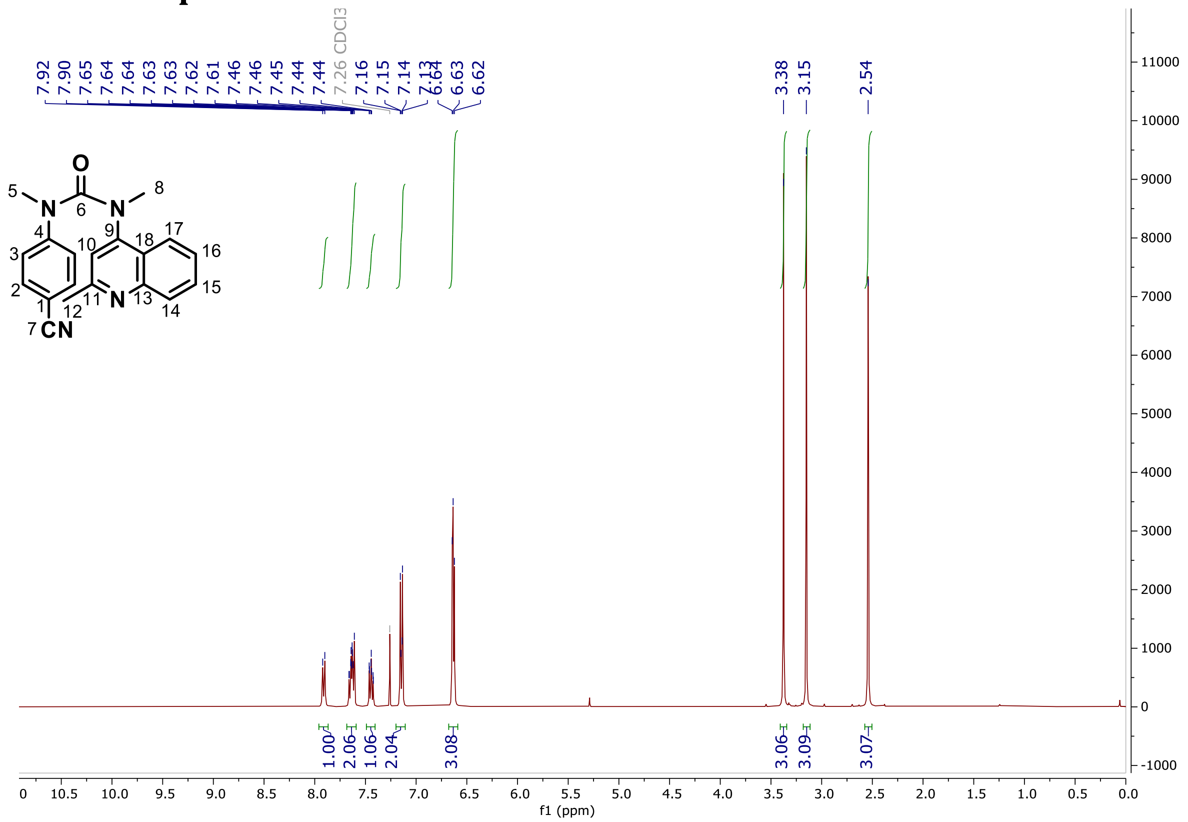

Supplementary Figure 12. ^{1}H NMR of N-(4-cyanophenyl)-N,N'-dimethyl-N'-(2-methylquinolin-4-yl)urea (**1a**) (400 MHz, 20 °C, $CDCl_3$)

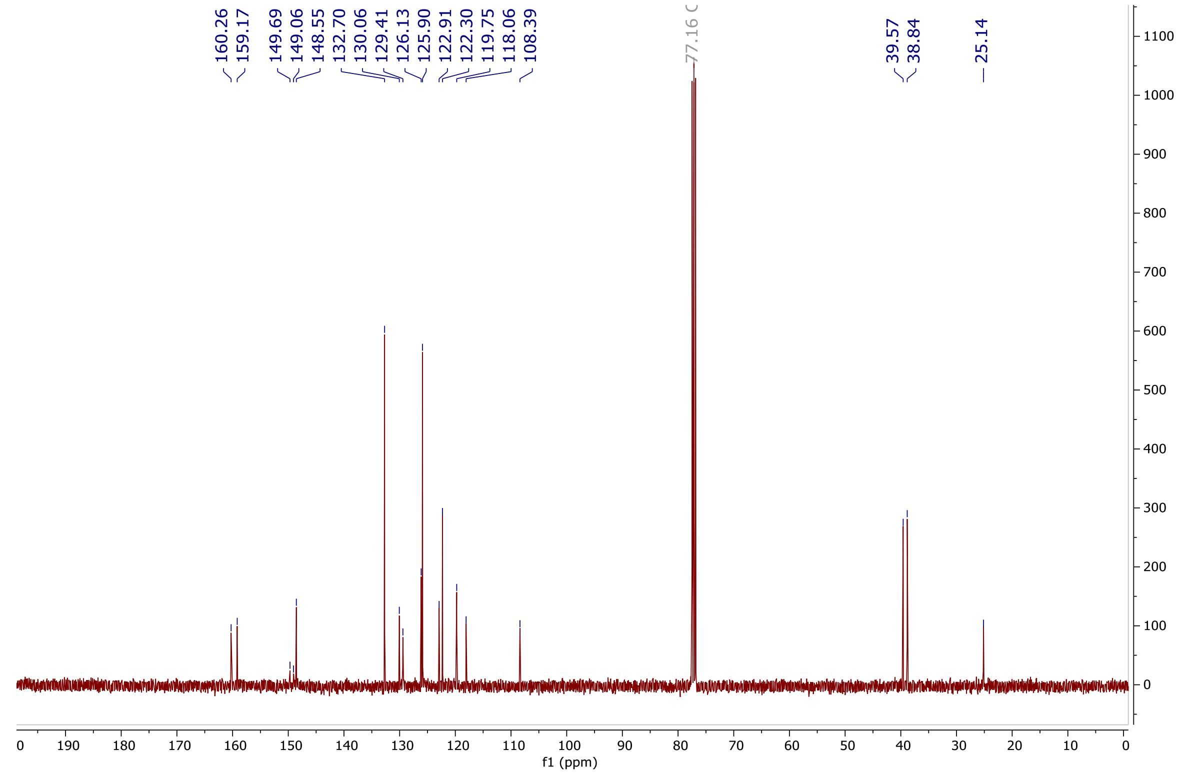

Supplementary Figure 13. ^{13}C NMR of N-(4-cyanophenyl)-N,N'-dimethyl-N'-(2-methylquinolin-4-yl)urea (**1a**) (101 MHz, 20 °C, $CDCl_3$)

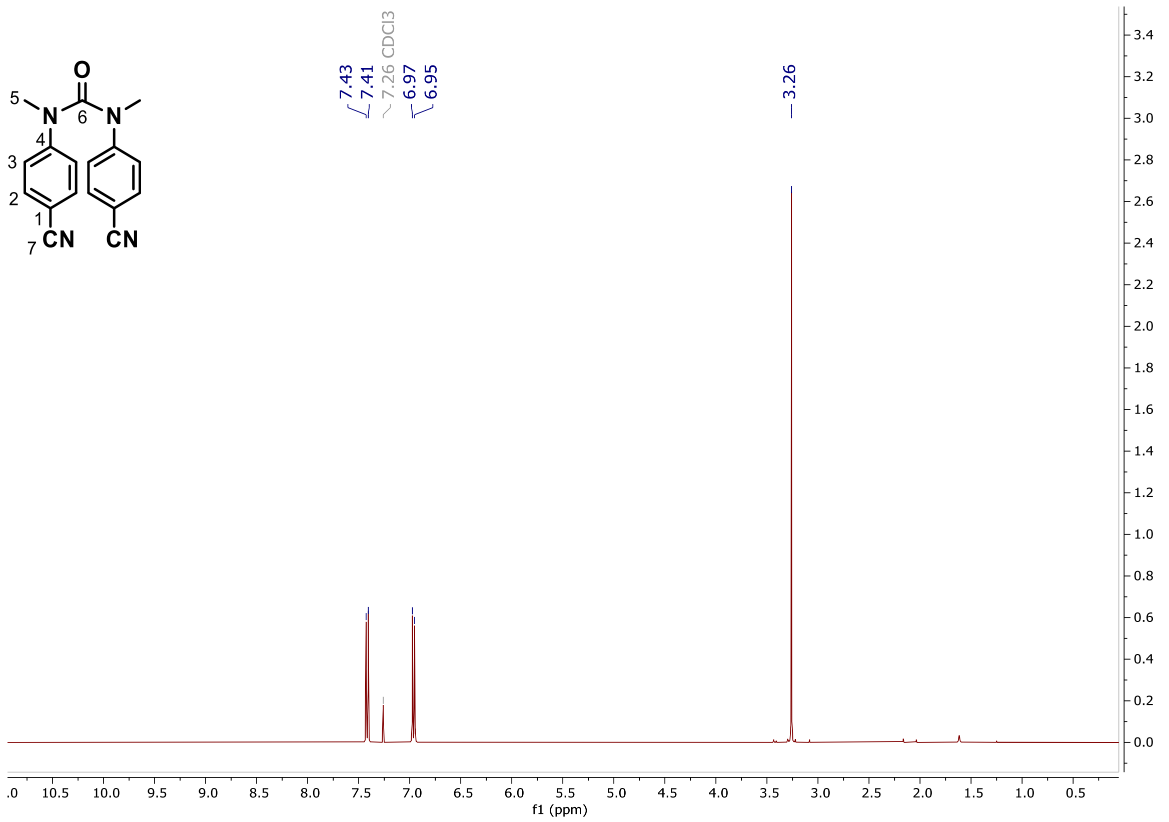

*Supplementary Figure 14. ¹H NMR of N,N'-bis(4-cyanophenyl)-N,N'-dimethylurea (**1b**) (400 MHz, 20 °C, $CDCl_3$)*

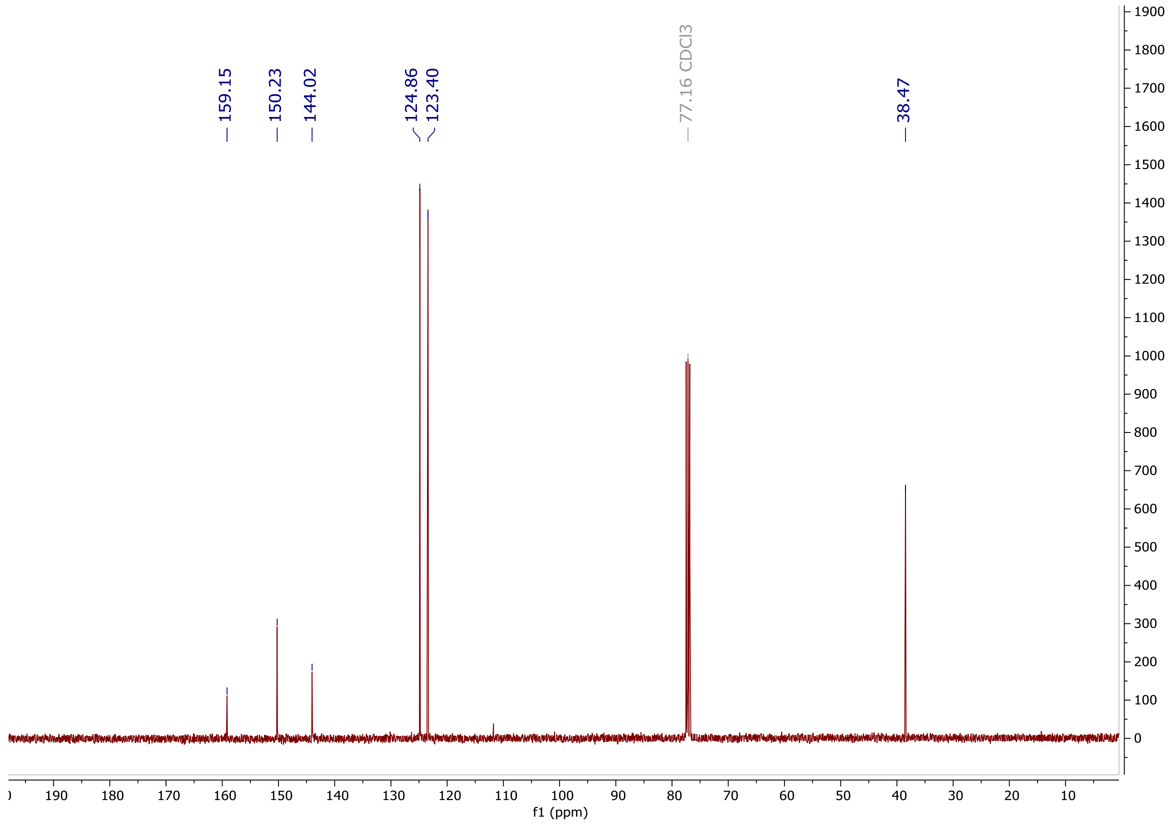

*Supplementary Figure 15. ¹³C NMR of N,N'-bis(4-cyanophenyl)-N,N'-dimethylurea (**1b**) (101 MHz, 20 °C, $CDCl_3$)*

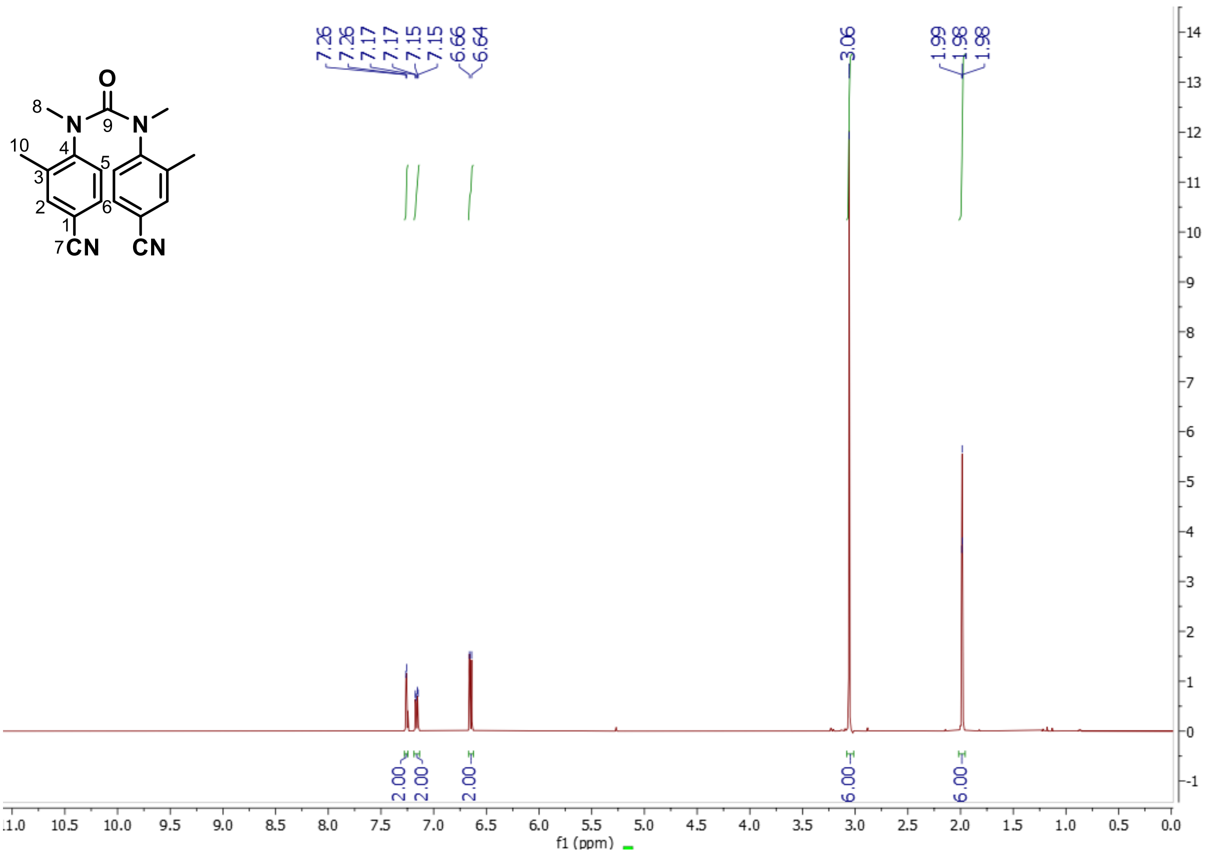

Supplementary Figure 16. ^{1}H *NMR of N,N'-bis(4-cyano-2-methylphenyl)-N,N'-dimethylurea (**1c**) (400 MHz, 20 °C,* $CDCl_3$*)*

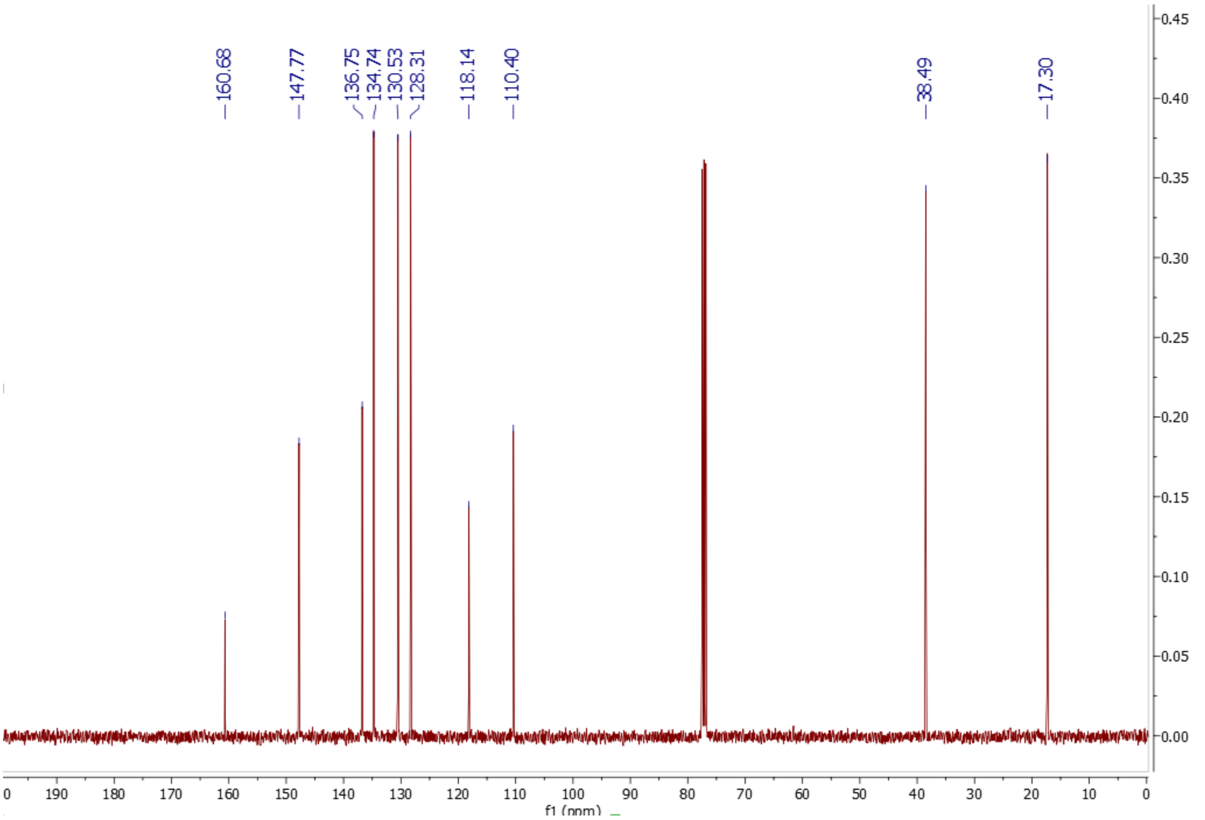

Supplementary Figure 17. ^{13}C *NMR of N,N'-bis(4-cyano-2-methylphenyl)-N,N'-dimethylurea (**1c**) (101 MHz, 20 °C,* $CDCl_3$*)*

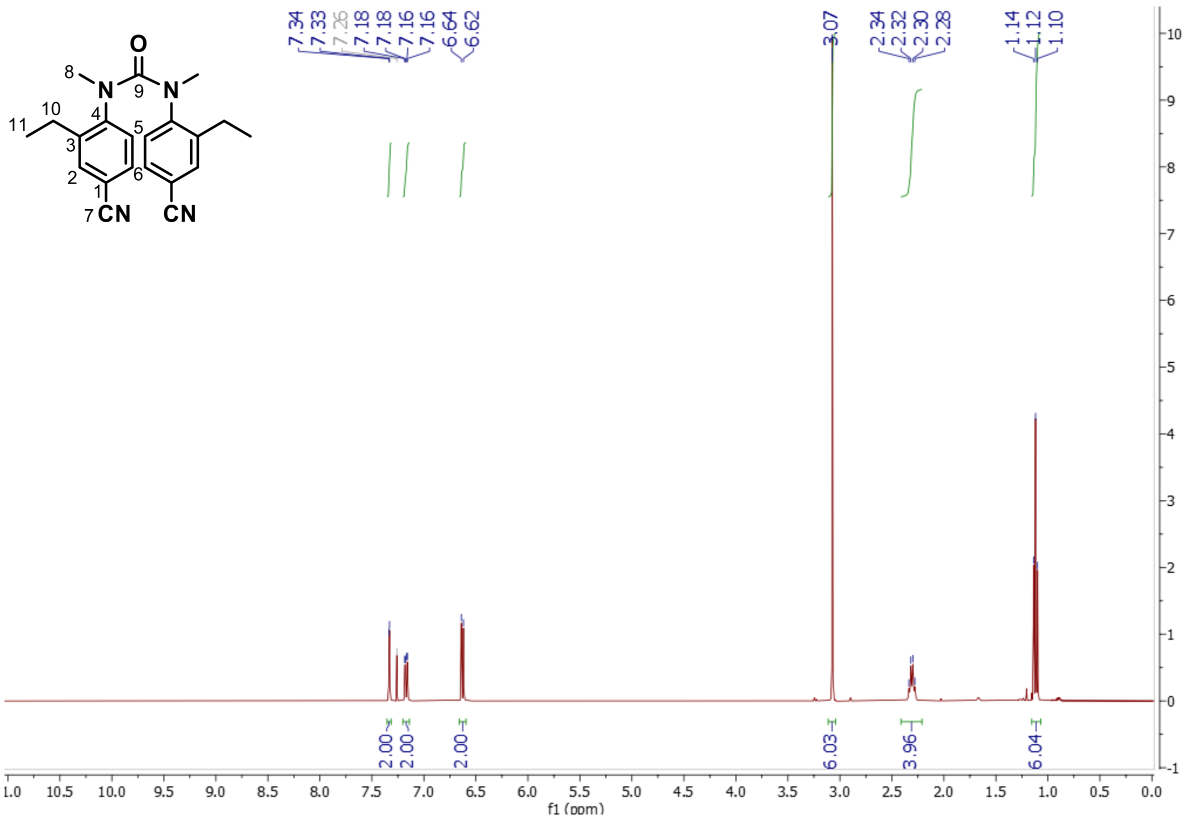

*Supplementary Figure 18. ^{1}H NMR of N,N'-bis(4-cyano-2-ethylphenyl)-N,N'-dimethylurea (**1d**) (400 MHz, 20 °C, $CDCl_3$)*

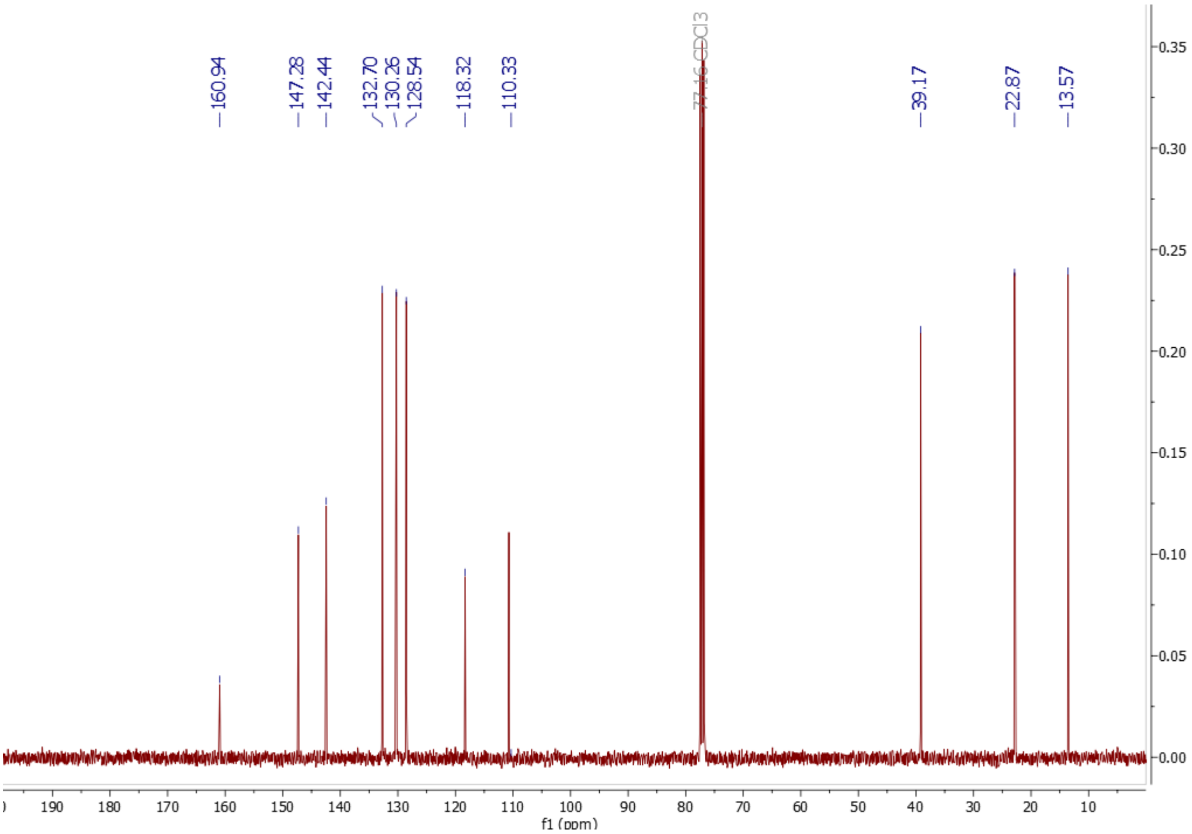

*Supplementary Figure 19. ^{13}C NMR of N,N'-bis(4-cyano-2-ethylphenyl)-N,N'-dimethylurea (**1d**) (101 MHz, 20 °C, $CDCl_3$)*

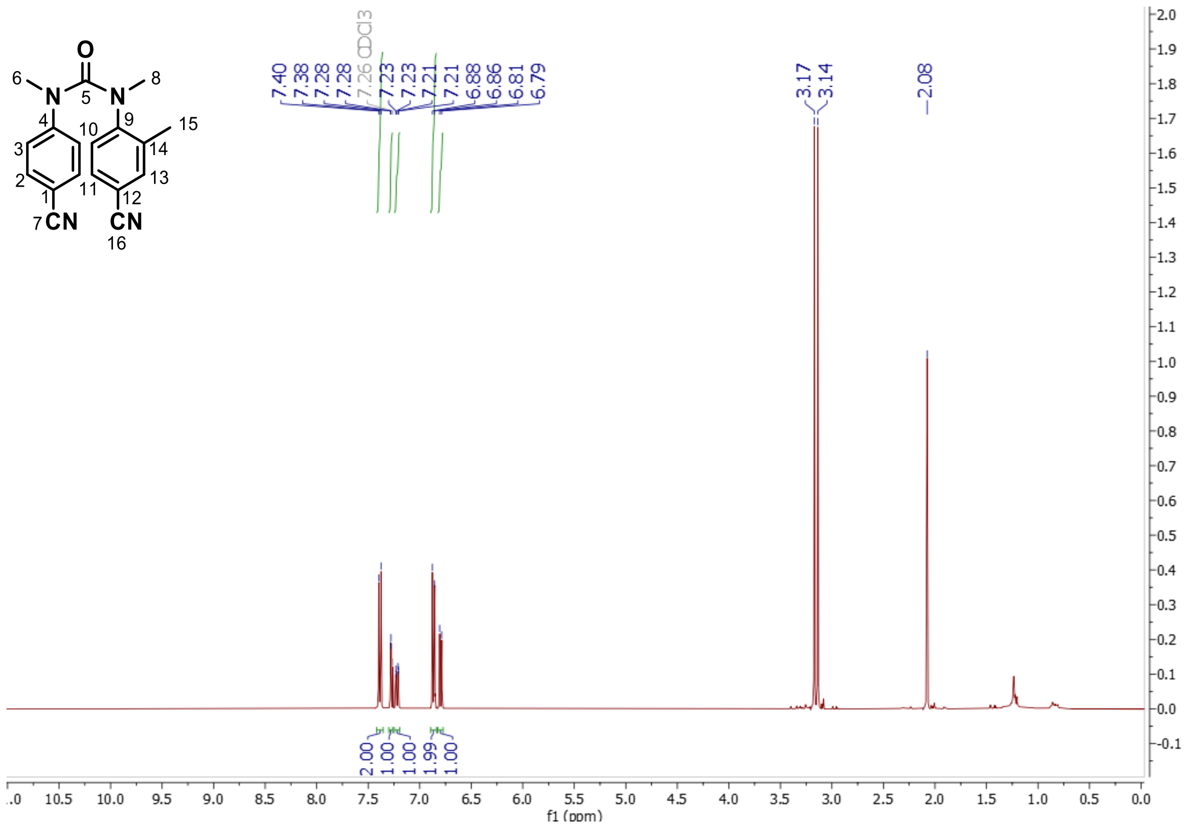

*Supplementary Figure 20. ^{1}H NMR of N-(4-cyano-2-methylphenyl)-N'-(4-cyanophenyl)-N,N'-dimethylurea (**1e**) (400 MHz, 20 °C, $CDCl_3$)*

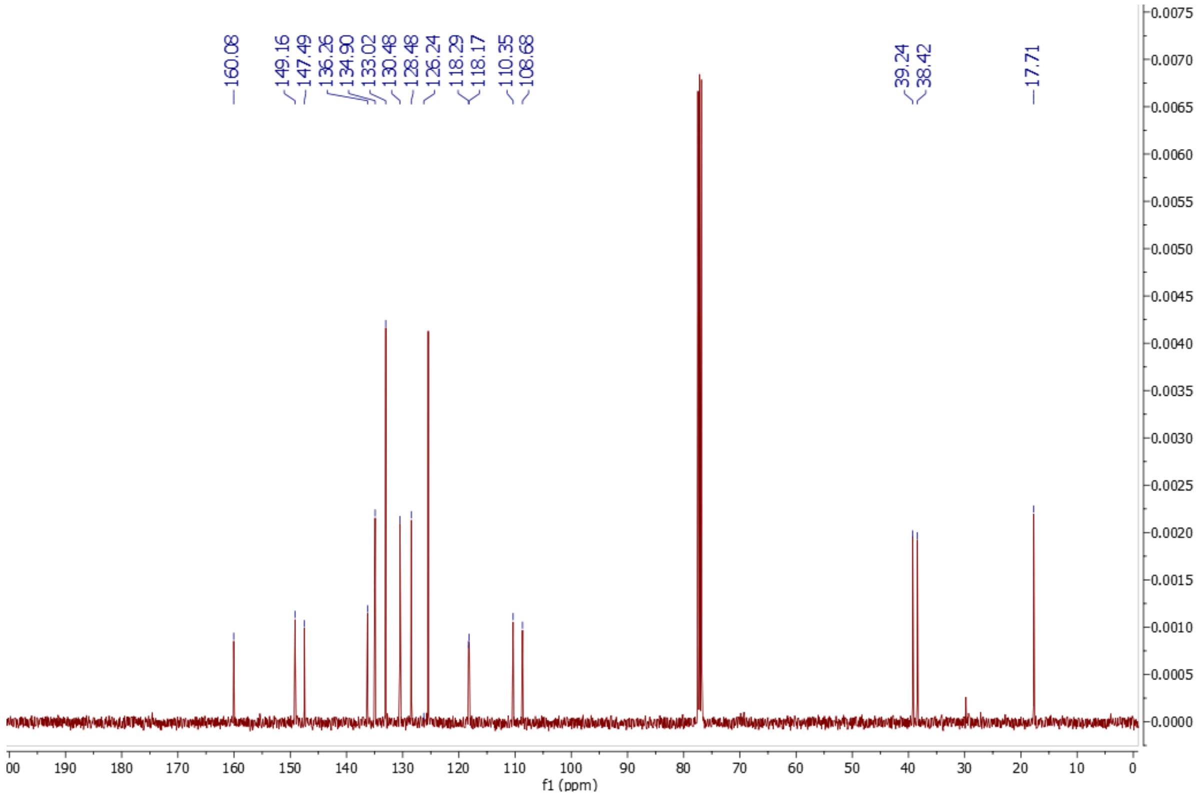

*Supplementary Figure 21. ^{13}C NMR of N-(4-cyano-2-methylphenyl)-N'-(4-cyanophenyl)-N,N'-dimethylurea (**1e**) (101 MHz, 20 °C, $CDCl_3$)*

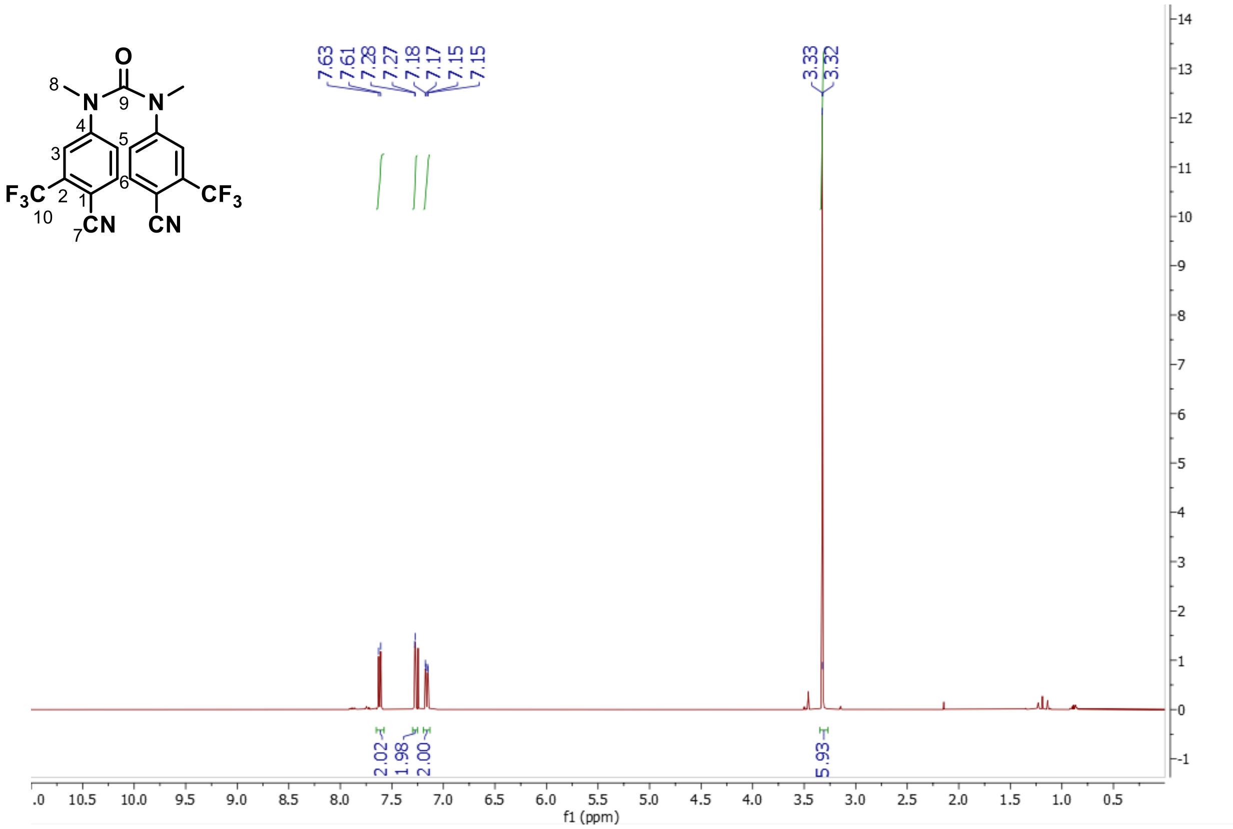

*Supplementary Figure 22.¹H NMR of N,N'-bis(4-cyano-3-(trifluoromethyl)phenyl)-N,N'-dimethylurea (**1f**) (400 MHz, 20 °C, $CDCl_3$)*

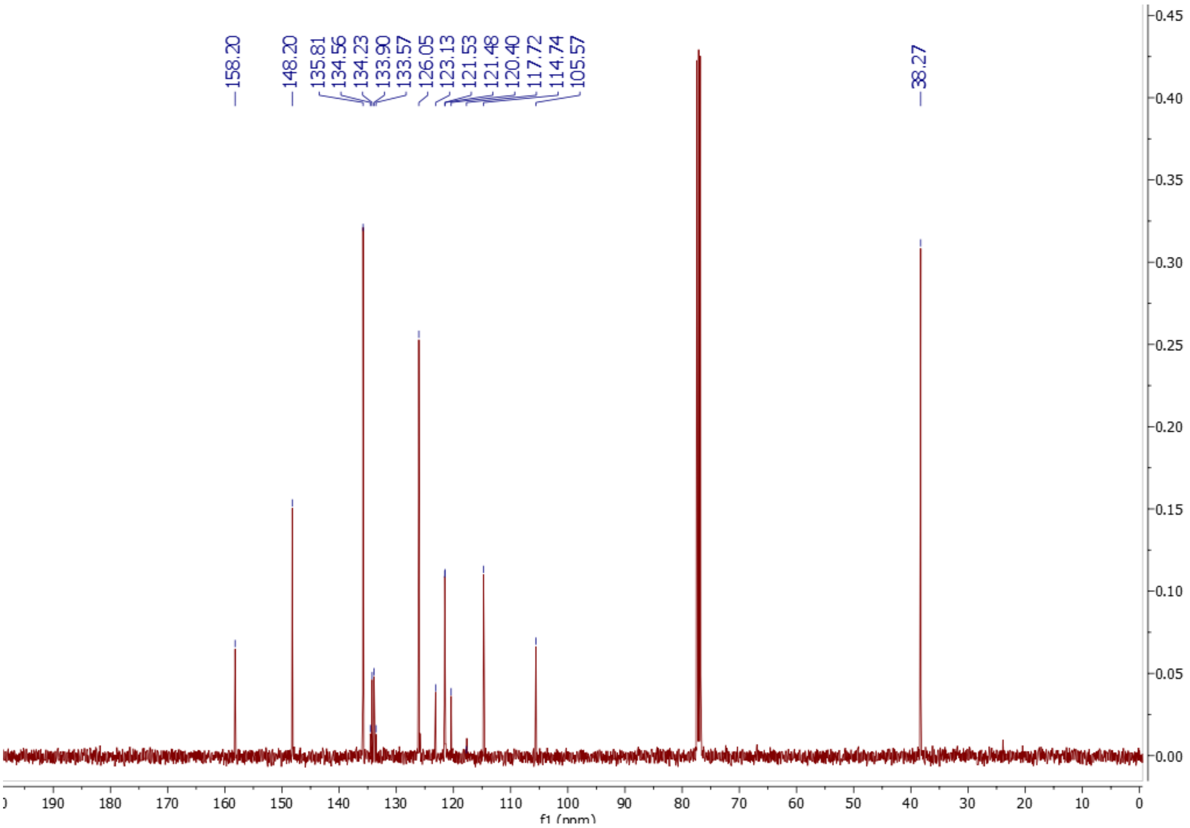

*Supplementary Figure 23. ¹³C NMR of N,N'-bis(4-cyano-3-(trifluoromethyl)phenyl)-N,N'-dimethylurea (**1f**) (101 MHz, 20 °C, $CDCl_3$)*

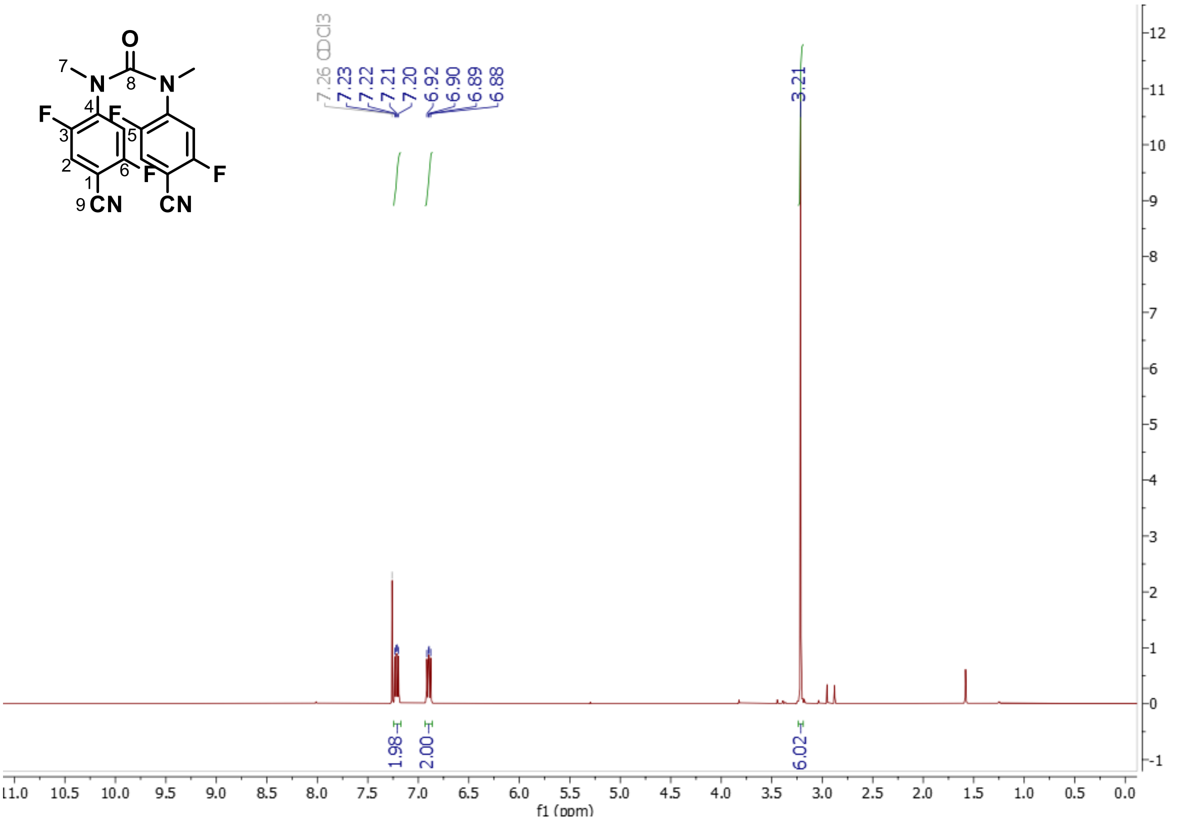

Supplementary Figure 24. ^{1}H NMR of N,N'-bis(4-cyano-2,5-difluorophenyl)-N,N'-dimethylurea (**1g**) (400 MHz, 20 °C, $CDCl_3$)

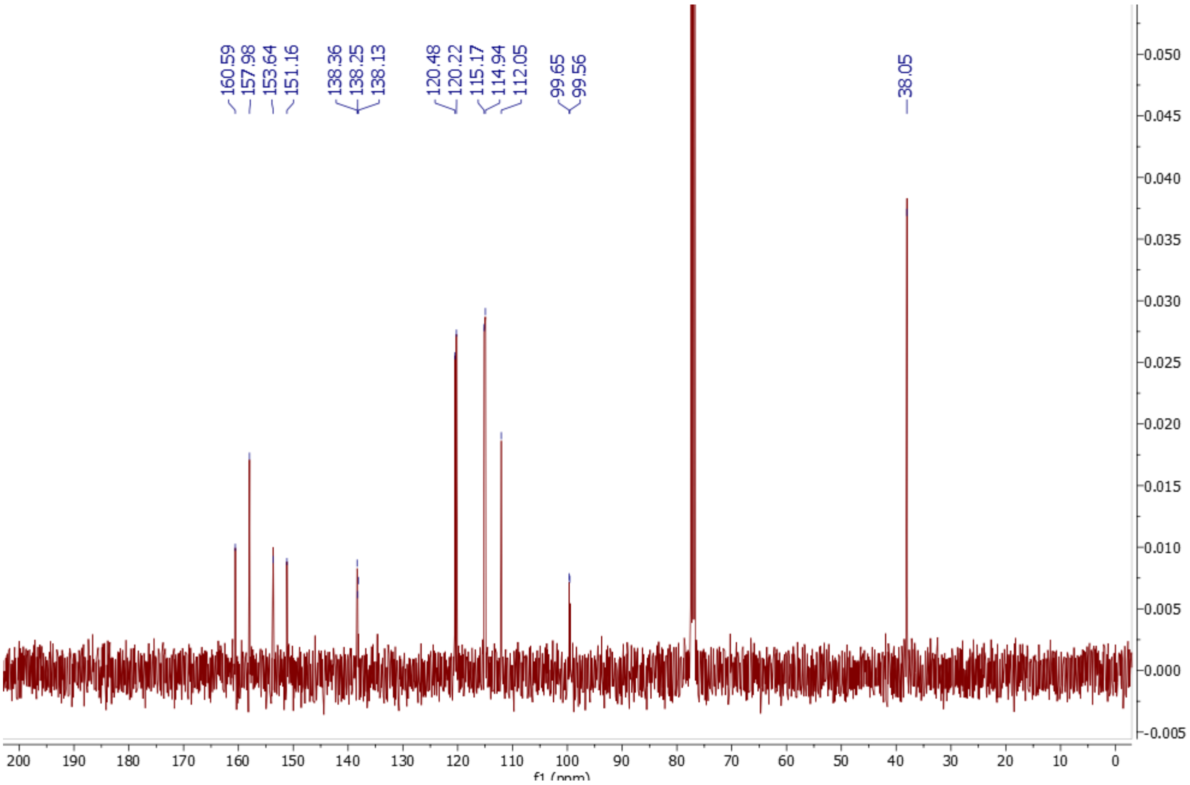

Supplementary Figure 25. ^{13}C NMR of N,N'-bis(4-cyano-2,5-difluorophenyl)-N,N'-dimethylurea (**1g**) (101 MHz, 20 °C, $CDCl_3$)

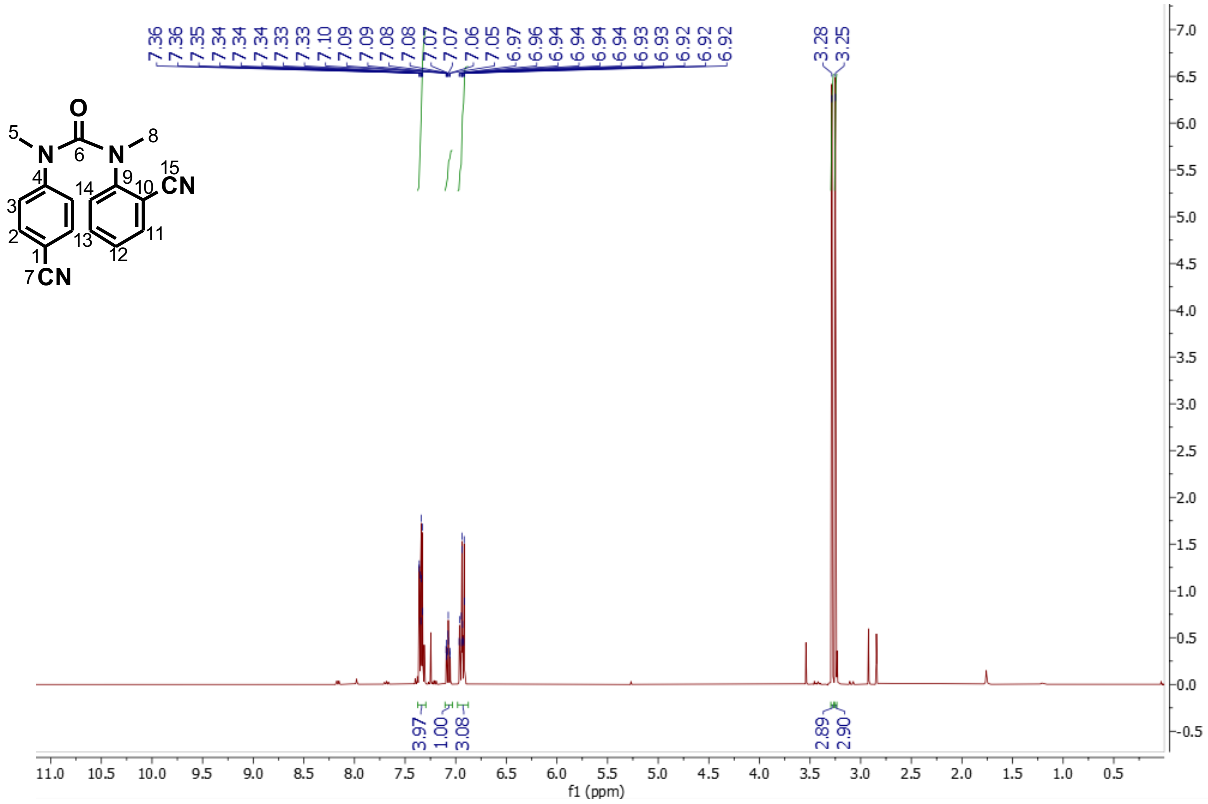

*Supplementary Figure 26. ^{1}H NMR of N-(2-cyanophenyl)-N'-(4-cyanophenyl)-N,N'-dimethylurea (**1h**) (400 MHz, 20 °C, $CDCl_3$)*

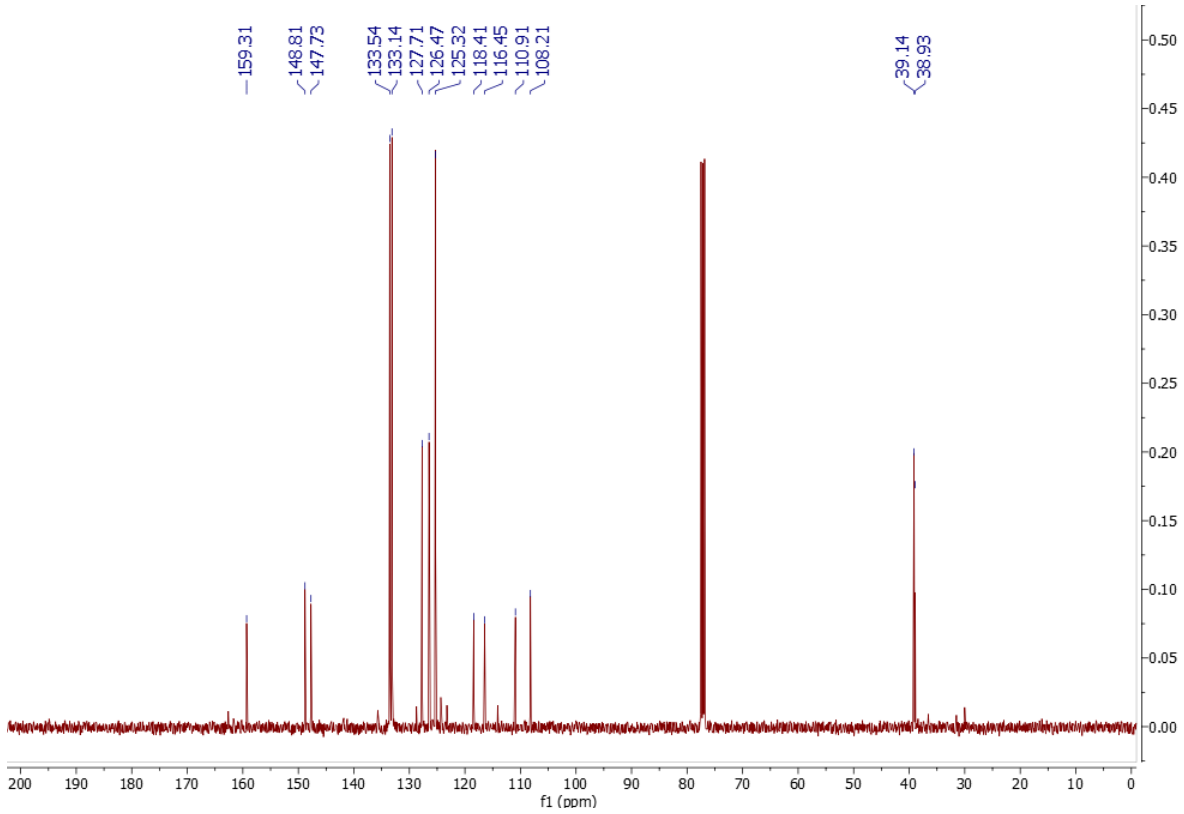

*Supplementary Figure 27. ^{13}C NMR of N-(2-cyanophenyl)-N'-(4-cyanophenyl)-N,N'-dimethylurea (**1h**) (101 MHz, 20 °C, $CDCl_3$)*

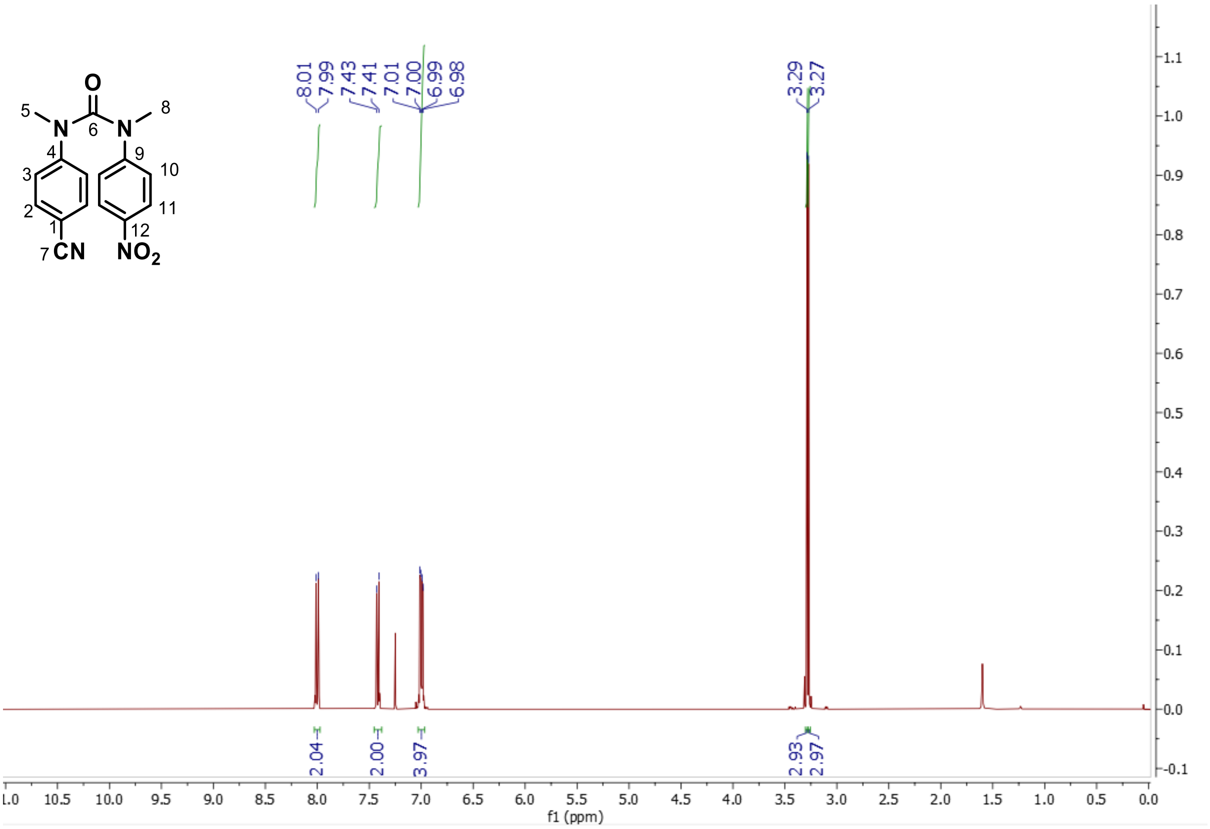

*Supplementary Figure 28. ^{1}H NMR of N-(4-cyanophenyl)-N,N'-dimethyl-N'-(4-nitrophenyl)urea (**1i**) (400 MHz, 20 °C, $CDCl_3$)*

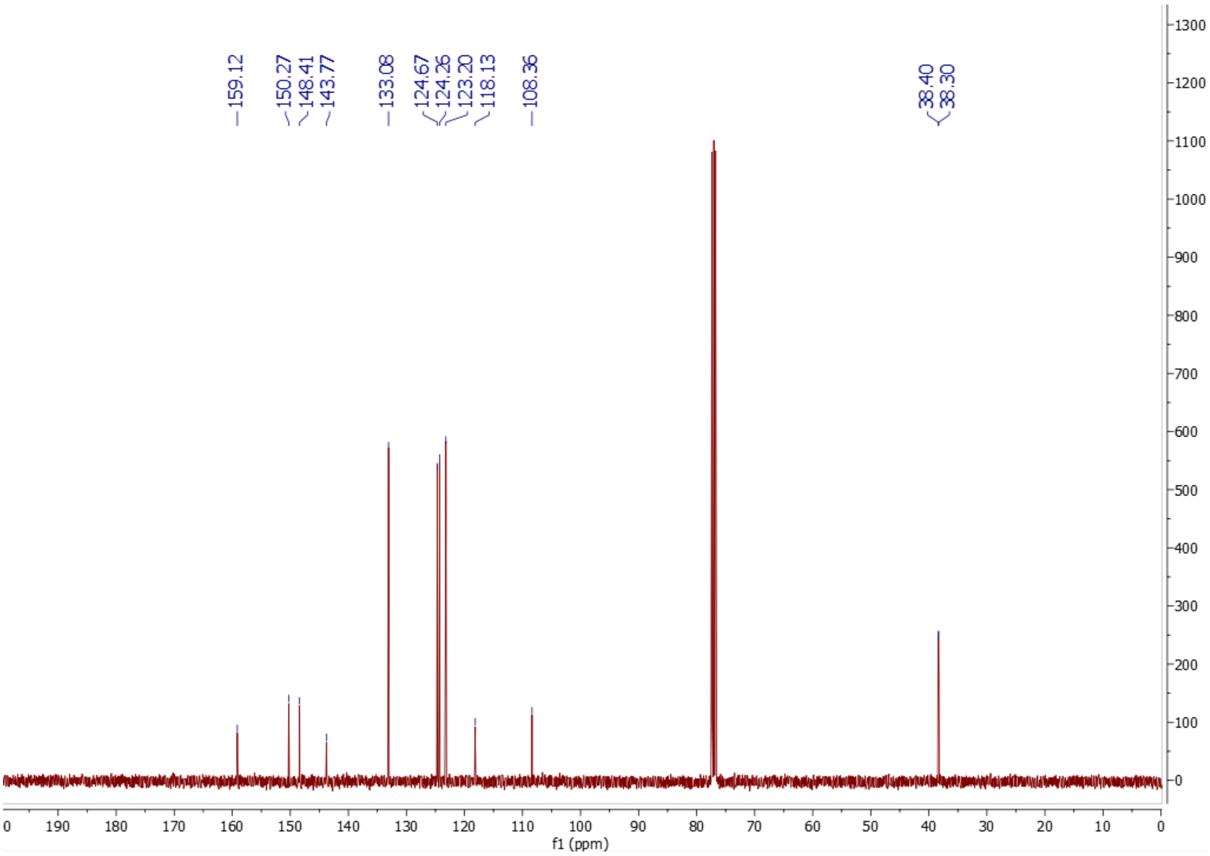

*Supplementary Figure 29. ^{13}C NMR of N-(4-cyanophenyl)-N,N'-dimethyl-N'-(4-nitrophenyl)urea (**1i**) (101 MHz, 20 °C, $CDCl_3$)*

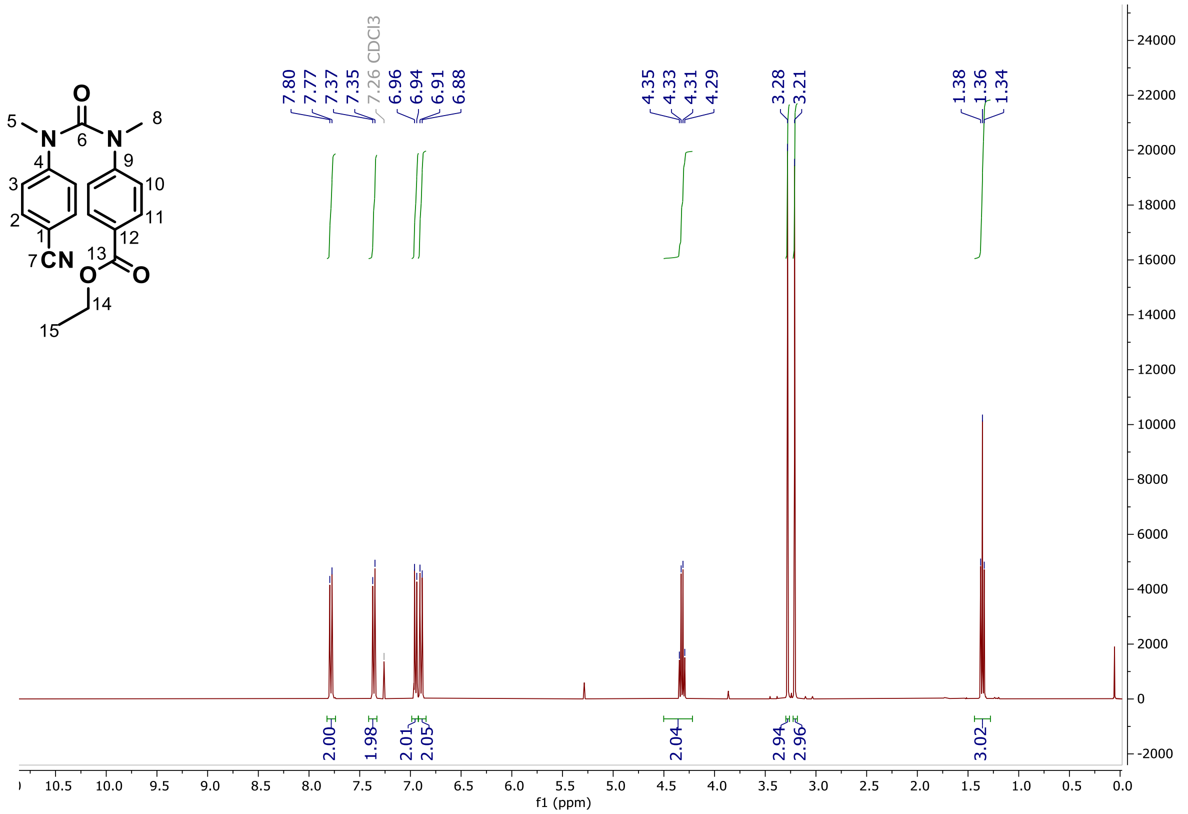

*Supplementary Figure 30. ¹H NMR of ethyl 4-(3-(4-cyanophenyl)-1,3-dimethylureido)benzoate (**1j**) (400 MHz, 20 °C, $CDCl_3$)*

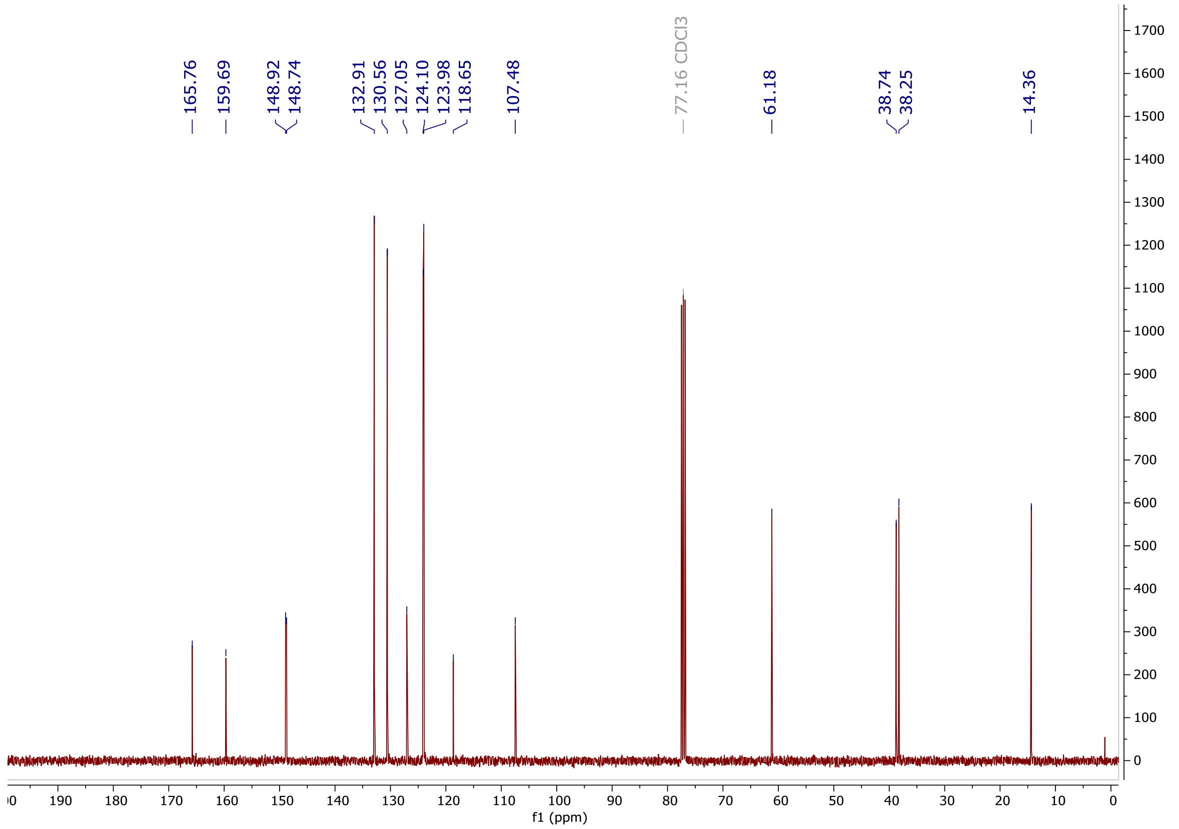

*Supplementary Figure 31. ¹³C NMR of ethyl 4-(3-(4-cyanophenyl)-1,3-dimethylureido)benzoate (**1j**) (101 MHz, 20 °C, $CDCl_3$)*

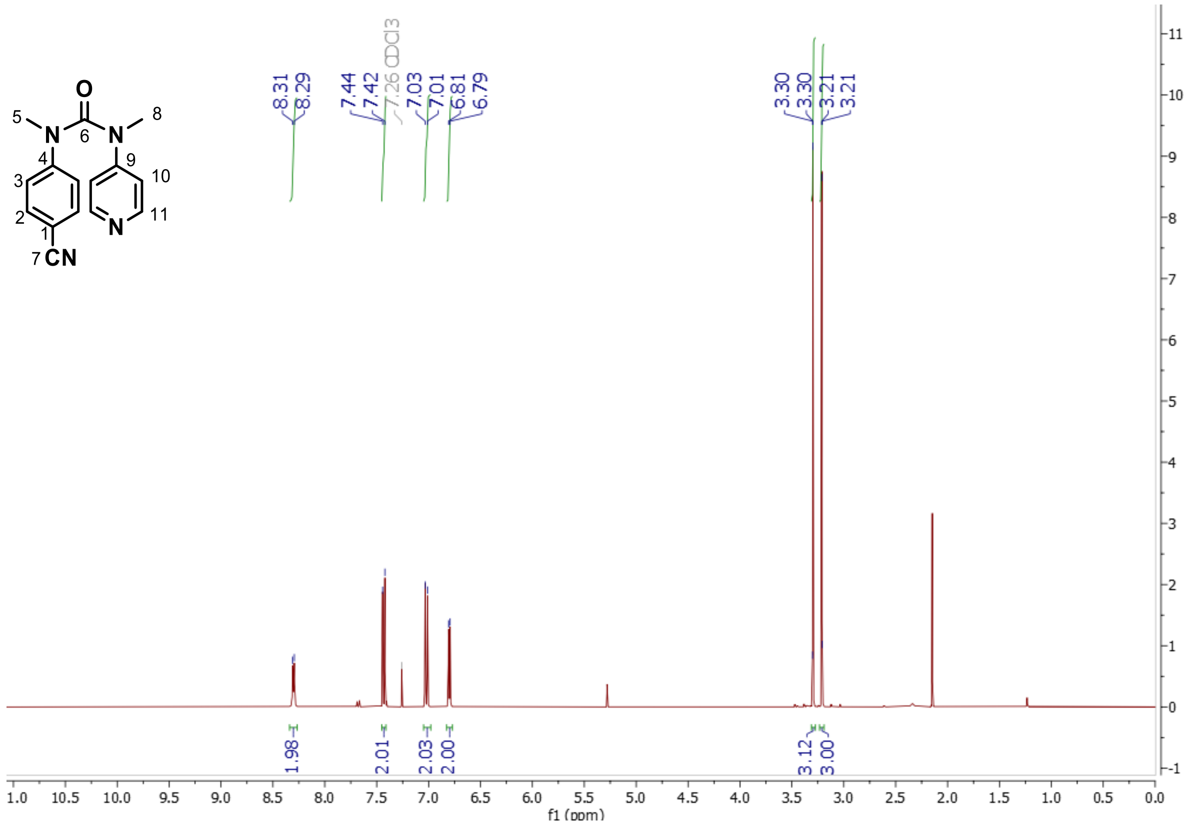

*Supplementary Figure 32. ¹H NMR of N-(4-cyanophenyl)-N,N'-dimethyl-N'-(pyridin-4-yl)urea (**1k**) (400 MHz, 20 °C, $CDCl_3$)*

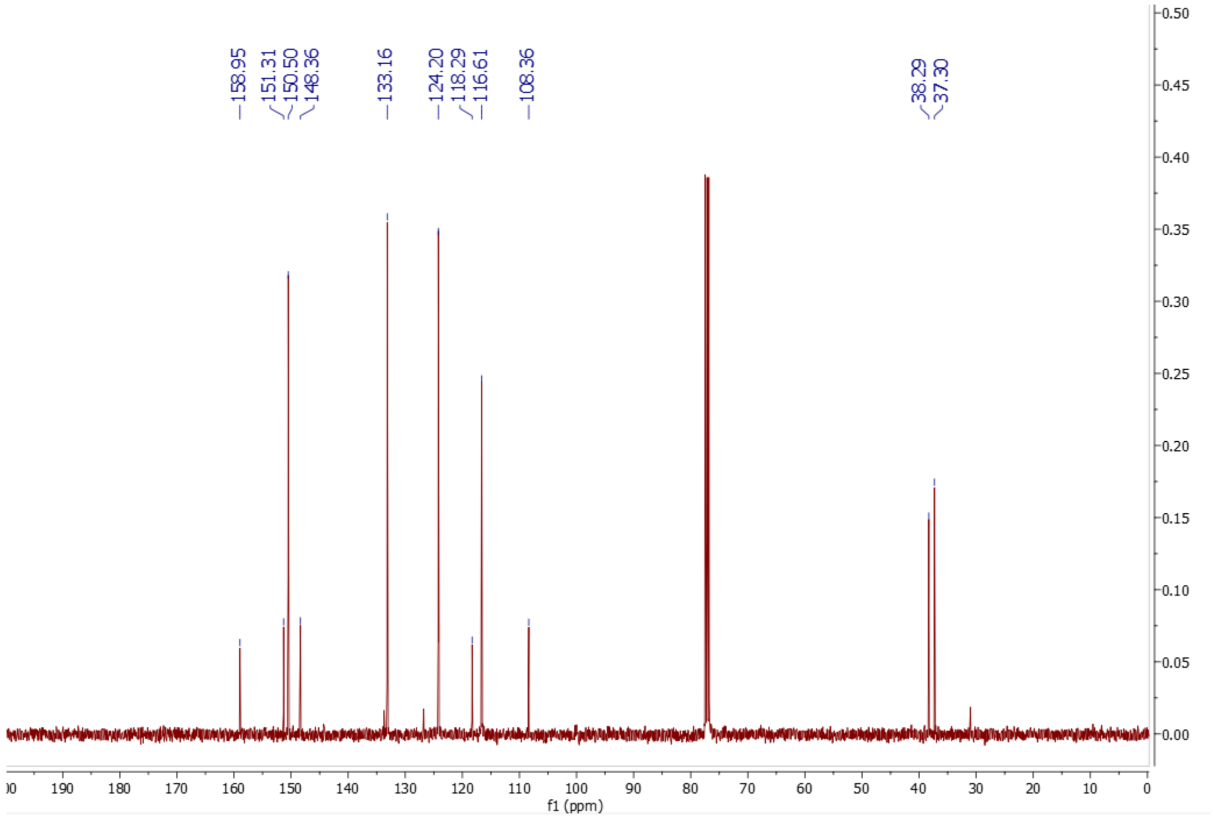

*Supplementary Figure 33.¹³C NMR of N-(4-cyanophenyl)-N,N'-dimethyl-N'-(pyridin-4-yl)urea (**1k**) (101 MHz, 20 °C, $CDCl_3$)*

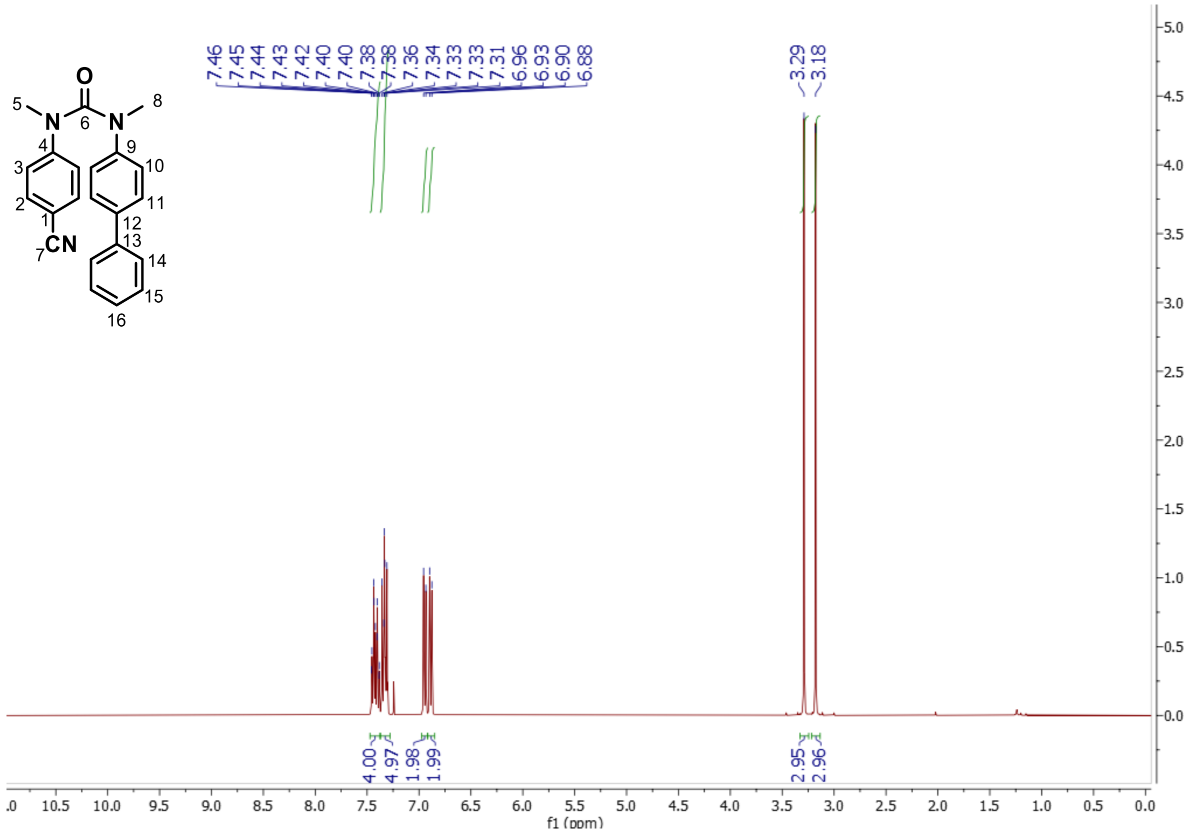

*Supplementary Figure 34. ^{1}H NMR of N-([1,1'-biphenyl]-4-yl)-N'-(4-cyanophenyl)-N,N'-dimethylurea (**1l**) (400 MHz, 20 °C, $CDCl_3$)*

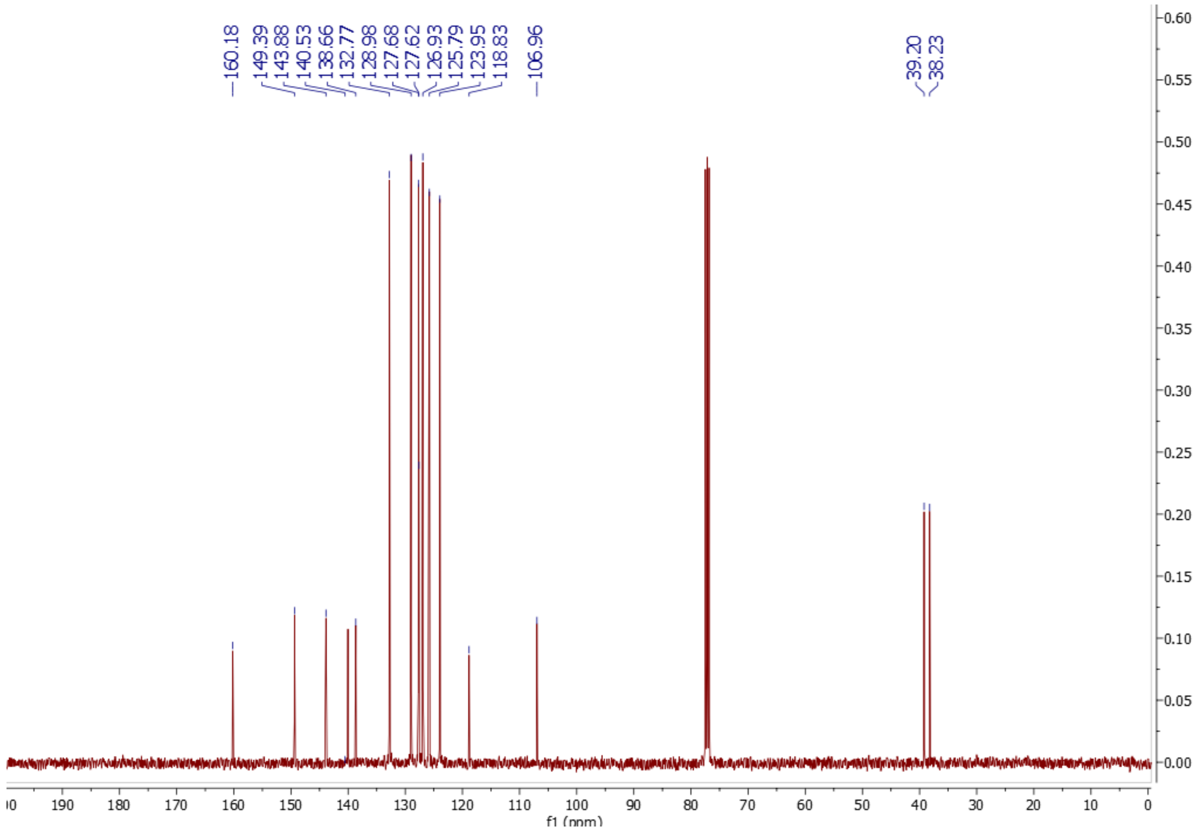

*Supplementary Figure 35. ^{13}C NMR of N-([1,1'-biphenyl]-4-yl)-N'-(4-cyanophenyl)-N,N'-dimethylurea (**1l**) (101 MHz, $CDCl_3$)*

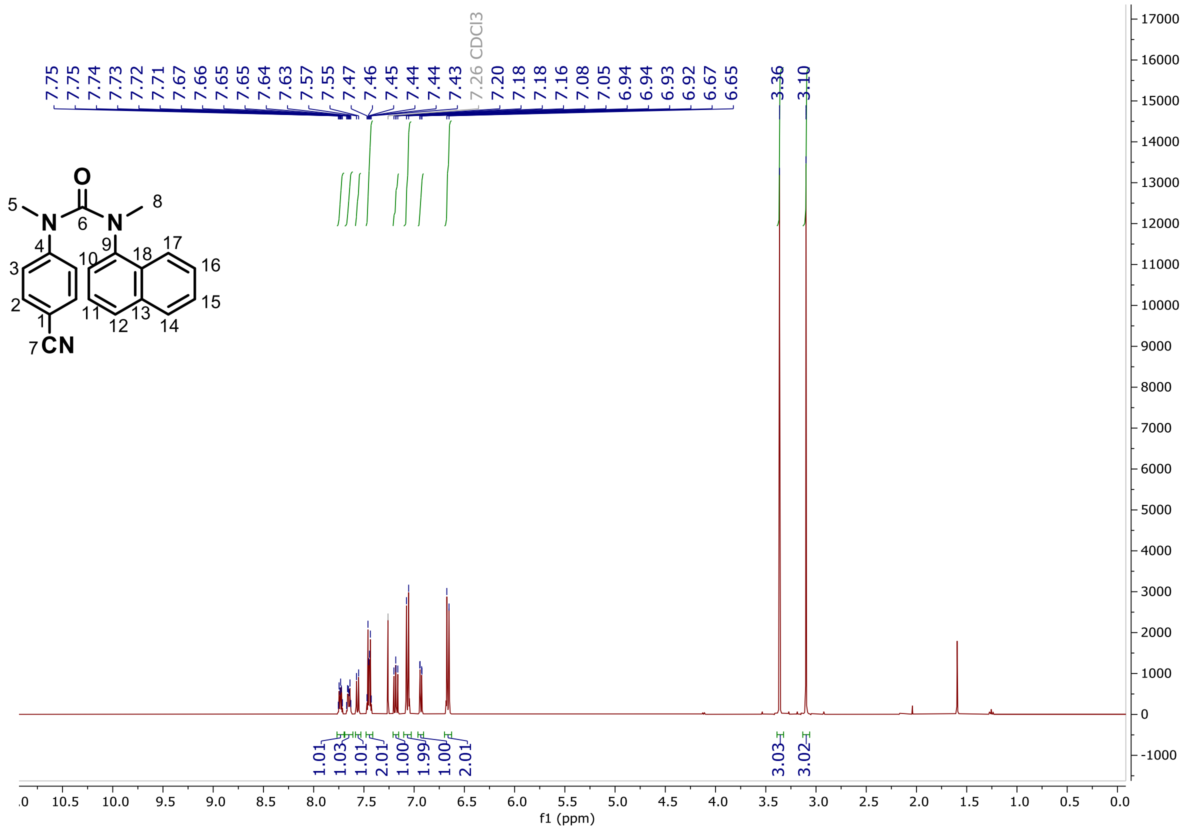

*Supplementary Figure 36. ^{1}H NMR of N-(4-cyanophenyl)-N,N'-dimethyl-N'-(naphthalen-1-yl)urea (**1m**) (400 MHz, 20 °C, $CDCl_3$)*

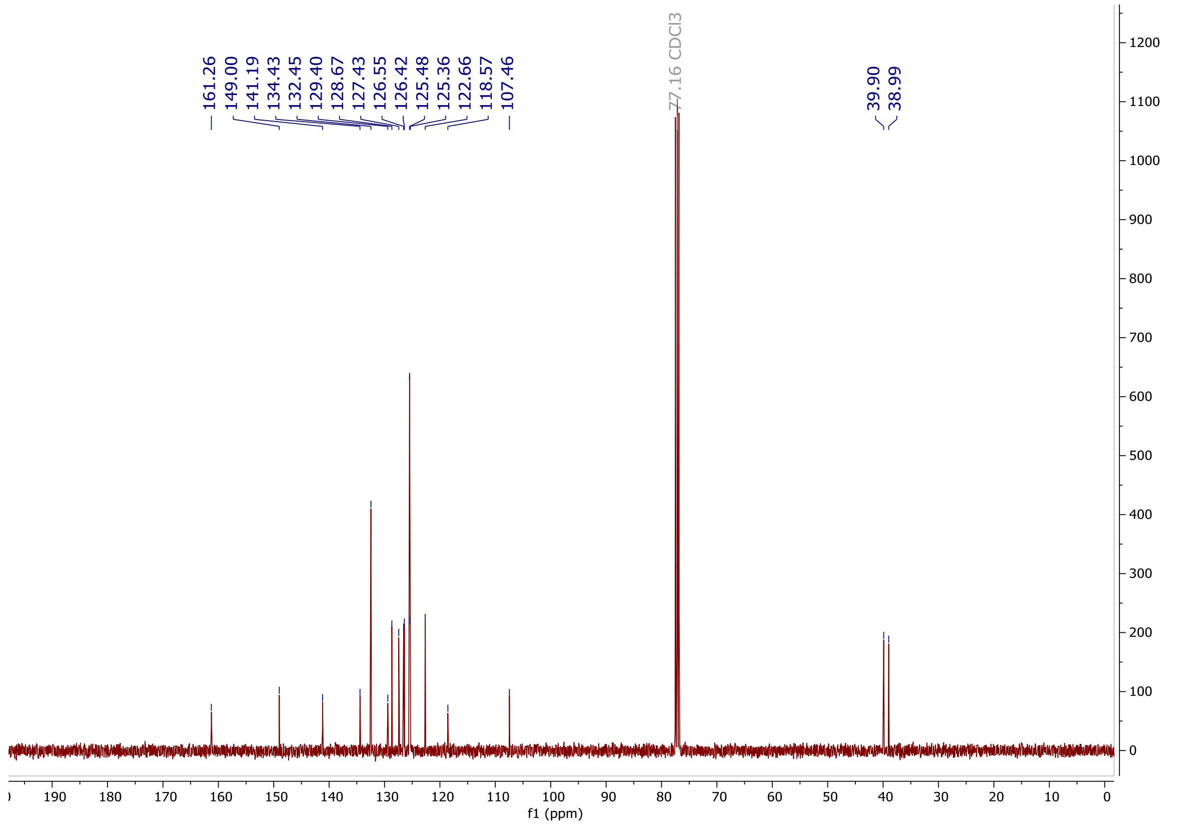

*Supplementary Figure 37. ^{13}C NMR of N-(4-cyanophenyl)-N,N'-dimethyl-N'-(naphthalen-1-yl)urea (**1m**) (101 MHz, 20 °C, $CDCl_3$)*

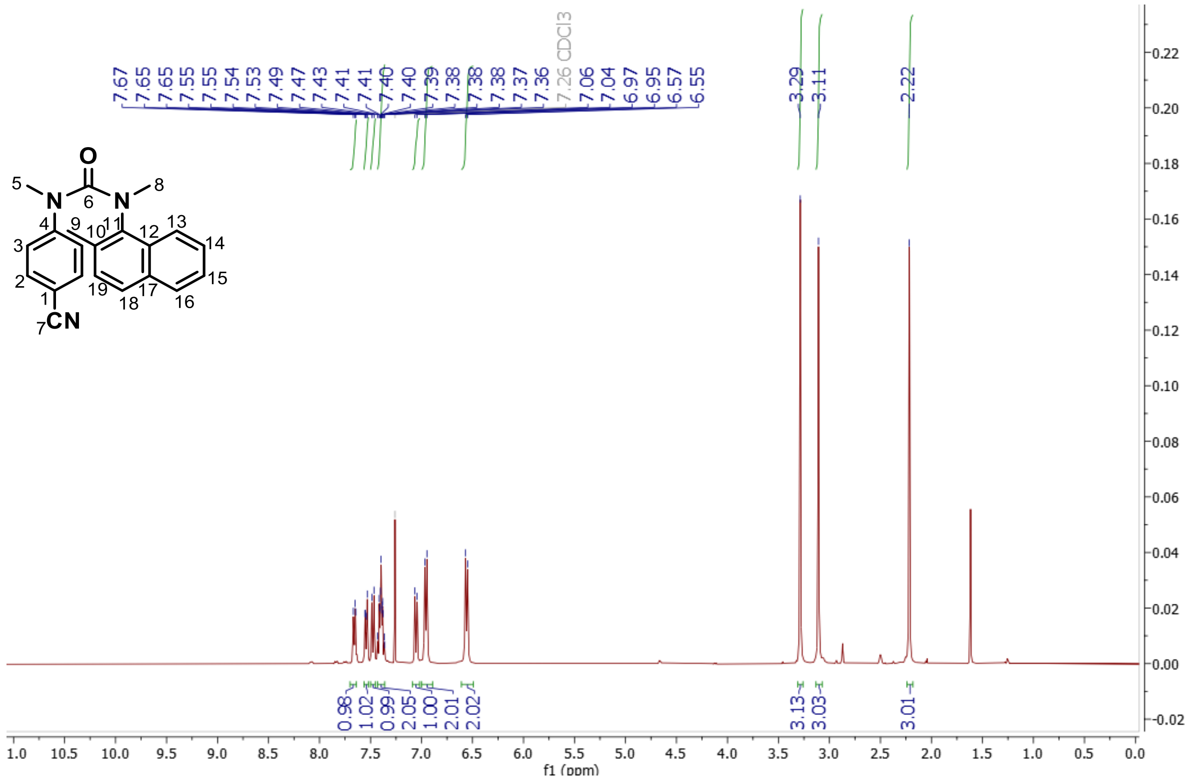

Supplementary Figure 38. ^{1}H NMR of N-(4-cyanophenyl)-N,N'-dimethyl-N'-(2-methylnaphthalen-1-yl)urea (**1n**) (400 MHz, 20 °C, $CDCl_3$)

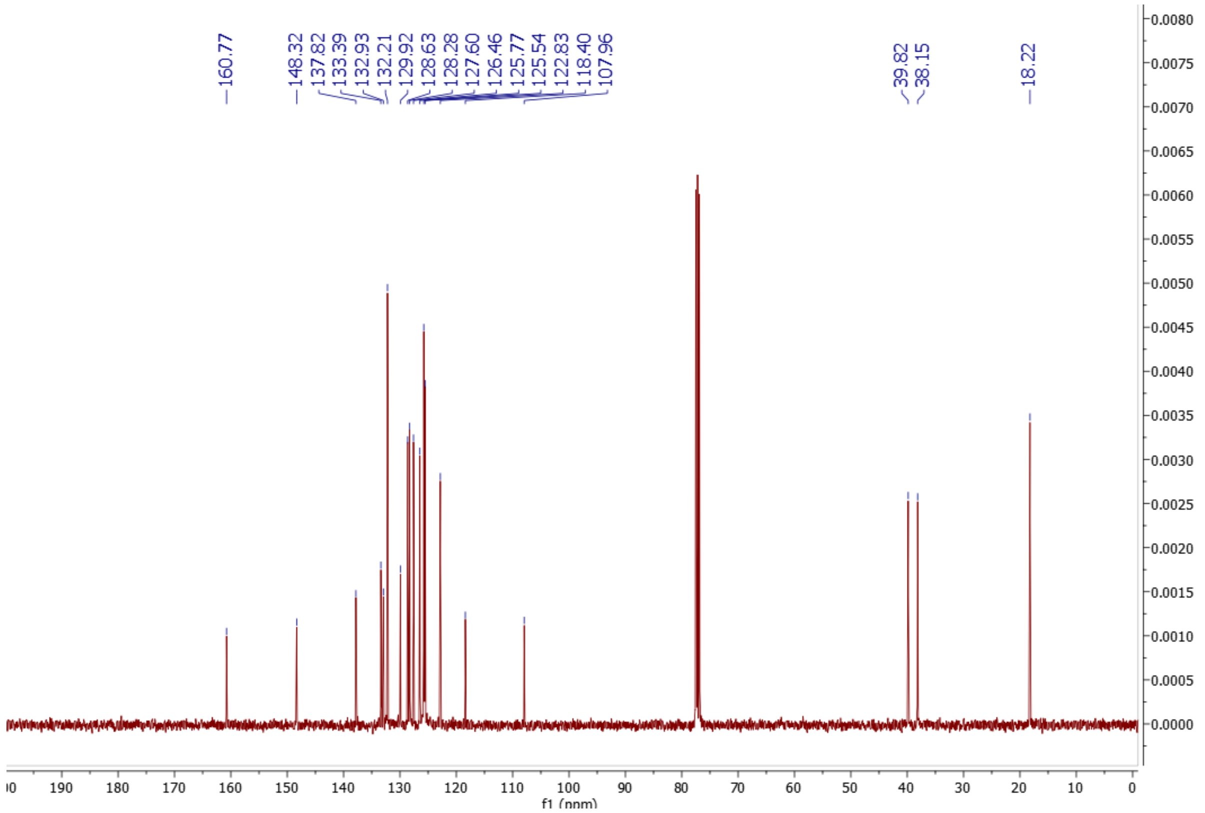

Supplementary Figure 39. ^{13}C NMR of N-(4-cyanophenyl)-N,N'-dimethyl-N'-(2-methylnaphthalen-1-yl)urea (**1n**) (101 MHz, 20 °C, $CDCl_3$)

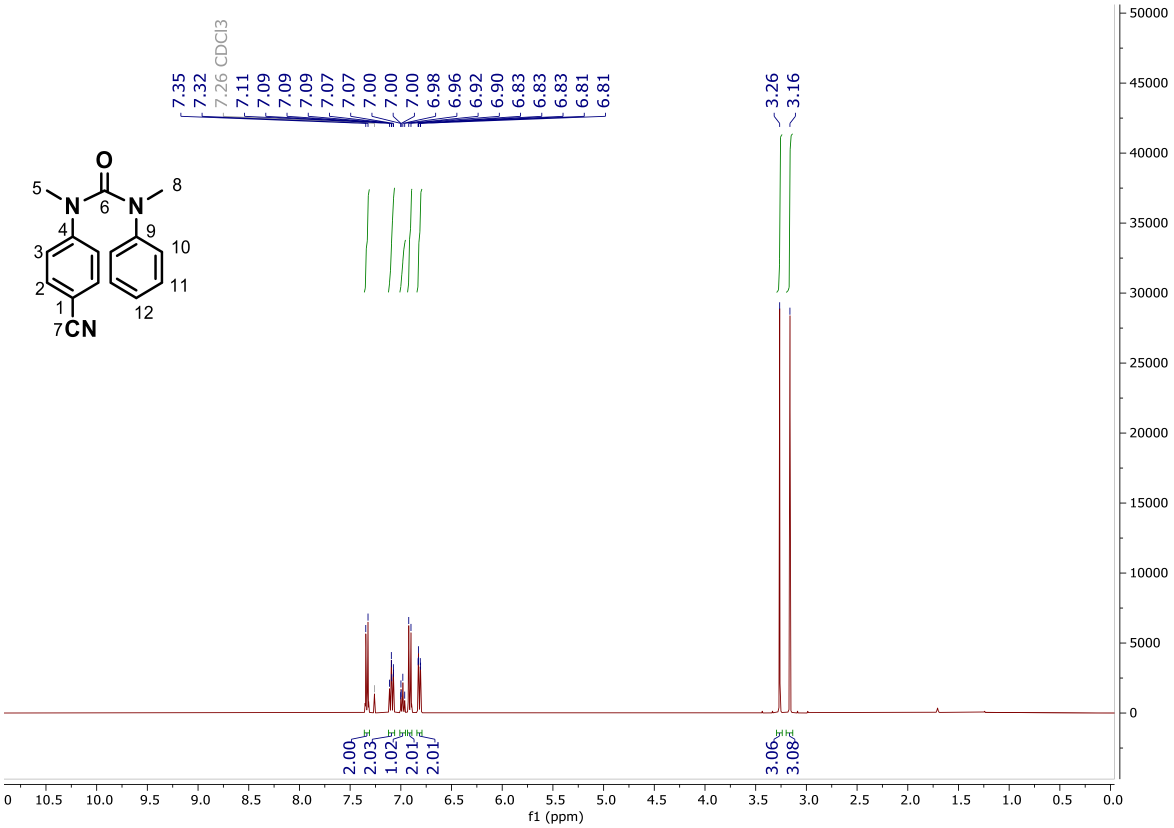

*Supplementary Figure 40. ^{1}H NMR of N-(4-cyanophenyl)-N,N'-dimethyl-N'-phenylurea (**1o**) (400 MHz, 20 °C, $CDCl_3$)*

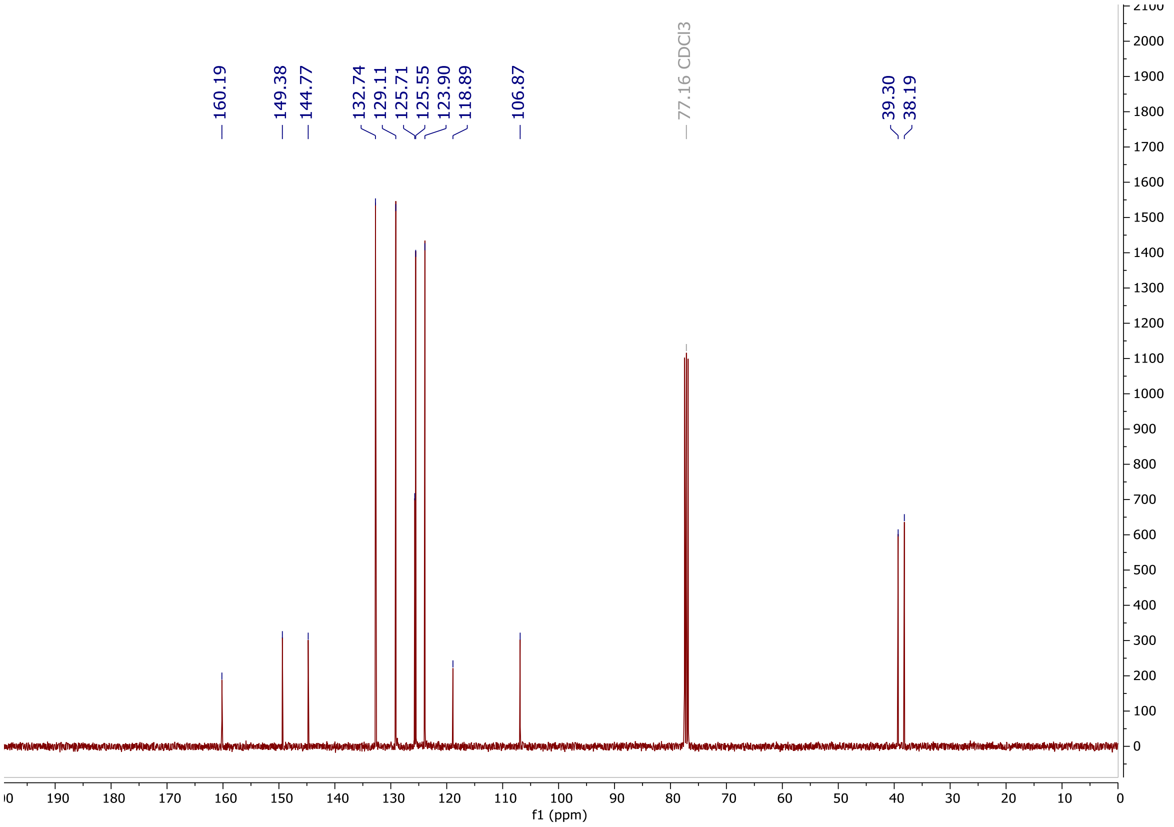

*Supplementary Figure 41. ^{13}C NMR of N-(4-cyanophenyl)-N,N'-dimethyl-N'-phenylurea (**1o**) (101 MHz, 20 °C, $CDCl_3$)*

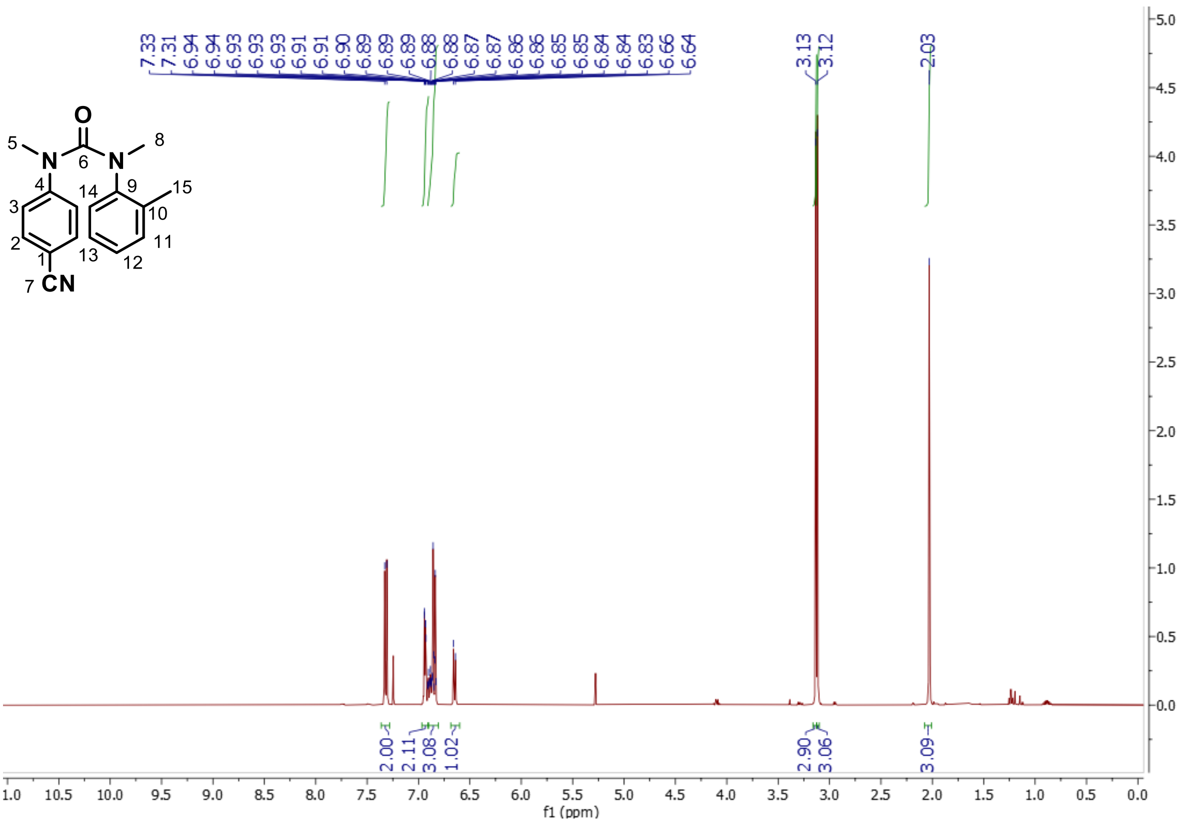

*Supplementary Figure 42. ¹H NMR of N-(4-cyanophenyl)-N,N'-dimethyl-N'-(o-tolyl)urea (**1p**) (400 MHz, 20 °C, CDCl₃)*

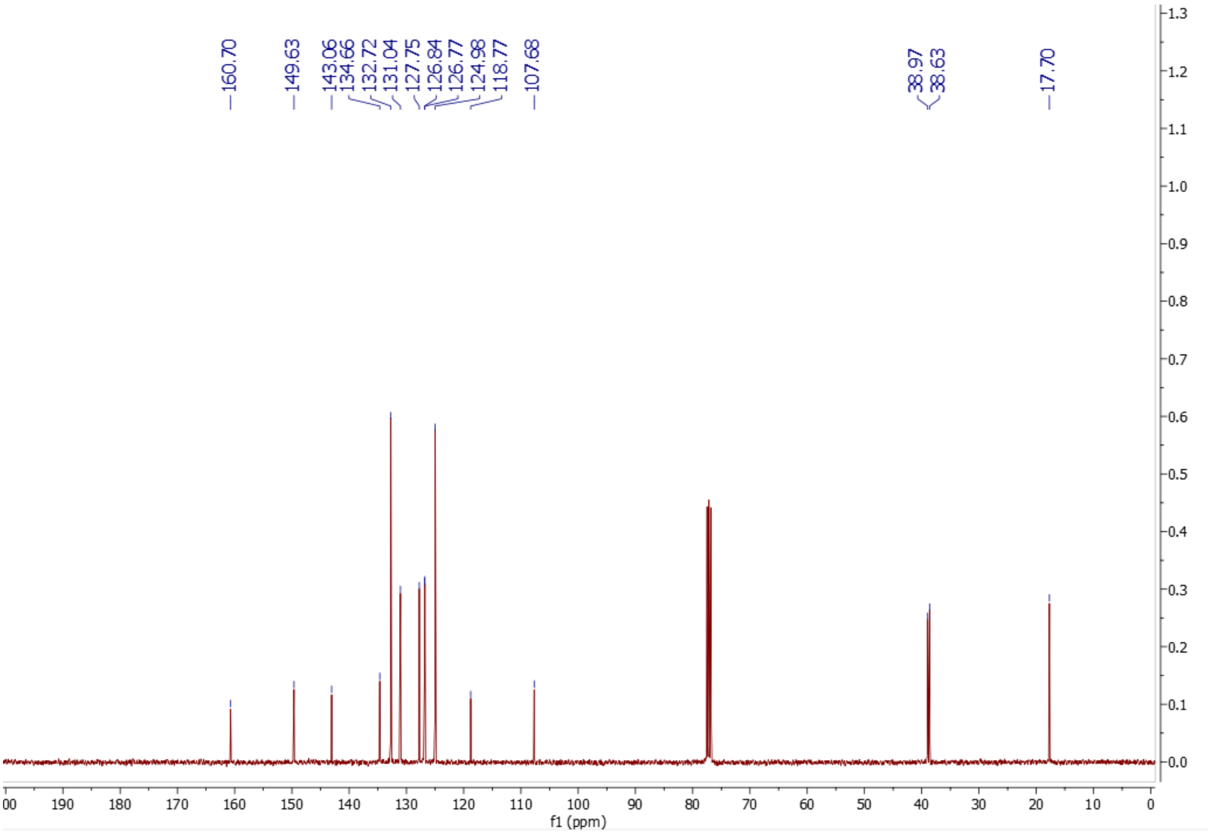

*Supplementary Figure 43. ¹³C NMR of N-(4-cyanophenyl)-N,N'-dimethyl-N'-(o-tolyl)urea (**1p**) (101 MHz, 20 °C, CDCl₃)*

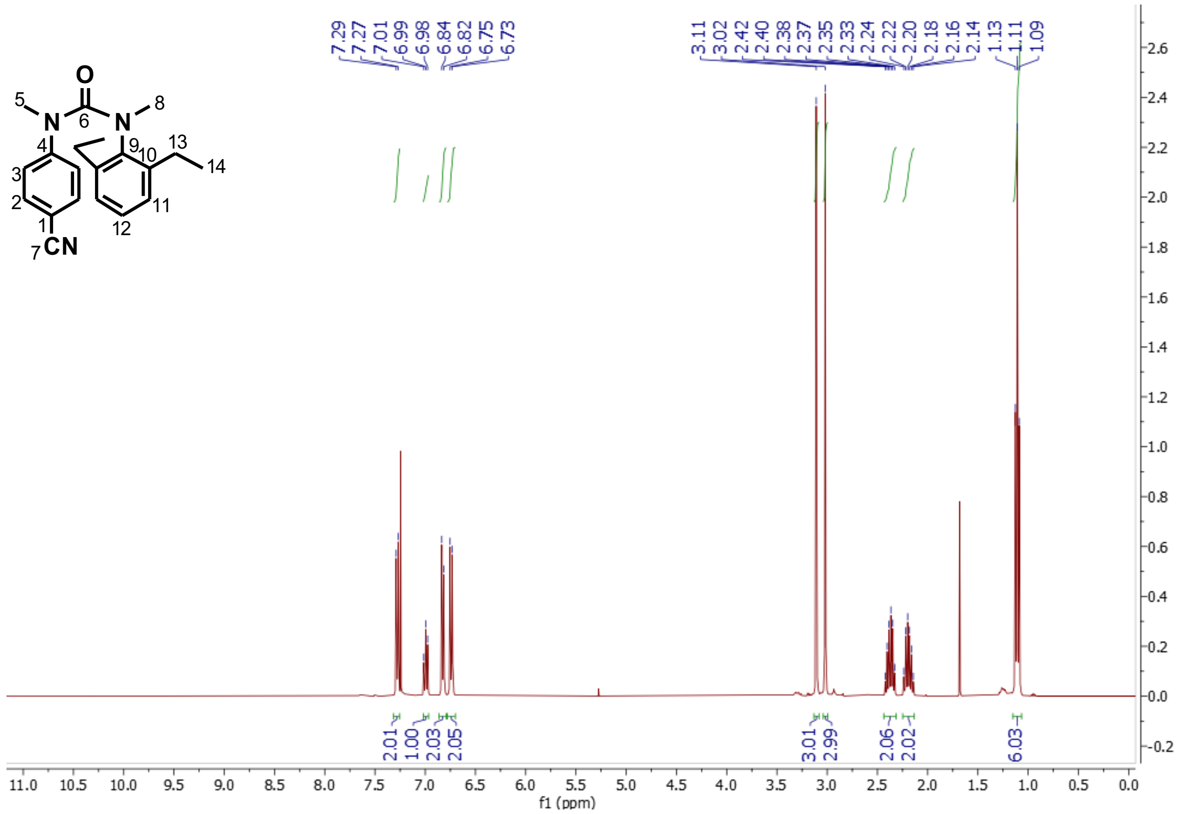

*Supplementary Figure 44. ^{1}H NMR of N-(4-cyanophenyl)-N'-(2,6-diethylphenyl)-N,N'-dimethylurea (**1q**) (400 MHz, 20 °C, $CDCl_3$)*

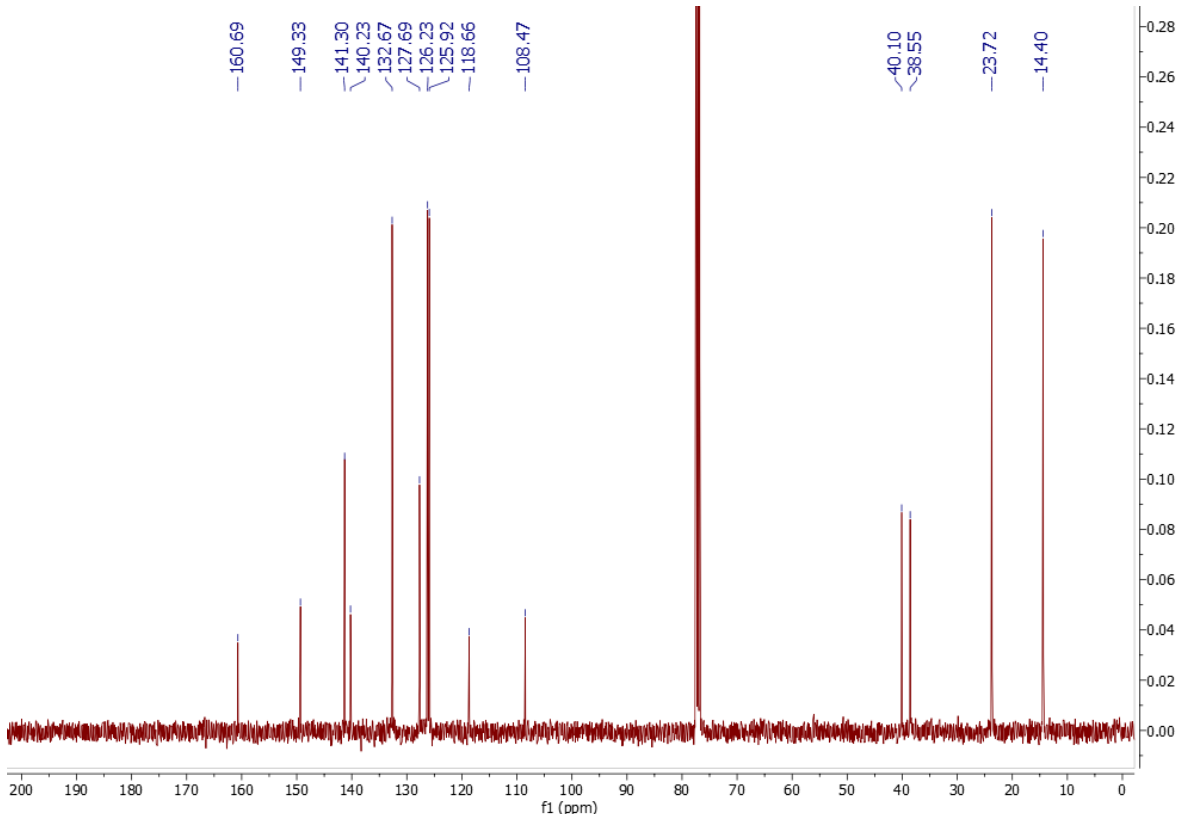

*Supplementary Figure 45. ^{13}C NMR of N-(4-cyanophenyl)-N'-(2,6-diethylphenyl)-N,N'-dimethylurea (**1q**) (101 MHz, 20 °C, $CDCl_3$)*

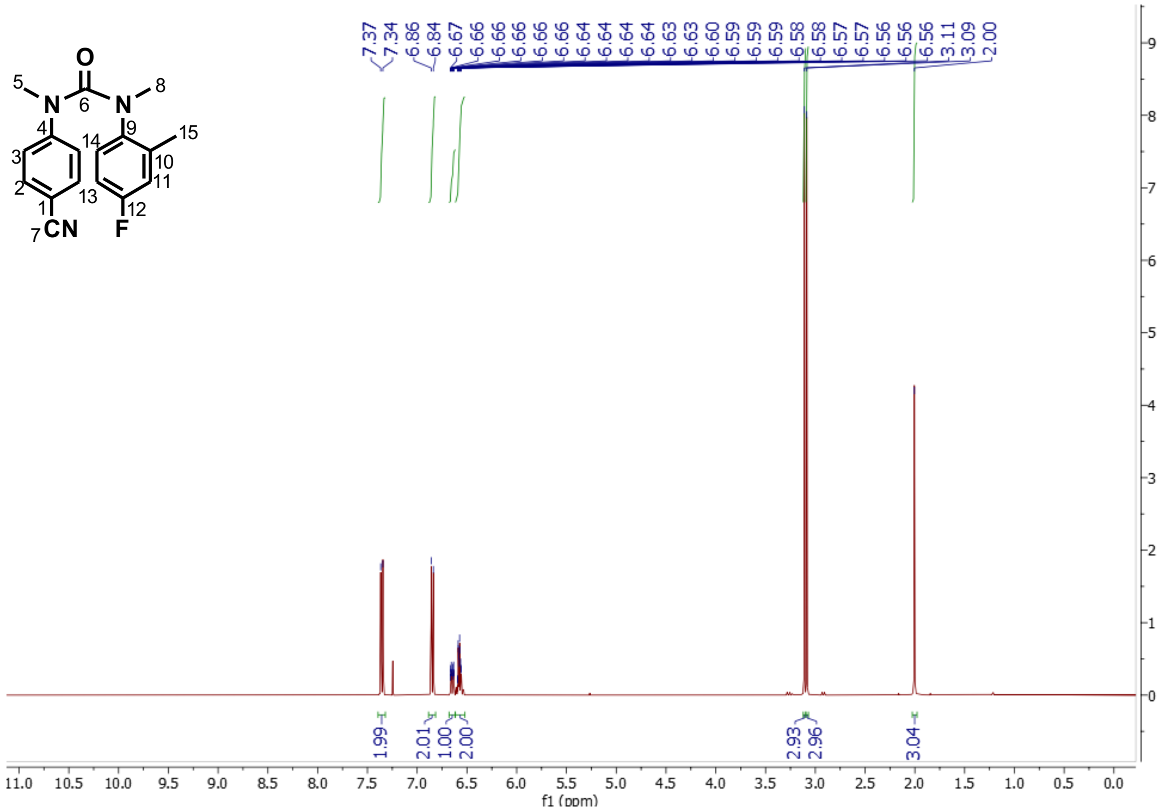

*Supplementary Figure 46. ¹H NMR of N-(4-cyanophenyl)-N'-(4-fluoro-2-methylphenyl)-N,N'-dimethylurea (**1r**) (400 MHz, 20 °C ,CDCl₃)*

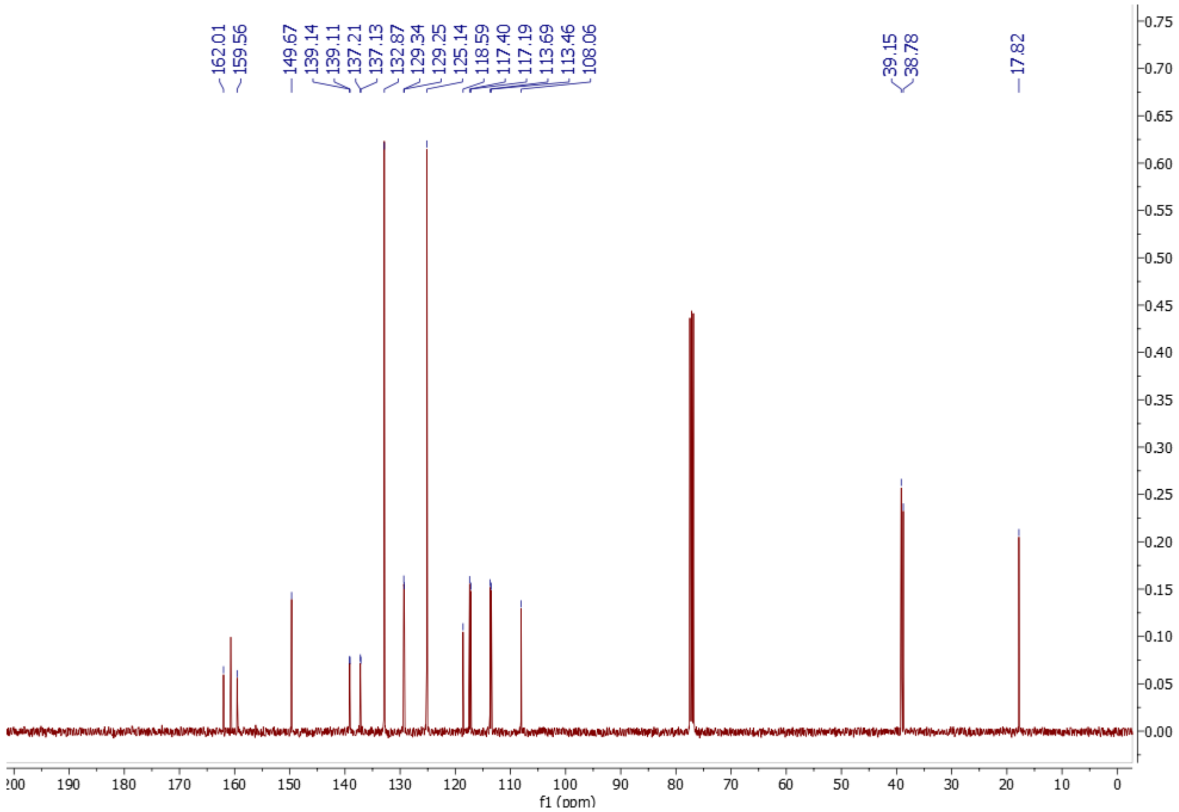

*Supplementary Figure 47. ¹³C NMR of N-(4-cyanophenyl)-N'-(4-fluoro-2-methylphenyl)-N,N'-dimethylurea (**1r**) (101 MHz, 20 °C, CDCl₃)*

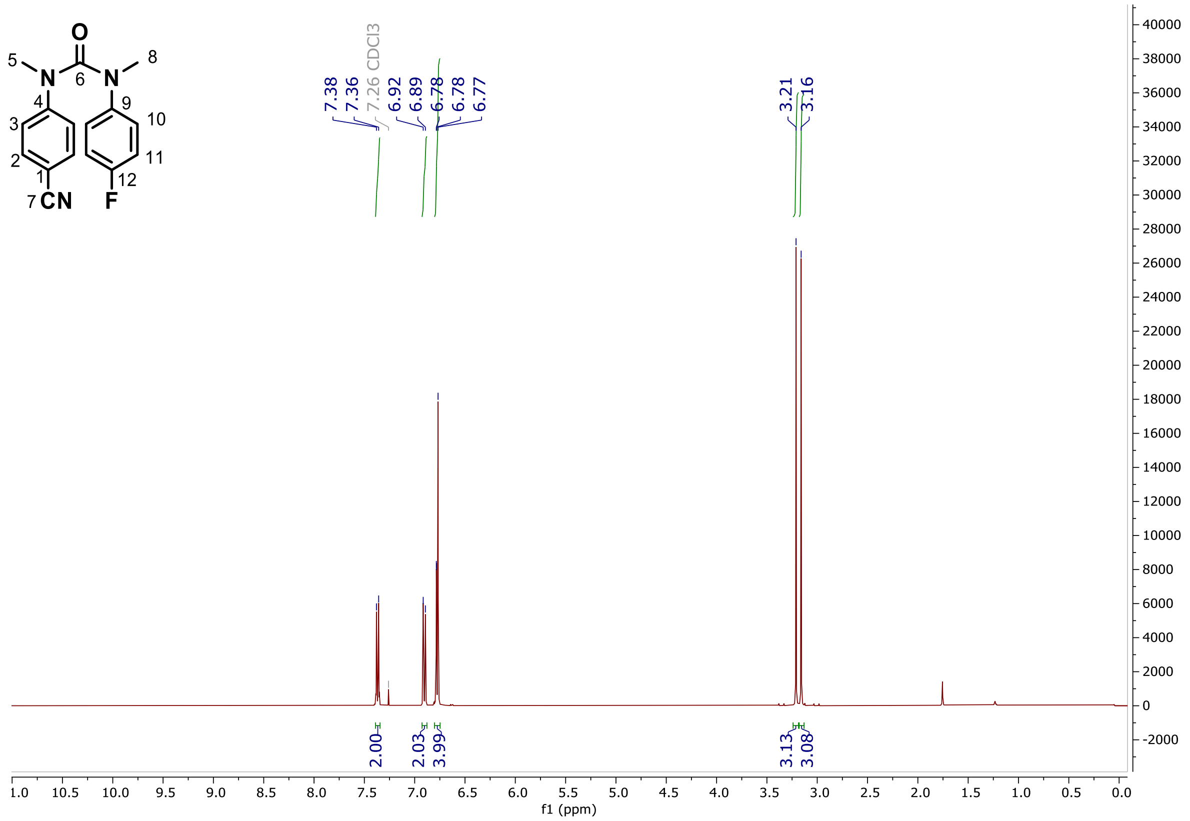

*Supplementary Figure 48. ¹H NMR of N-(4-cyanophenyl)-N'-(4-fluorophenyl)-N,N'-dimethylurea (**1s**) (400 MHz, 20 °C, $CDCl_3$)*

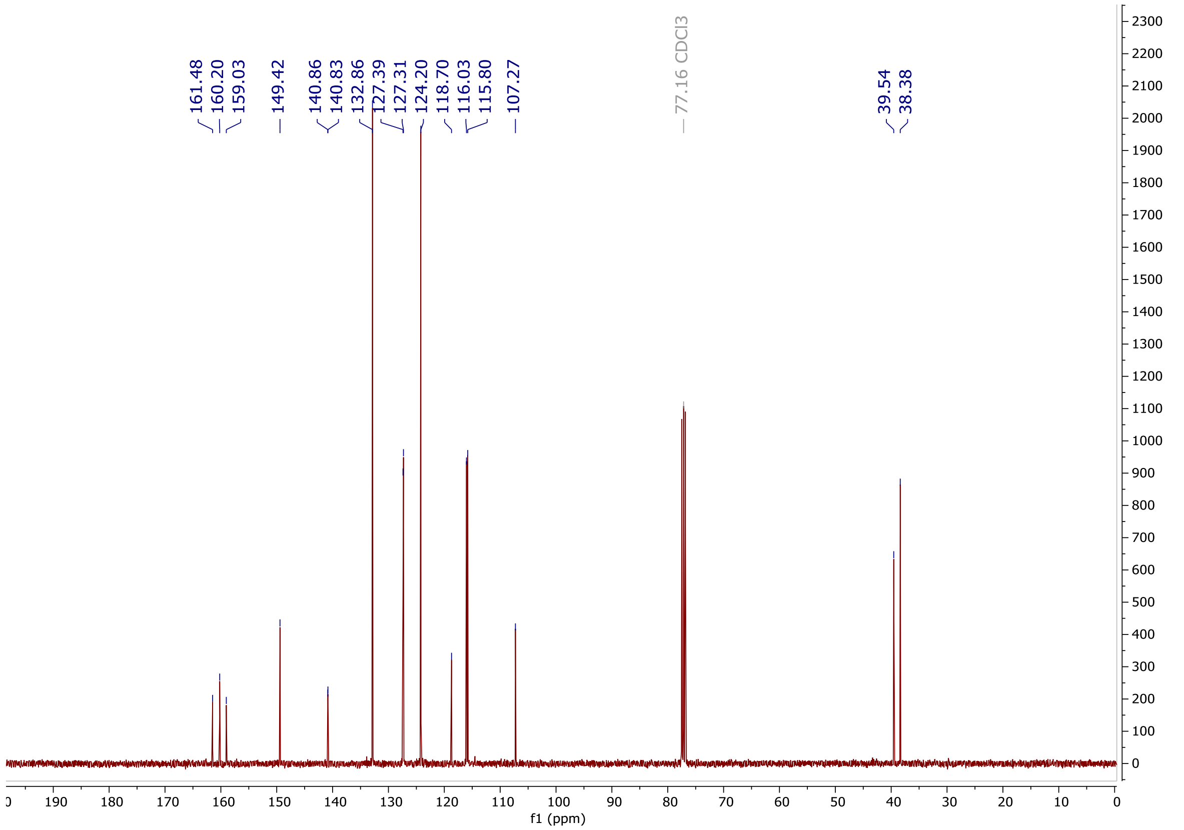

*Supplementary Figure 49. ¹³C NMR of N-(4-cyanophenyl)-N'-(4-fluorophenyl)-N,N'-dimethylurea (**1s**) (101 MHz, 20 °C, $CDCl_3$)*

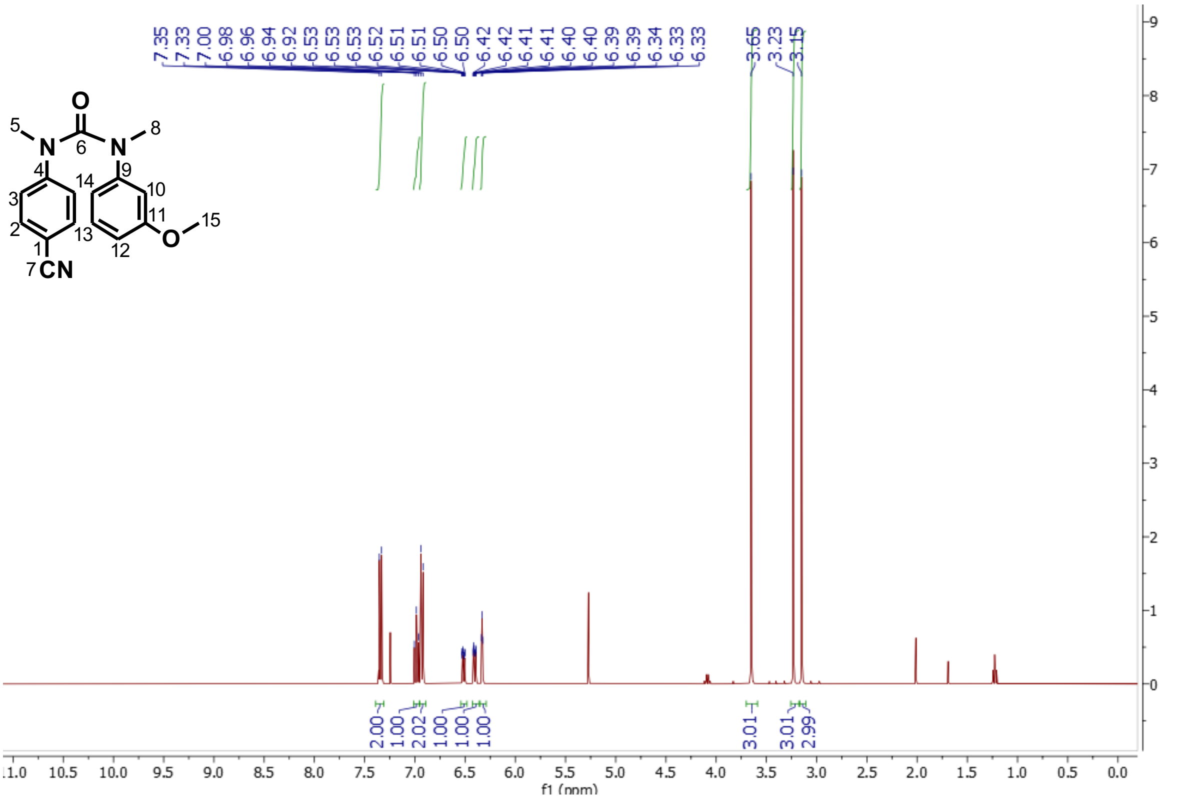

*Supplementary Figure 50. ^{1}H NMR of N-(4-cyanophenyl)-N'-(3-methoxyphenyl)-N,N'-dimethylurea (**1t**) (400 MHz, 20 °C, $CDCl_3$)*

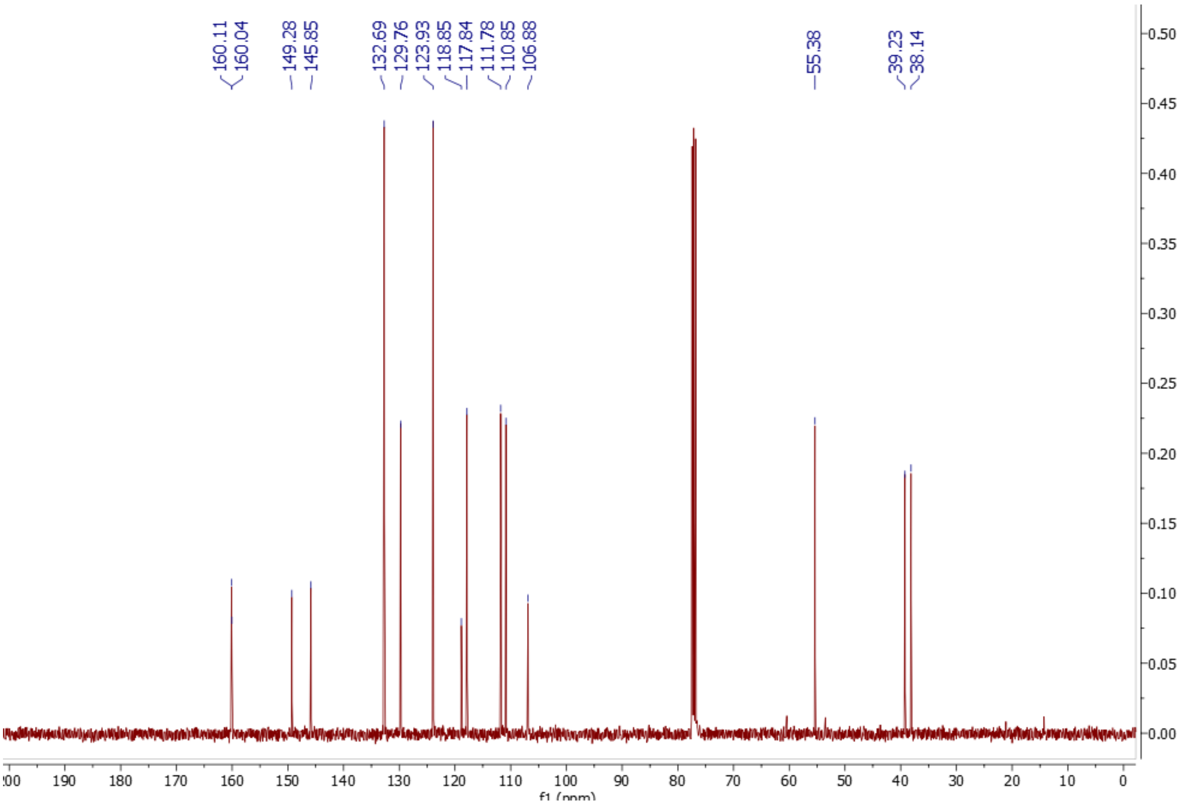

*Supplementary Figure 51. ^{13}C NMR of N-(4-cyanophenyl)-N'-(3-methoxyphenyl)-N,N'-dimethylurea (**1t**) (101 MHz, 20 °C, $CDCl_3$)*

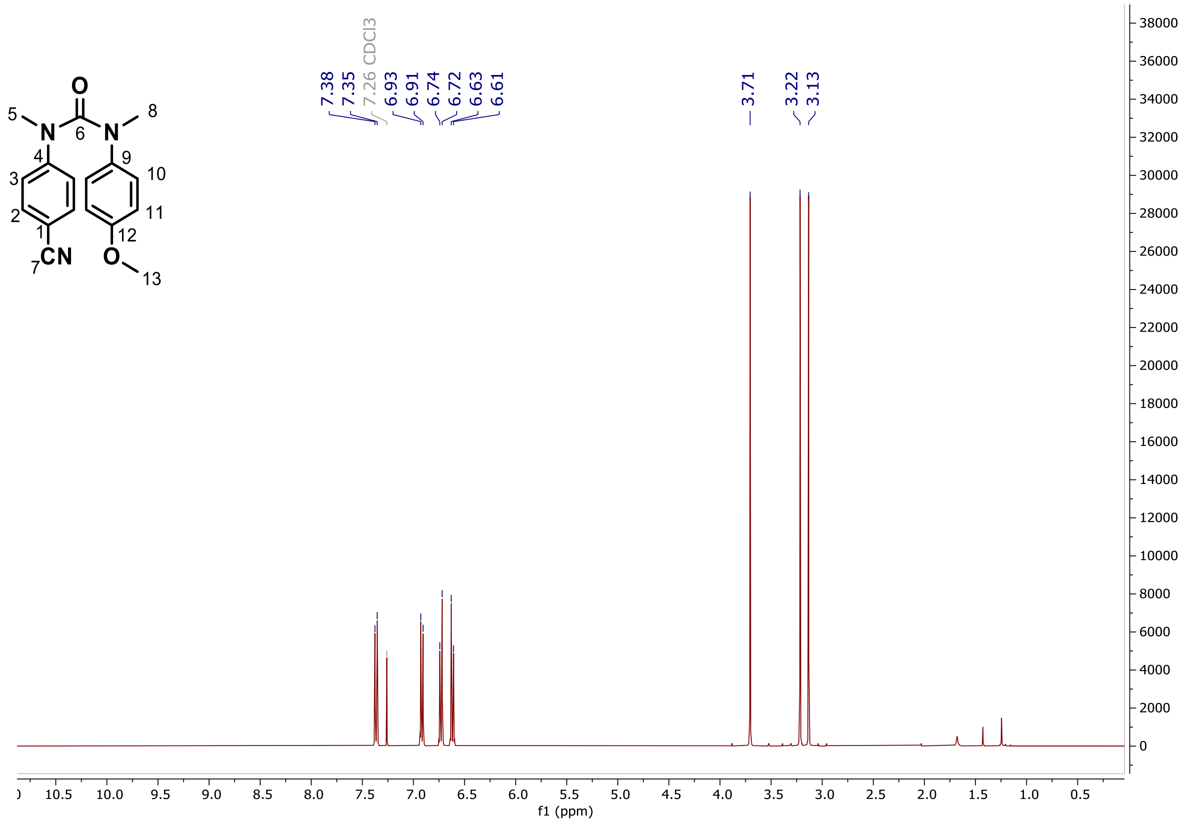

*Supplementary Figure 52. ^{1}H NMR of N-(4-cyanophenyl)-N'-(4-methoxyphenyl)-N,N'-dimethylurea (**1u**) (400 MHz, 20 °C, $CDCl_3$)*

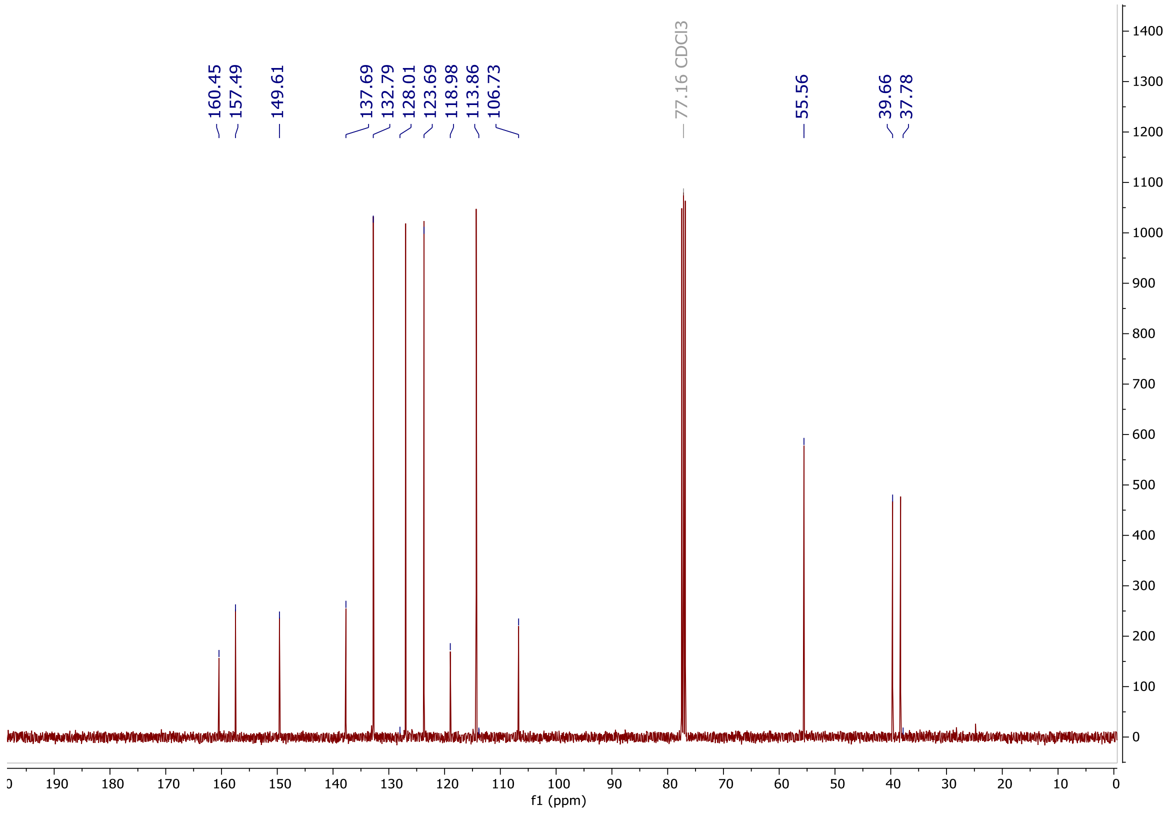

*Supplementary Figure 53. ^{13}C NMR of N-(4-cyanophenyl)-N'-(4-methoxyphenyl)-N,N'-dimethylurea (**1u**) (101 MHz, 20 °C, $CDCl_3$)*

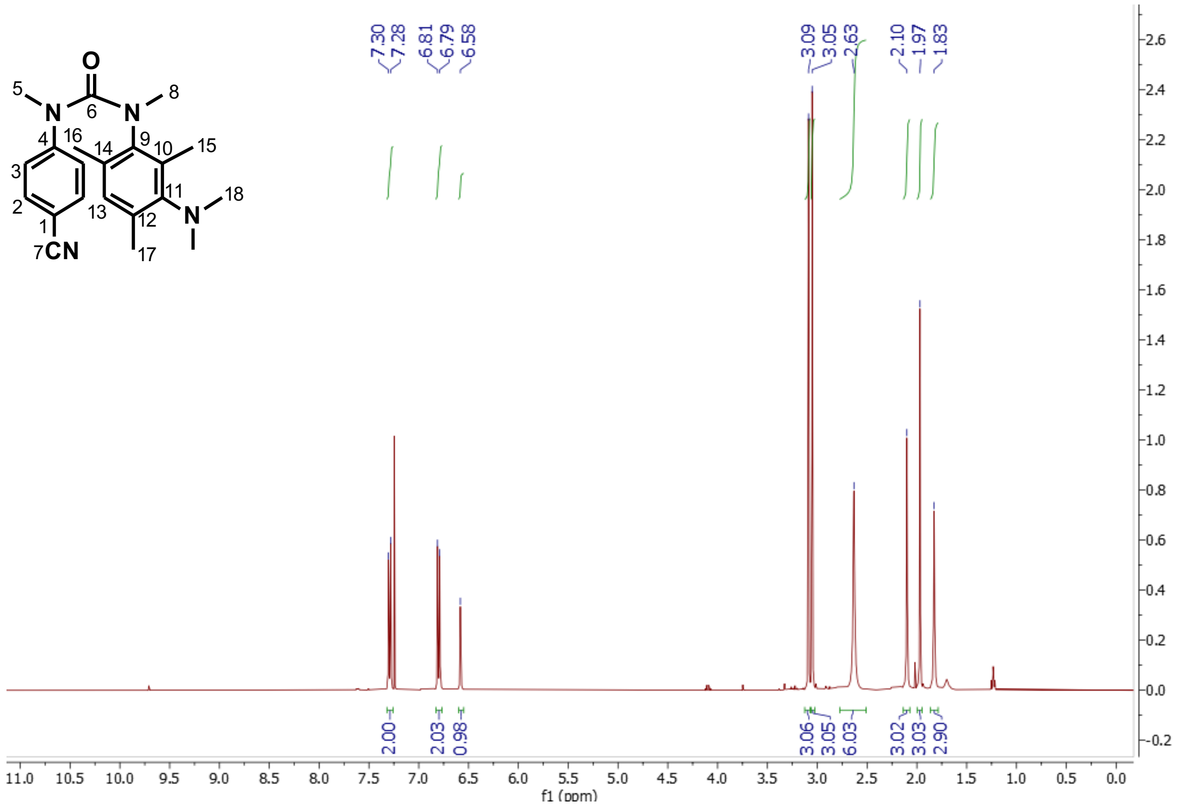

*Supplementary Figure 54. ¹H NMR of N-(4-cyanophenyl)-N'-(3-(dimethylamino)-2,4,6-trimethylphenyl)-N,N'-dimethylurea (**1v**) (400 MHz, 20 °C, CDCl$_3$)*

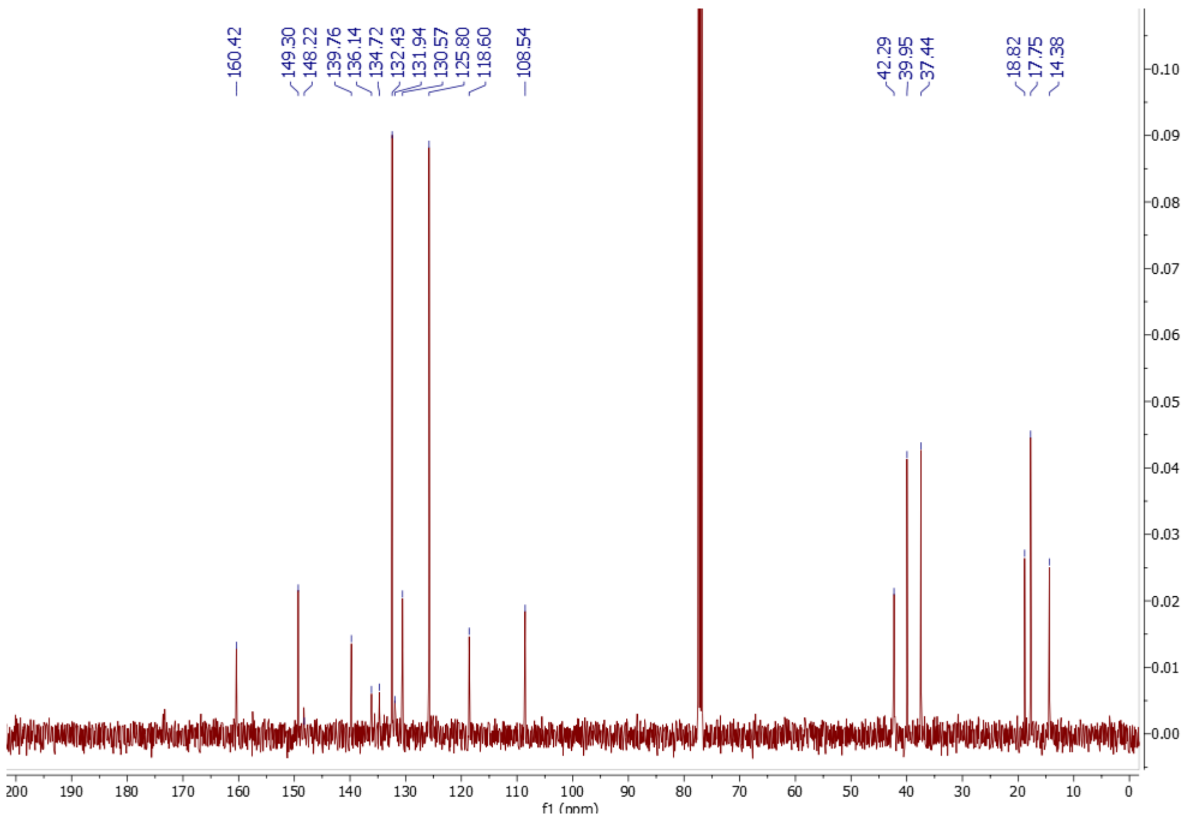

*Supplementary Figure 55. ¹³C NMR of N-(4-cyanophenyl)-N'-(3-(dimethylamino)-2,4,6-trimethylphenyl)-N,N'-dimethylurea (**1v**) (101 MHz, 20 °C, CDCl$_3$)*

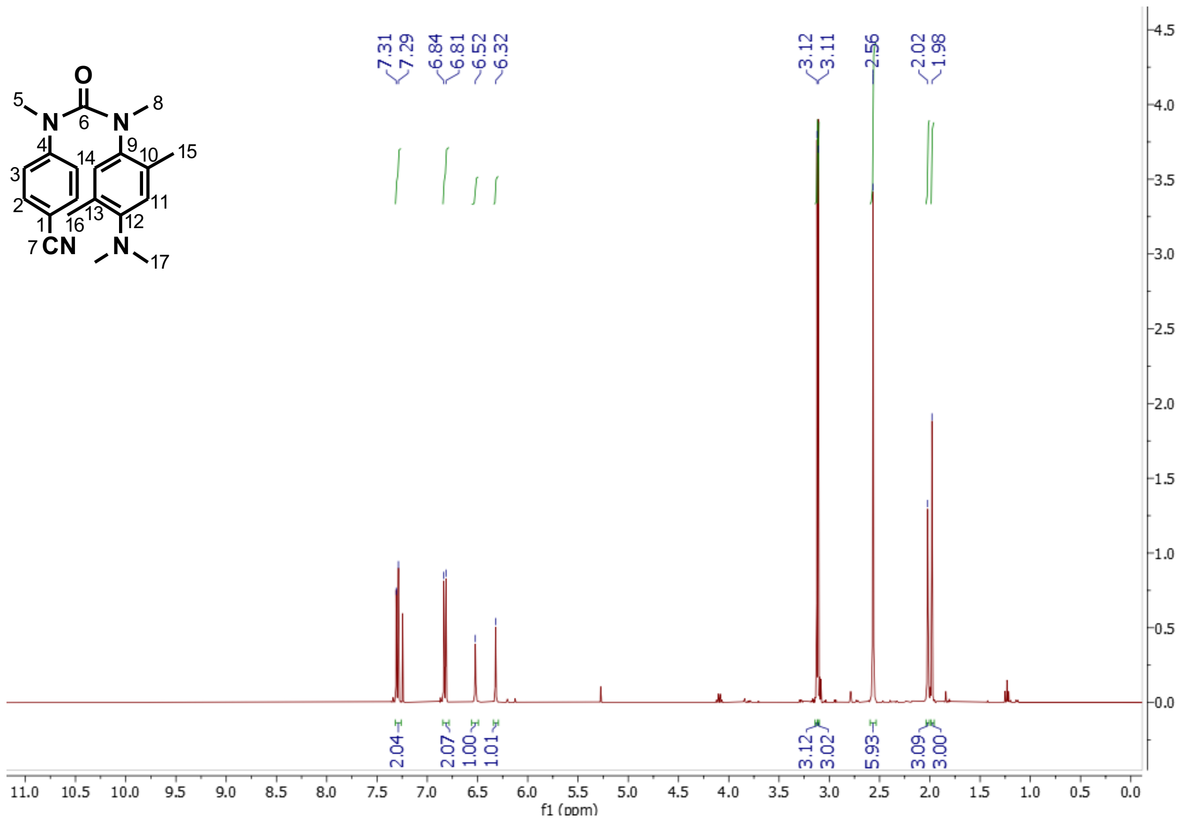

*Supplementary Figure 56. ¹H NMR of N-(4-cyanophenyl)-N'-(4-(dimethylamino)-2,5-dimethylphenyl)-N,N'-dimethylurea (**1w**) (400 MHz, 20 °C, $CDCl_3$)*

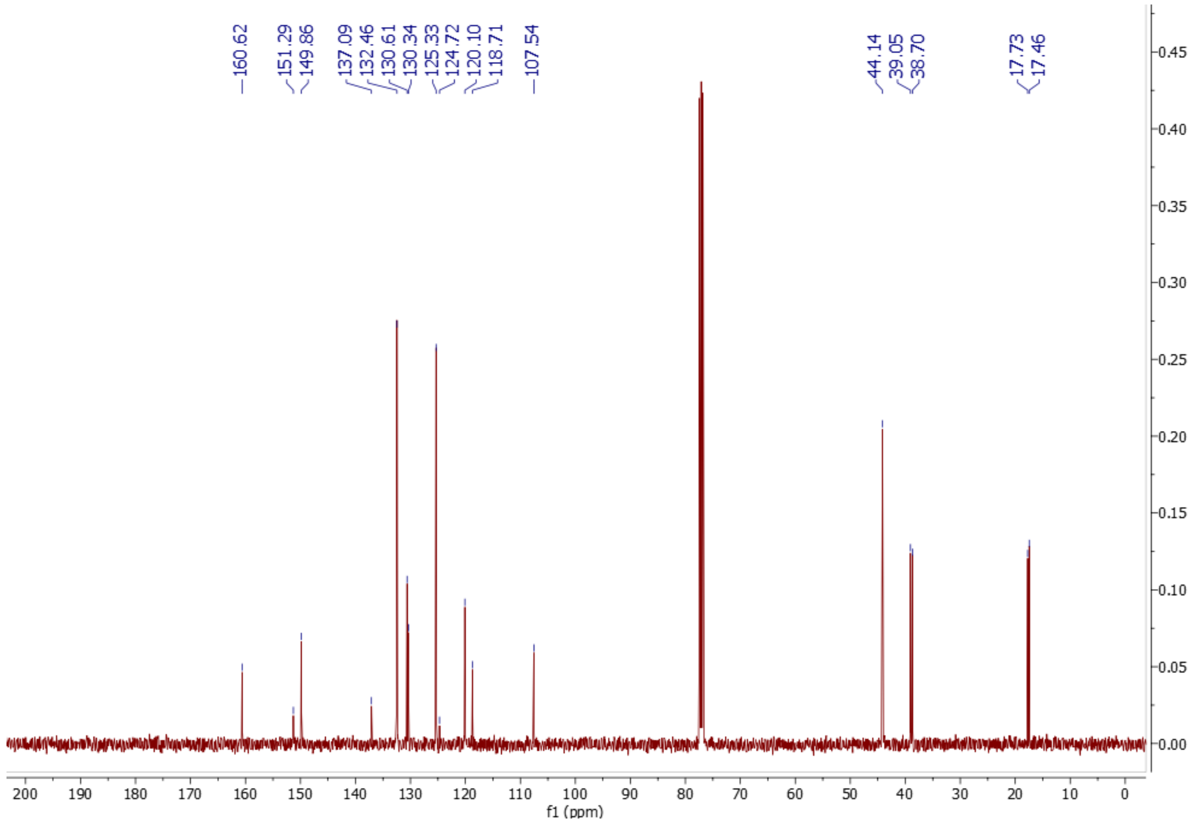

*Supplementary Figure 57. ¹³C NMR of N-(4-cyanophenyl)-N'-(4-(dimethylamino)-2,5-dimethylphenyl)-N,N'-dimethylurea (**1w**) (101 MHz, 20 °C, $CDCl_3$)*

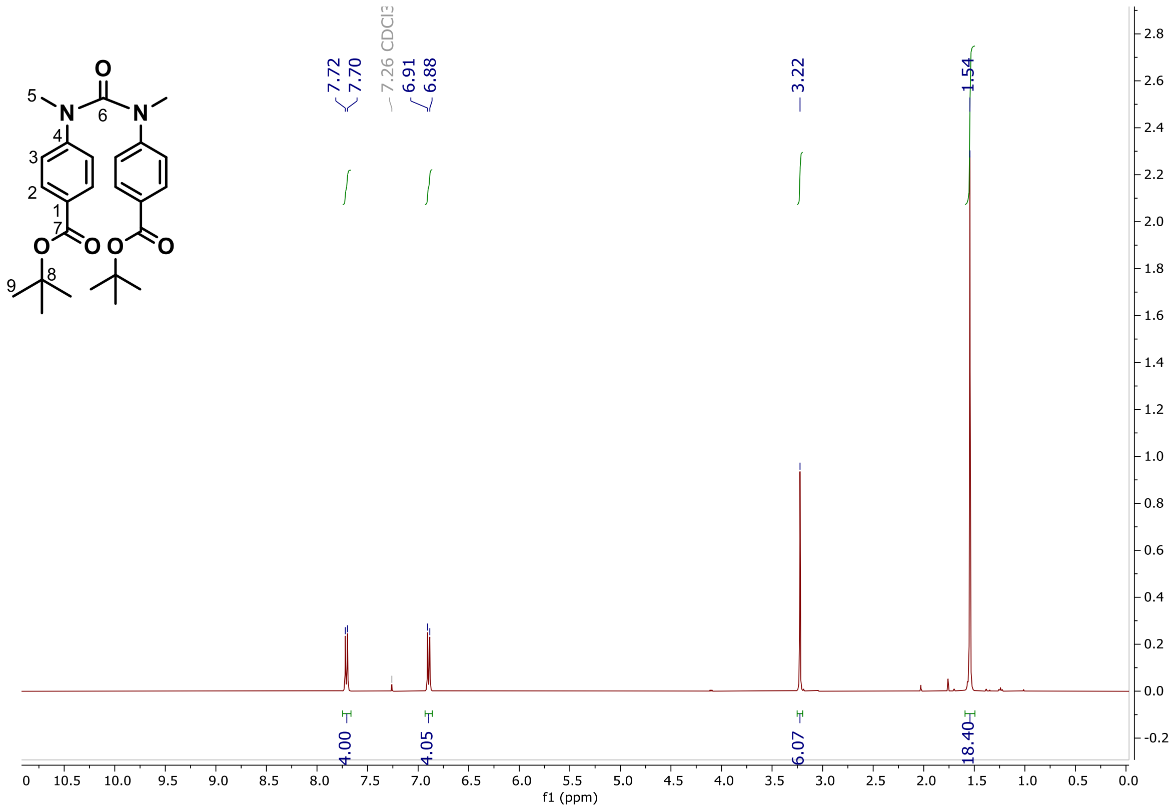

Supplementary Figure 58. [1]*H NMR of di-tert-butyl 4,4'-(carbonylbis(methylazanediyl))dibenzoate (**1x**) (400 MHz, 20 °C, CDCl*$_3$*)*

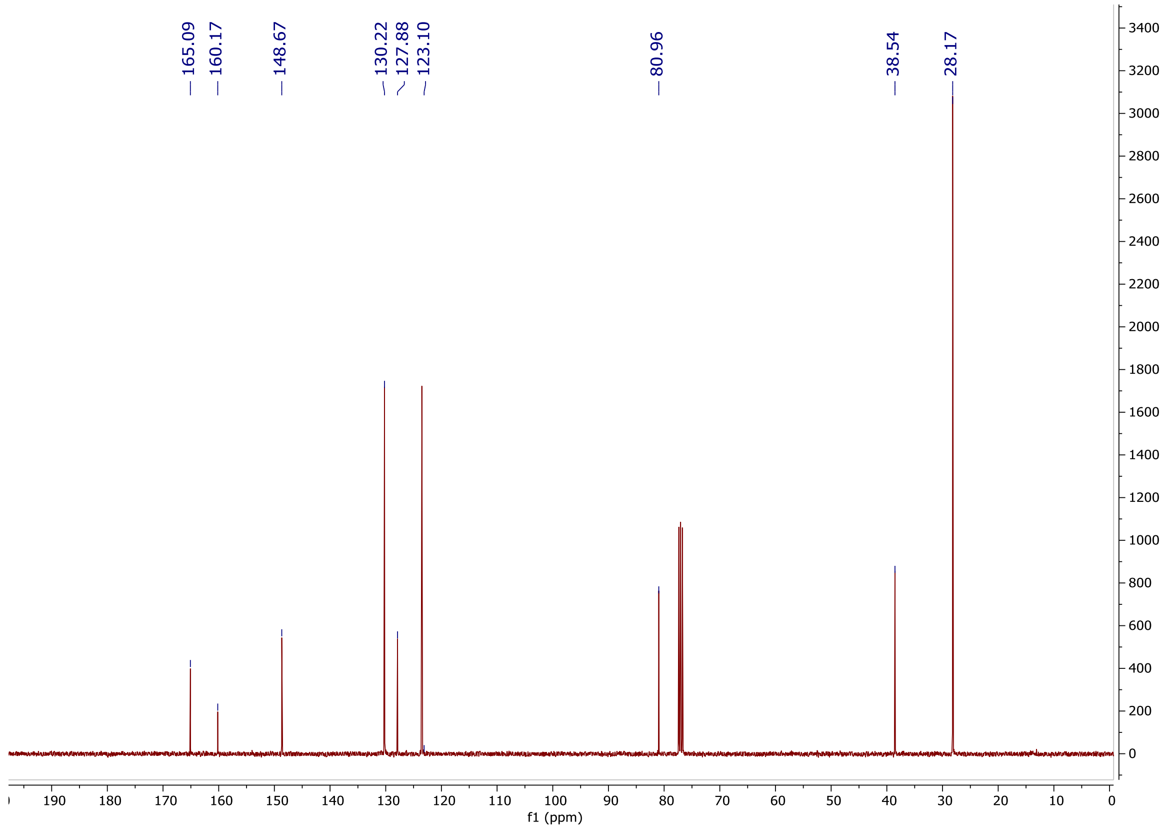

Supplementary Figure 59. [13]*C NMR of di-tert-butyl 4,4'-(carbonylbis(methylazanediyl))dibenzoate (**1x**) (101 MHz, 20 °C, CDCl*$_3$*)*

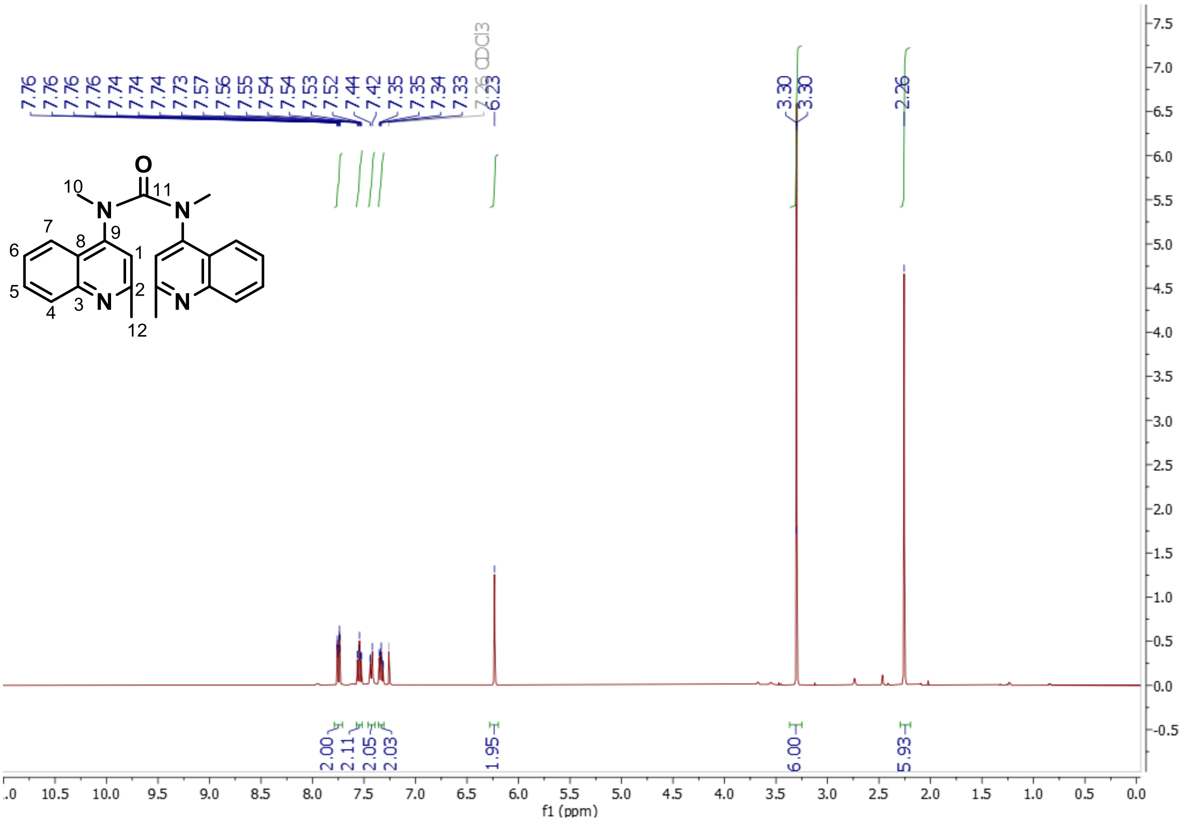

Supplementary Figure 60. ^{1}H NMR of N,N'-dimethyl-N,N'-bis(2-methylquinolin-4-yl)urea (**1y**) (400 MHz, 20 °C, $CDCl_3$)

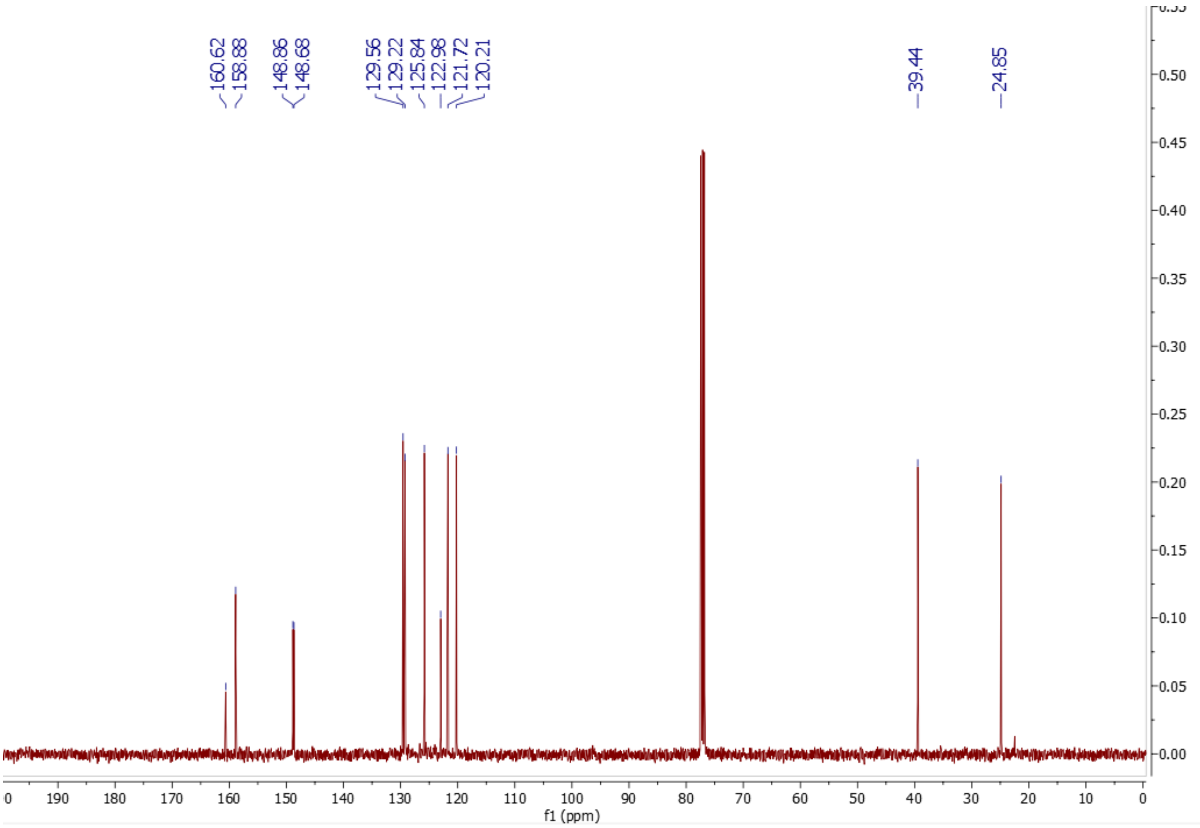

Supplementary Figure 61. ^{13}C NMR of N,N'-dimethyl-N,N'-bis(2-methylquinolin-4-yl)urea (**1y**) (101 MHz, 20 °C, $CDCl_3$)

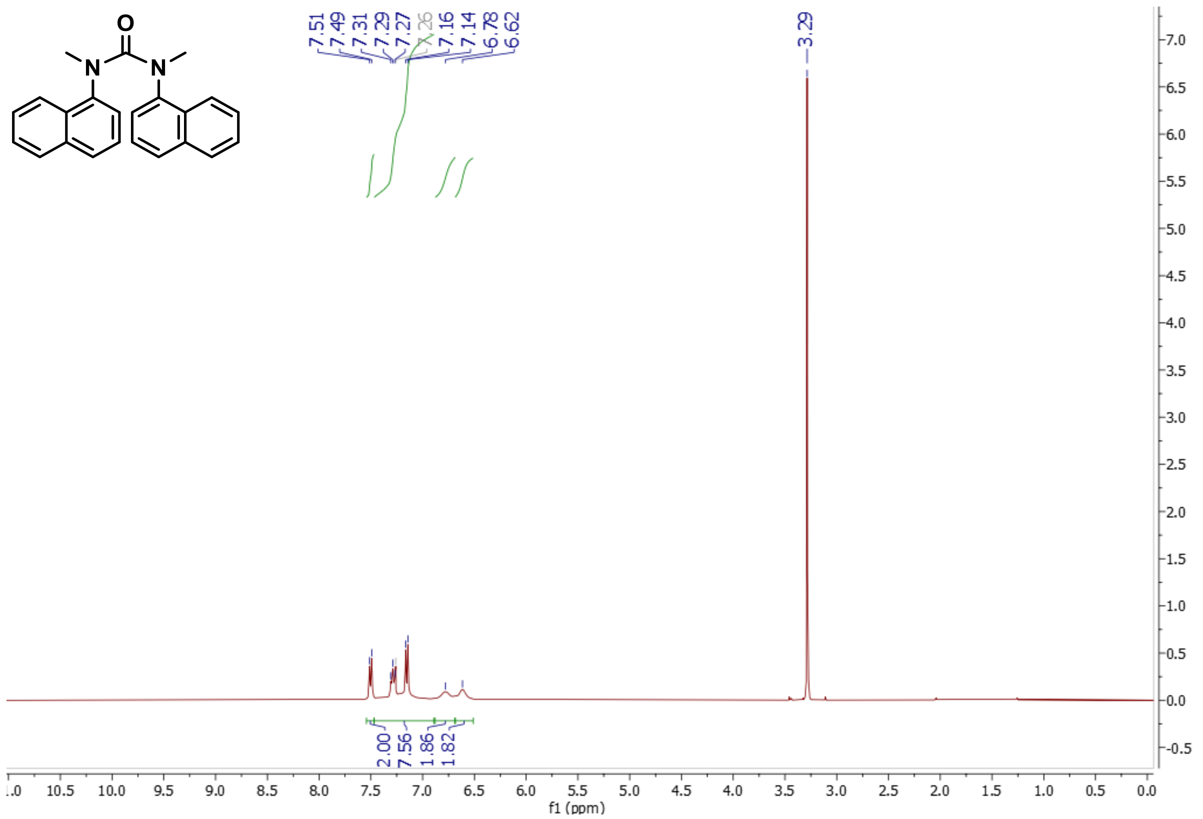

Supplementary Figure 62. ^{1}H *NMR of N,N'-dimethyl-N,N'-di(naphthalen-1-yl)urea (**1z**) (400 MHz, 20 °C,* $CDCl_3$*)*

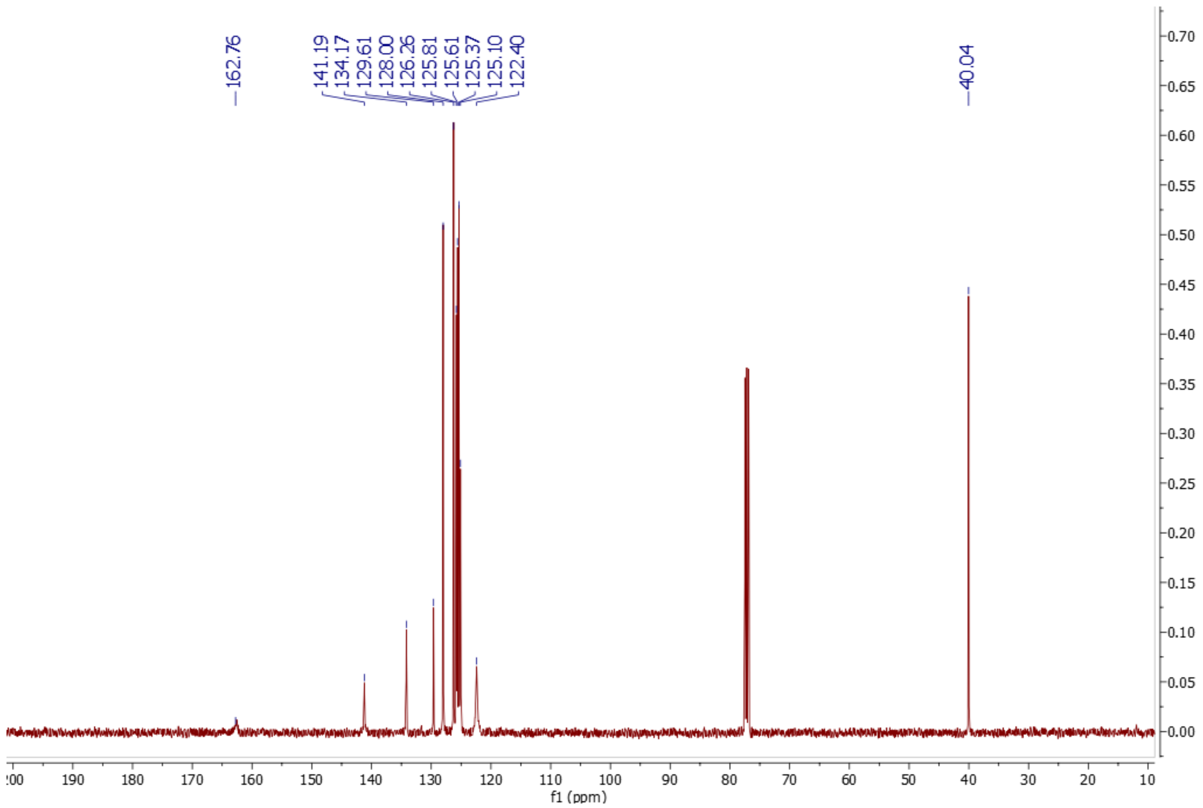

Supplementary Figure 63. ^{13}C *NMR of N,N'-dimethyl-N,N'-di(naphthalen-1-yl)urea (**1z**) (101 MHz, 20 °C,* $CDCl_3$*)*

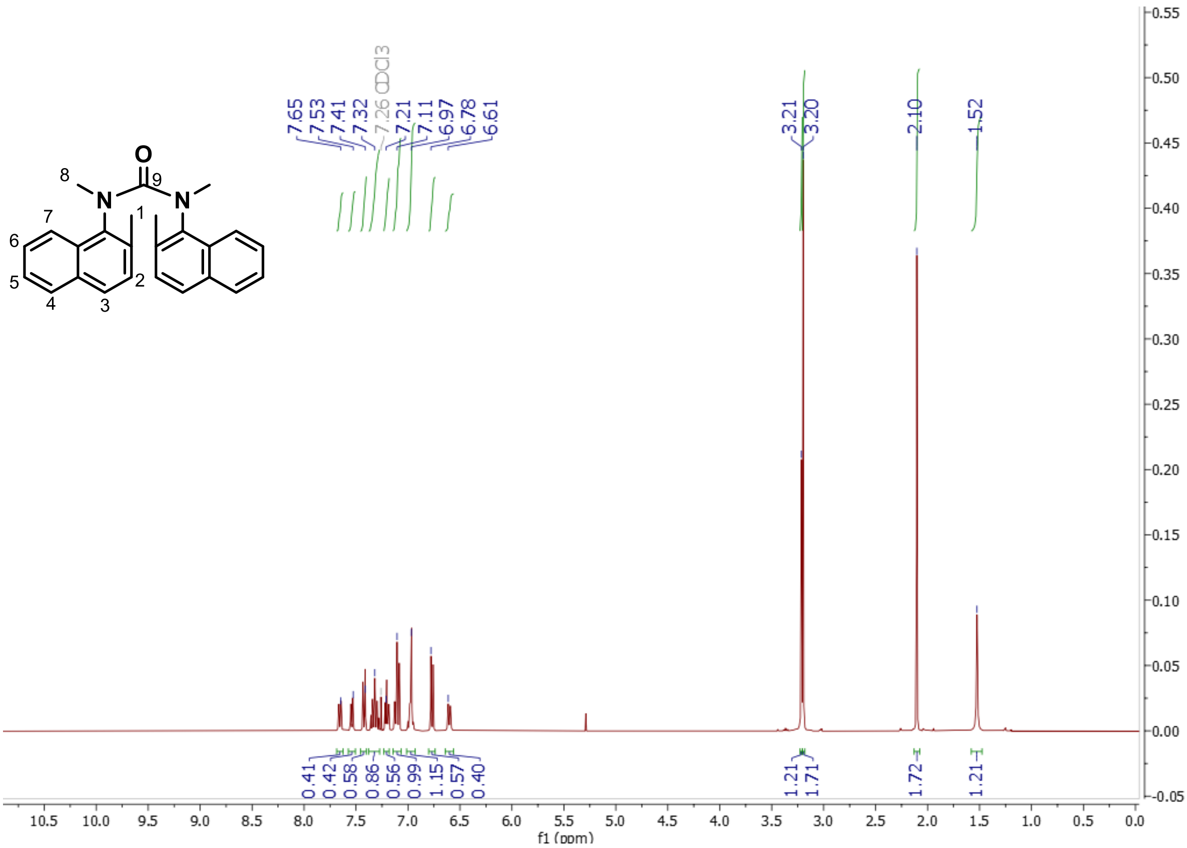

*Supplementary Figure 64. ^{1}H NMR of N,N'-dimethyl-N,N'-bis(2-methylnaphthalen-1-yl)urea (**1aa**) (400 MHz, 20 °C, $CDCl_3$)*

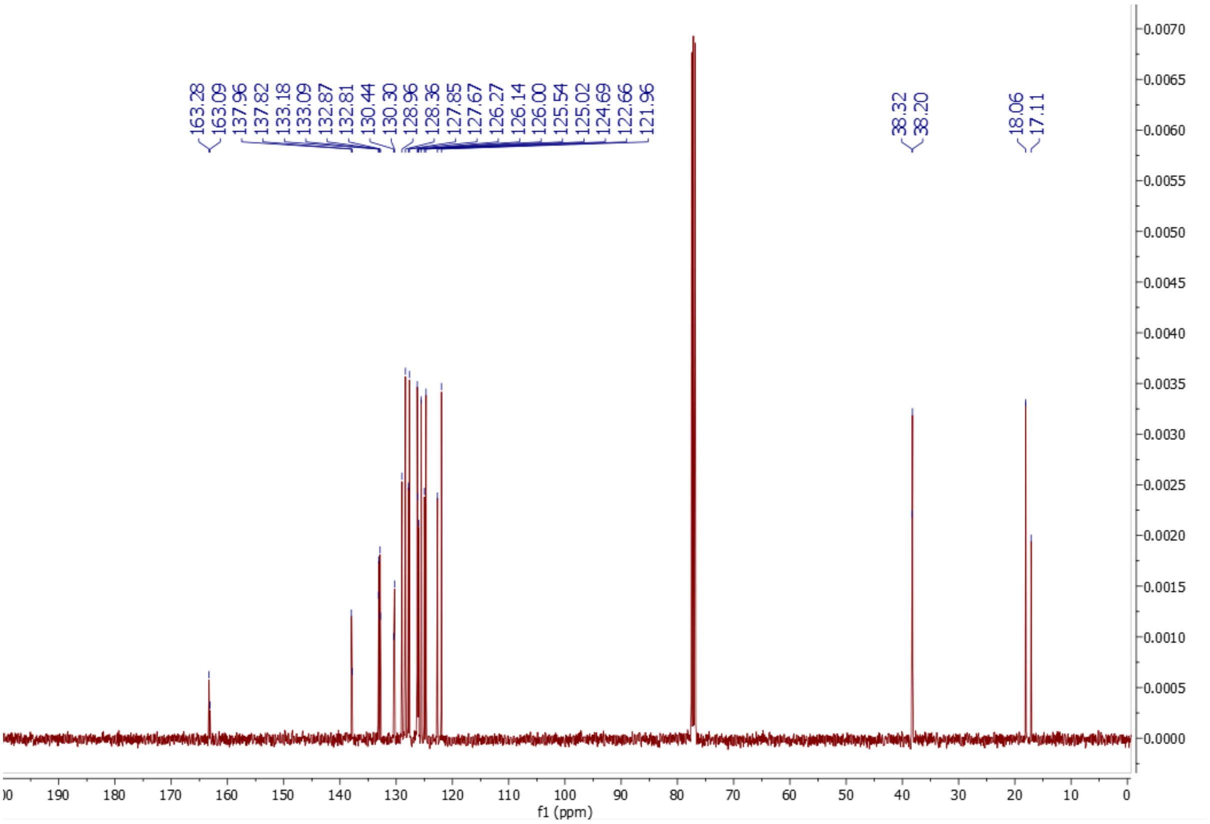

*Supplementary Figure 65. ^{13}C NMR of N,N'-dimethyl-N,N'-bis(2-methylnaphthalen-1-yl)urea (**1aa**) (101 MHz, 20 °C, $CDCl_3$)*

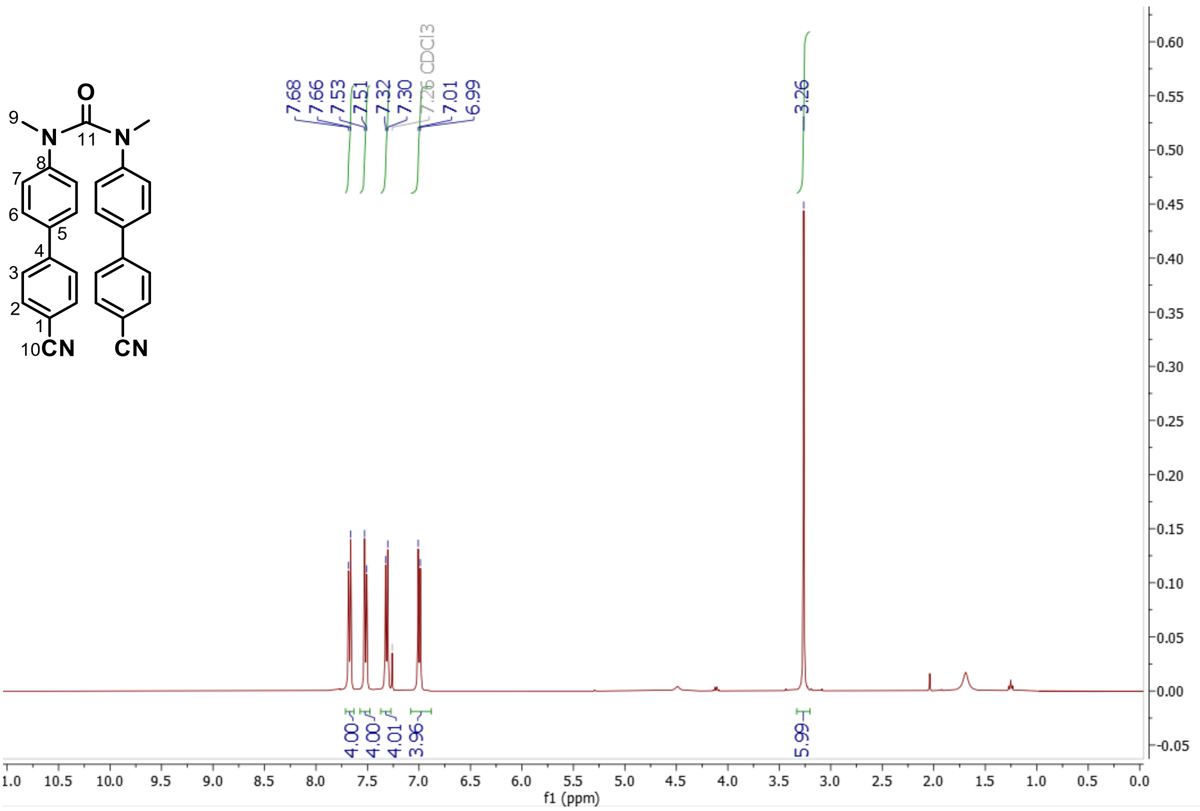

*Supplementary Figure 66. ^{1}H NMR of N,N'-bis(4'-cyano-[1,1'-biphenyl]-4-yl)-N,N'-dimethylurea (**1ab**) (400 MHz, 20 °C, $CDCl_3$)*

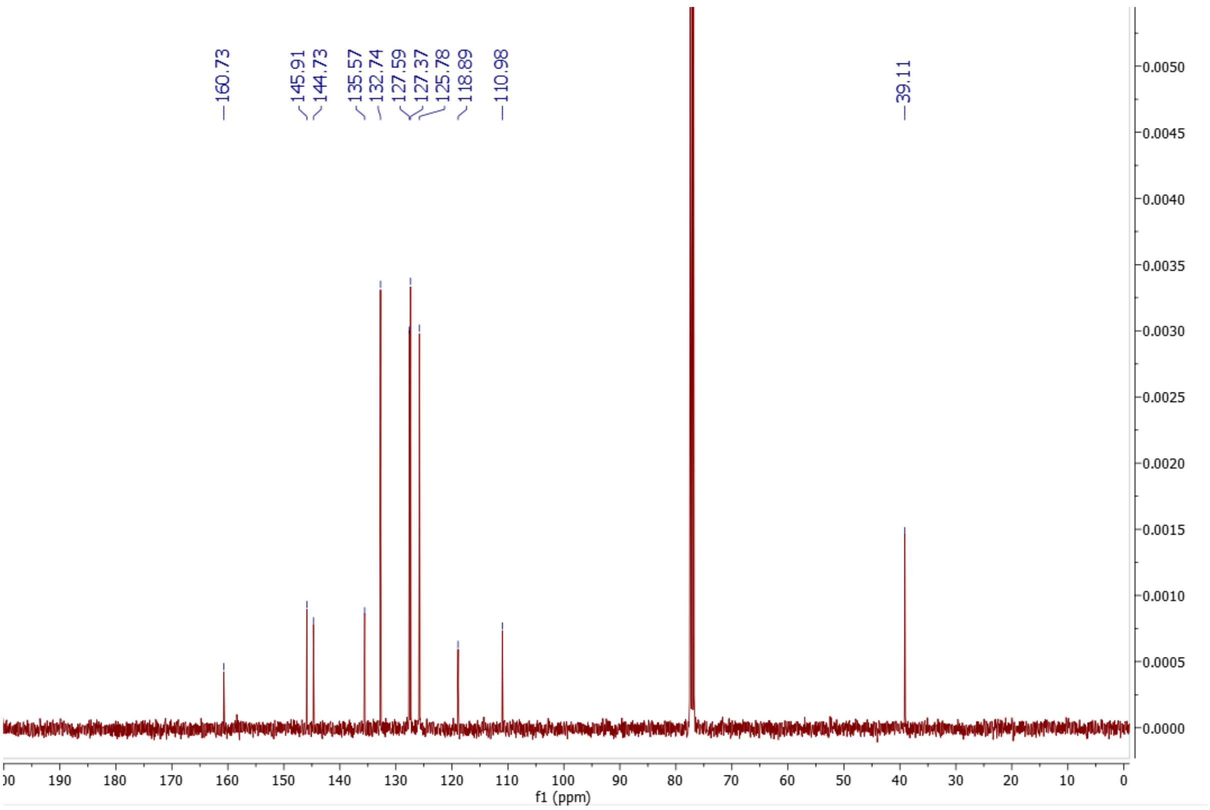

*Supplementary Figure 67. ^{13}C NMR of N,N'-bis(4'-cyano-[1,1'-biphenyl]-4-yl)-N,N'-dimethylurea (**1ab**) (101 MHz, 20 °C, $CDCl_3$)*

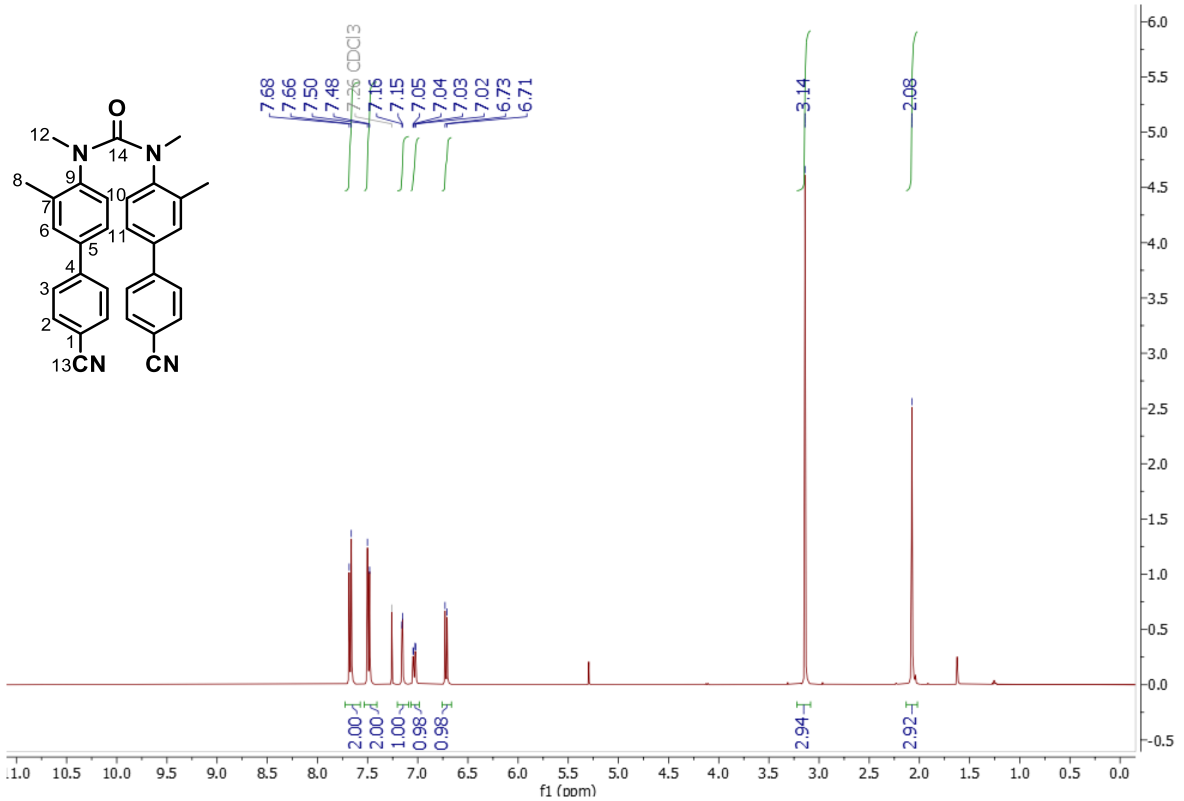

Supplementary Figure 68. ^{1}H NMR of N,N'-bis(4'-cyano-3-methyl-[1,1'-biphenyl]-4-yl)-N,N'-dimethylurea (**1ac**) (400 MHz, 20 °C, $CDCl_3$)

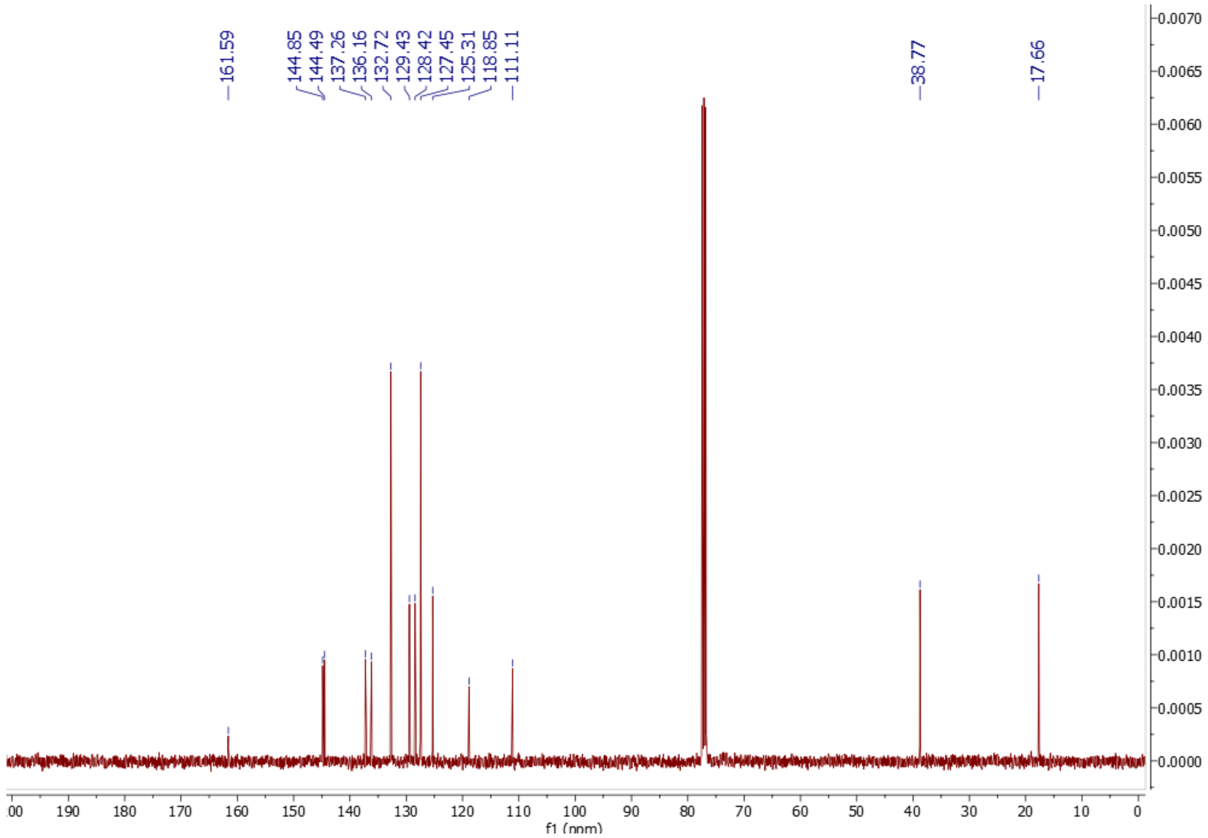

Supplementary Figure 69. ^{13}C NMR of N,N'-bis(4'-cyano-3-methyl-[1,1'-biphenyl]-4-yl)-N,N'-dimethylurea (**1ac**) (101 MHz, 20 °C, $CDCl_3$)

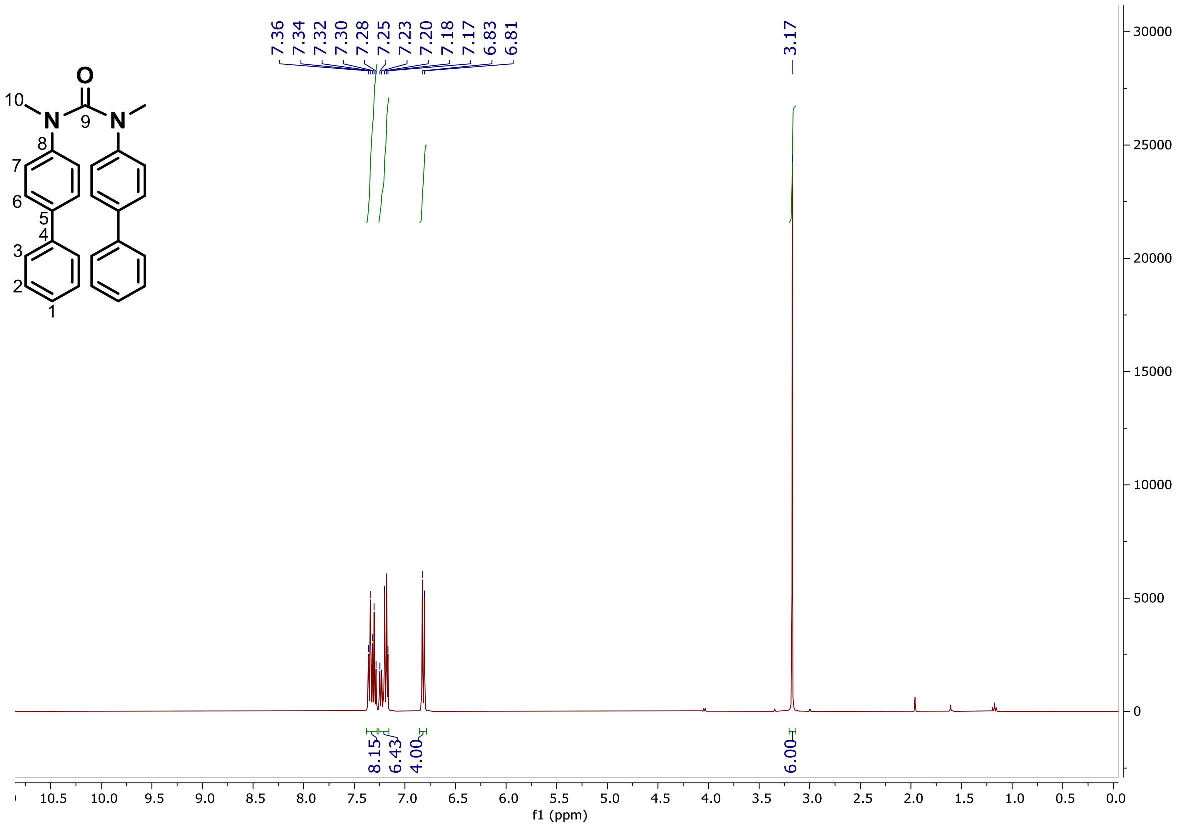

*Supplementary Figure 70. ¹H NMR of N,N'-di([1,1'-biphenyl]-4-yl)-N,N'-dimethylurea (**1ad**) (400 MHz, 20 °C, CDCl₃)*

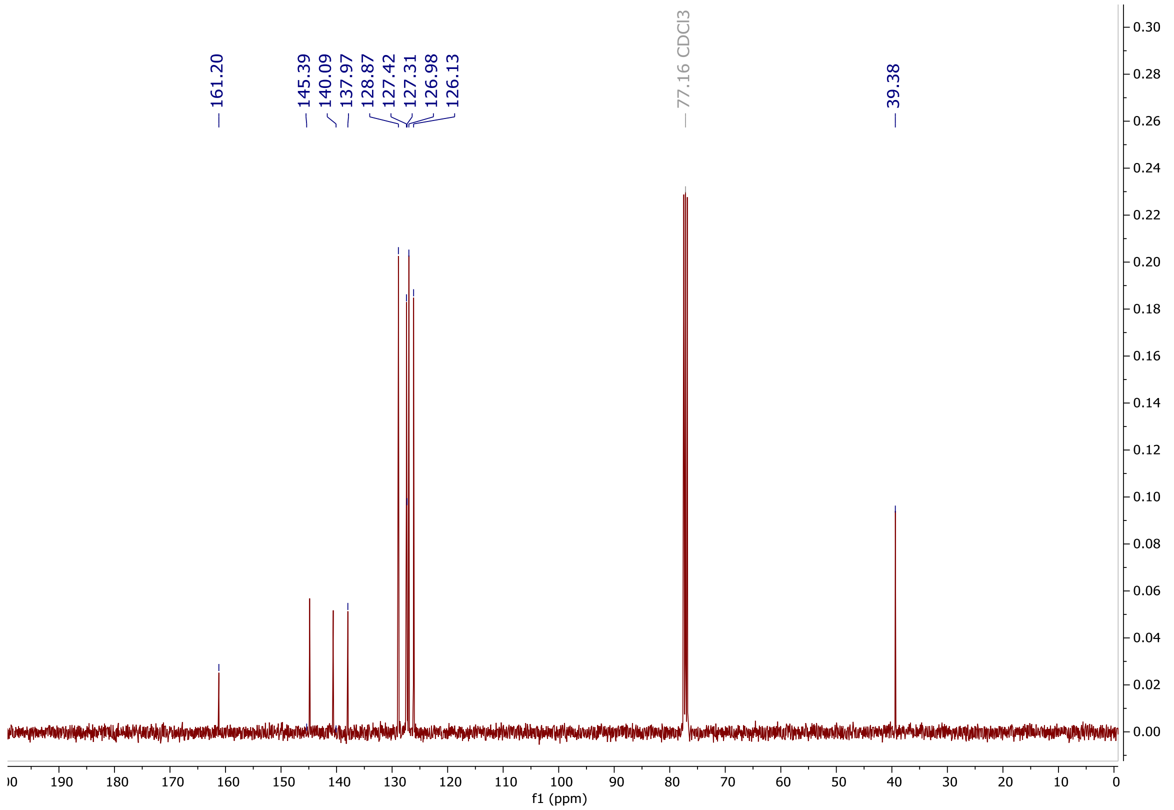

*Supplementary Figure 71. ¹³C NMR of N,N'-di([1,1'-biphenyl]-4-yl)-N,N'-dimethylurea (**1ad**) (101 MHz, 20 °C, CDCl₃)*

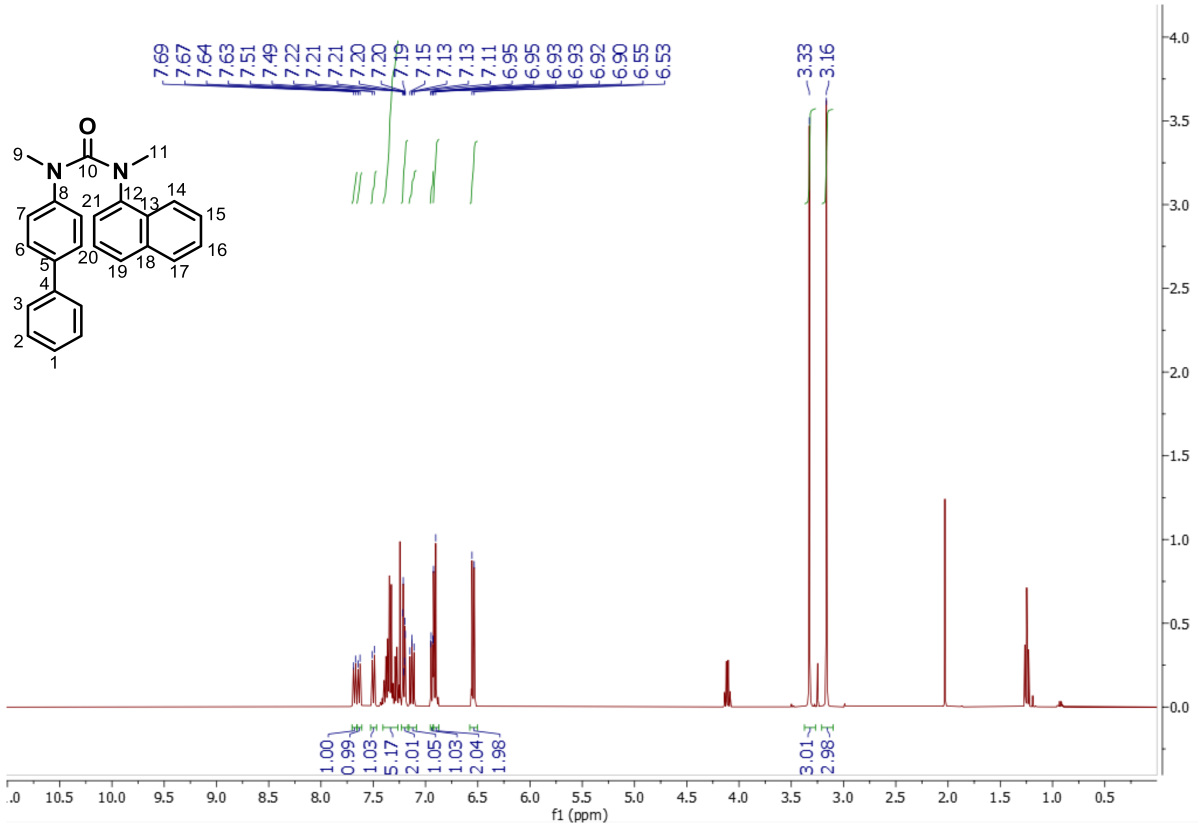

*Supplementary Figure 72. ^{1}H NMR of N-([1,1'-biphenyl]-4-yl)-N,N'-dimethyl-N'-(naphthalen-1-yl)urea (**1ae**) (400 MHz, 20 °C, $CDCl_3$)*

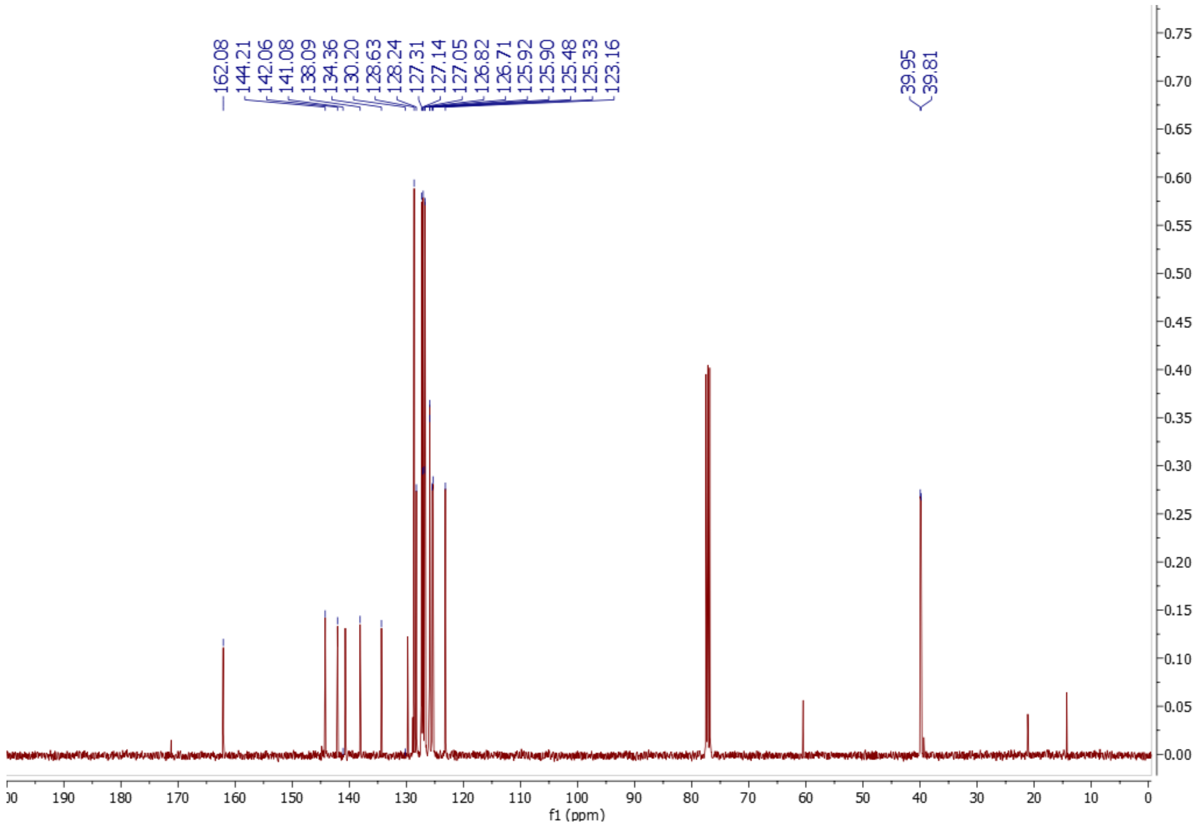

*Supplementary Figure 73. ^{13}C NMR of N-([1,1'-biphenyl]-4-yl)-N,N'-dimethyl-N'-(naphthalen-1-yl)urea (**1ae**) (101 MHz, 20 °C, $CDCl_3$)*

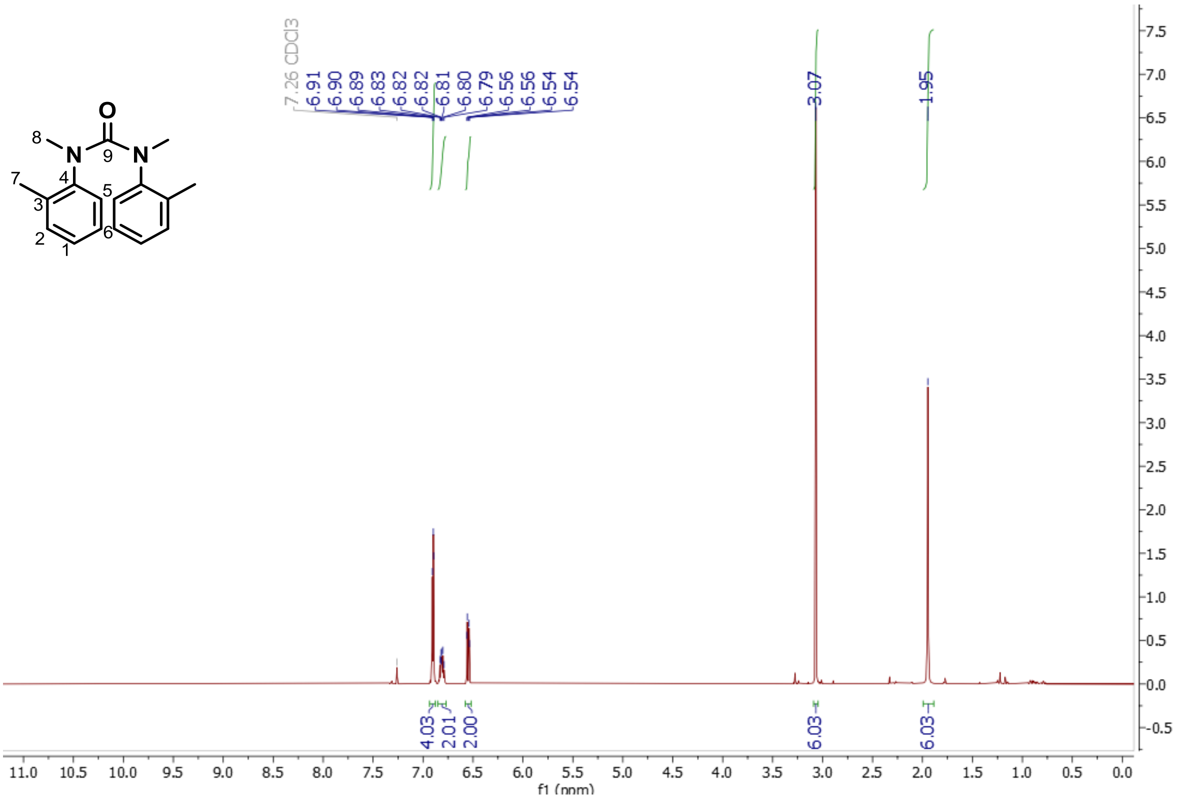

*Supplementary Figure 74. ^{1}H NMR of N,N'-dimethyl-N,N'-di-o-tolylurea (**1af**) (400 MHz, 20 °C, $CDCl_3$)*

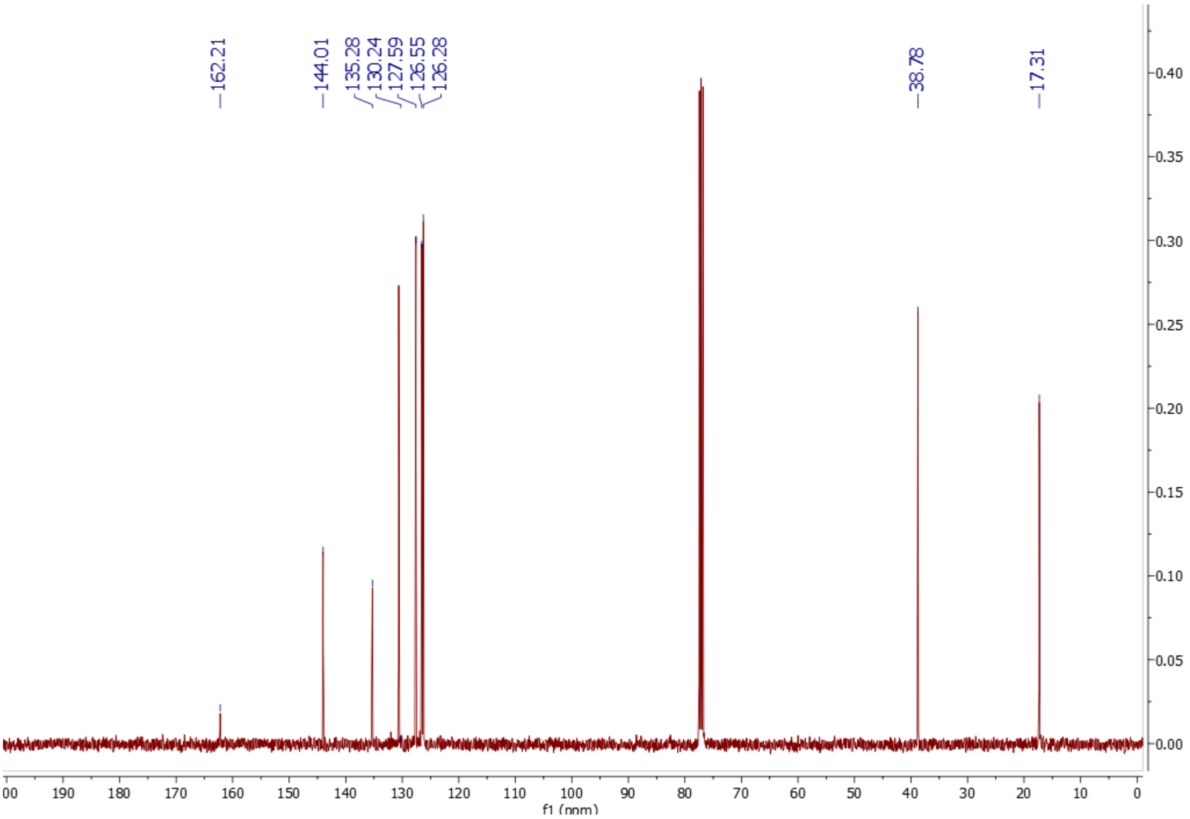

*Supplementary Figure 75. ^{13}C NMR of N,N'-dimethyl-N,N'-di-o-tolylurea (**1af**) (101 MHz, 20 °C, $CDCl_3$)*

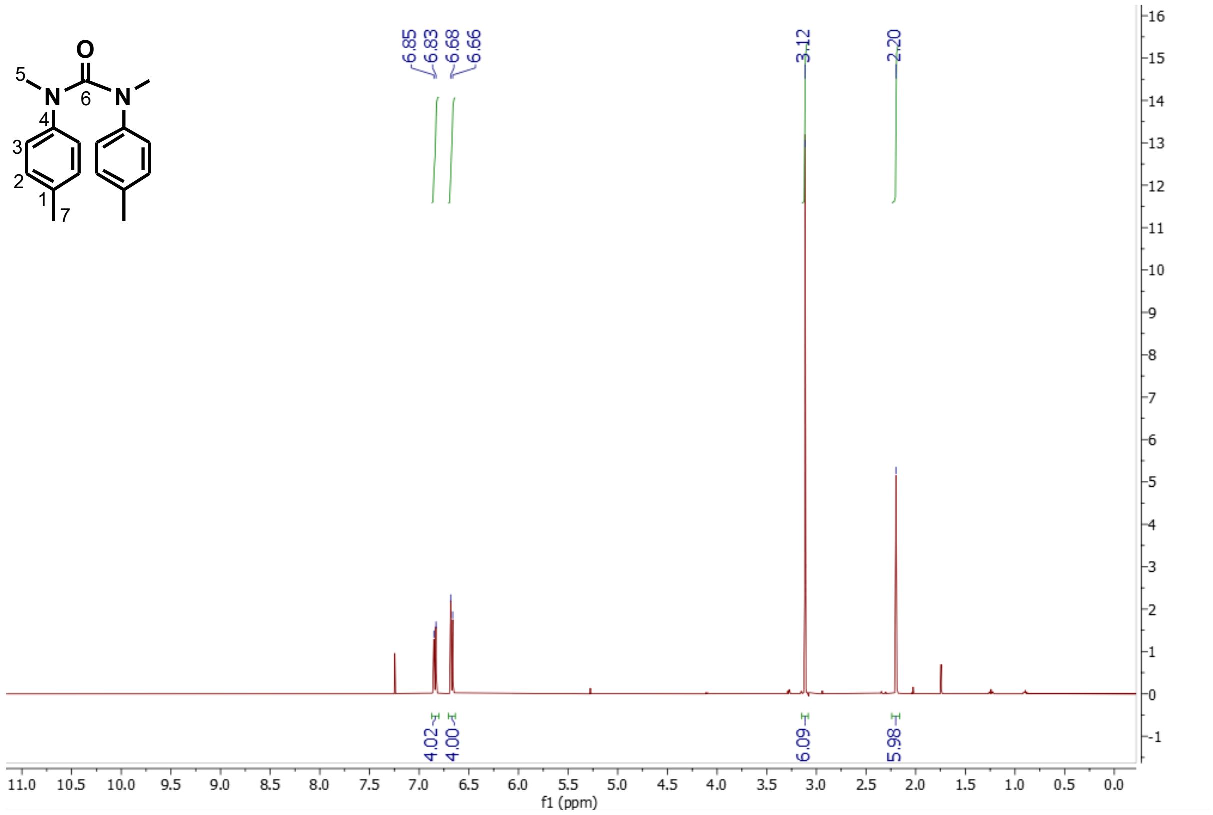

*Supplementary Figure 76. ¹H NMR of N,N'-dimethyl-N,N'-di-p-tolylurea (**1ag**) (400 MHz, 20 °C, $CDCl_3$)*

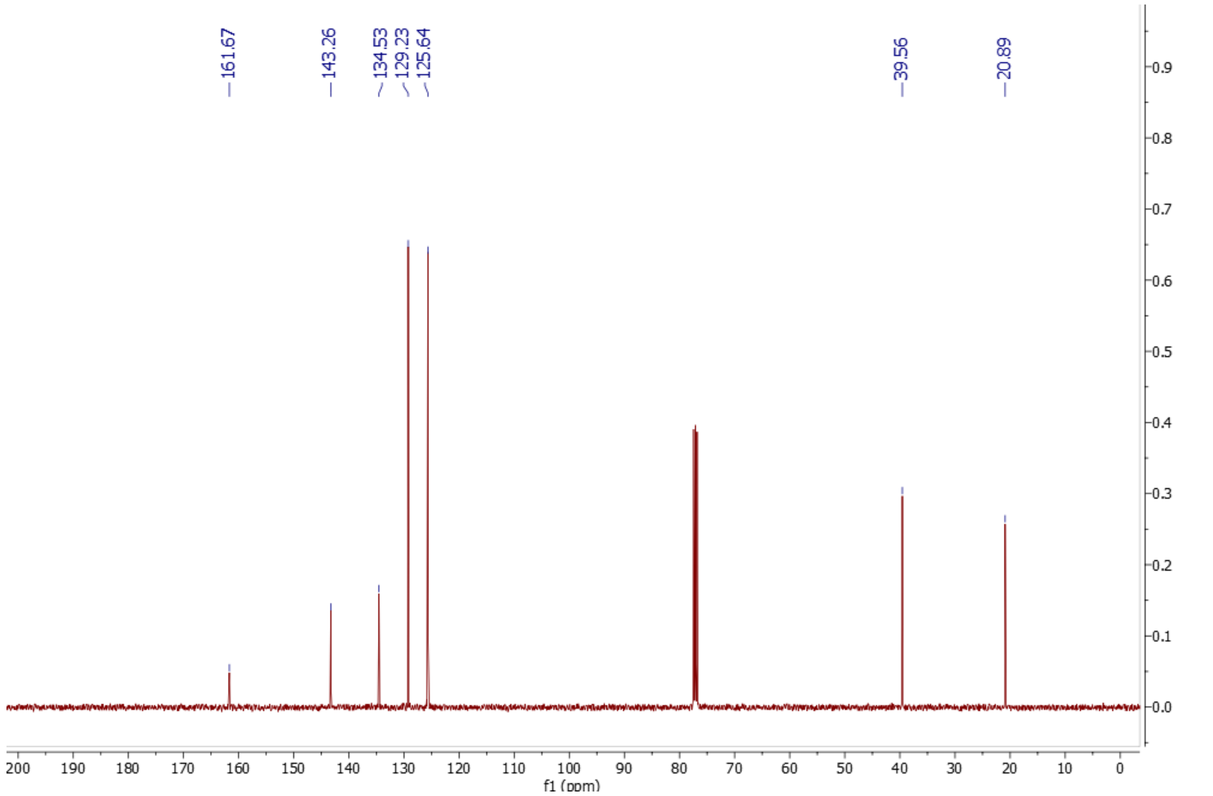

*Supplementary Figure 77. ¹³C NMR of N,N'-dimethyl-N,N'-di-p-tolylurea (**1ag**) (101 MHz, 20 °C, $CDCl_3$)*

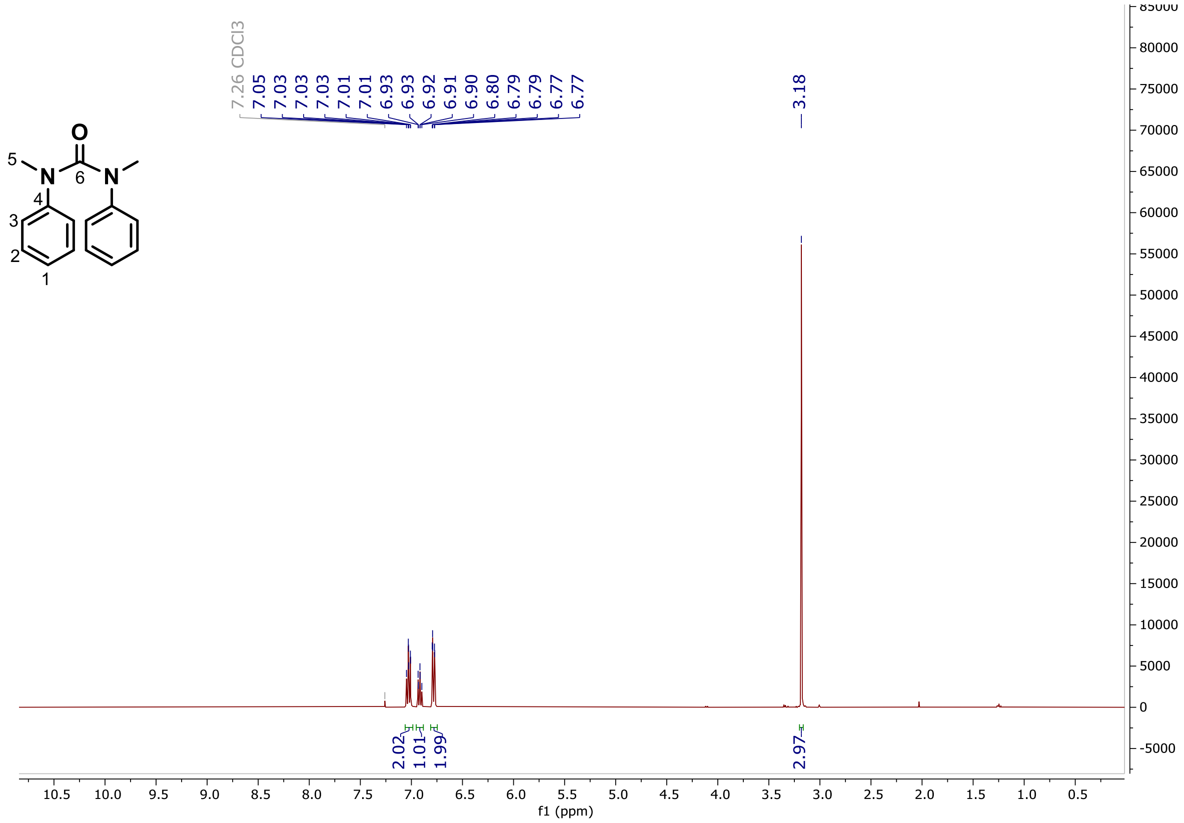

*Supplementary Figure 78. ^{1}H NMR of N,N'-dimethyl-N,N'-diphenylurea (**1ah**) (400 MHz, 20 °C, $CDCl_3$)*

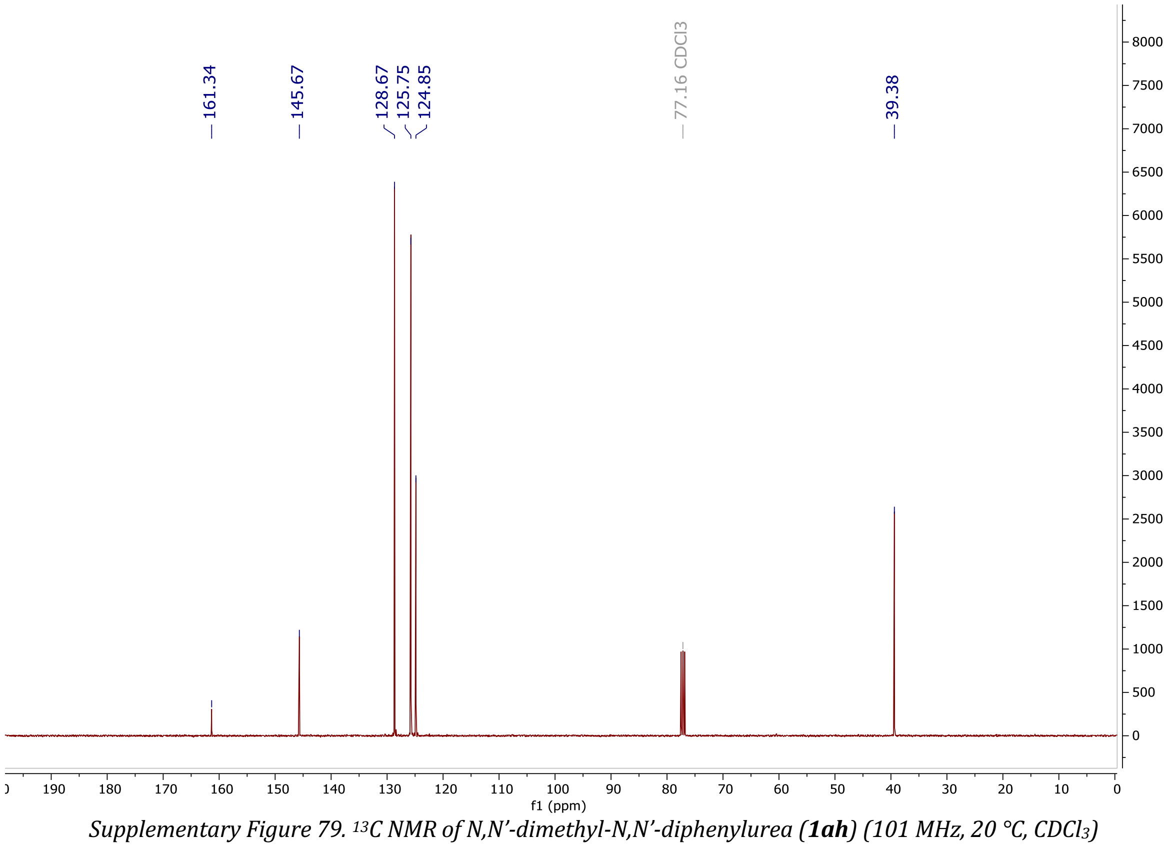

*Supplementary Figure 79. ^{13}C NMR of N,N'-dimethyl-N,N'-diphenylurea (**1ah**) (101 MHz, 20 °C, $CDCl_3$)*

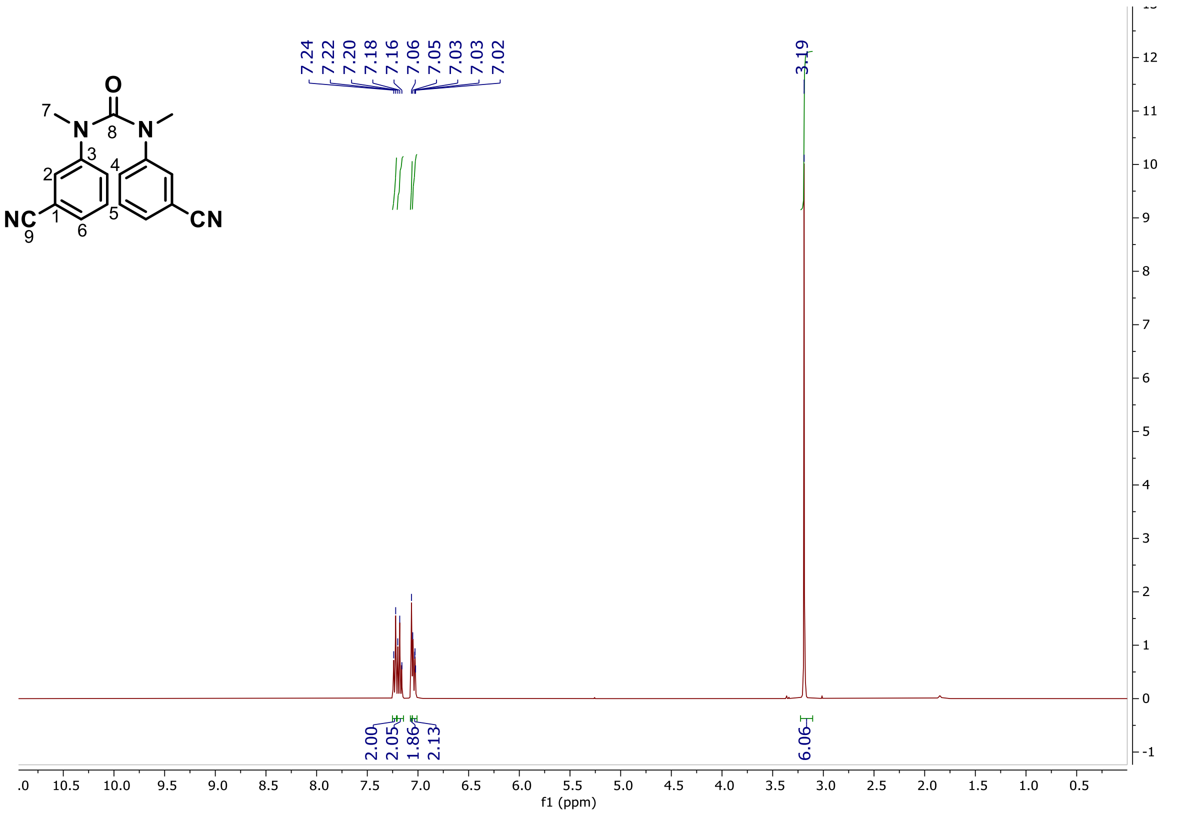

*Supplementary Figure 80. ^{1}H NMR of N,N'-bis(3-cyanophenyl)-N,N'-dimethylurea (**1ai**) (400 MHz, 20 °C, $CDCl_3$)*

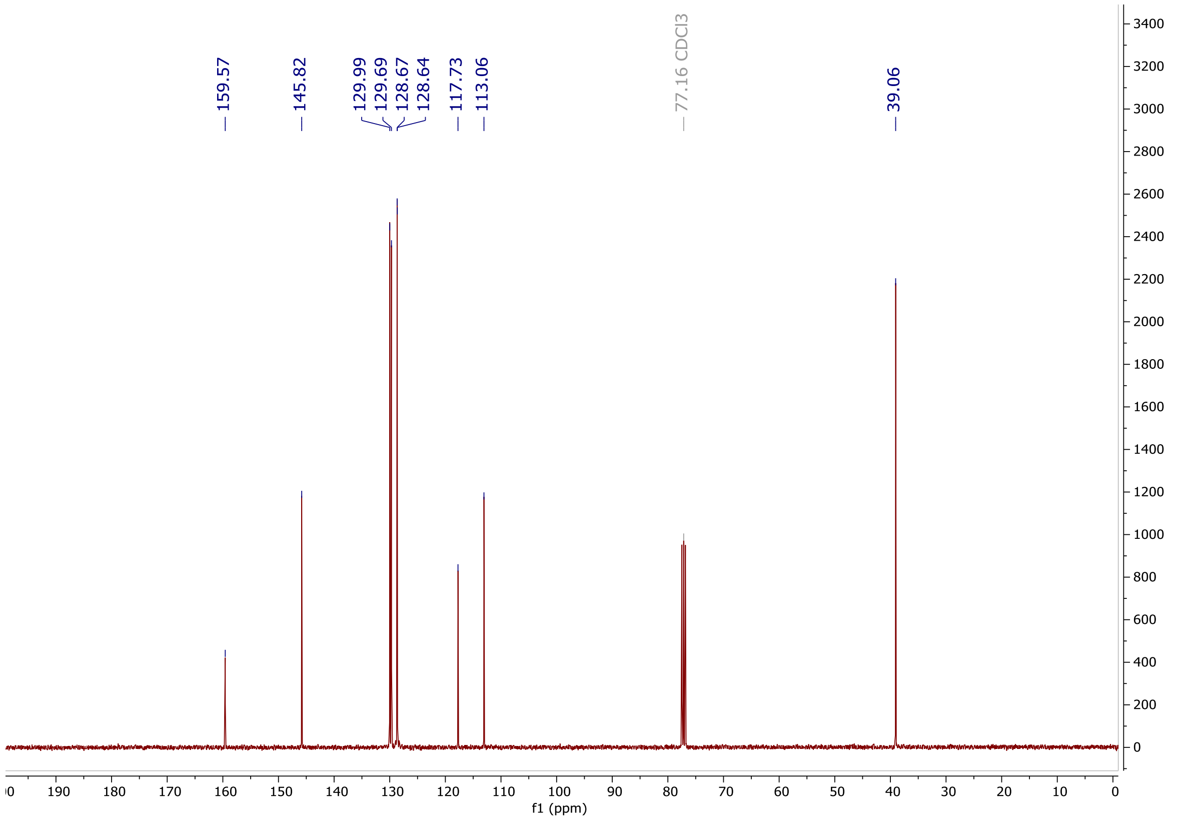

*Supplementary Figure 81. ^{13}C NMR of N,N'-bis(3-cyanophenyl)-N,N'-dimethylurea (**1ai**) (101 MHz, 20 °C, $CDCl_3$)*

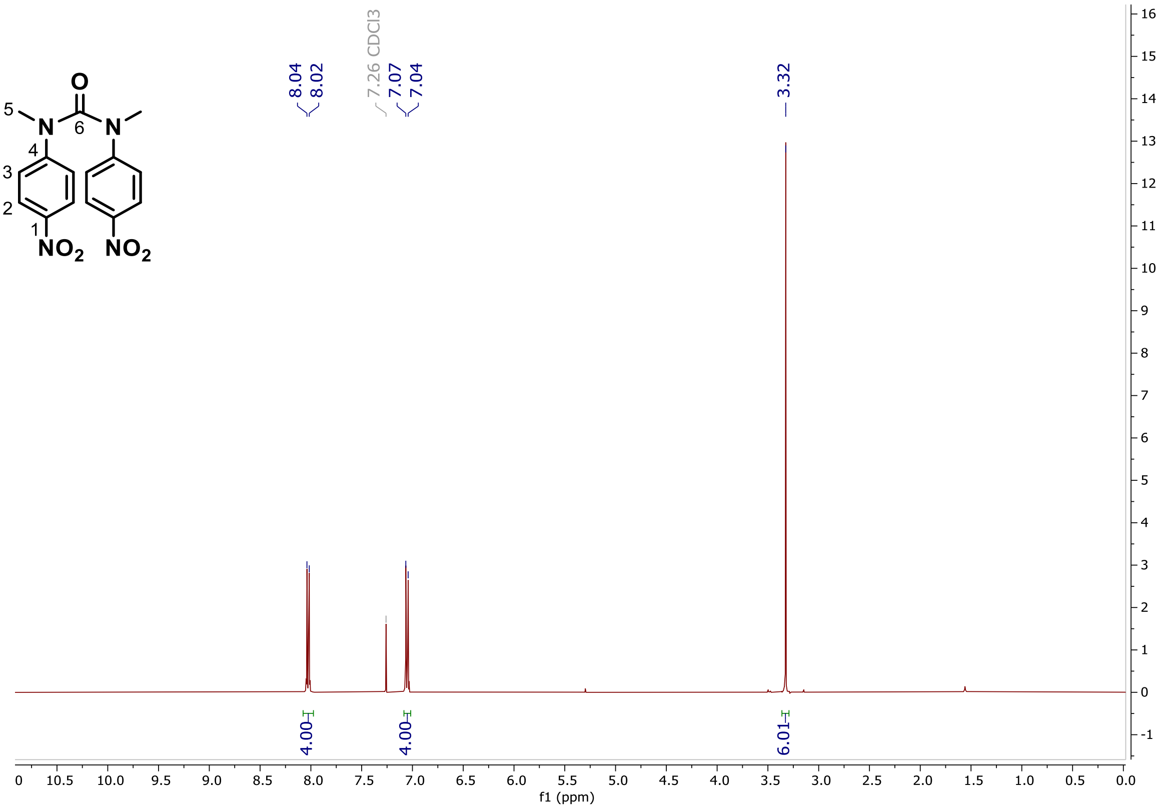

*Supplementary Figure 82. ^{1}H NMR of N,N'-dimethyl-N,N'-bis(4-nitrophenyl)urea (**1aj**) (400 MHz, 20 °C, $CDCl_3$)*

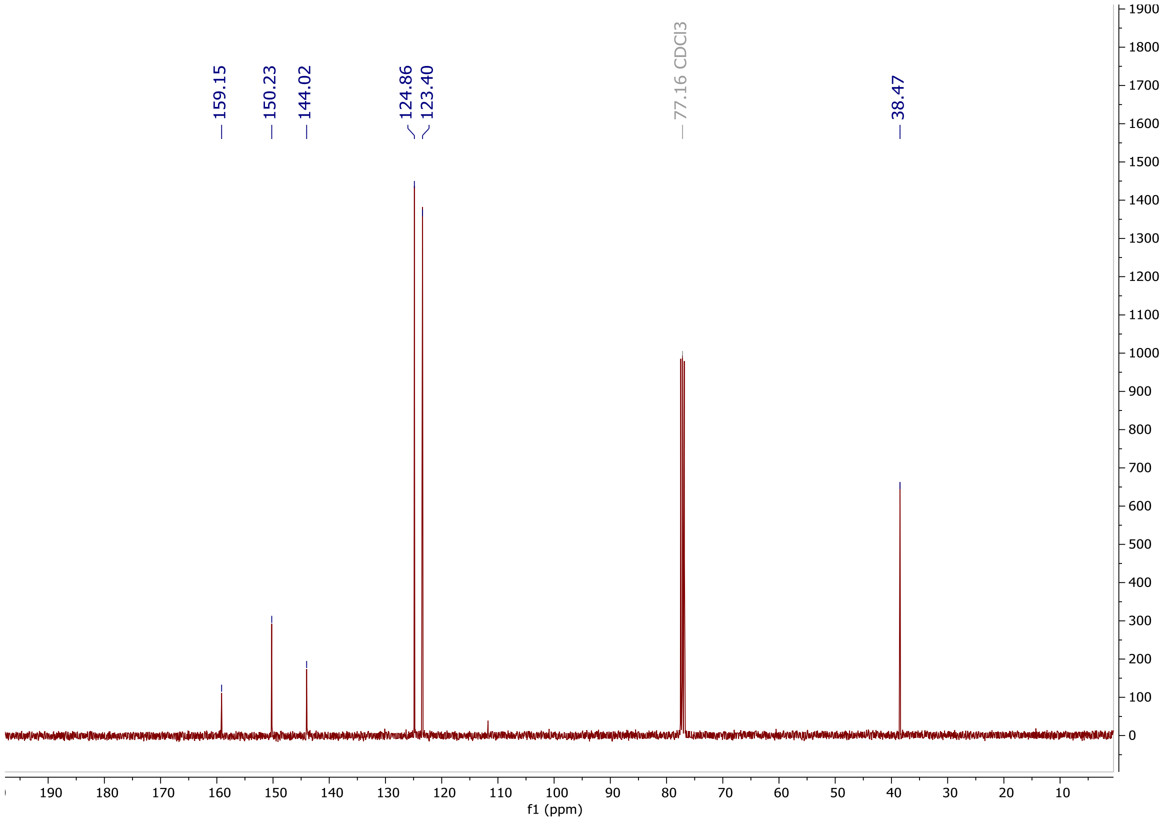

*Supplementary Figure 83. ^{13}C NMR of N,N'-dimethyl-N,N'-bis(4-nitrophenyl)urea (**1aj**) (101 MHz, 20 °C, $CDCl_3$)*

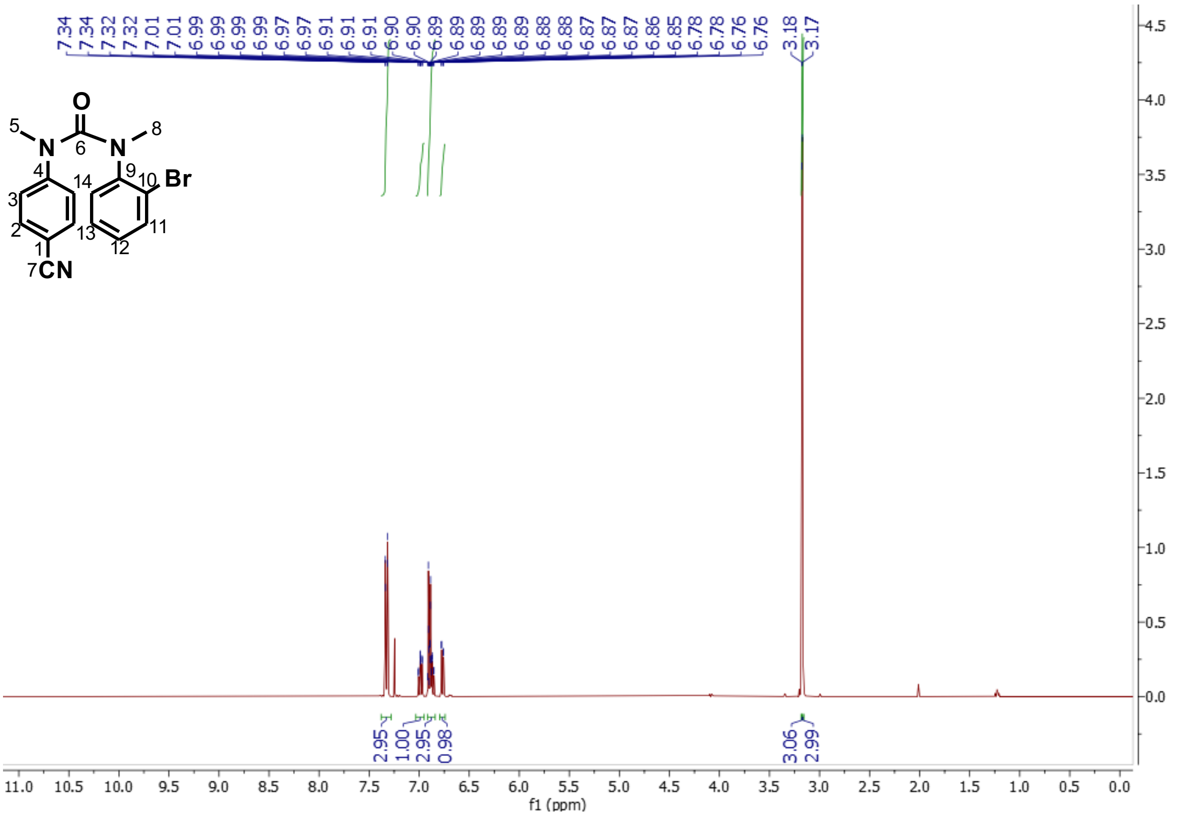

Supplementary Figure 84. ^{1}H NMR of N-(2-bromophenyl)-N'-(4-cyanophenyl)-N,N'-dimethylurea (**1ak**) (400 MHz, 20 °C, $CDCl_3$)

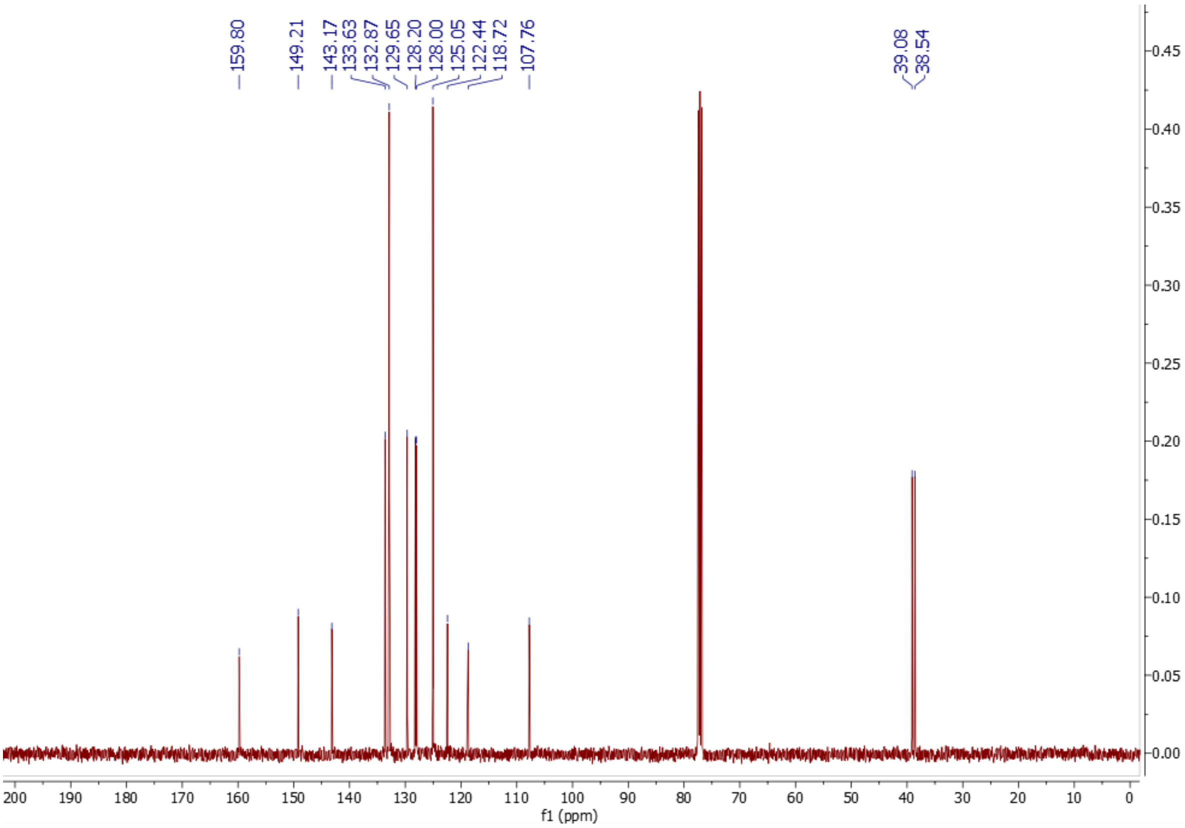

Supplementary Figure 85. ^{13}C NMR of N-(2-bromophenyl)-N'-(4-cyanophenyl)-N,N'-dimethylurea (**1ak**) (101 MHz, 20 °C, $CDCl_3$)

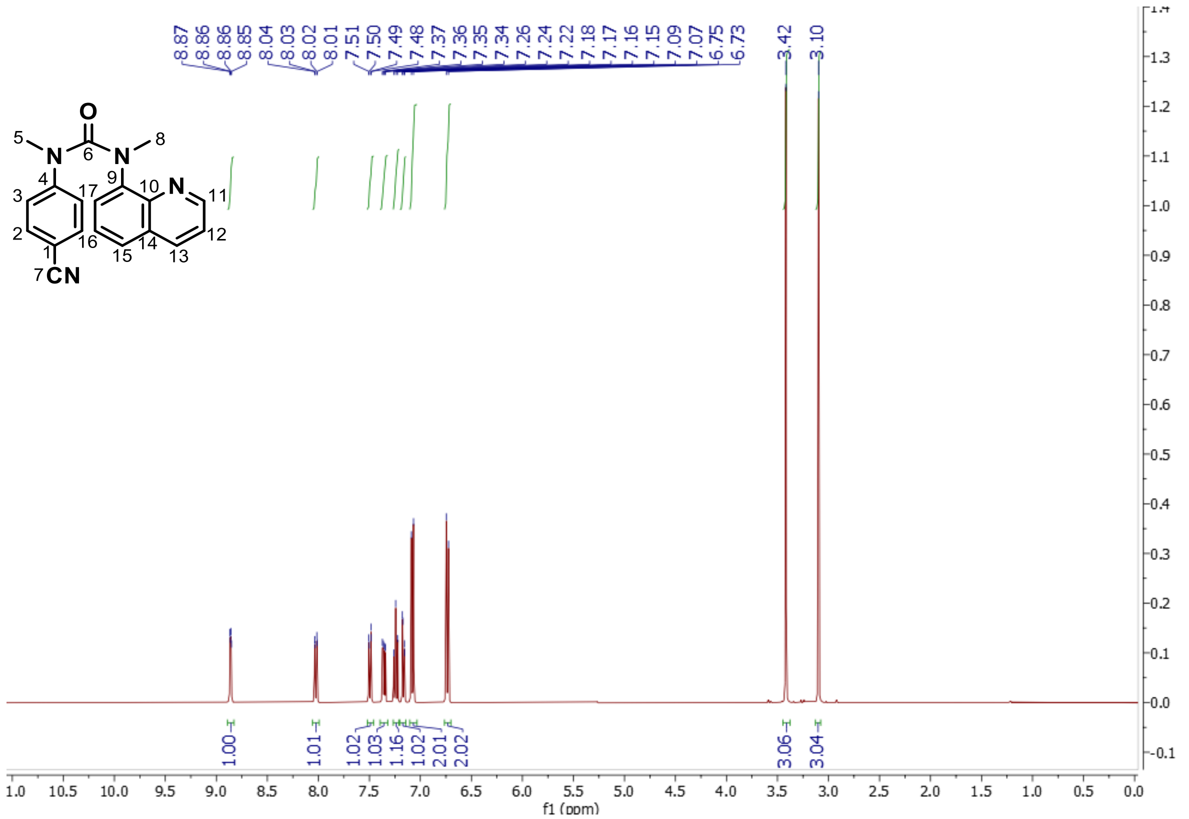

*Supplementary Figure 86. ^{1}H NMR of N-(4-cyanophenyl)-N,N'-dimethyl-N'-(quinolin-8-yl) (**1al**) (400 MHz, 20 °C, $CDCl_3$)*

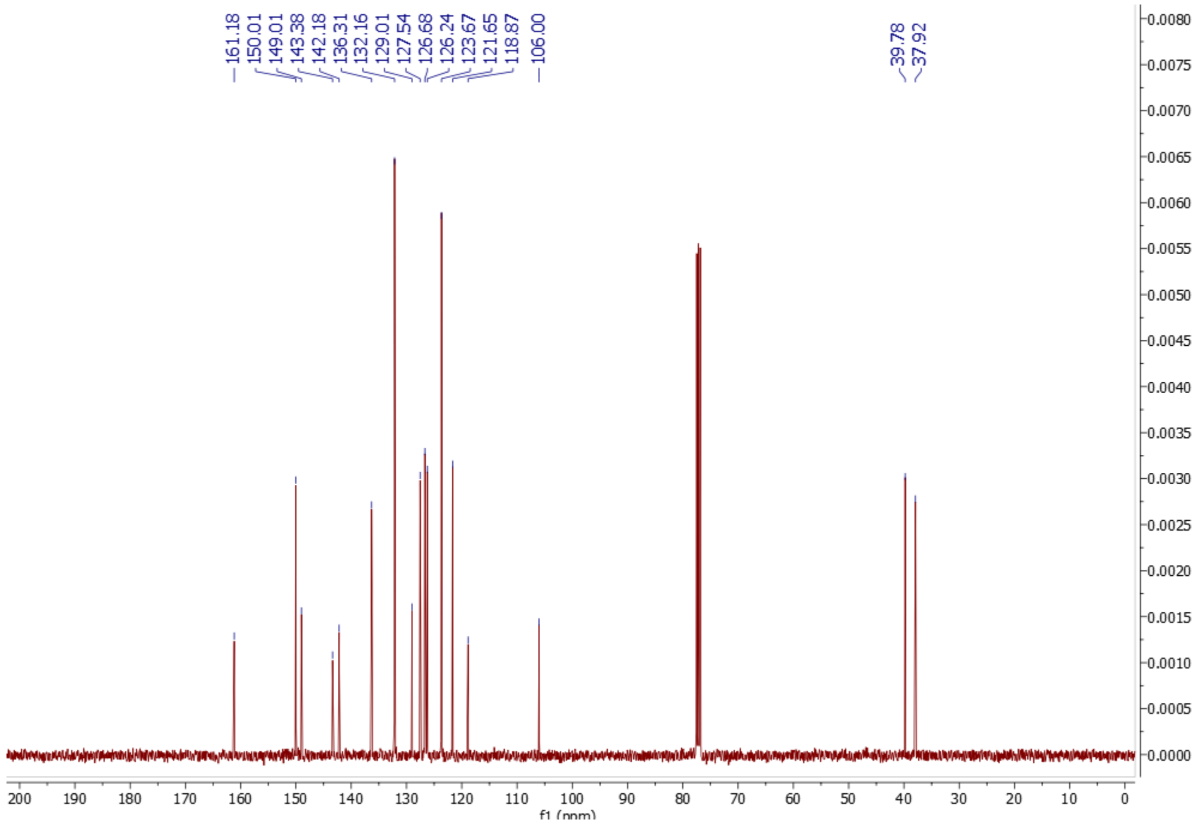

*Supplementary Figure 87. ^{13}C NMR of N-(4-cyanophenyl)-N,N'-dimethyl-N'-(quinolin-8-yl) (**1al**) (101 MHz, 20 °C, $CDCl_3$)*

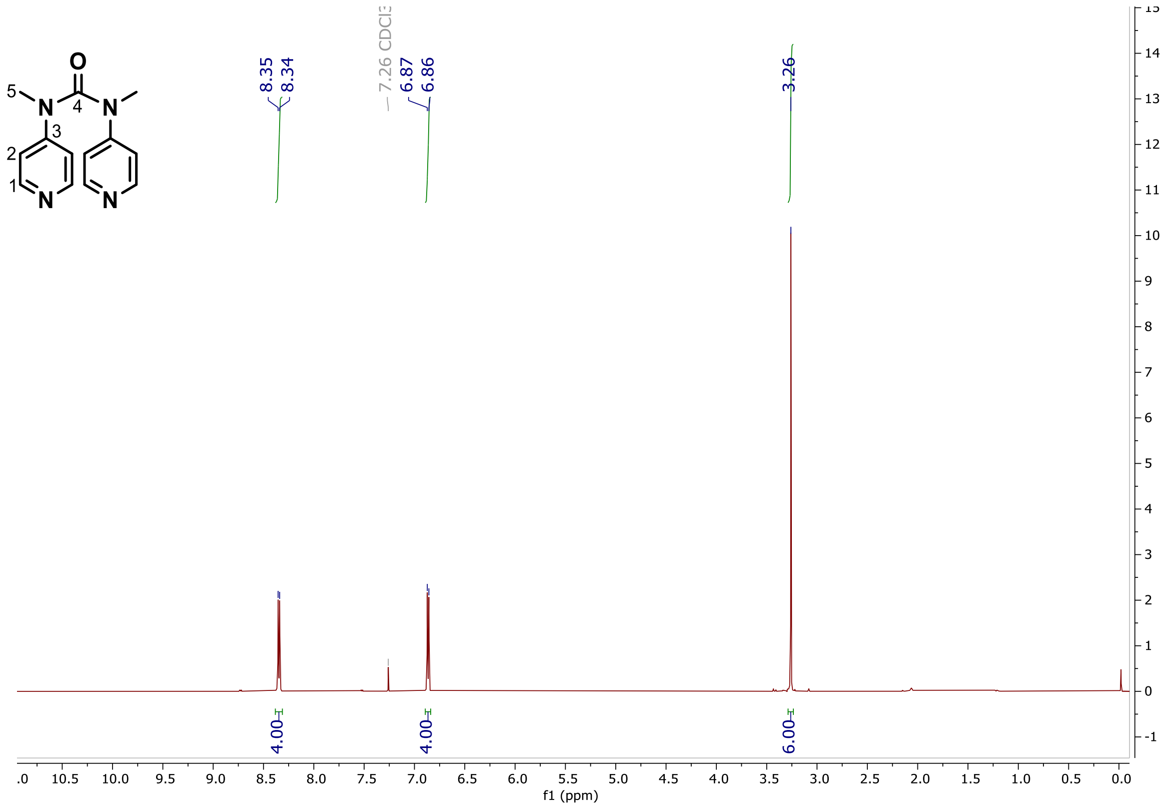

*Supplementary Figure 88. ¹H NMR of N,N'-dimethyl-N,N'-di(pyridin-4-yl)urea (**1am**) (400 MHz, 20 °C, $CDCl_3$)*

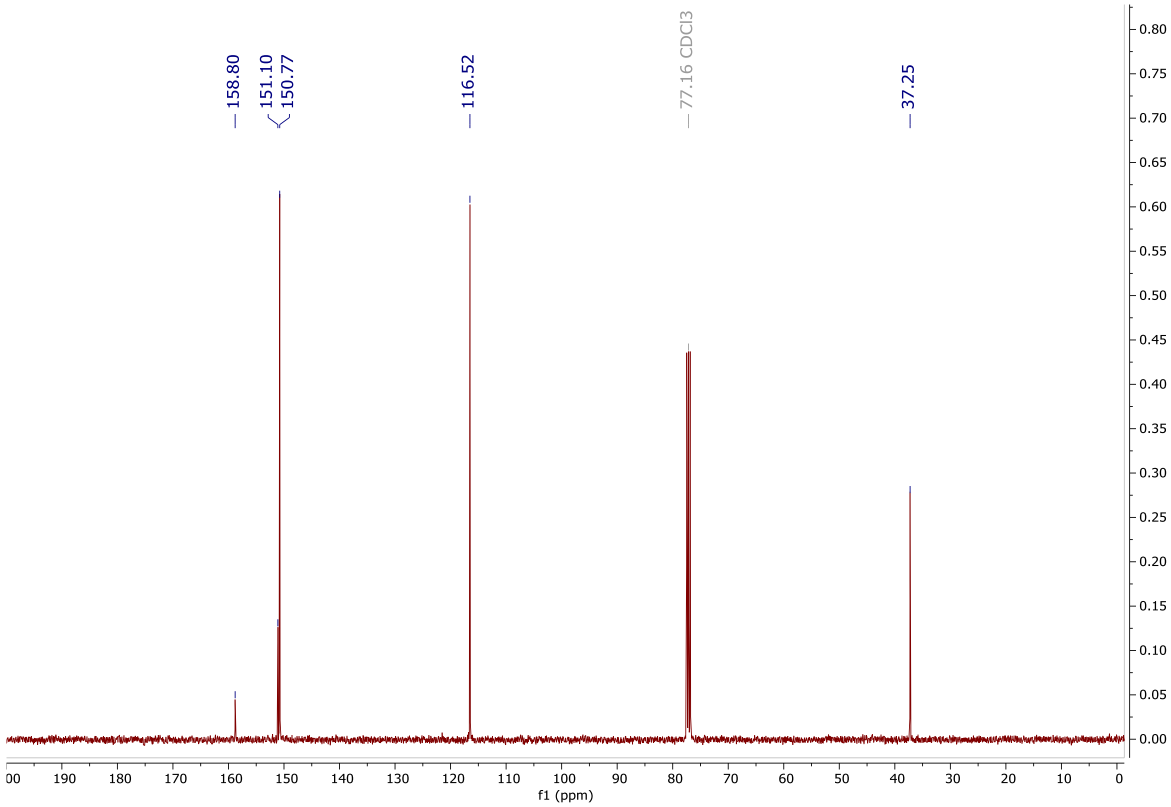

*Supplementary Figure 89. ¹³C NMR of N,N'-dimethyl-N,N'-di(pyridin-4-yl)urea (**1am**) (101 MHz, 20 °C, $CDCl_3$)*

12.1 NMR spectra of biaryl products

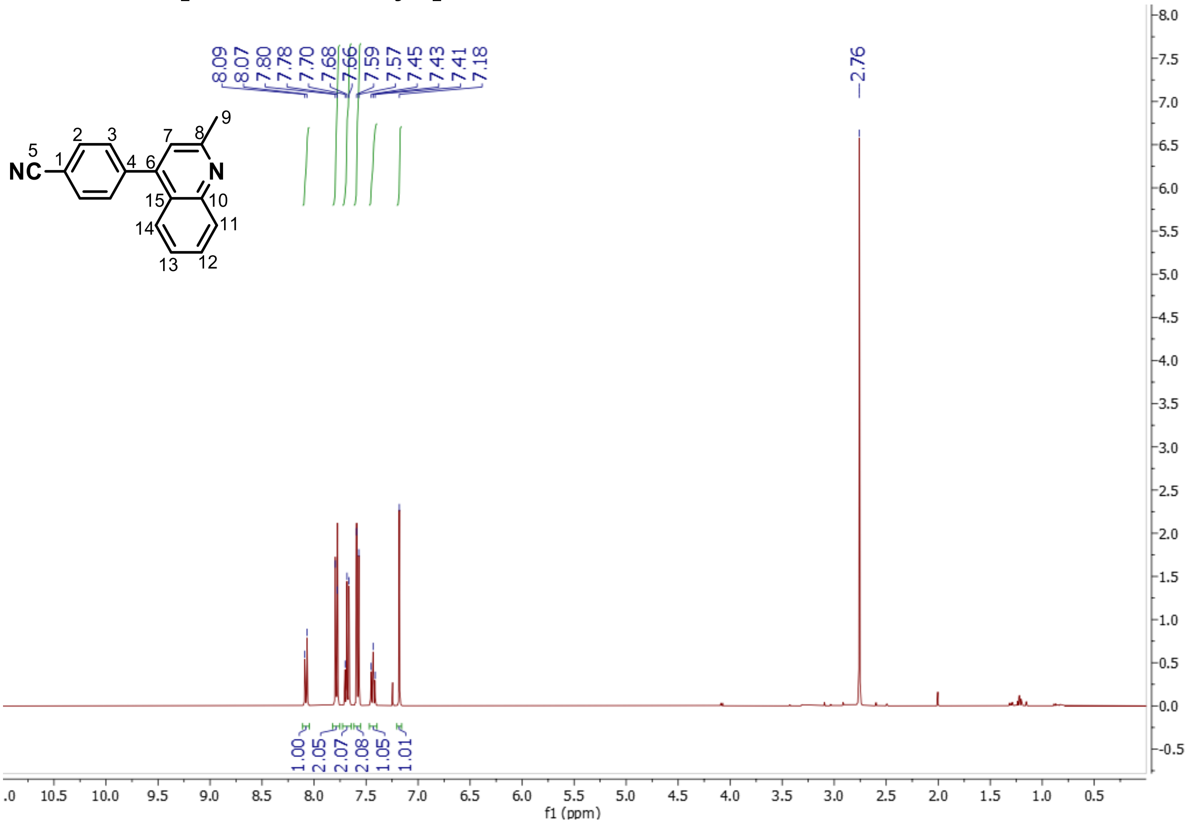

*Supplementary Figure 90. ^{1}H NMR of 4-(2-methylquinolin-4-yl)benzonitrile (**2a**) (400 MHz, 20 °C, $CDCl_3$)*

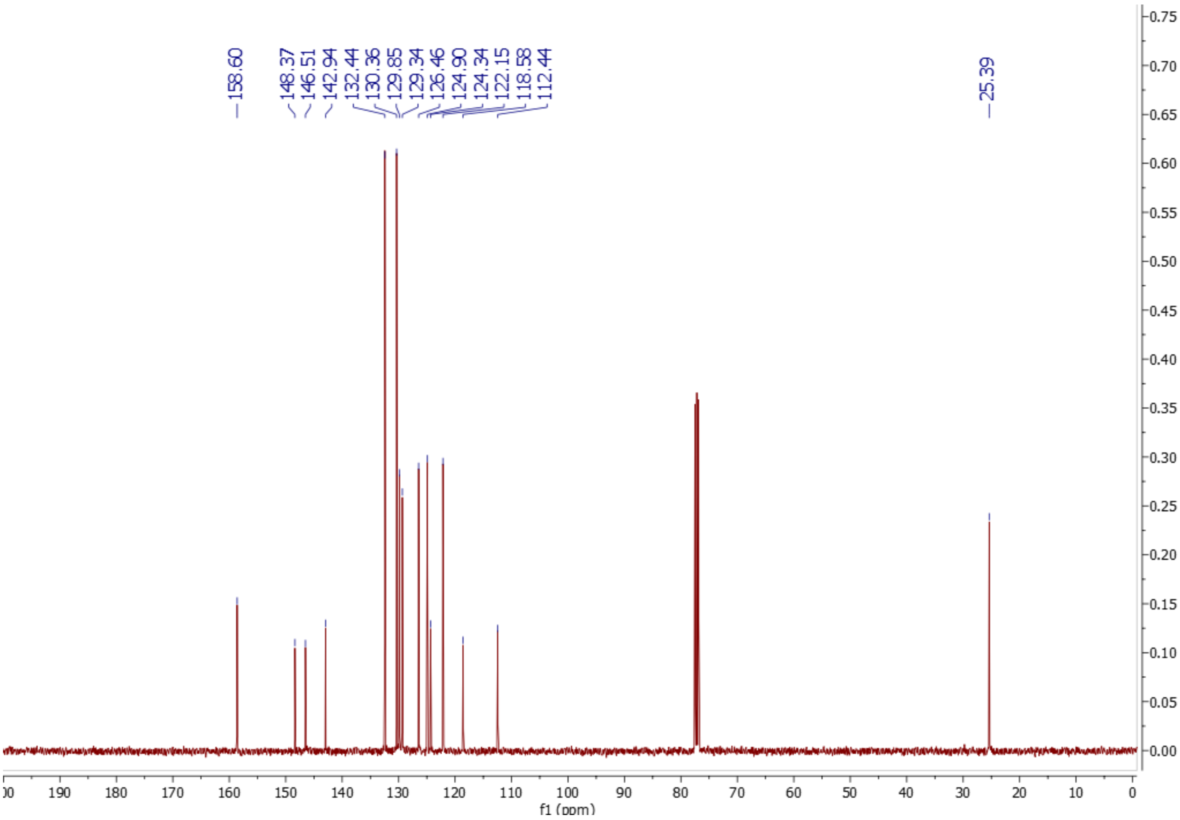

*Supplementary Figure 91. ^{13}C NMR of 4-(2-methylquinolin-4-yl)benzonitrile (**2a**) (101 MHz, 20 °C, $CDCl_3$)*

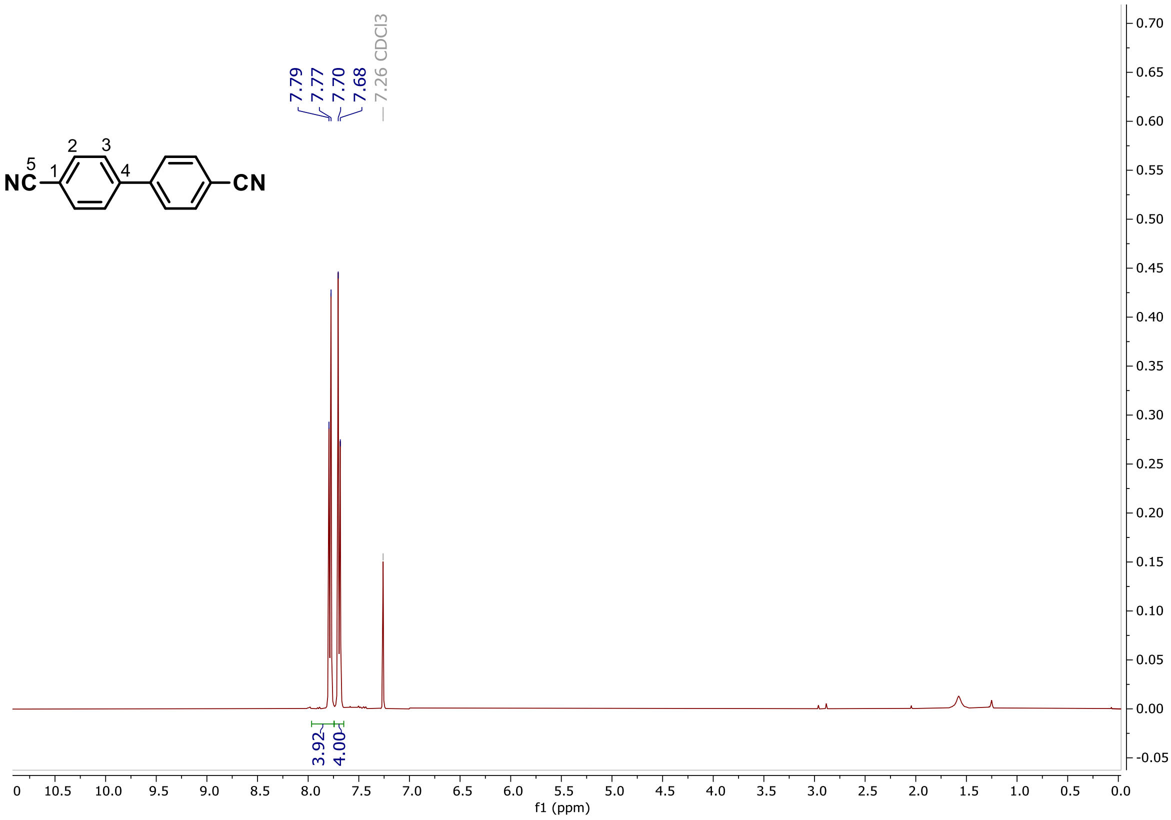

*Supplementary Figure 92. ^{1}H NMR of [1,1'-biphenyl]-4,4'-dicarbonitrile (**2b**) (400 MHz, 20 °C, $CDCl_3$)*

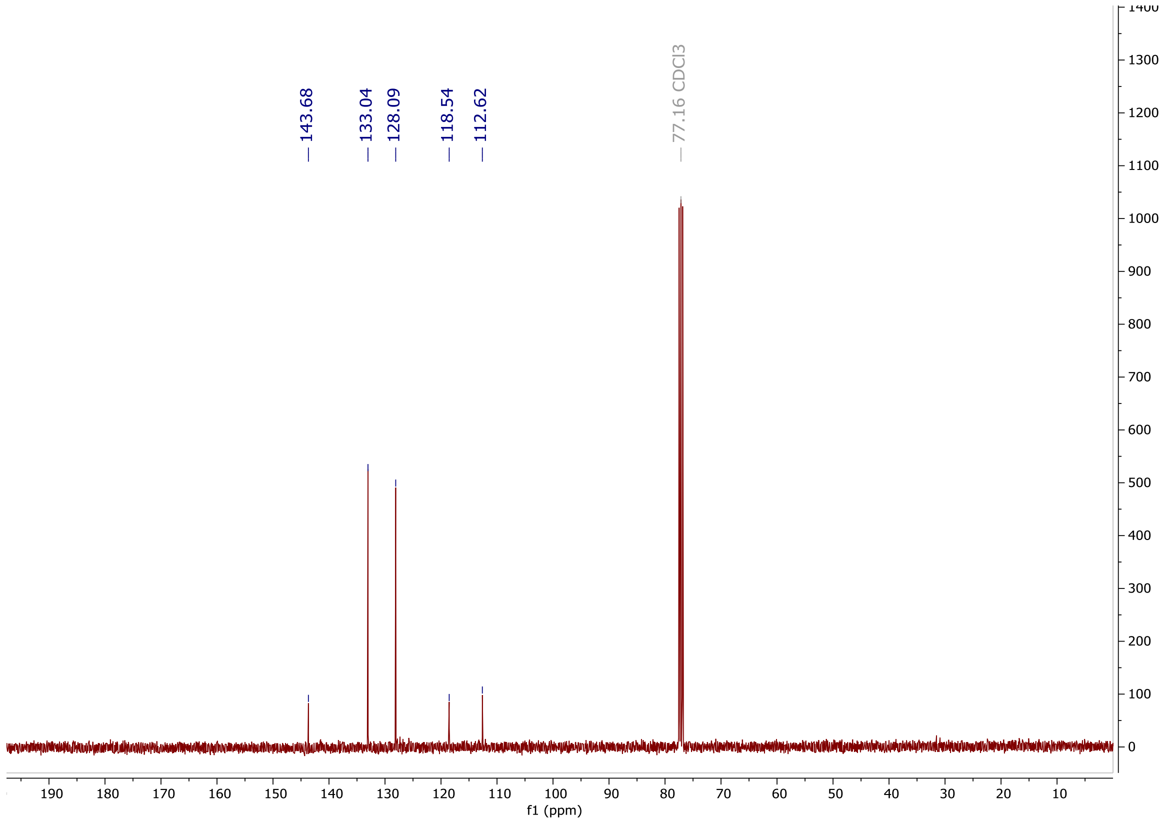

*Supplementary Figure 93. ^{13}C NMR of [1,1'-biphenyl]-4,4'-dicarbonitrile (**2b**) (101 MHz, 20 °C, $CDCl_3$)*

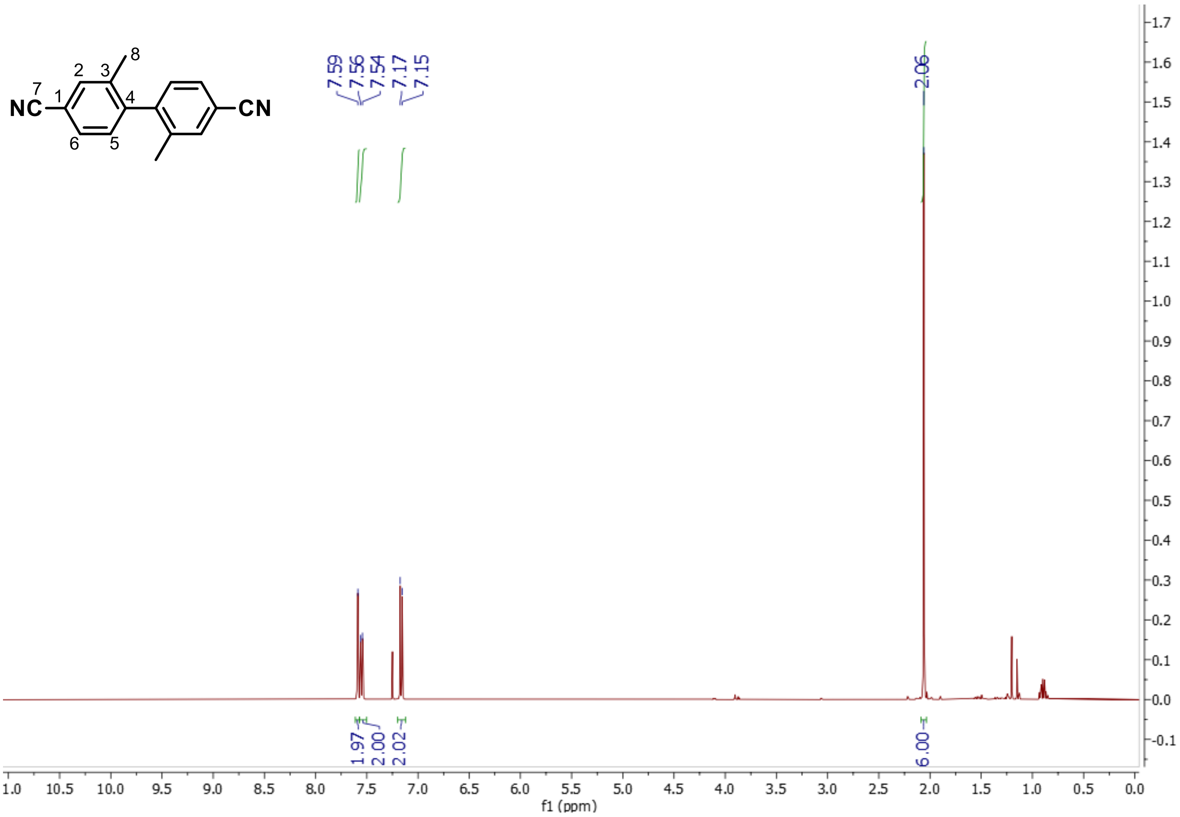

*Supplementary Figure 94. ¹H NMR of 2,2'-dimethyl-[1,1'-biphenyl]-4,4'-dicarbonitrile (**2c**) (400 MHz, 20 °C, $CDCl_3$)*

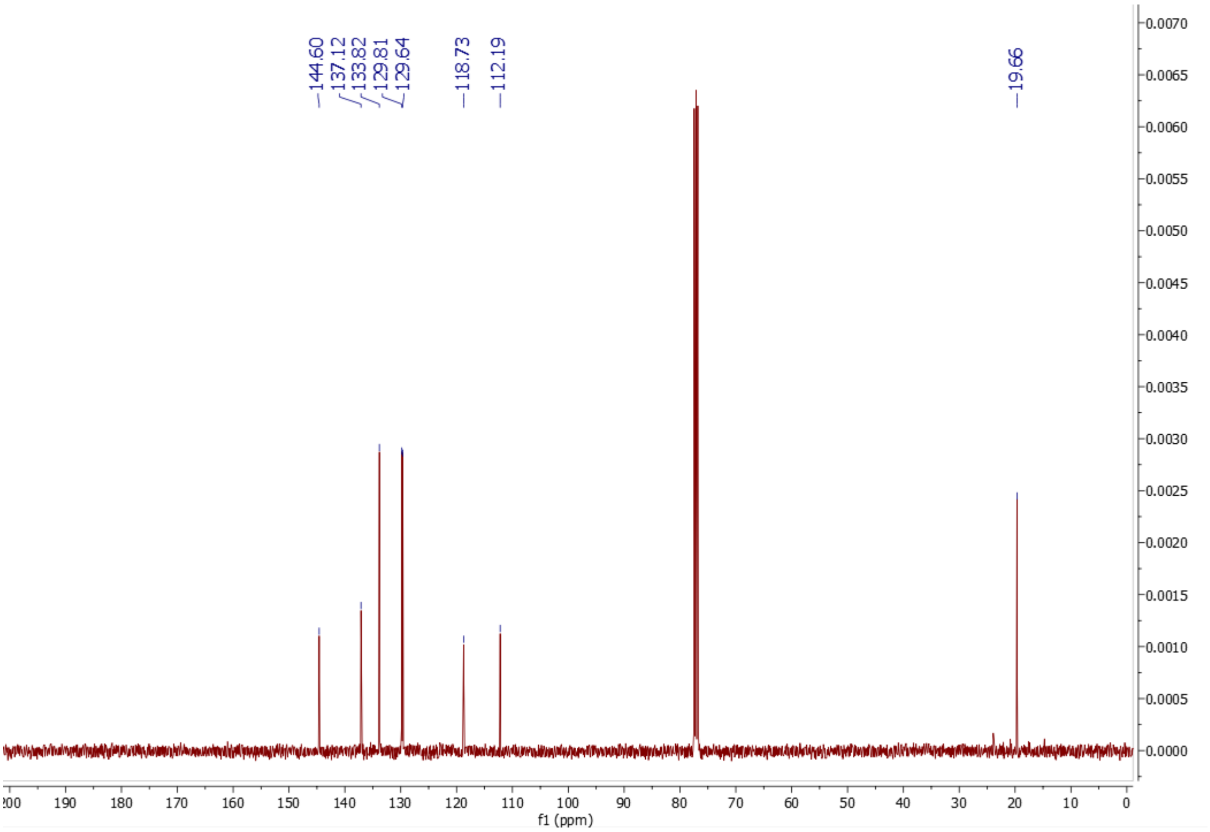

*Supplementary Figure 95. ¹³C NMR of 2,2'-dimethyl-[1,1'-biphenyl]-4,4'-dicarbonitrile (**2c**) (101 MHz, 20 °C, $CDCl_3$)*

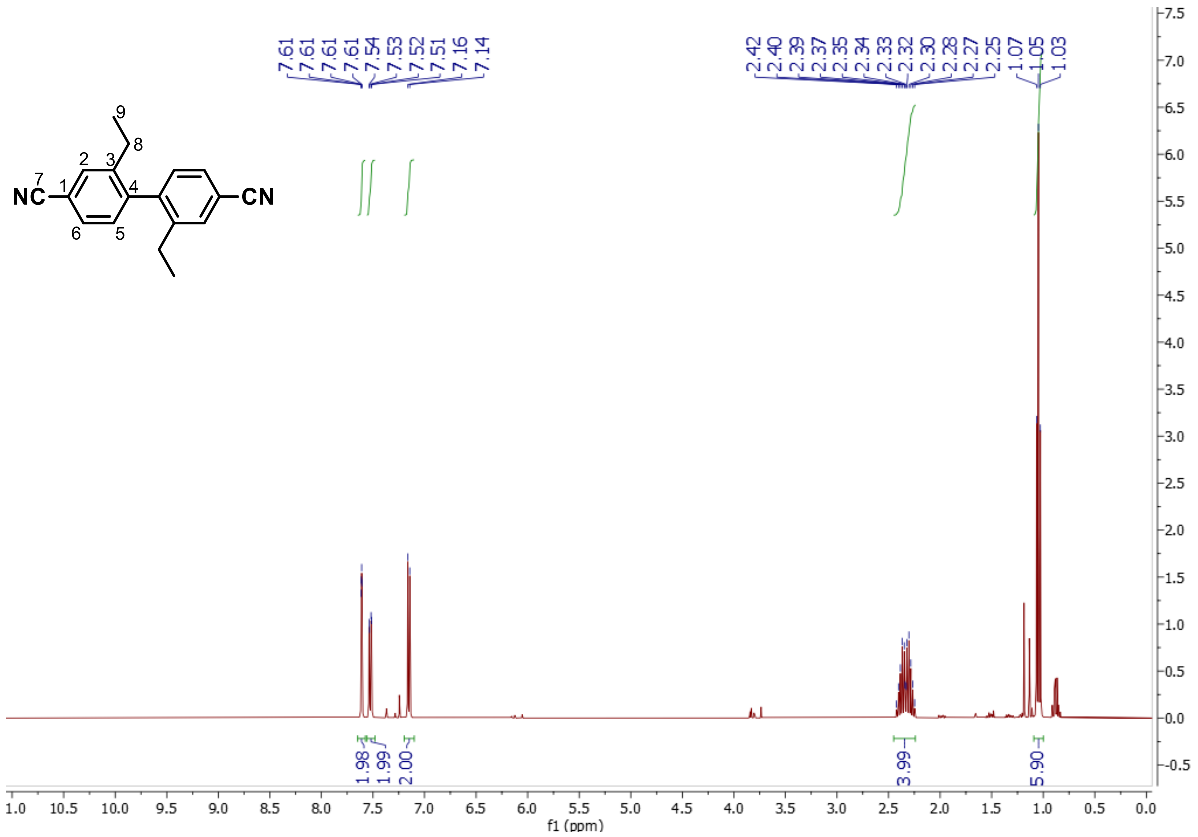

*Supplementary Figure 96. ^{1}H NMR of 2,2'-diethyl-[1,1'-biphenyl]-4,4'-dicarbonitrile (**2d**) (400 MHz, 20 °C, $CDCl_3$)*

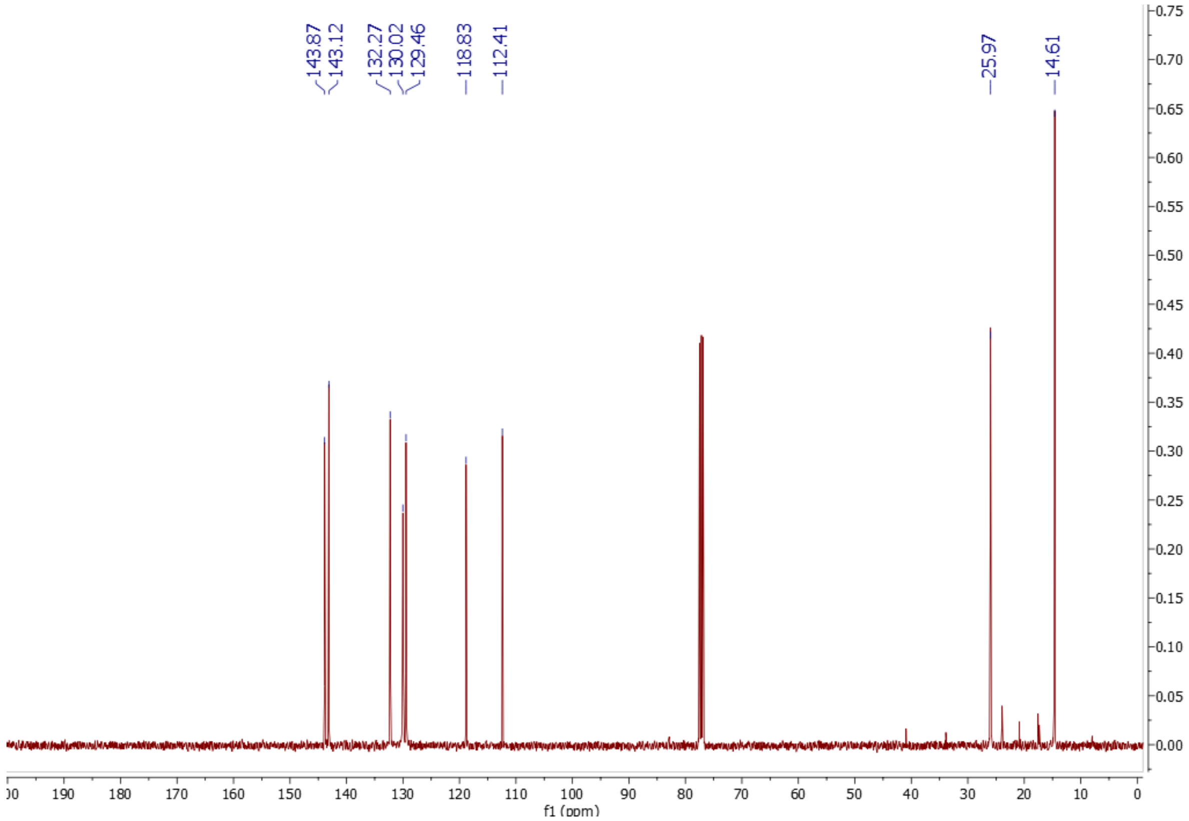

*Supplementary Figure 97. ^{13}C NMR of 2,2'-diethyl-[1,1'-biphenyl]-4,4'-dicarbonitrile (**2d**) (101 MHz, 20 °C, $CDCl_3$)*

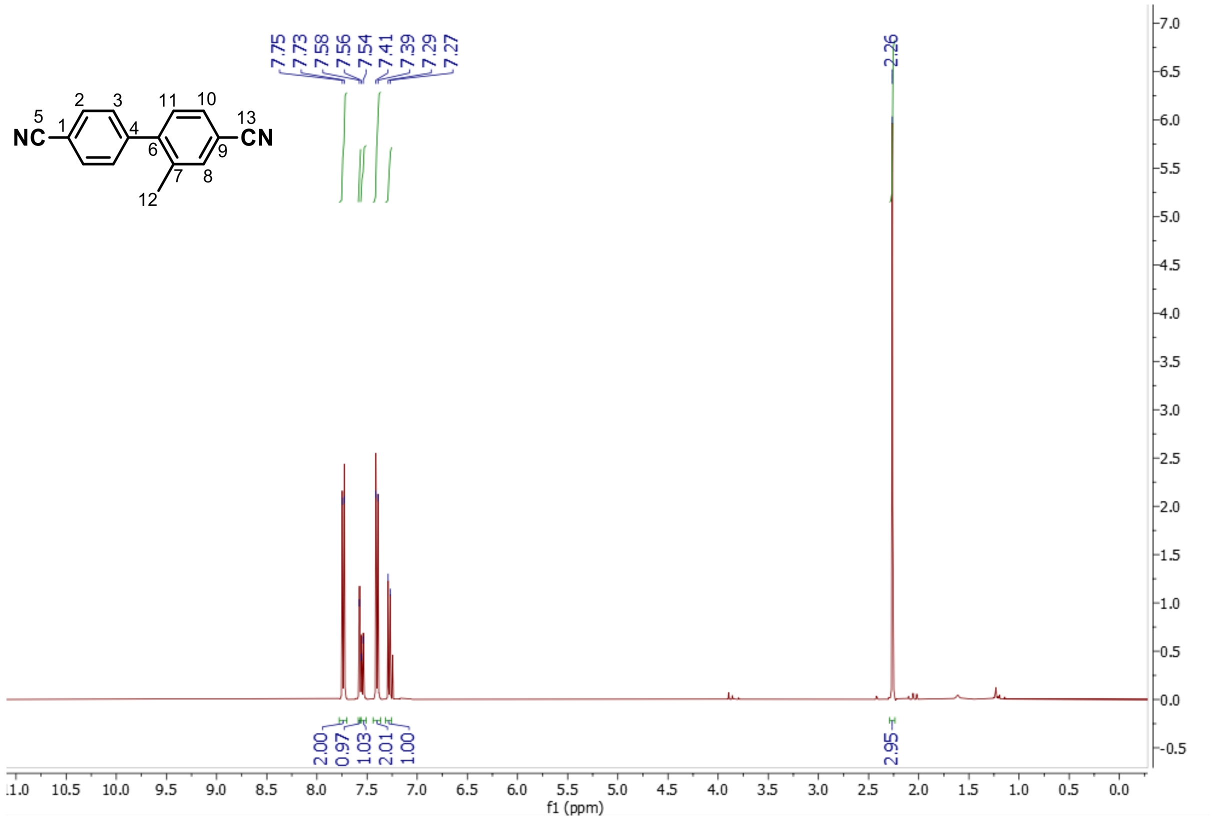

*Supplementary Figure 98. ^{1}H NMR of 2-methyl-[1,1'-biphenyl]-4,4'-dicarbonitrile (**2e**) (400 MHz, 20 °C, $CDCl_3$)*

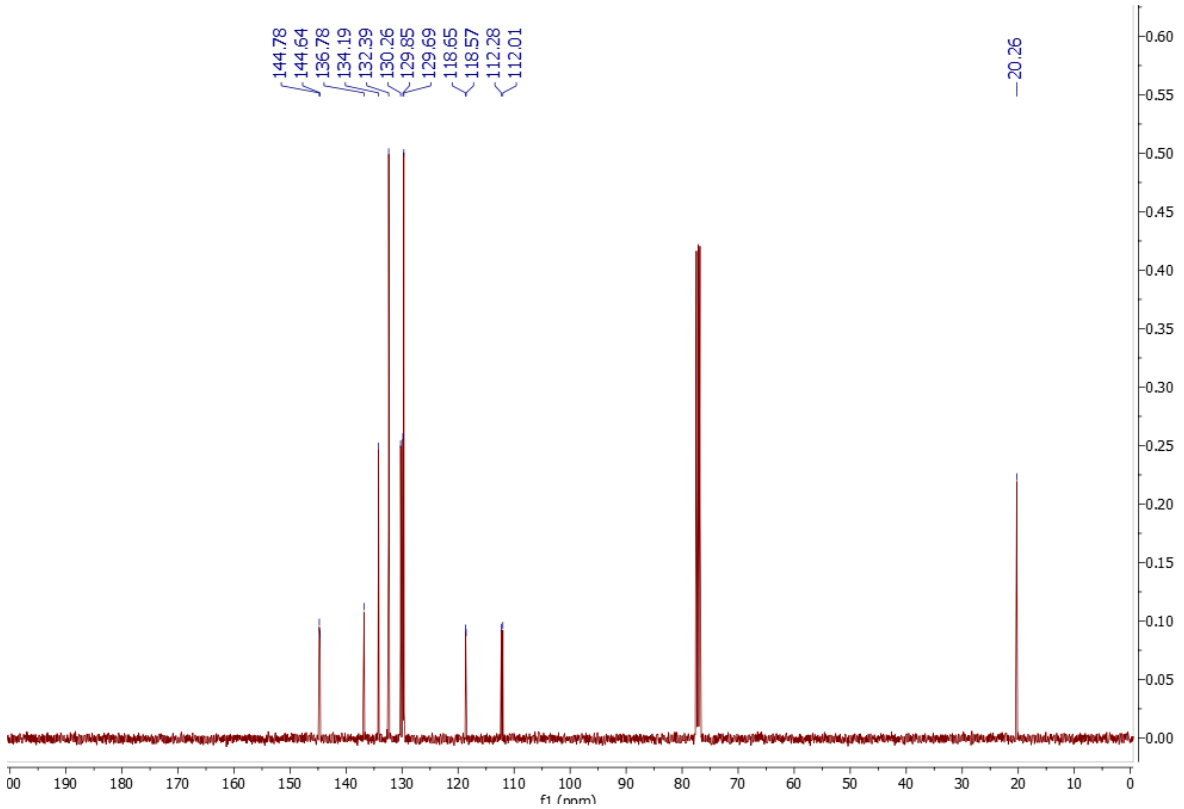

*Supplementary Figure 99. ^{13}C NMR of 2-methyl-[1,1'-biphenyl]-4,4'-dicarbonitrile (**2e**) (101 MHz, 20 °C, $CDCl_3$)*

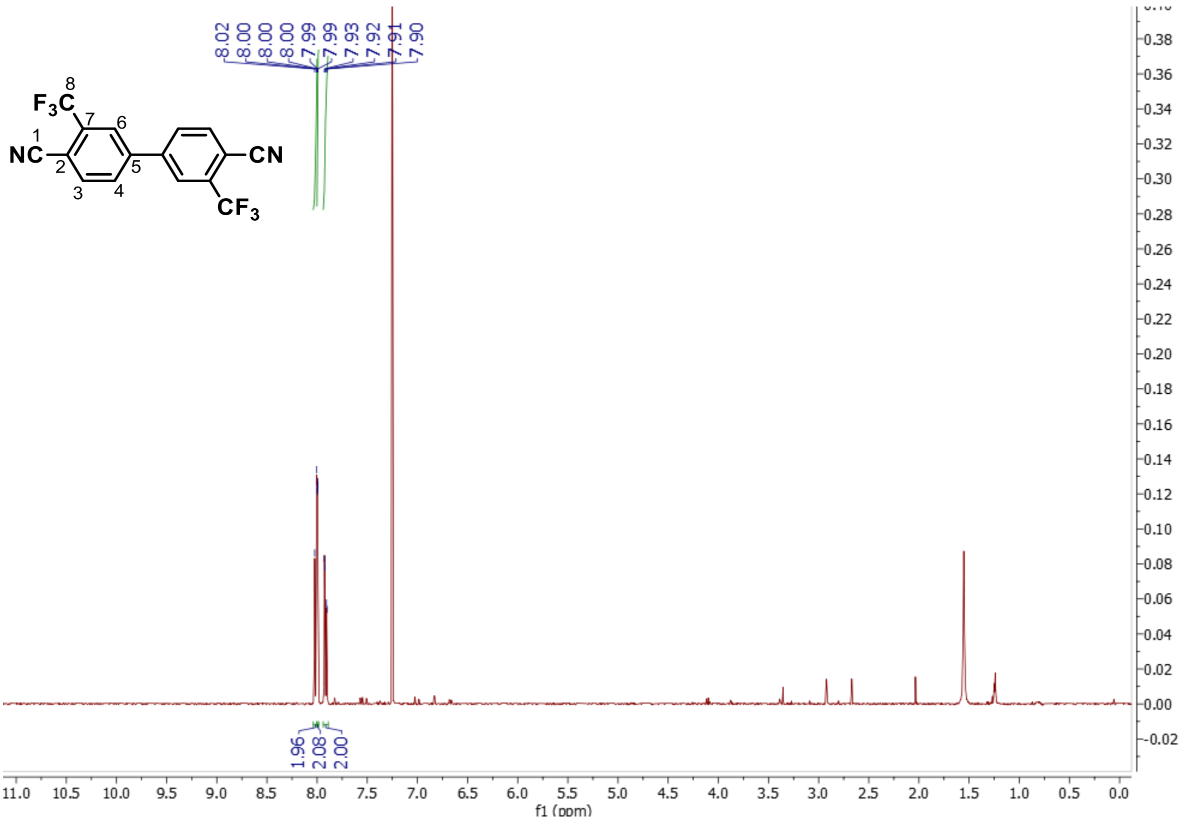

Supplementary Figure 100. ¹H NMR of 3,3'-bis(trifluoromethyl)-[1,1'-biphenyl]-4,4'-dicarbonitrile (**2f**) (400 MHz, 20 °C, $CDCl_3$)

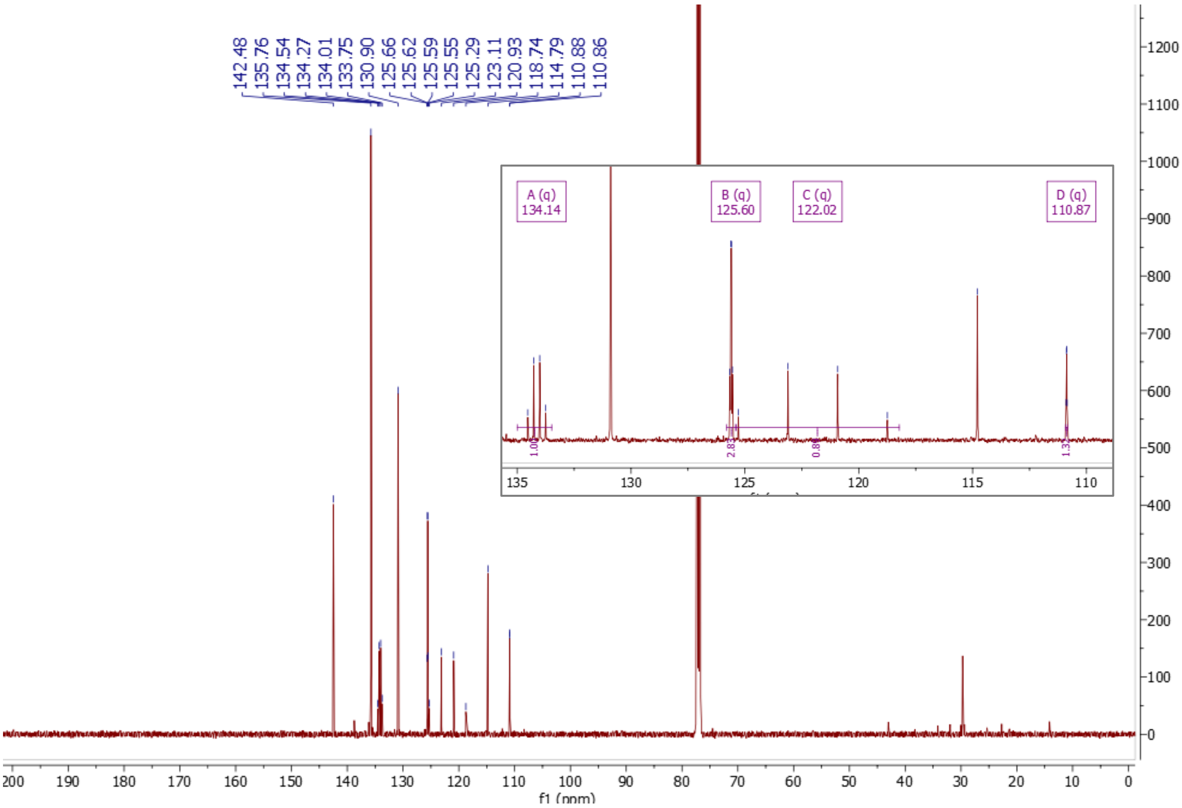

Supplementary Figure 101. ¹³C NMR of 3,3'-bis(trifluoromethyl)-[1,1'-biphenyl]-4,4'-dicarbonitrile (**2f**) (126 MHz, 20 °C, $CDCl_3$)

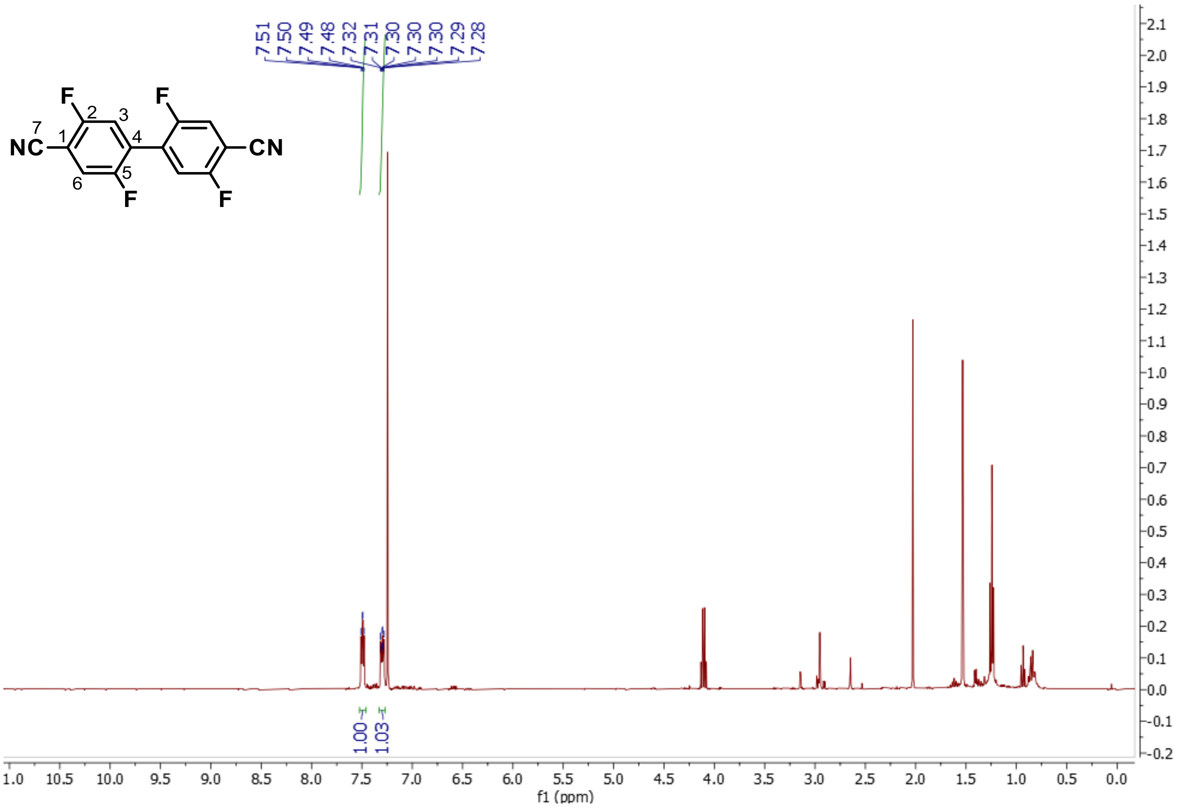

*Supplementary Figure 102. ^{1}H NMR of 2,2',5,5'-tetrafluoro-[1,1'-biphenyl]-4,4'-dicarbonitrile (**2g**) (400 MHz, 20 °C, $CDCl_3$)*

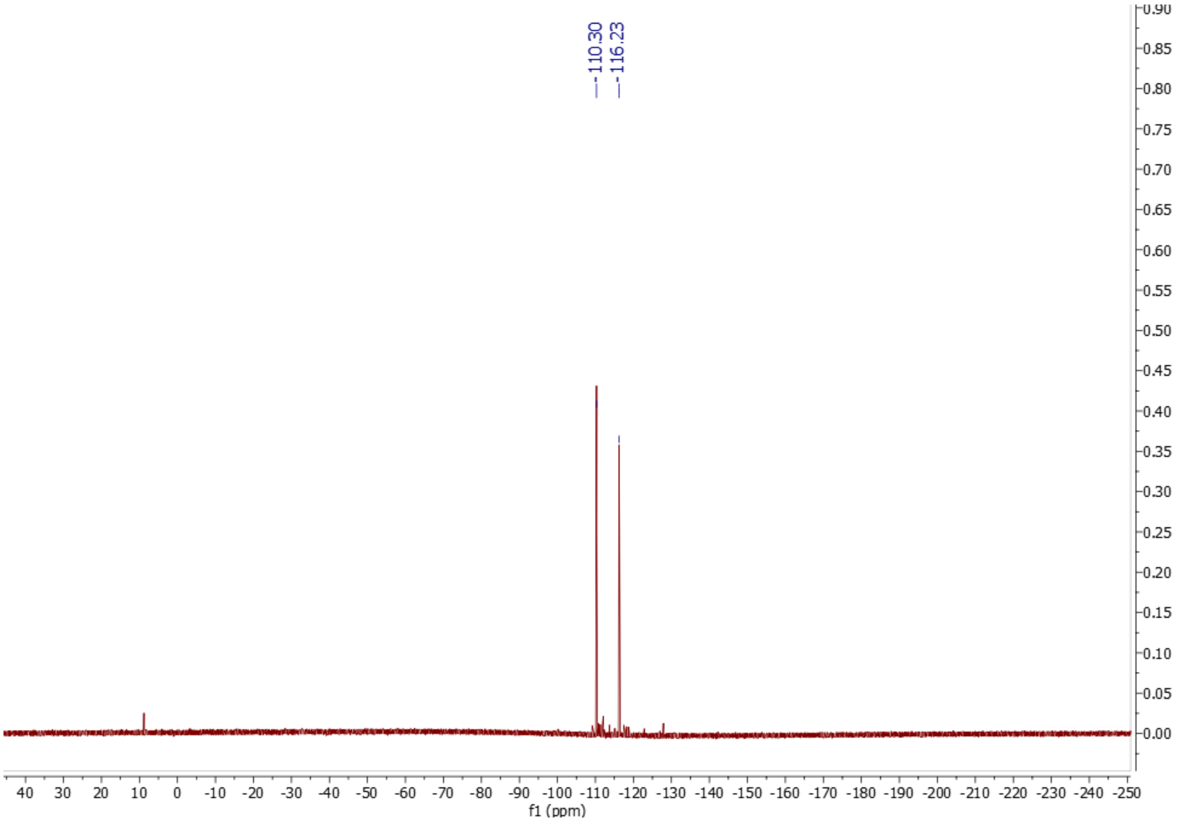

*Supplementary Figure 103. ^{19}F NMR of 2,2',5,5'-tetrafluoro-[1,1'-biphenyl]-4,4'-dicarbonitrile (**2g**) (282 MHz, 20 °C, $CDCl_3$)*

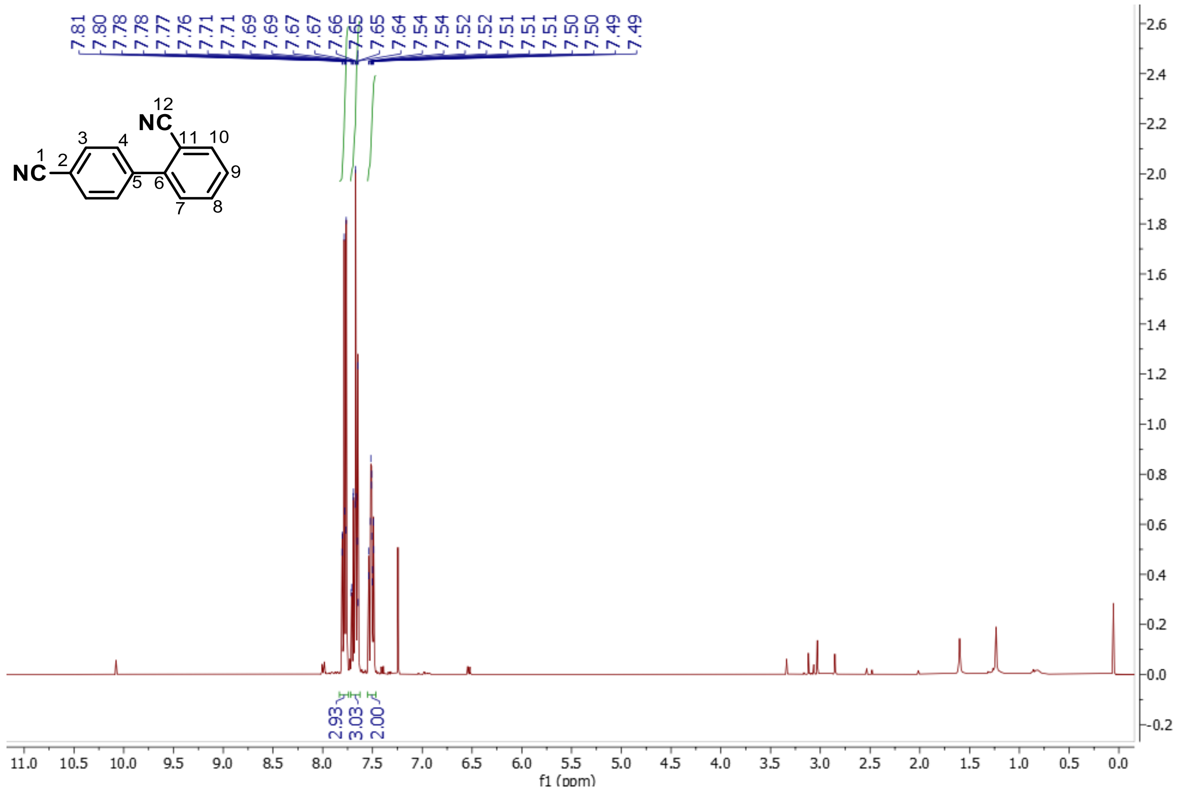

Supplementary Figure 104. ^{1}H NMR of [1,1'-biphenyl]-2,4'-dicarbonitrile (**2h**) (400 MHz, 20 °C, $CDCl_3$)

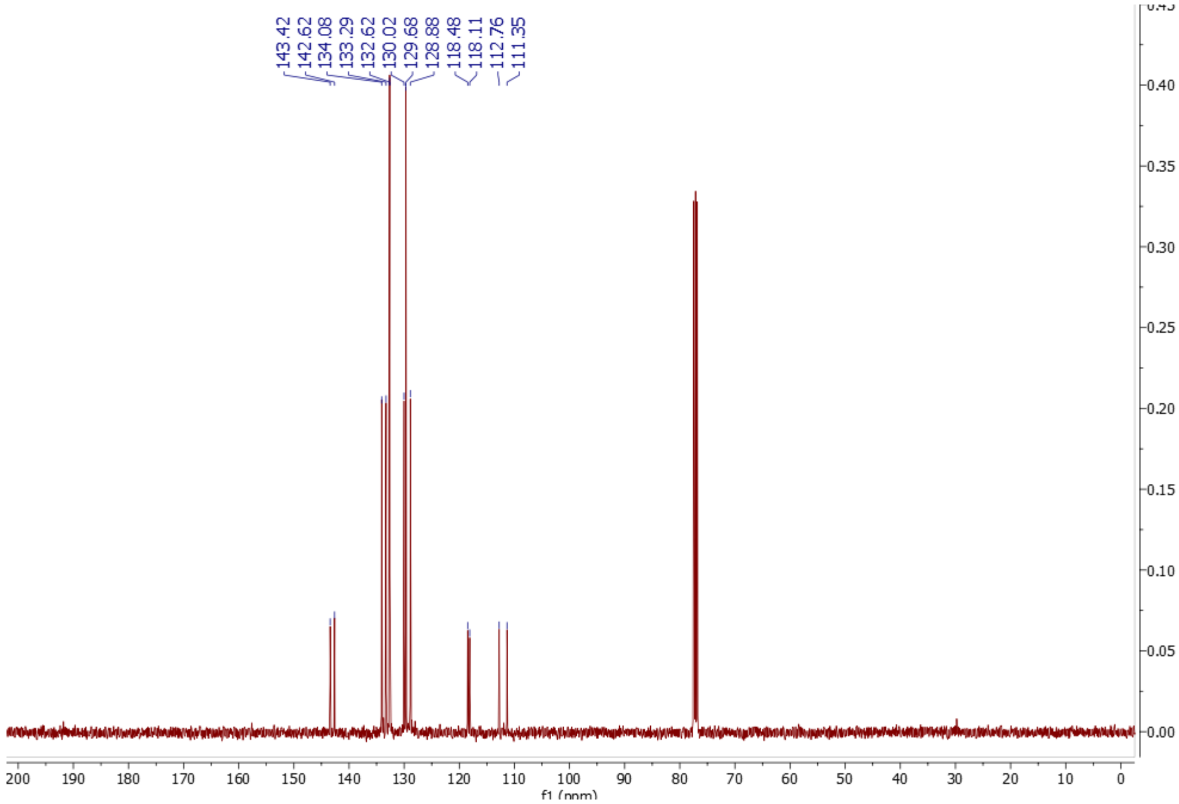

Supplementary Figure 105. ^{13}C NMR of [1,1'-biphenyl]-2,4'-dicarbonitrile (**2h**) (101 MHz, 20 °C, $CDCl_3$)

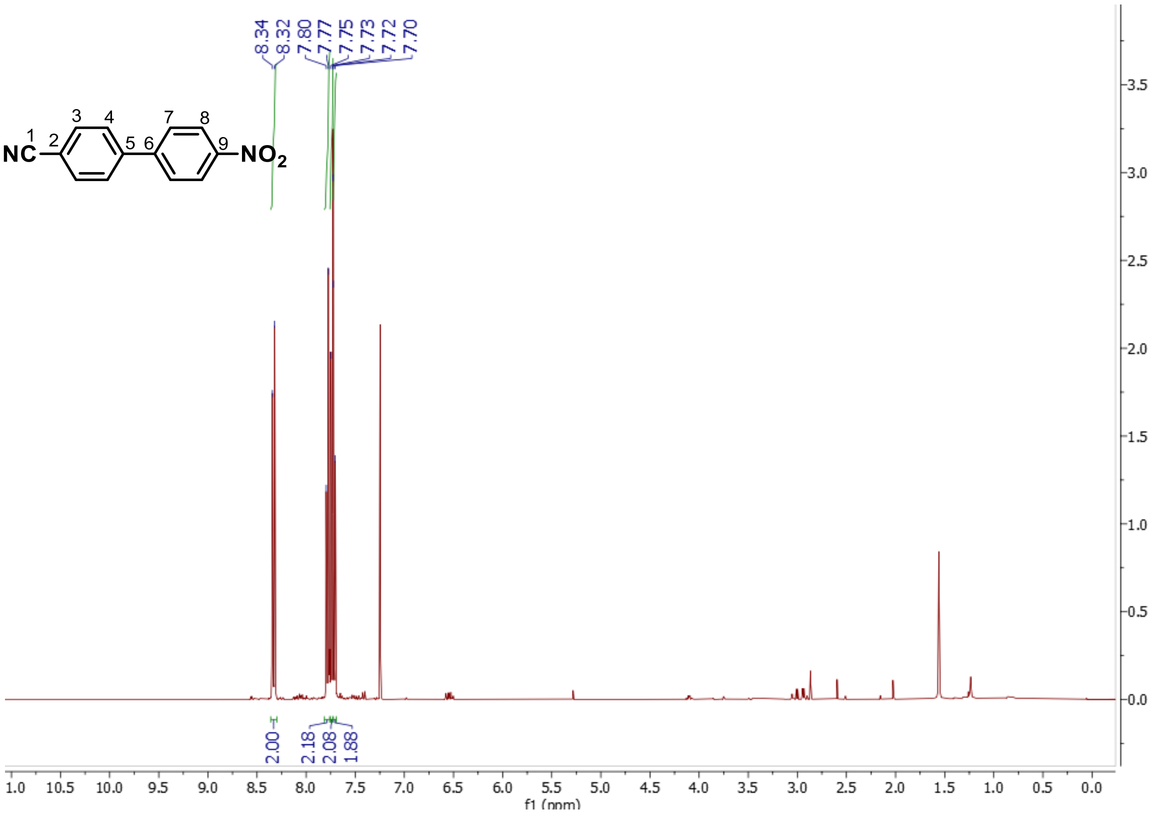

*Supplementary Figure 106. ^{1}H NMR of 4'-nitro-[1,1'-biphenyl]-4-carbonitrile (**2i**) (400 MHz, 20 °C, $CDCl_3$)*

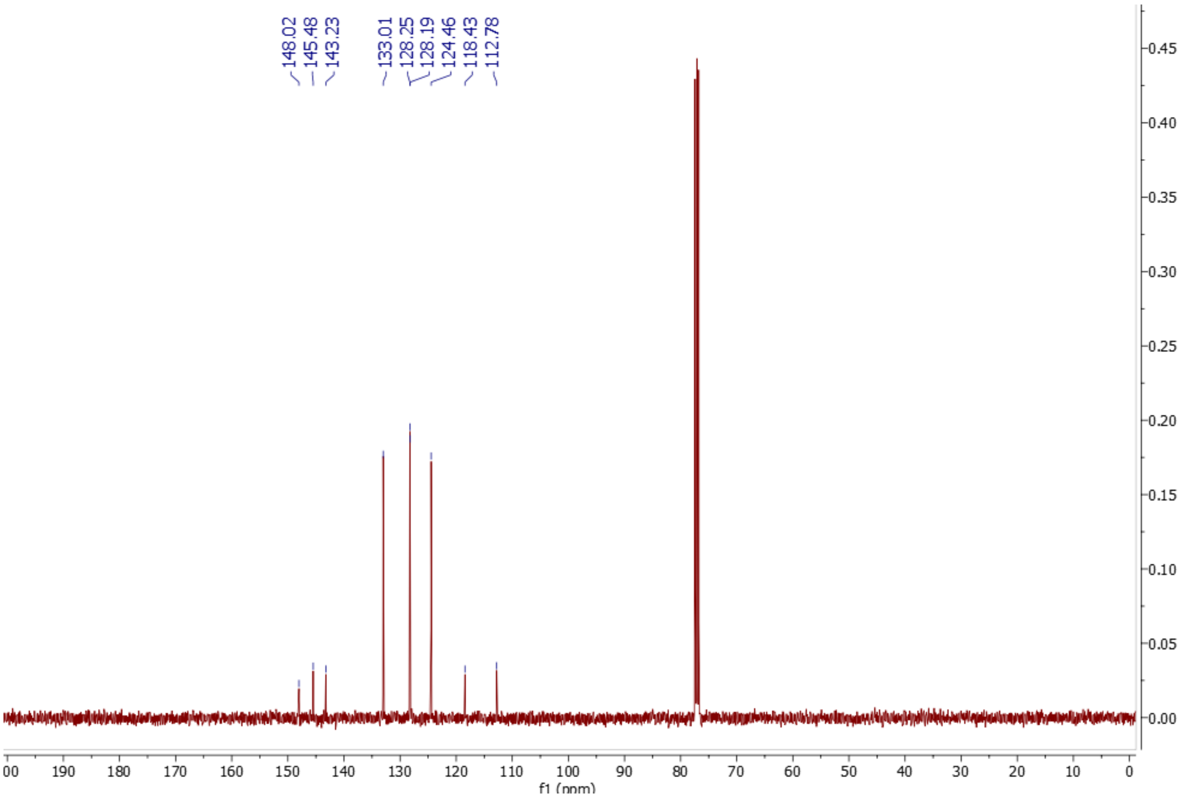

*Supplementary Figure 107. ^{13}C NMR of 4'-nitro-[1,1'-biphenyl]-4-carbonitrile (**2i**) (101 MHz, 20 °C, $CDCl_3$)*

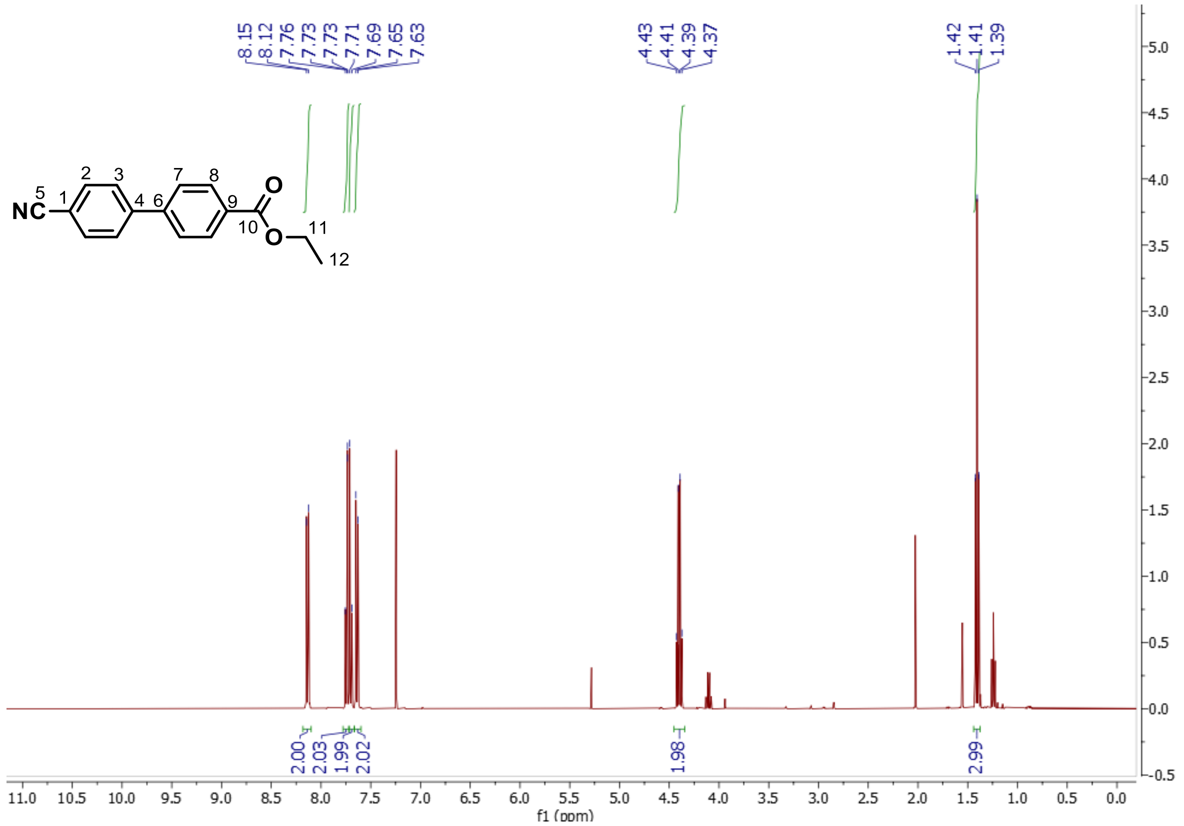

Supplementary Figure 108. *¹H NMR of ethyl 4'-cyano-[1,1'-biphenyl]-4-carboxylate (**2j**) (400 MHz, 20 °C, $CDCl_3$)*

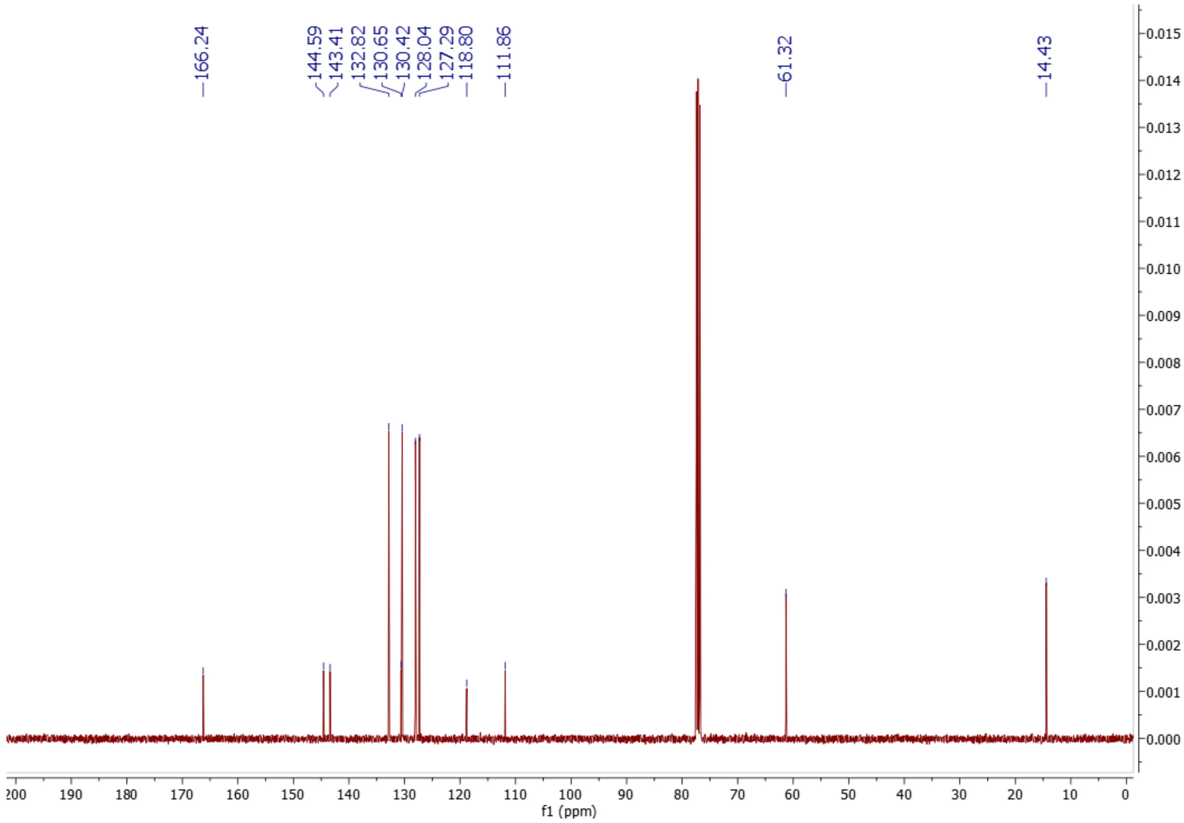

Supplementary Figure 109. *¹³C NMR of ethyl 4'-cyano-[1,1'-biphenyl]-4-carboxylate (**2j**) (101 MHz, 20 °C, $CDCl_3$)*

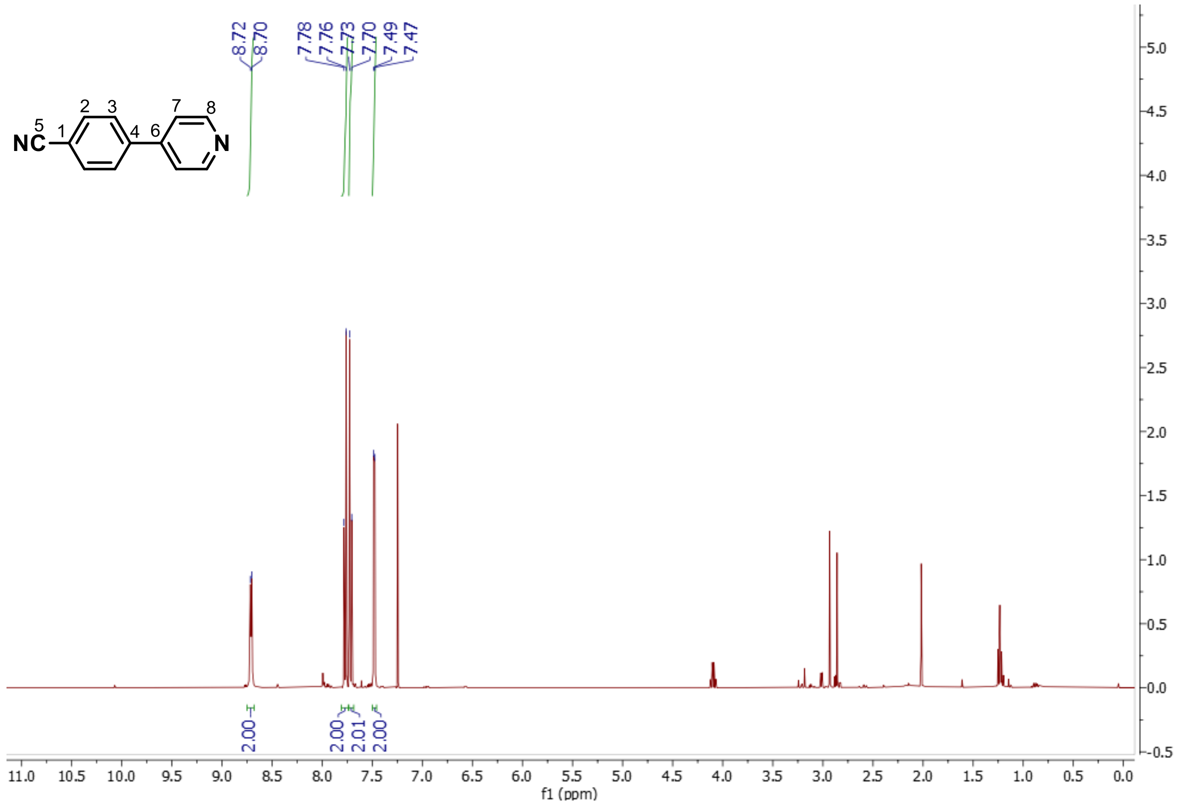

*Supplementary Figure 110. ¹H NMR of 4-(pyridin-4-yl)benzonitrile (**2k**) (400 MHz, 20 °C, $CDCl_3$)*

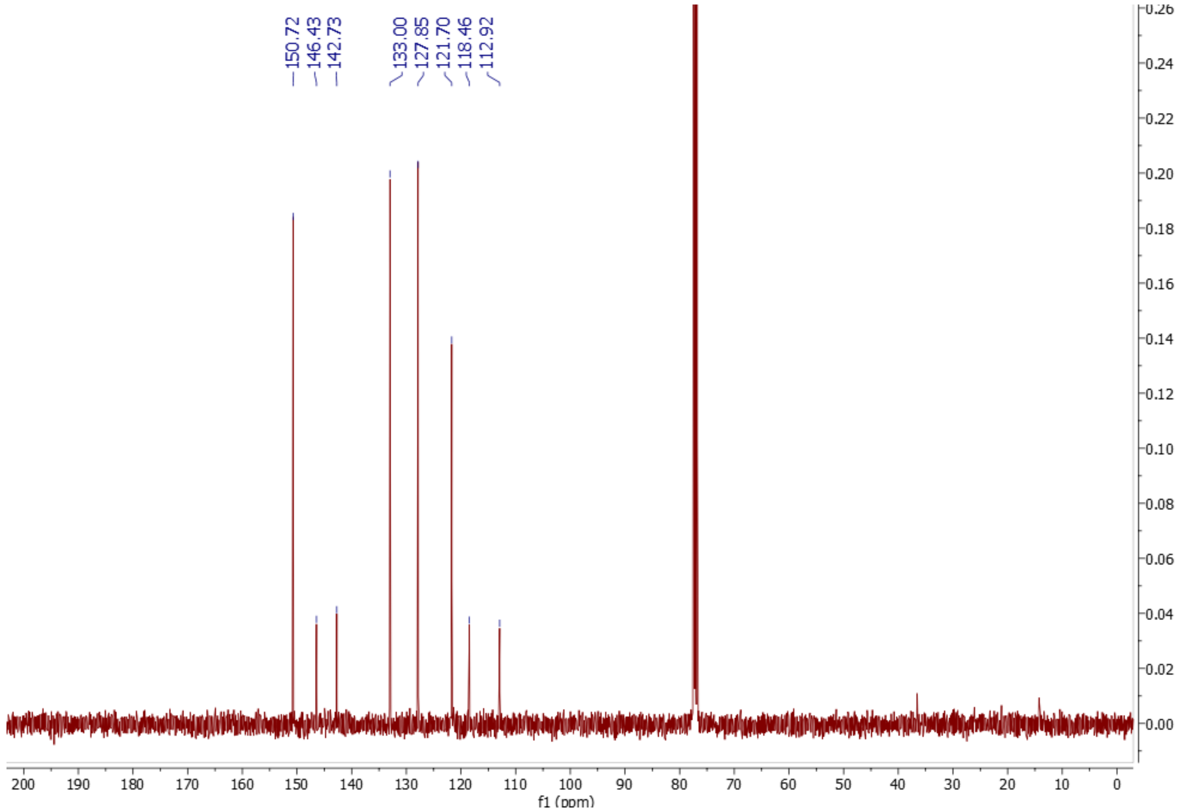

*Supplementary Figure 111. ¹³C NMR of 4-(pyridin-4-yl)benzonitrile (**2k**) (101 MHz, 20 °C, $CDCl_3$)*

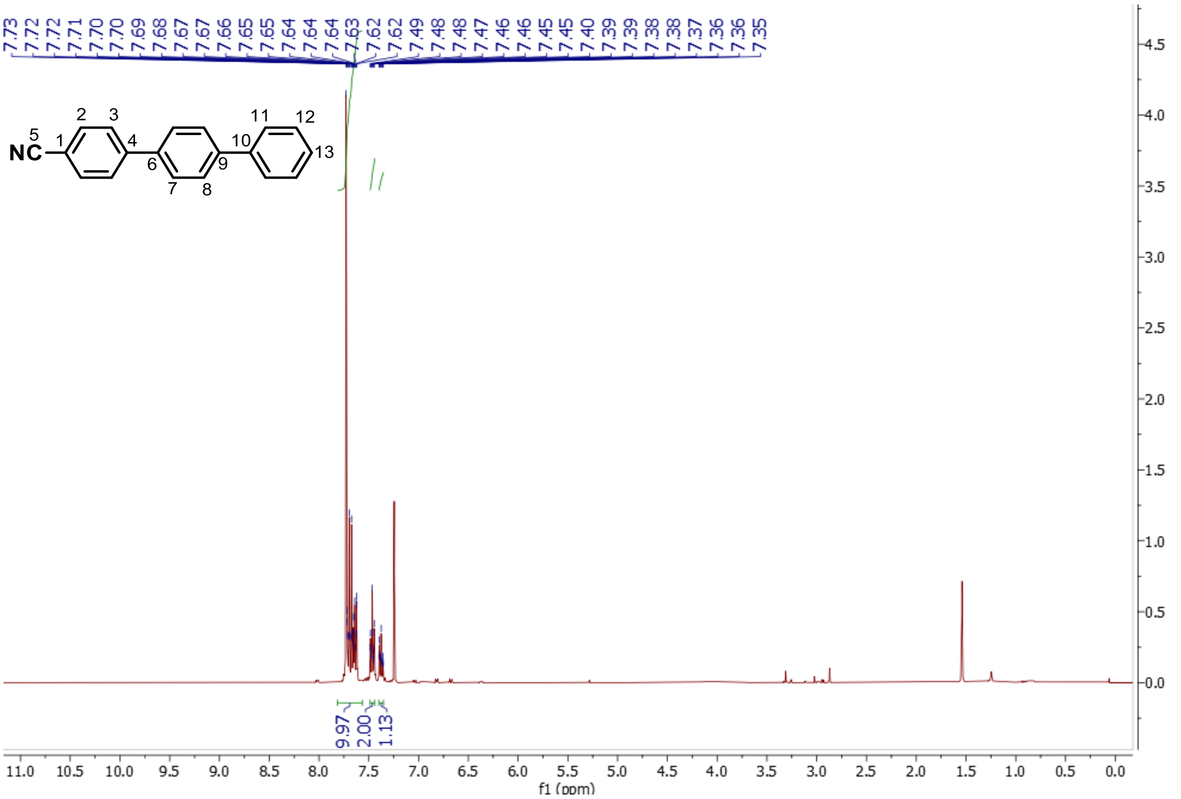

*Supplementary Figure 112. ¹H NMR of [1,1':4',1''-Terphenyl]-4-carbonitrile (**2l**) (400 MHz, 20 °C, $CDCl_3$)*

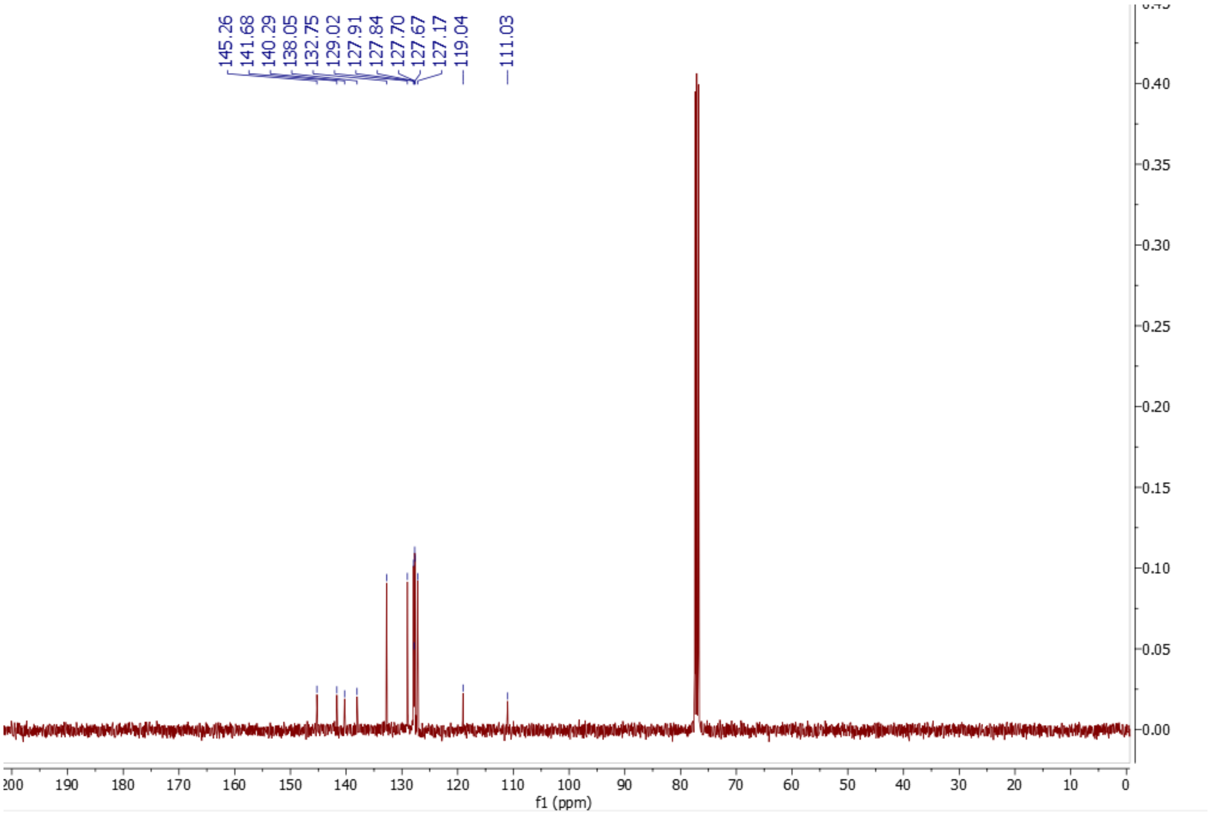

*Supplementary Figure 113. ¹³C NMR of [1,1':4',1''-Terphenyl]-4-carbonitrile (**2l**) (101 MHz, 20 °C, $CDCl_3$)*

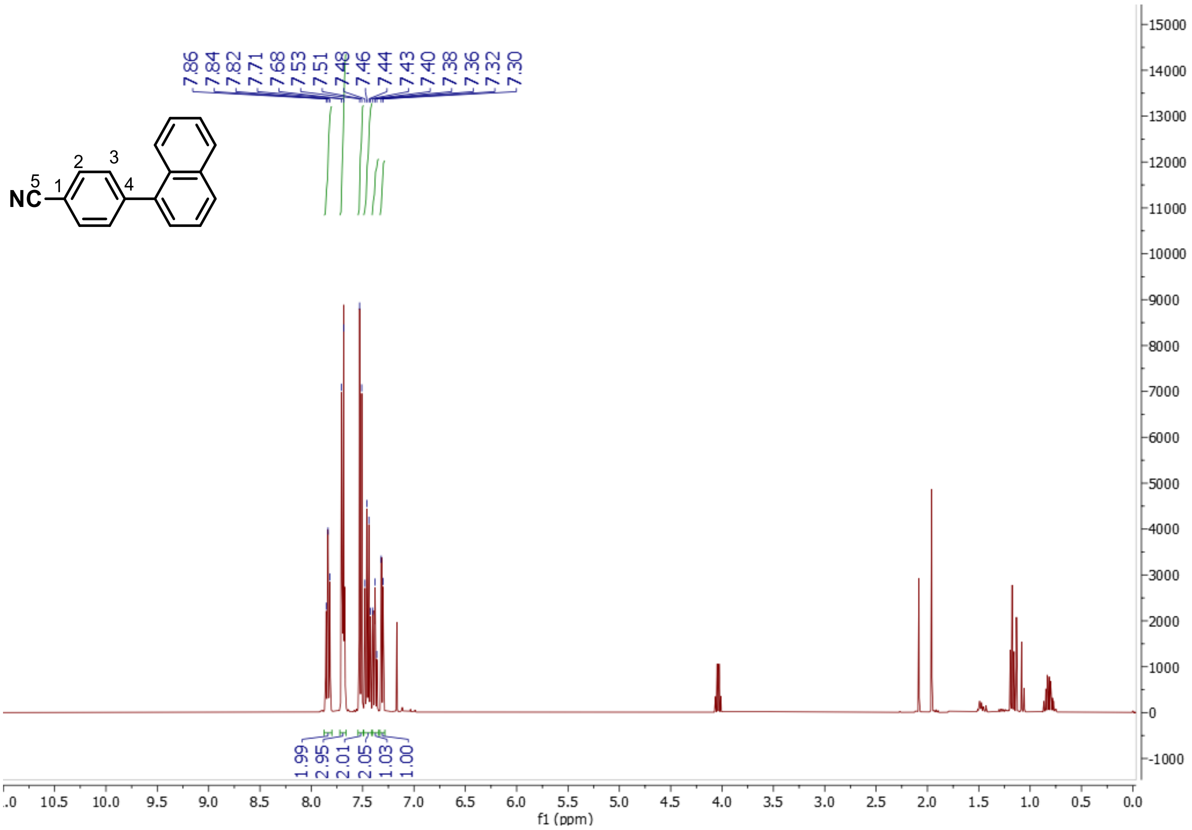

*Supplementary Figure 114. ¹H NMR of 4-(naphthalen-1-yl)benzonitrile (**2m**) (400 MHz, 20 °C, CDCl₃)*

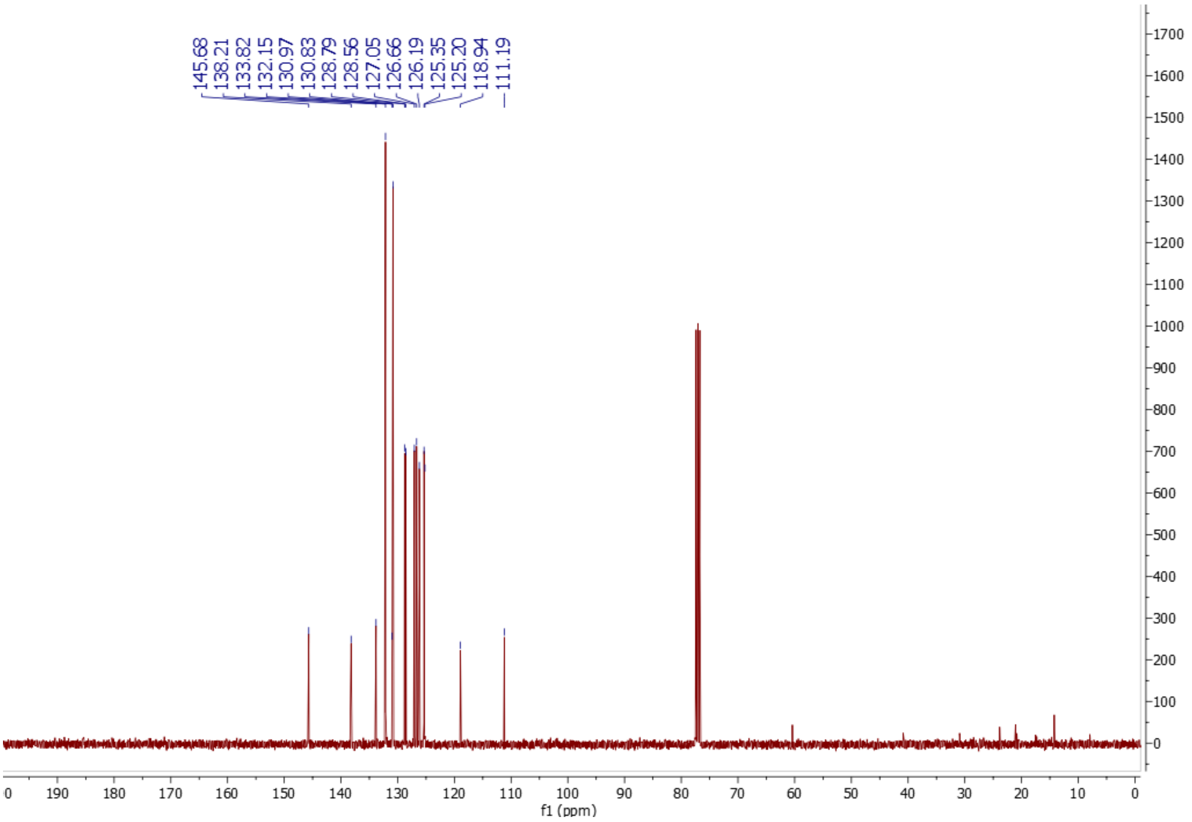

*Supplementary Figure 115. ¹³C NMR of 4-(naphthalen-1-yl)benzonitrile (**2m**) (101 MHz, 20 °C, CDCl₃)*

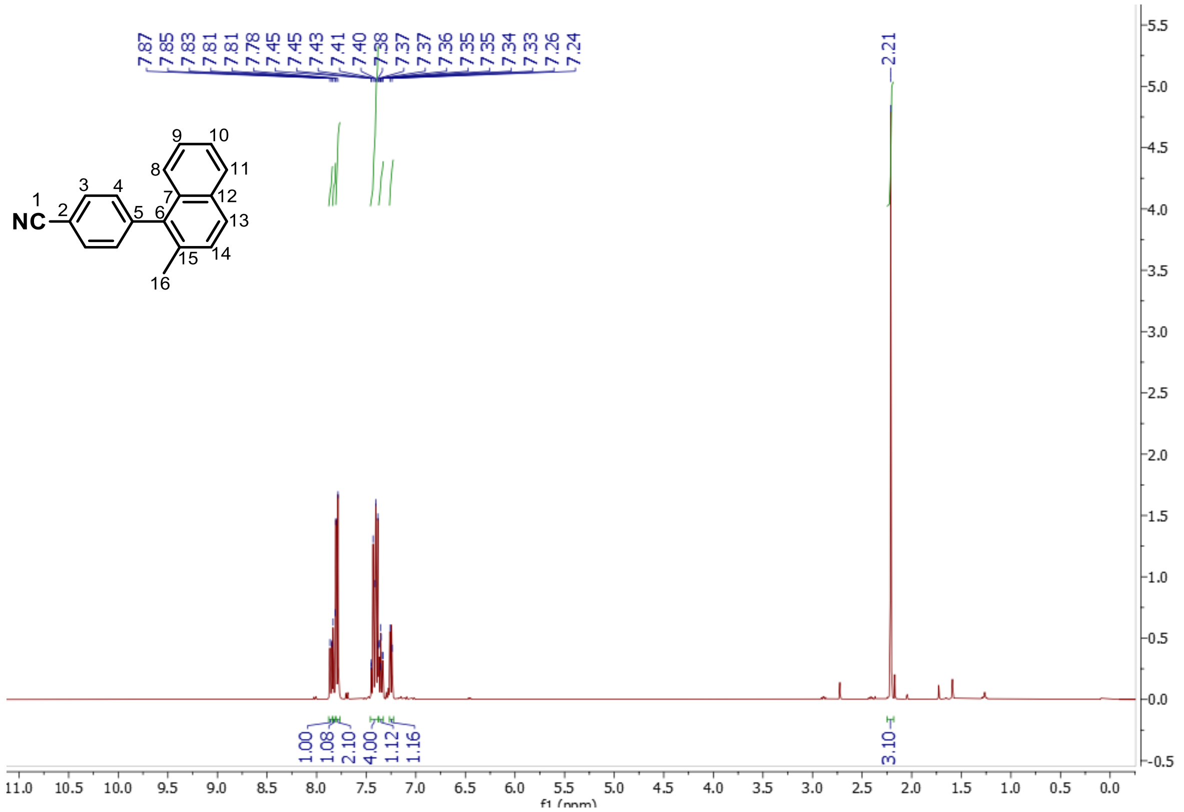

*Supplementary Figure 116. ¹H NMR of 4-(naphthalen-1-yl)benzonitrile (**2n**) (400 MHz, 20 °C, $CDCl_3$)*

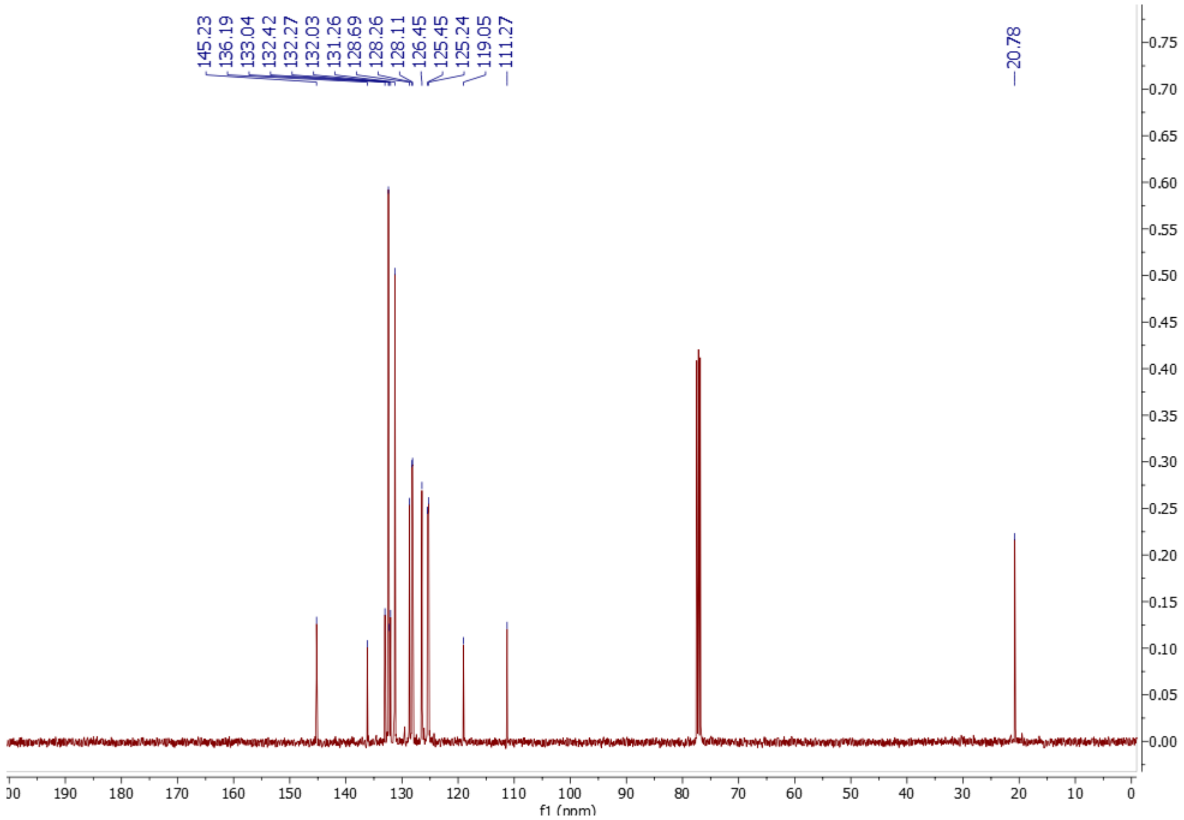

*Supplementary Figure 117. ¹³C NMR of 4-(naphthalen-1-yl)benzonitrile (**2n**) (101 MHz, 20 °C, $CDCl_3$)*

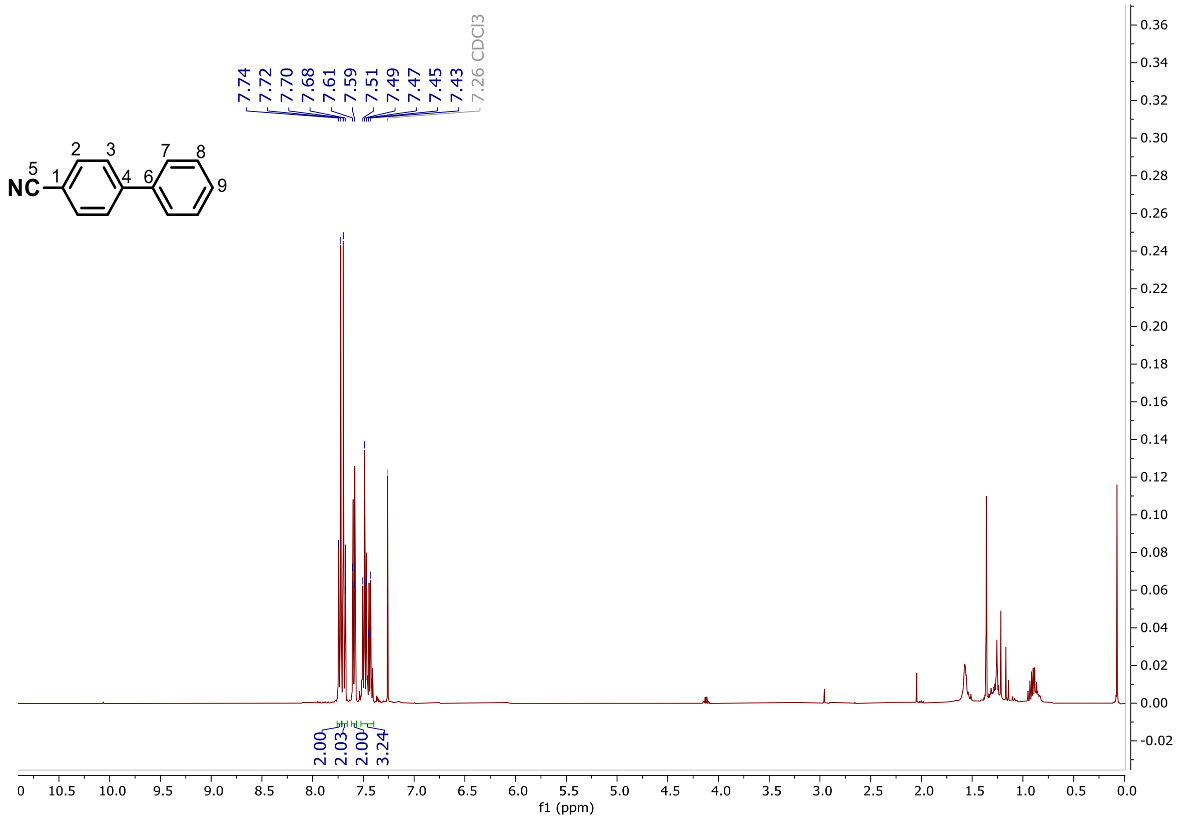

*Supplementary Figure 118. ^{1}H NMR of [1,1'-biphenyl]-4-carbonitrile (**2o**) (400 MHz, 20 °C, $CDCl_3$)*

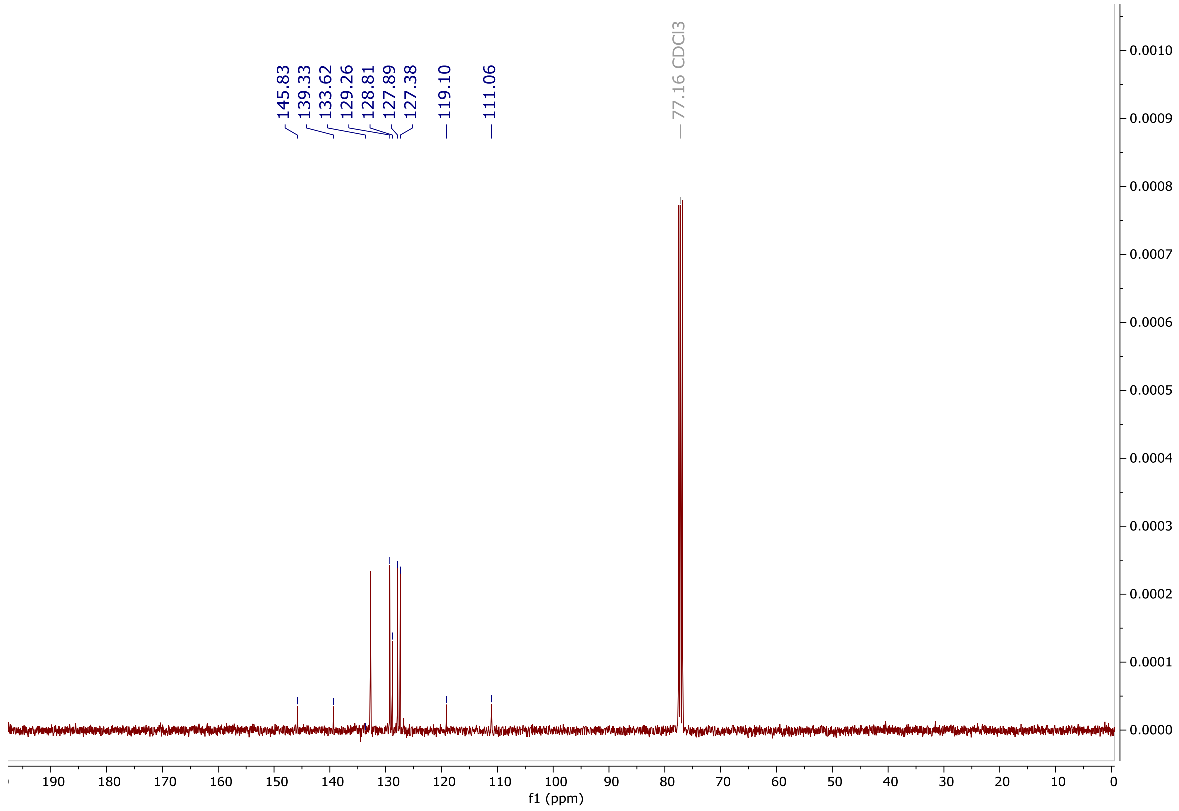

*Supplementary Figure 119. ^{13}C NMR of [1,1'-biphenyl]-4-carbonitrile (**2o**) (101 MHz, 20 °C, $CDCl_3$)*

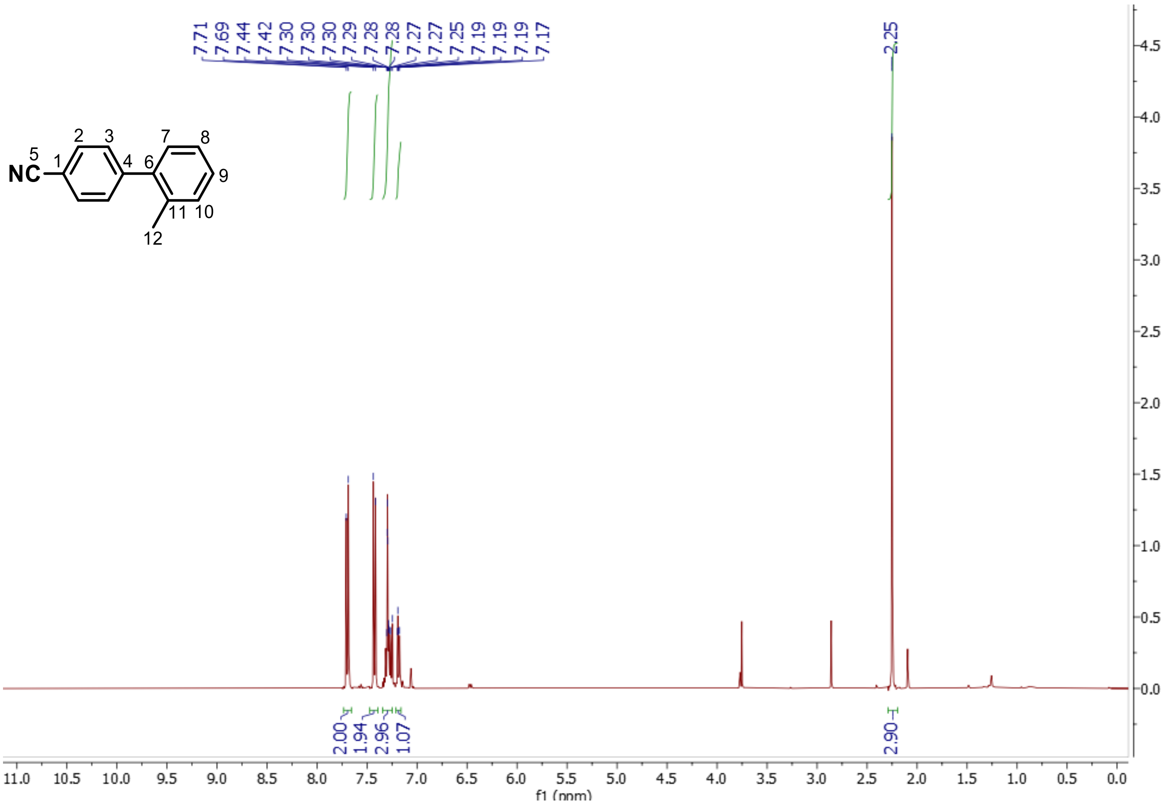

*Supplementary Figure 120. ^{1}H NMR of 2'-methyl-[1,1'-biphenyl]-4-carbonitrile (**2p**) (400 MHz, 20 °C, $CDCl_3$)*

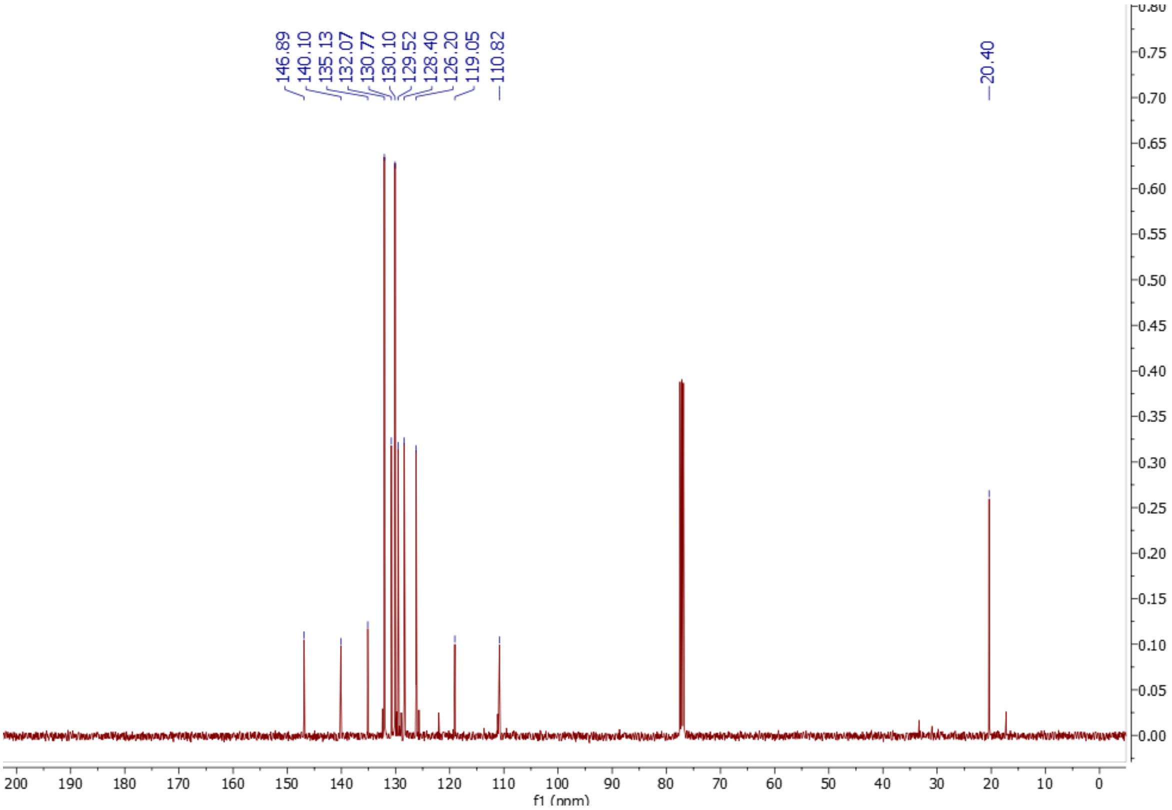

*Supplementary Figure 121. ^{13}C NMR of 2'-methyl-[1,1'-biphenyl]-4-carbonitrile (**2p**) (101 MHz, 20 °C, $CDCl_3$)*

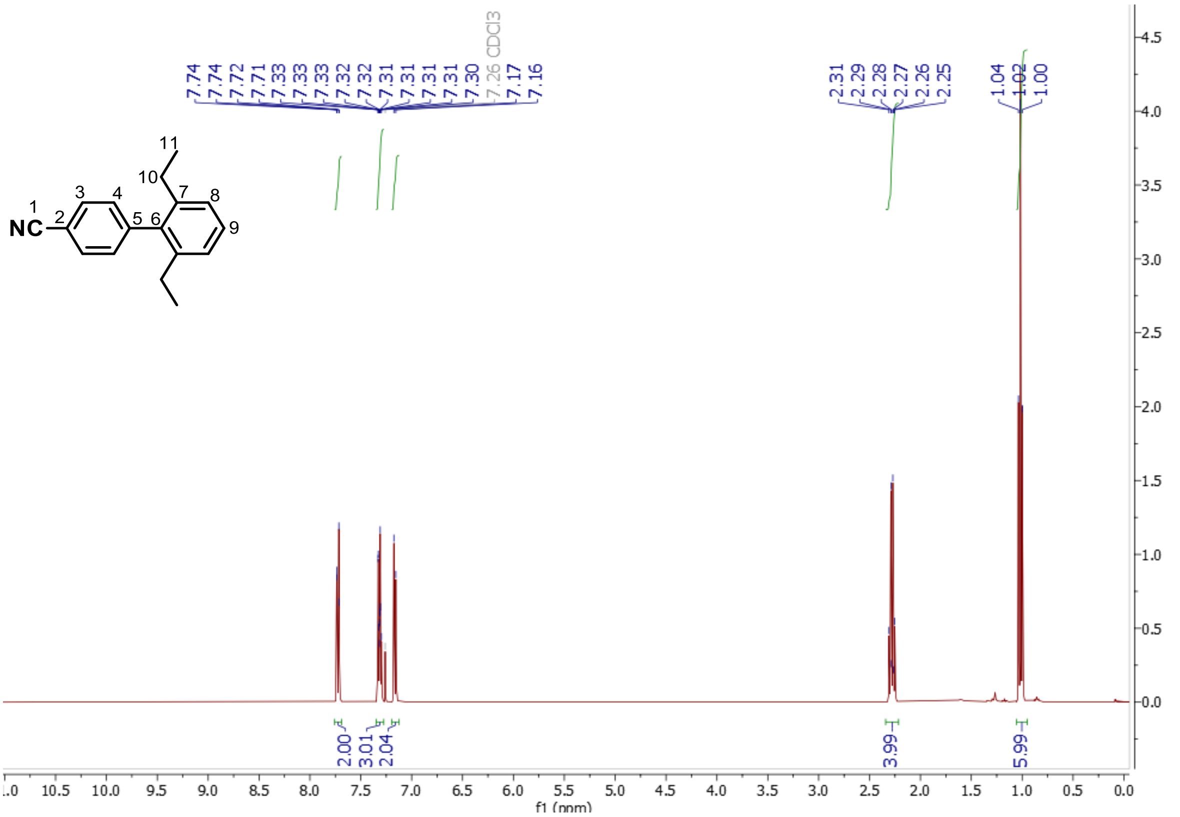

*Supplementary Figure 122. ¹H NMR of 2',6'-diethyl-[1,1'-biphenyl]-4-carbonitrile (**2q**) (400 MHz, 20 °C, CDCl₃)*

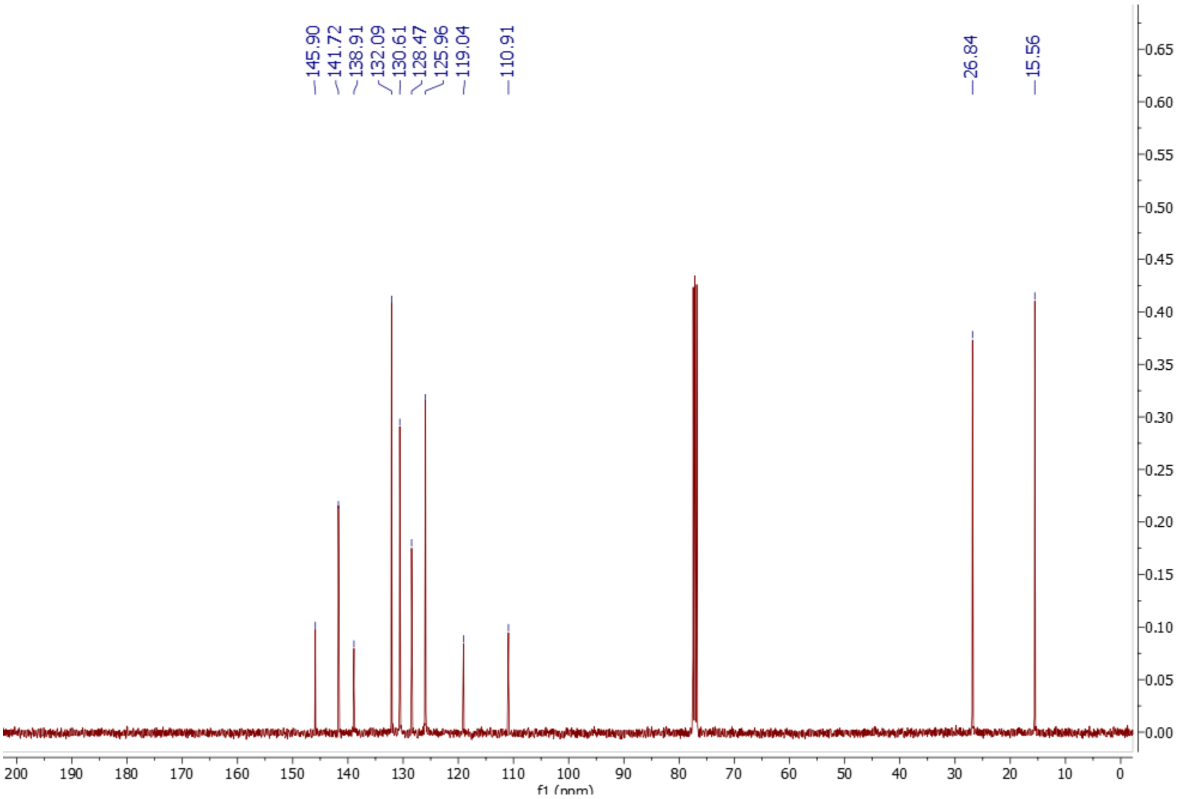

*Supplementary Figure 123. ¹³C NMR of 2',6'-diethyl-[1,1'-biphenyl]-4-carbonitrile (**2q**) (101 MHz, 20 °C, CDCl₃)*

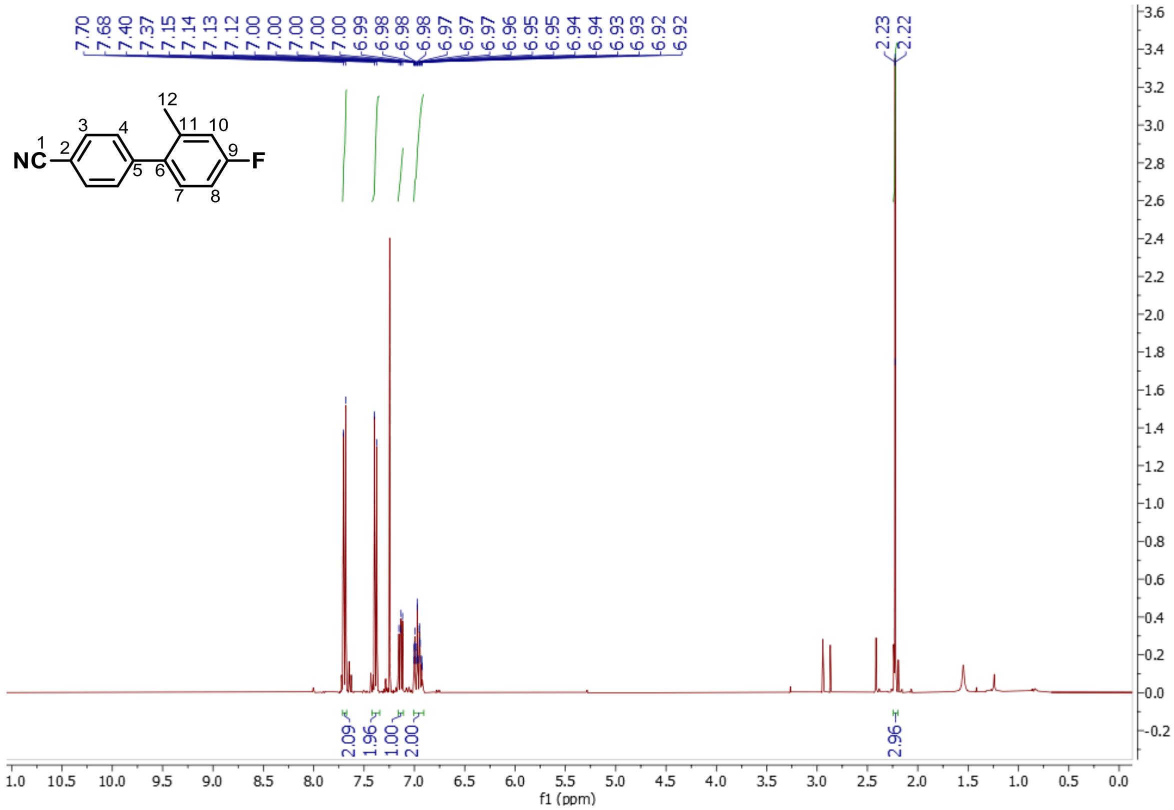

*Supplementary Figure 124. ^{1}H NMR of 4'-fluoro-2'-methyl-[1,1'-biphenyl]-4-carbonitrile (**2r**) (400 MHz, 20 °C, $CDCl_3$)*

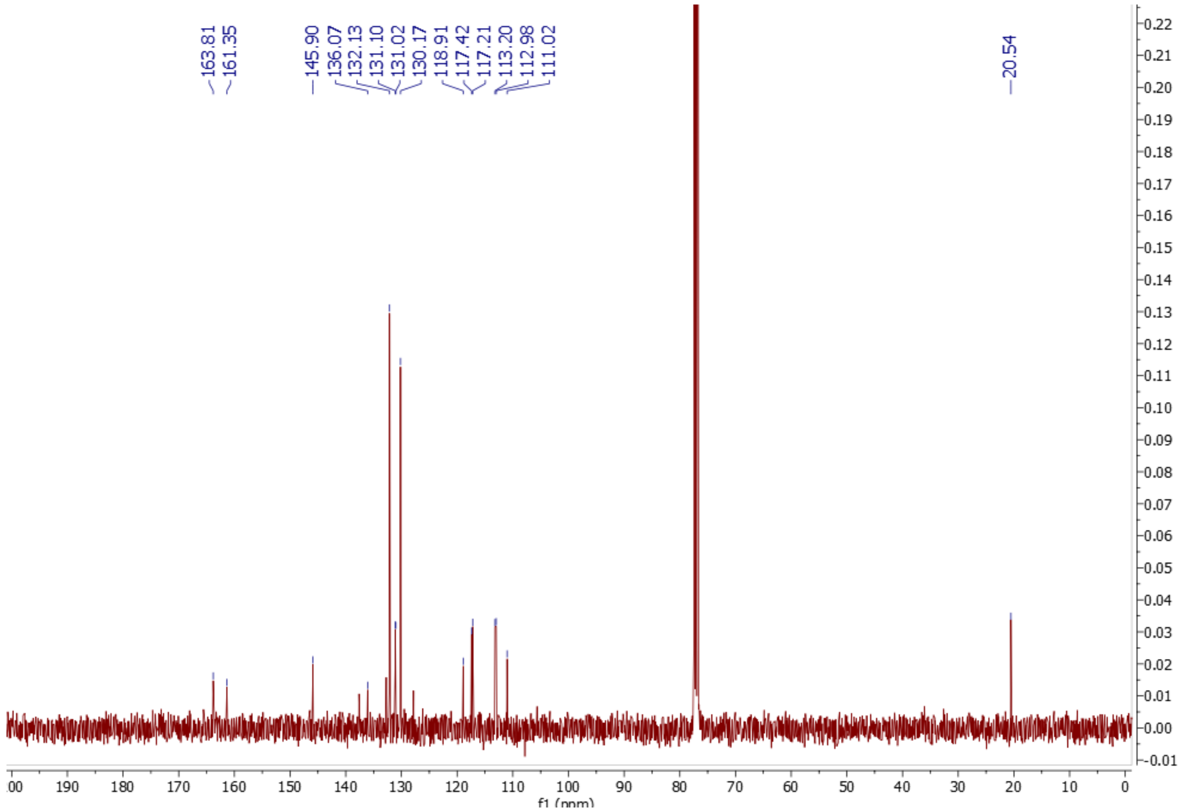

*Supplementary Figure 125. ^{13}C NMR of 4'-fluoro-2'-methyl-[1,1'-biphenyl]-4-carbonitrile (**2r**) (101 MHz, 20 °C, $CDCl_3$)*

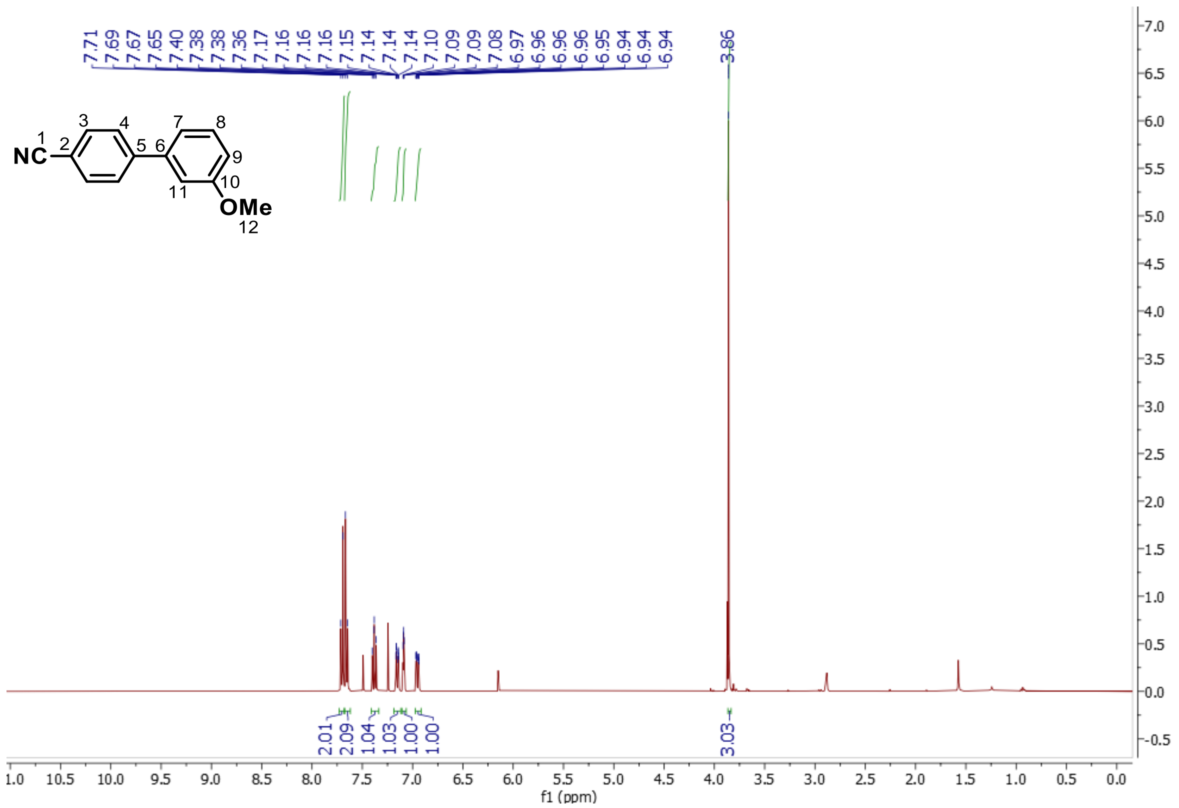

*Supplementary Figure 126. ¹H NMR of 3'-methoxy-[1,1'-biphenyl]-4-carbonitrile (**2t**) (400 MHz, 20 °C, CDCl₃)*

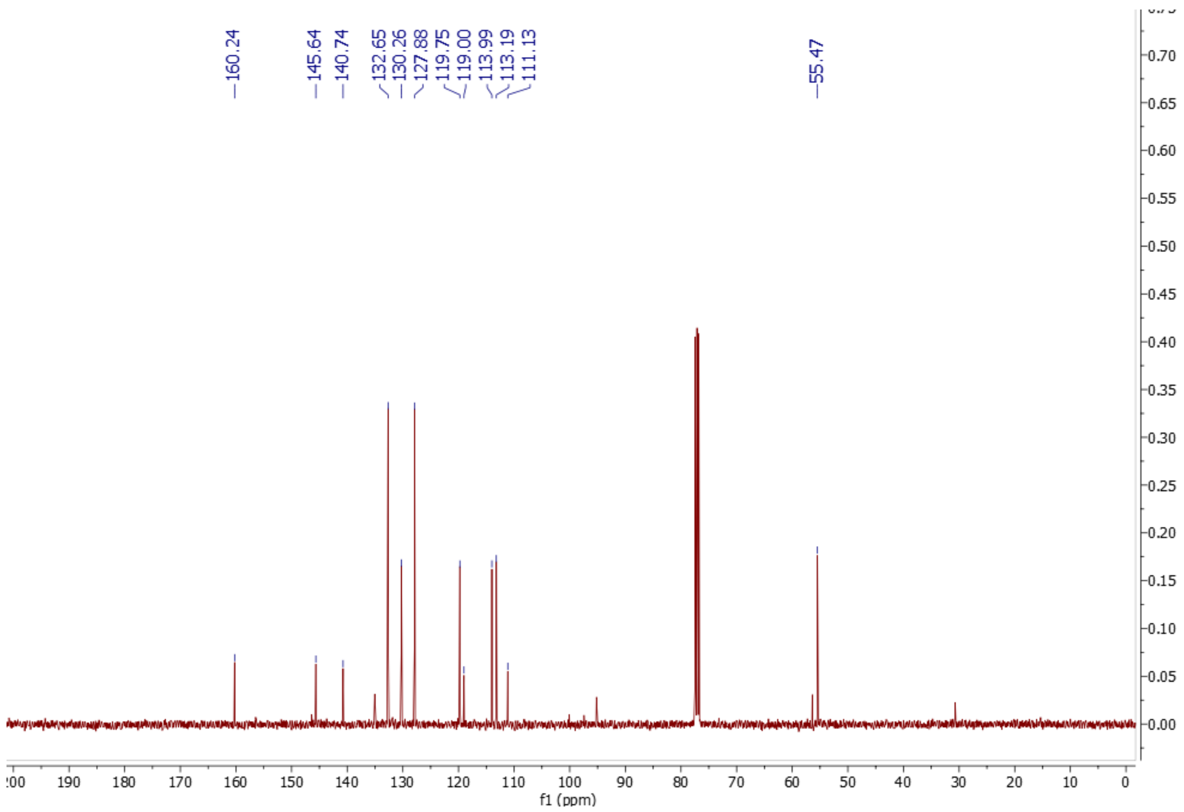

*Supplementary Figure 127. ¹³C NMR of 3'-methoxy-[1,1'-biphenyl]-4-carbonitrile (**2t**) (101 MHz, 20 °C, CDCl₃)*

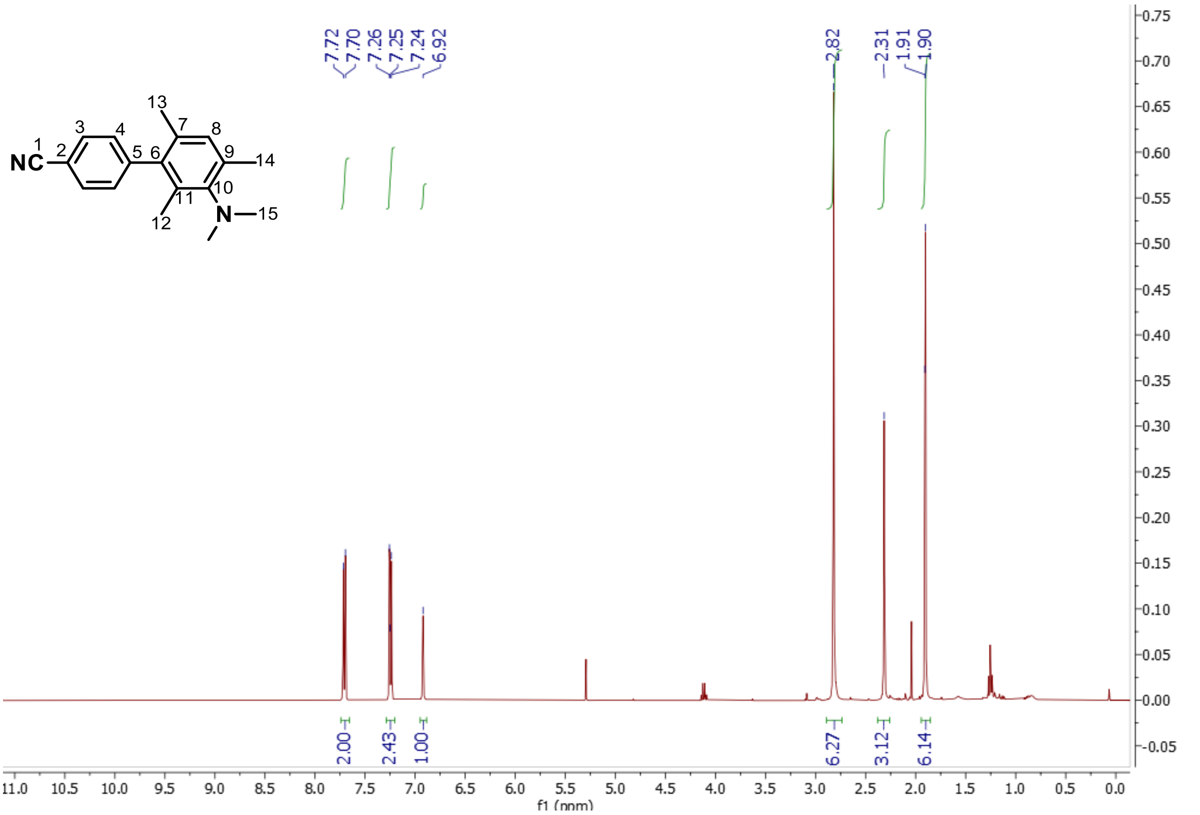

Supplementary Figure 128. ^{1}H NMR of 3'-(dimethylamino)-2',4',6'-trimethyl-[1,1'-biphenyl]-4-carbonitrile (**2v**) (400 MHz, 20 °C, CDCl$_3$)

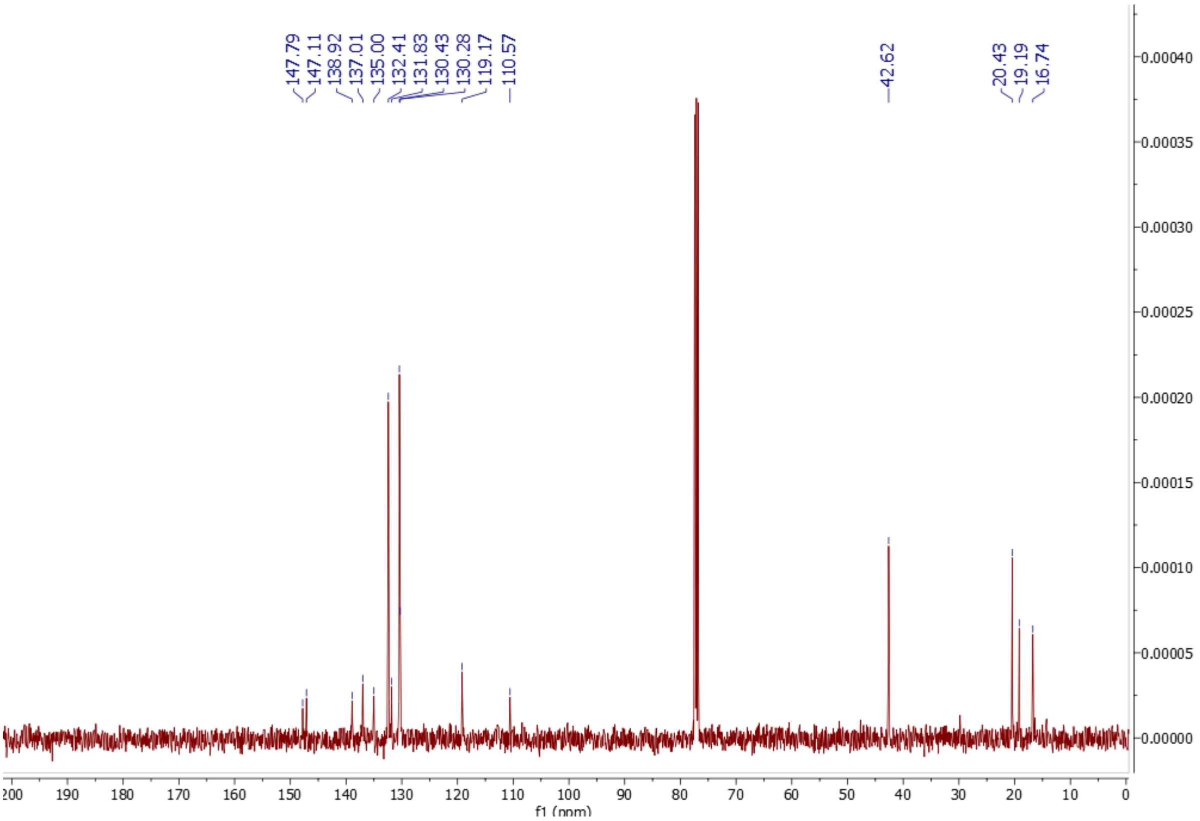

Supplementary Figure 129. ^{13}C NMR of 3'-(dimethylamino)-2',4',6'-trimethyl-[1,1'-biphenyl]-4-carbonitrile (**2v**) (101 MHz, 20 °C, CDCl$_3$)

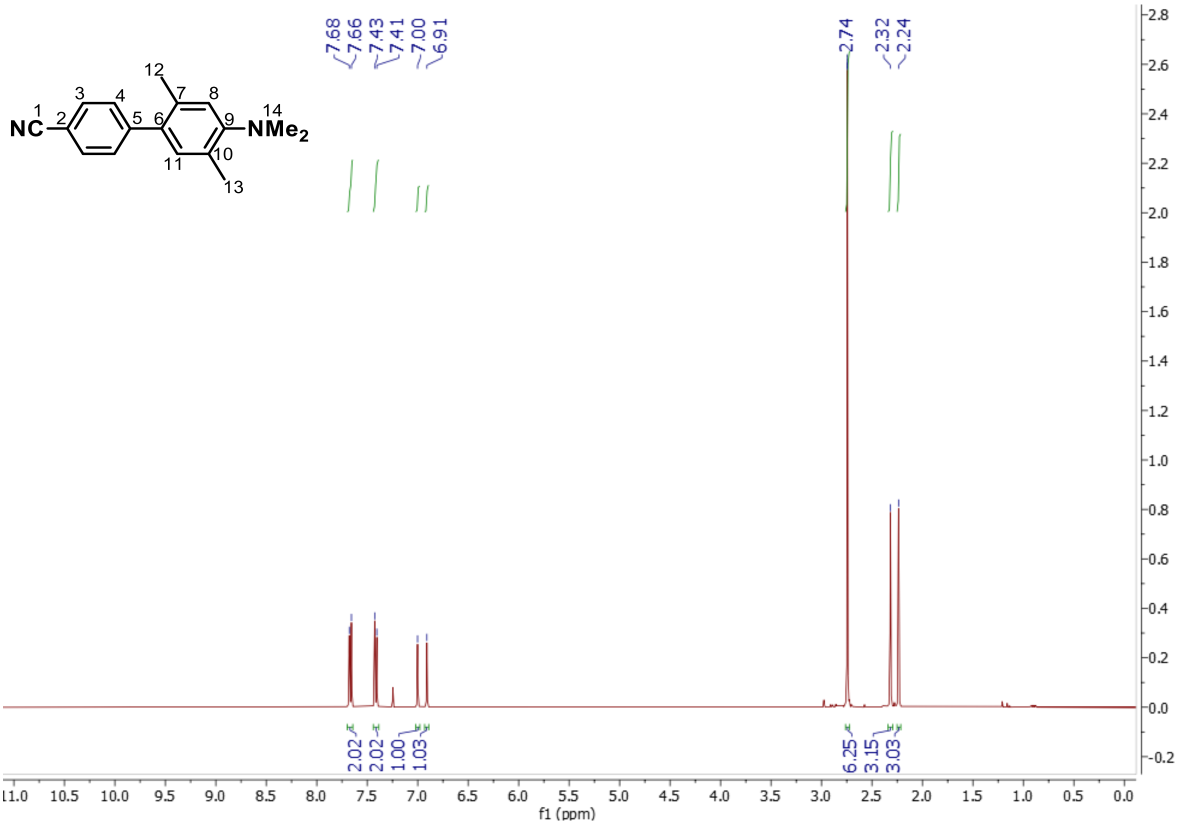

*Supplementary Figure 130. ^{1}H NMR of 4'-(dimethylamino)-2',5'-dimethyl-[1,1'-biphenyl]-4-carbonitrile (**2w**) (400 MHz, 20 °C, $CDCl_3$)*

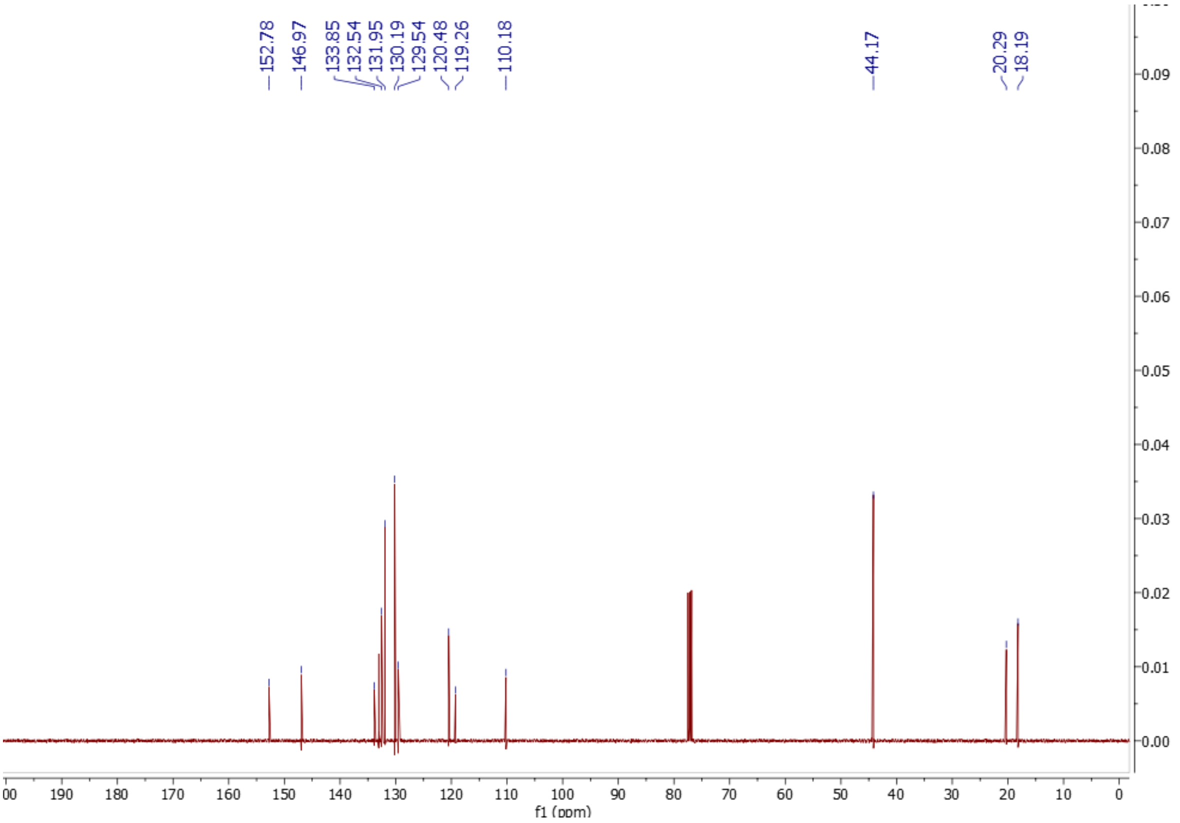

*Supplementary Figure 131. ^{13}C NMR of 4'-(dimethylamino)-2',5'-dimethyl-[1,1'-biphenyl]-4-carbonitrile (**2w**) (101 MHz, 20 °C, $CDCl_3$)*

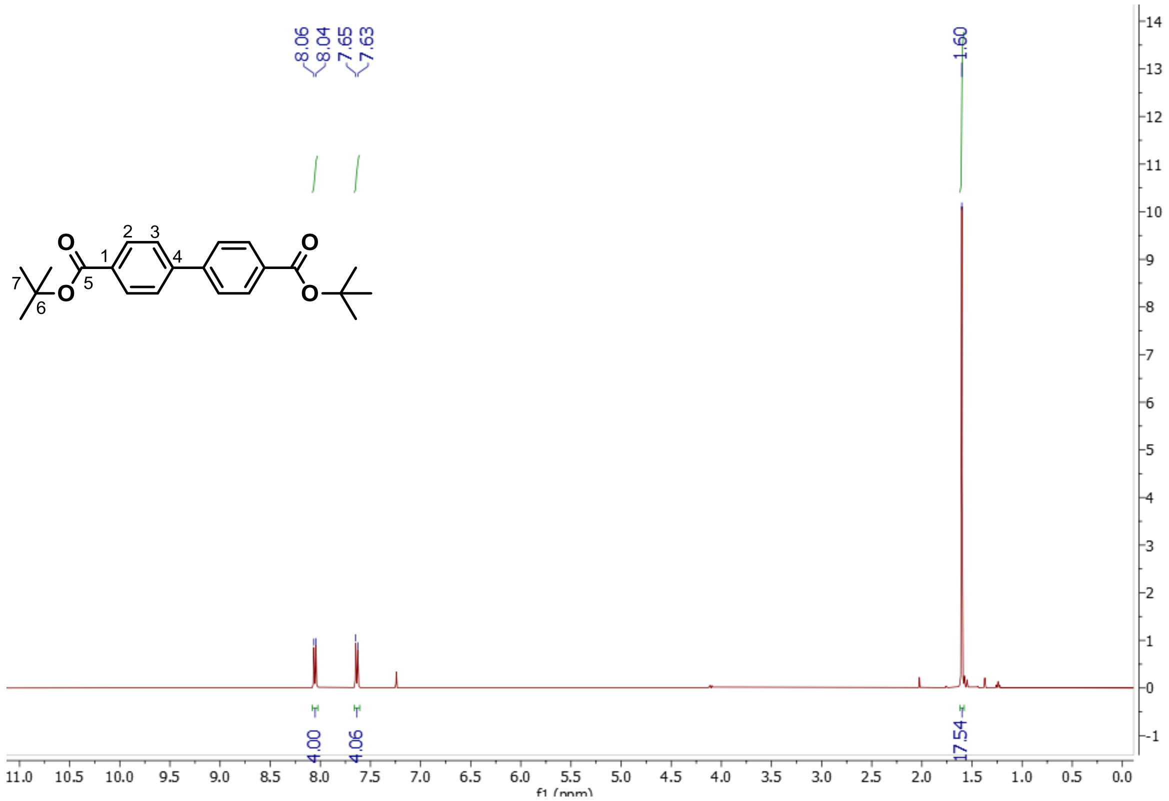

*Supplementary Figure 132. ¹H NMR of di-tert-butyl [1,1'-biphenyl]-4,4'-dicarboxylate (**2x**) (400 MHz, 20 °C, $CDCl_3$)*

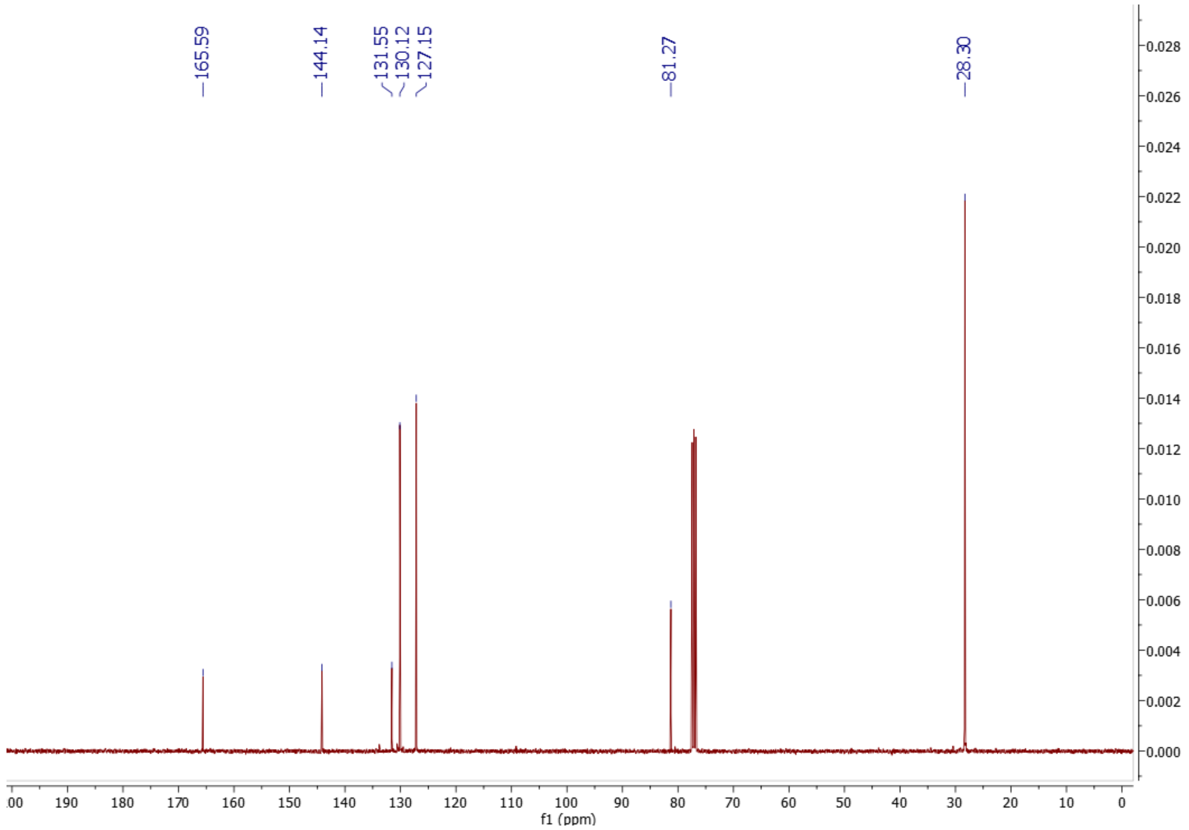

*Supplementary Figure 133. ¹³C NMR of di-tert-butyl [1,1'-biphenyl]-4,4'-dicarboxylate (**2x**) (101 MHz, 20 °C, $CDCl_3$)*

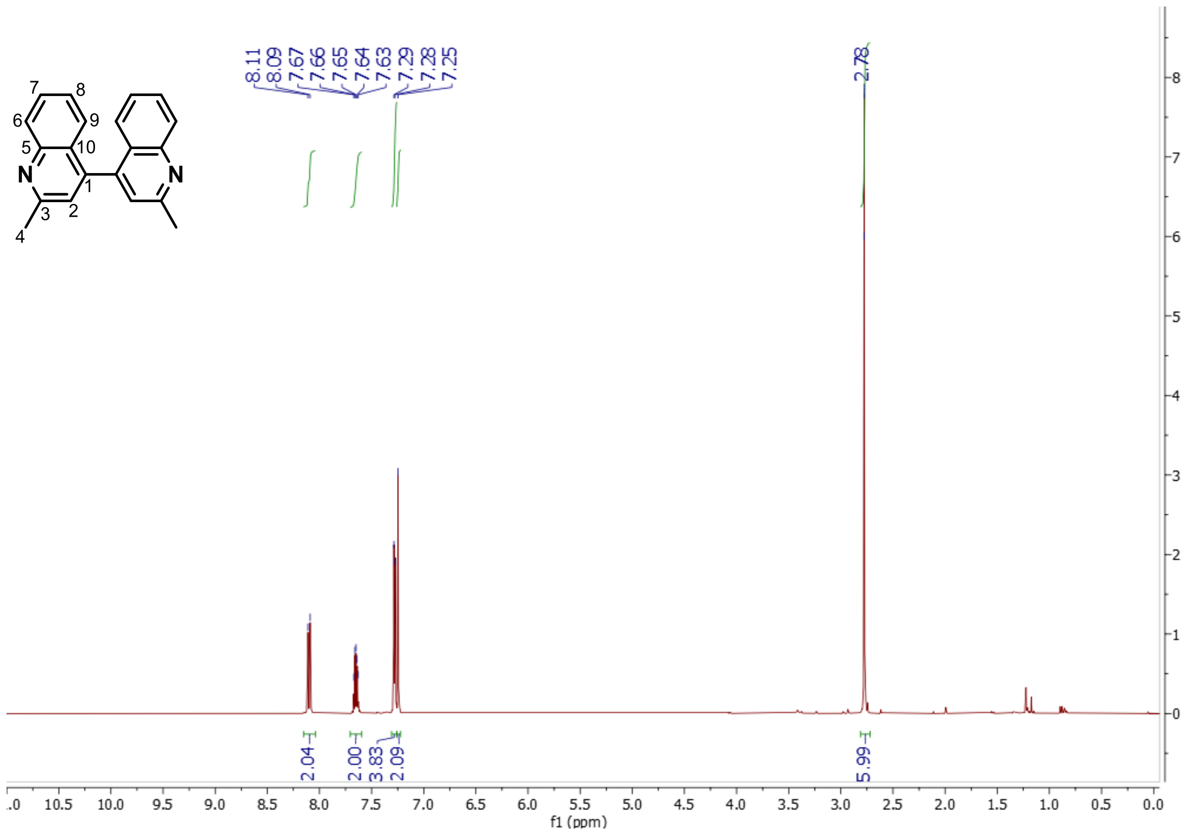

*Supplementary Figure 134. ^{1}H NMR of 2,2'-dimethyl-4,4'-biquinoline (**2y**) (400 MHz, 20 °C, $CDCl_3$)*

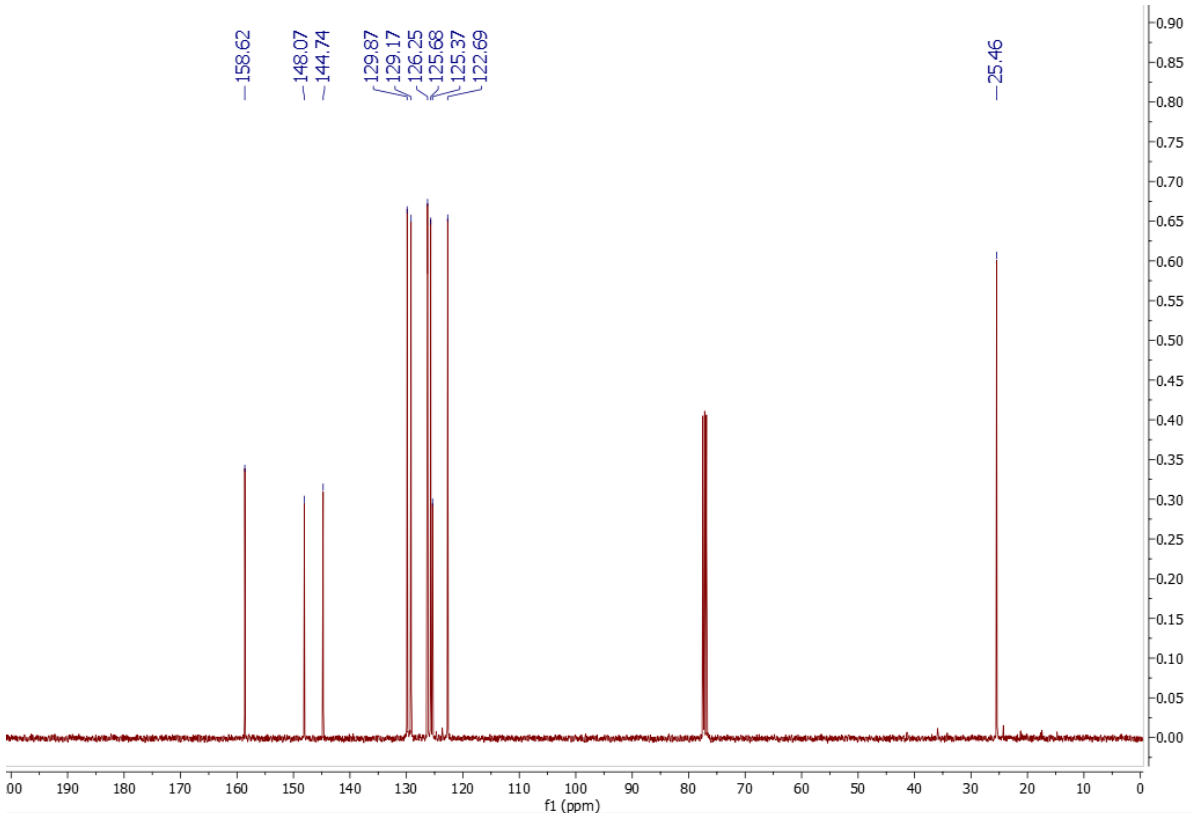

*Supplementary Figure 135. ^{13}C NMR of 2,2'-dimethyl-4,4'-biquinoline (**2y**) (101 MHz, 20 °C, $CDCl_3$)*

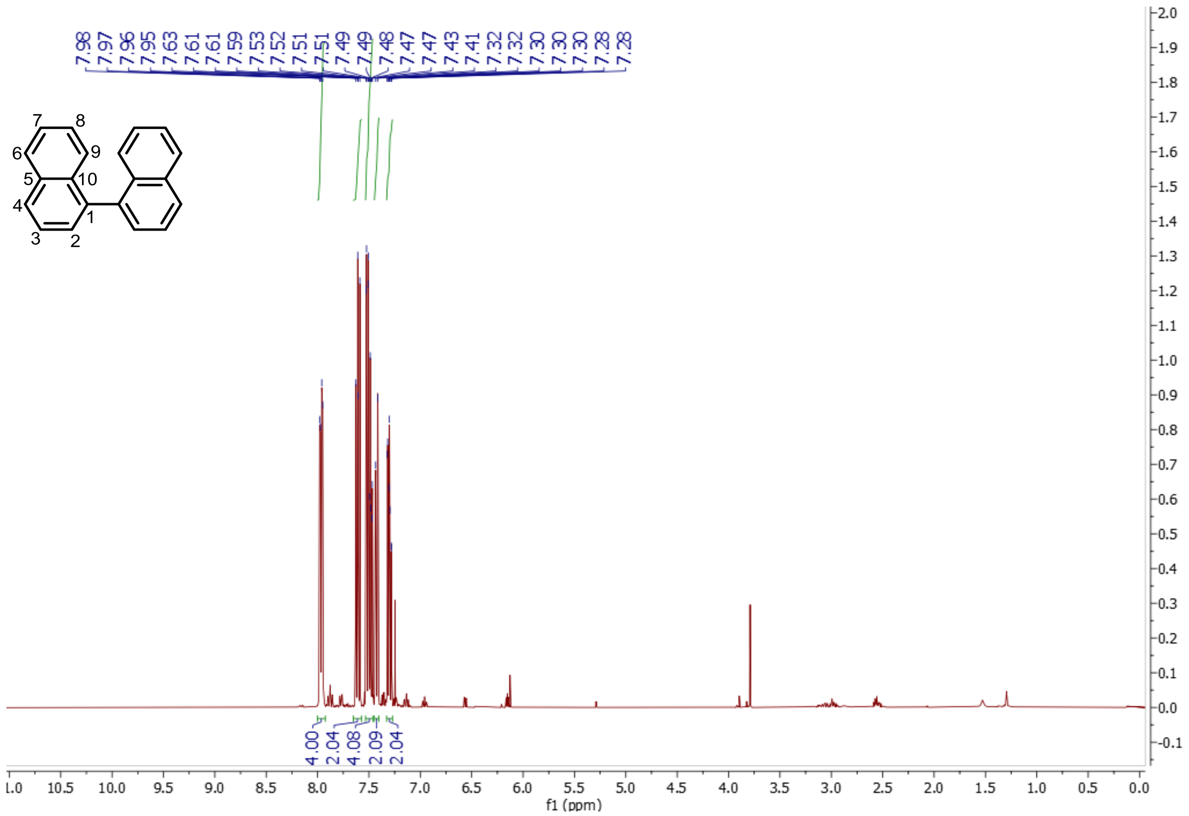

*Supplementary Figure 136. ^{1}H NMR of 1,1'-binaphthalene (**2z**) (400 MHz, 20 °C, $CDCl_3$)*

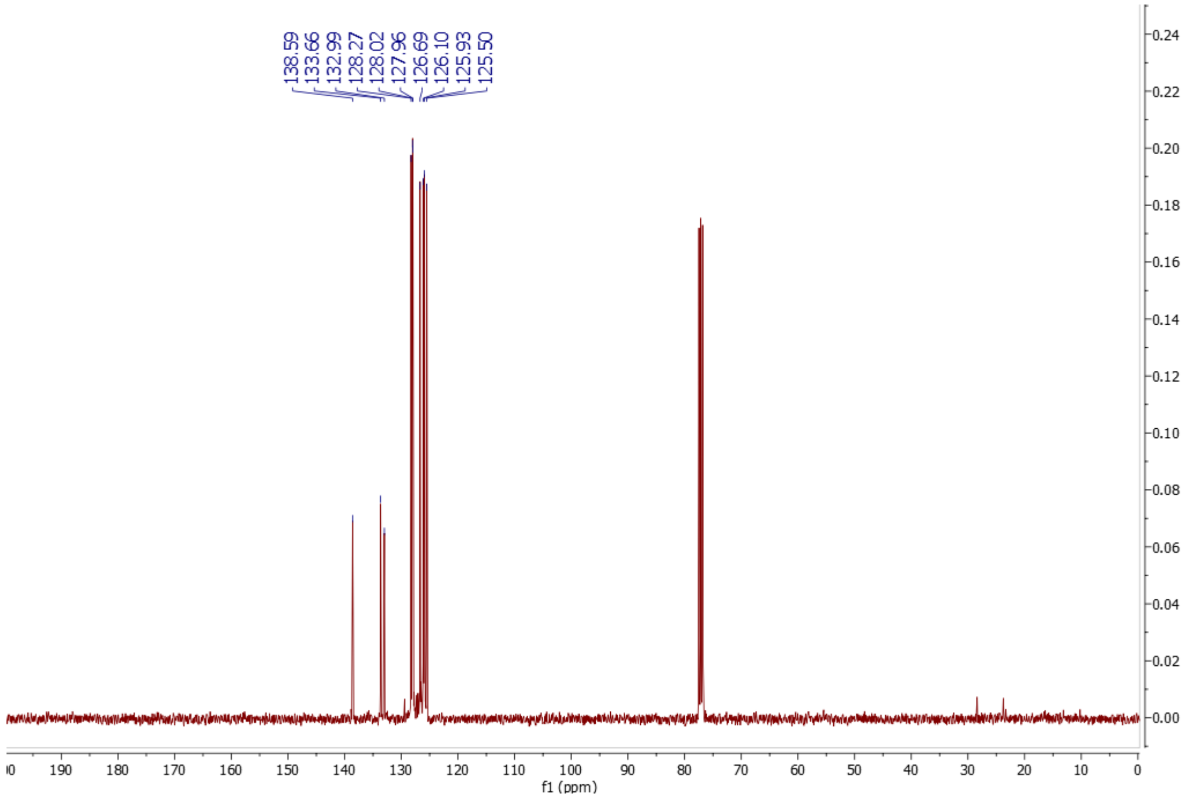

*Supplementary Figure 137. ^{13}C NMR of 1,1'-binaphthalene (**2z**) (101 MHz, 20 °C, $CDCl_3$)*

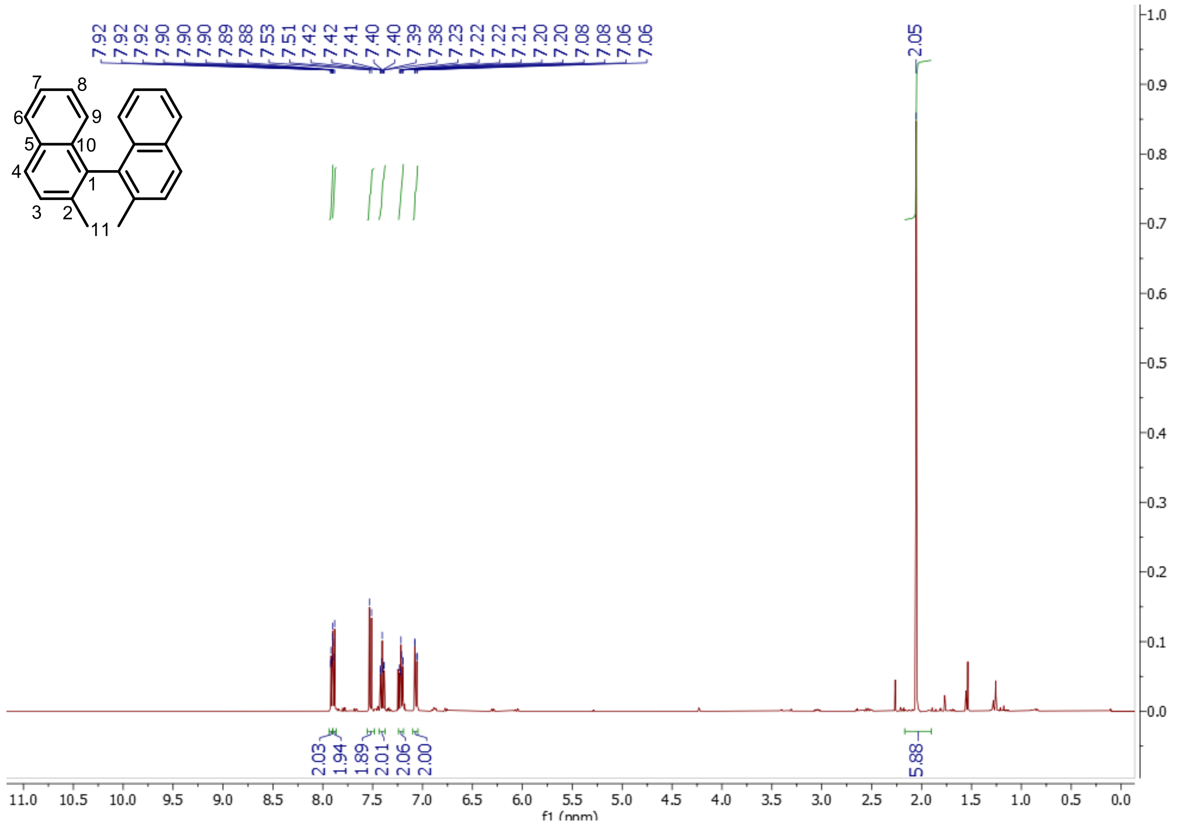

*Supplementary Figure 138. ¹H NMR of 2,2'-dimethyl-1,1'-binaphthalene (**2aa**) (400 MHz, 20 °C, CDCl₃)*

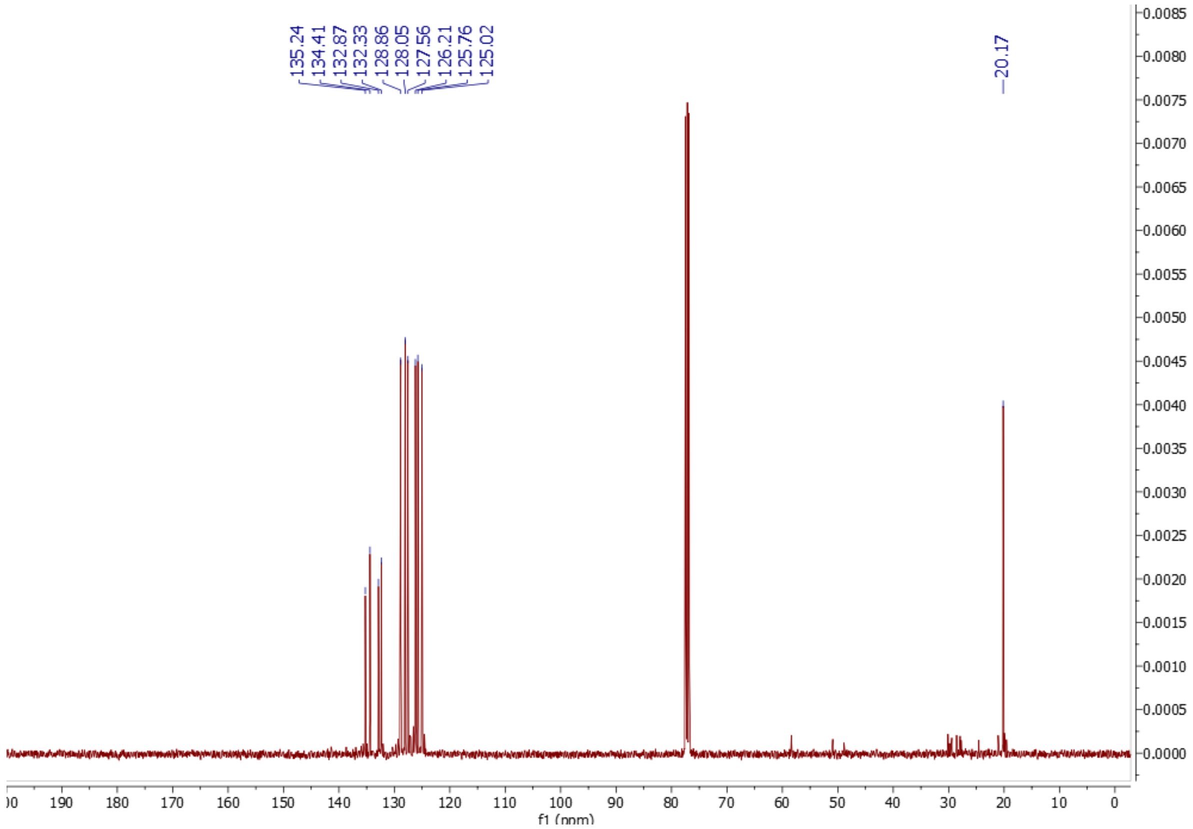

*Supplementary Figure 139. ¹³C NMR of 2,2'-dimethyl-1,1'-binaphthalene (**2aa**) (101 MHz, 20 °C, CDCl₃)*

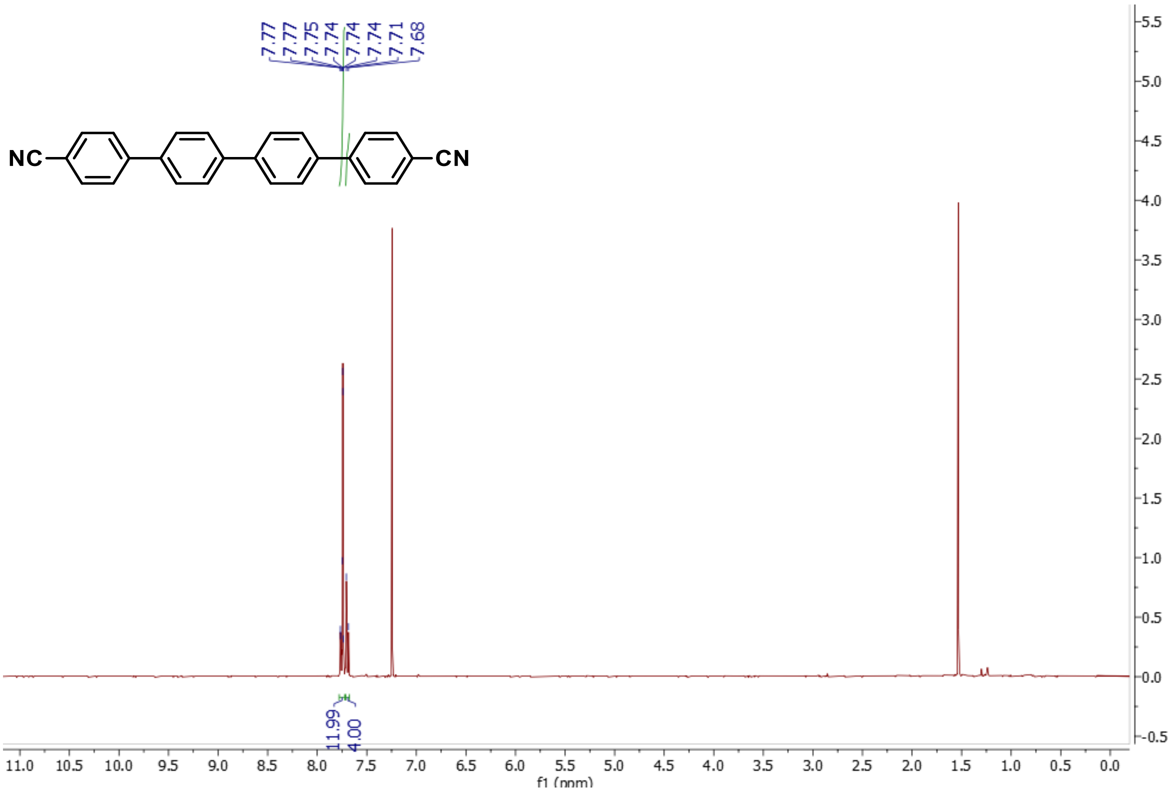

*Supplementary Figure 140. ^{1}H NMR of [1,1':4',1'':4'',1'''-quaterphenyl]-4,4'''-dicarbonitrile (**2ab**) (400 MHz, 20 °C, $CDCl_3$)*

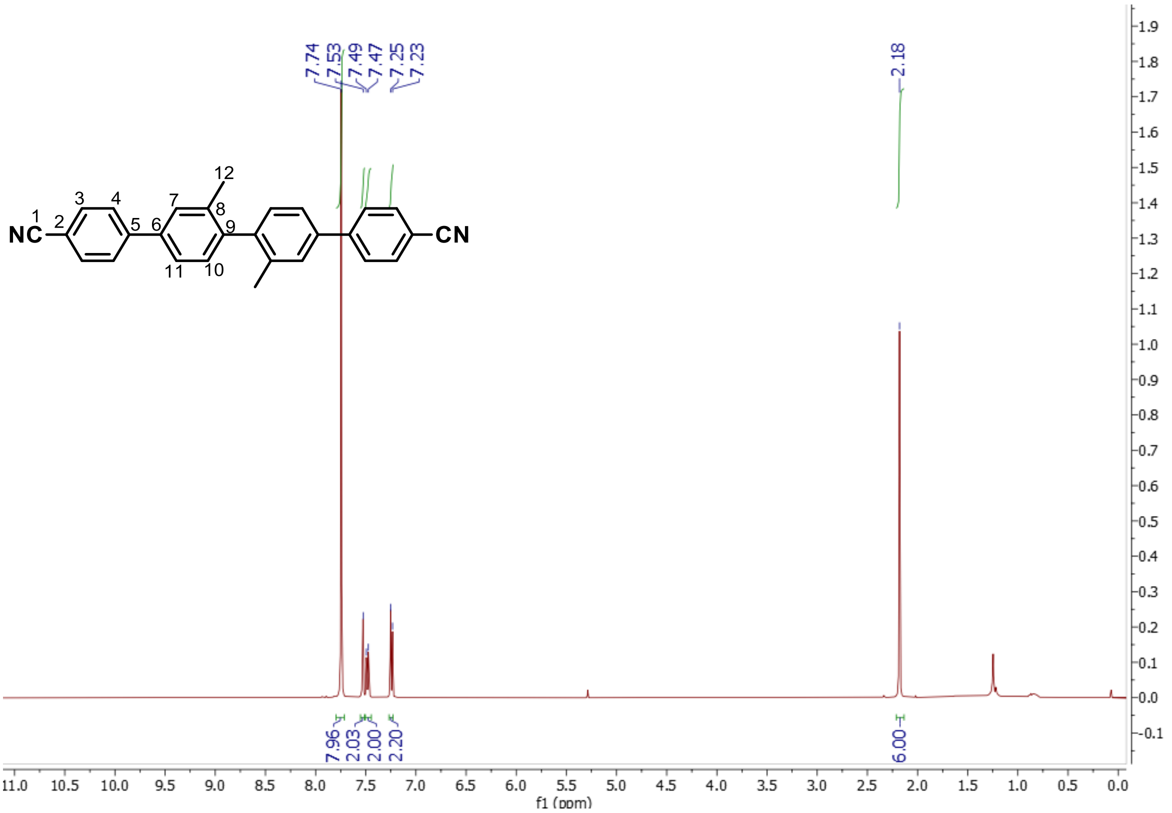

*Supplementary Figure 141. ¹H NMR of 2'',3'-dimethyl-[1,1':4',1'':4'',1'''-quaterphenyl]-4,4'''-dicarbonitrile (**2ac**) (400 MHz, 20 °C, $CDCl_3$)*

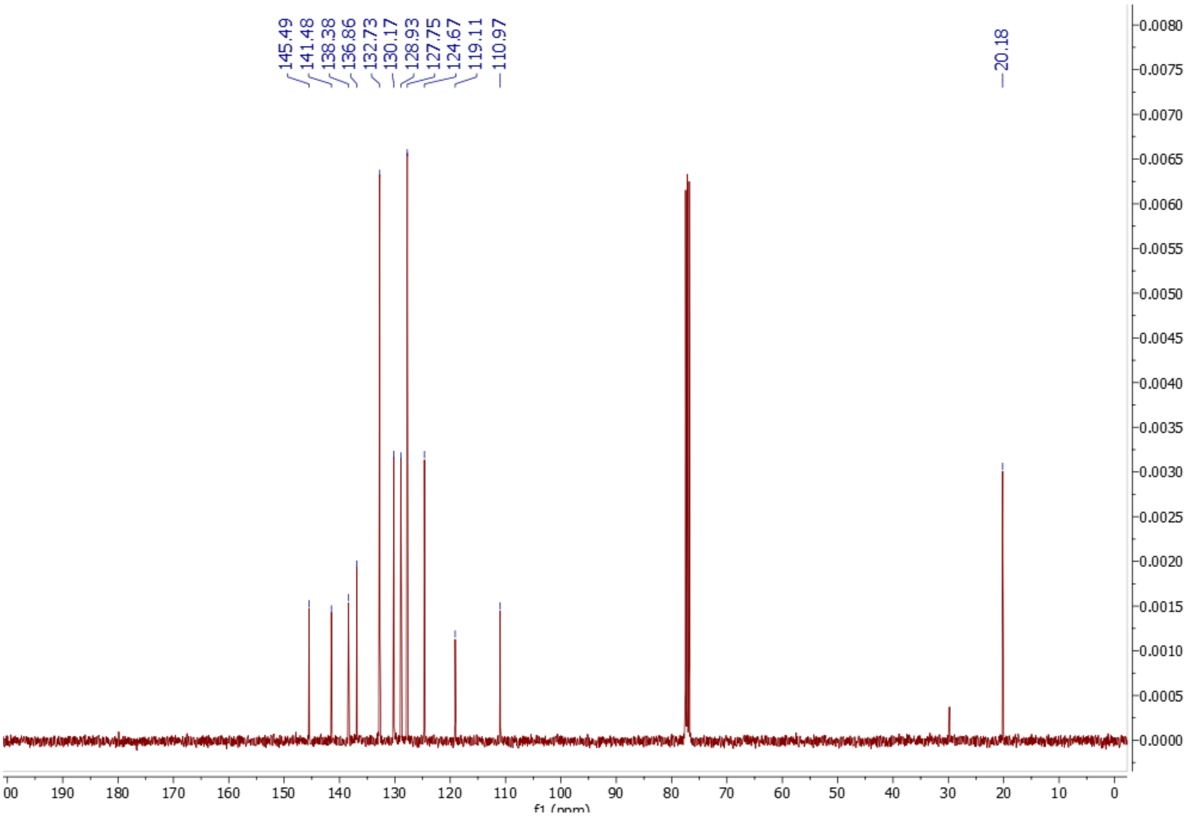

*Supplementary Figure 142. ¹³C NMR of 2'',3'-dimethyl-[1,1':4',1'':4'',1'''-quaterphenyl]-4,4'''-dicarbonitrile (**2ac**) (101 MHz, $CDCl_3$)*

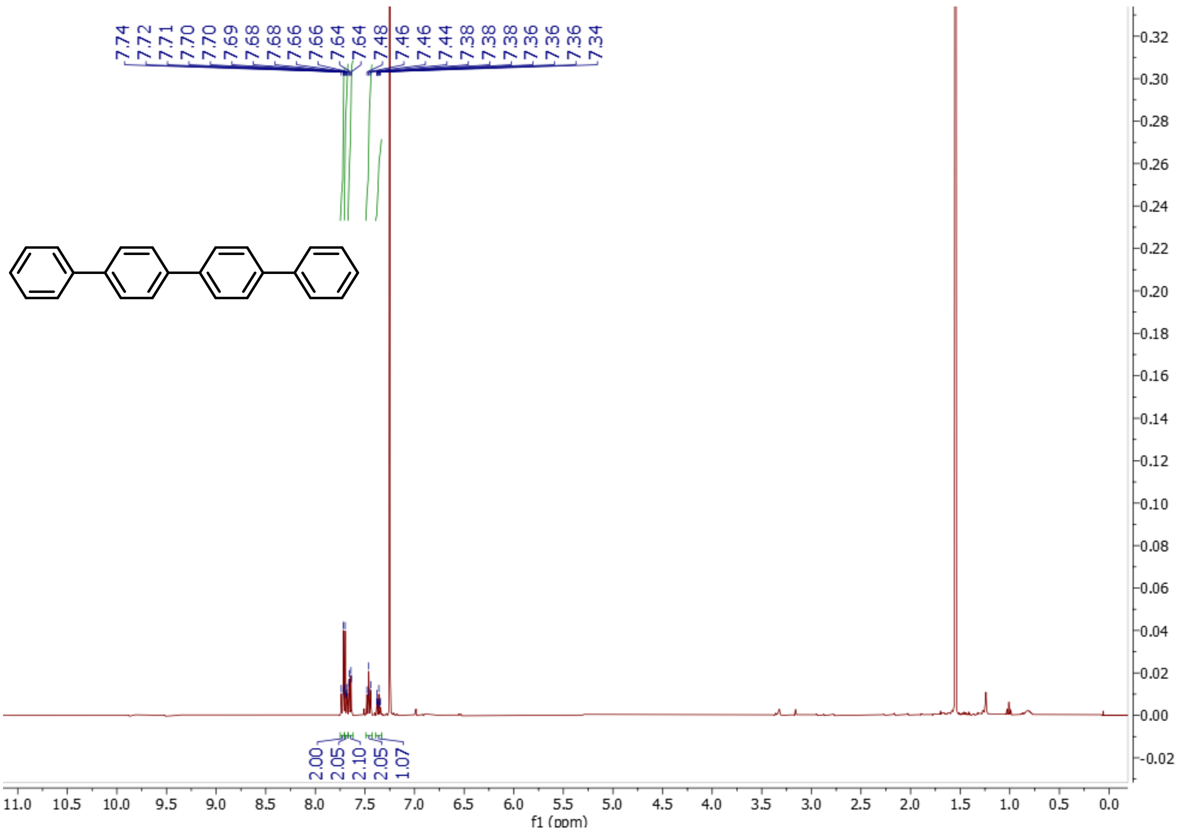

*Supplementary Figure 143. ¹H NMR of 1,1':4',1'':4'',1'''-quaterphenyl (**2ad**) (400 MHz, 20 °C, CDCl₃)*

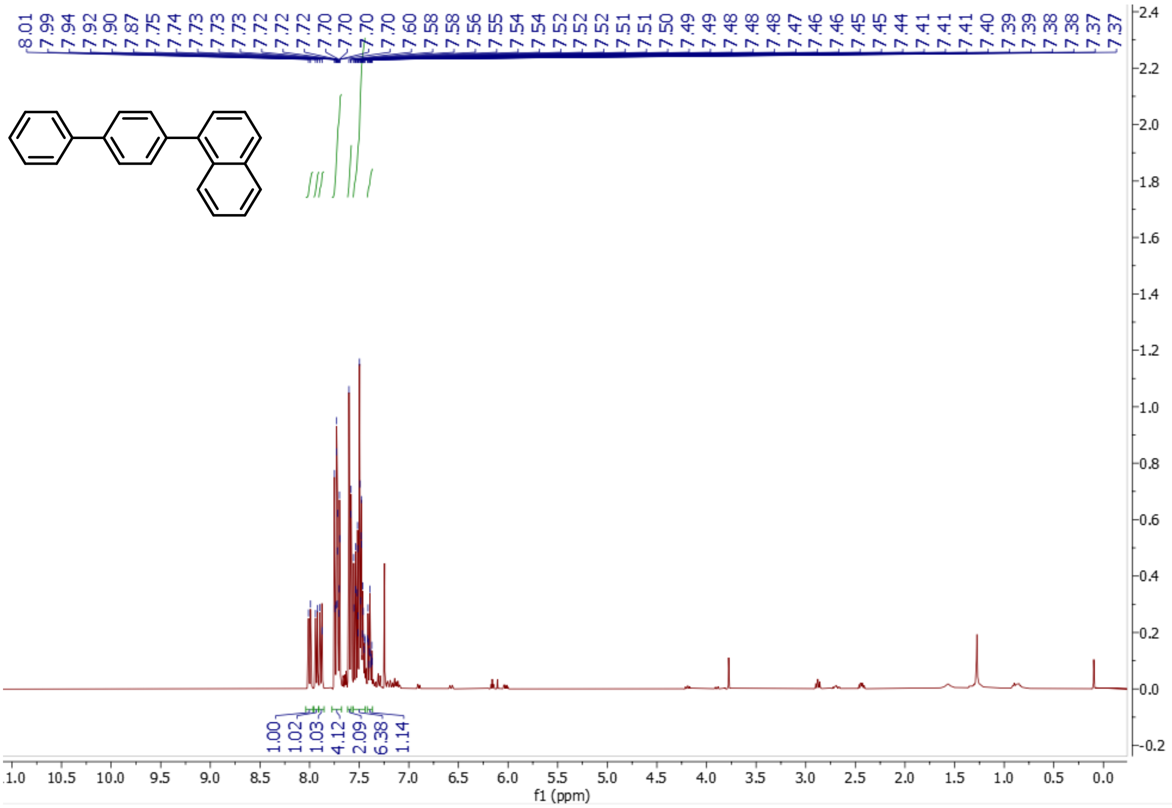

*Supplementary Figure 144. ¹H NMR of 1-([1,1'-biphenyl]-4-yl)naphthalene (**2ae**) (400 MHz, 20 °C, CDCl₃)*

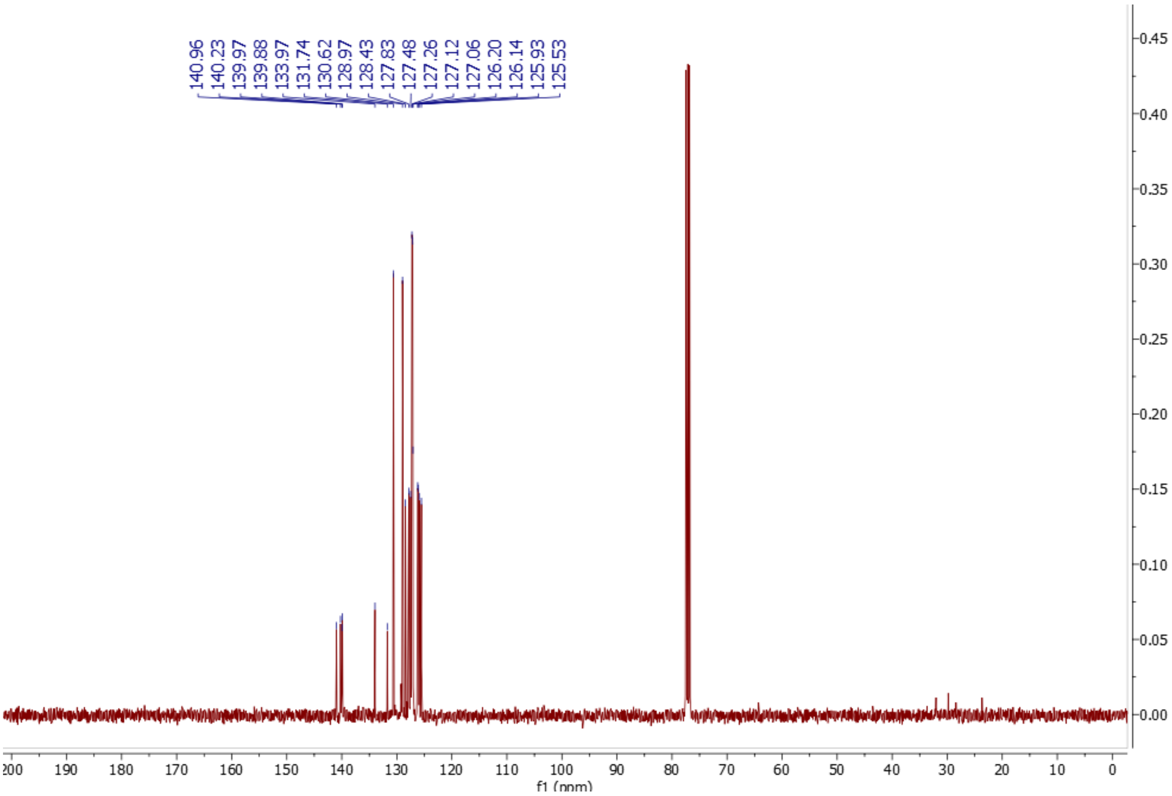

*Supplementary Figure 145. ¹³C NMR of 1-([1,1'-biphenyl]-4-yl)naphthalene (**2ae**) (101 MHz, 20 °C, CDCl₃)*

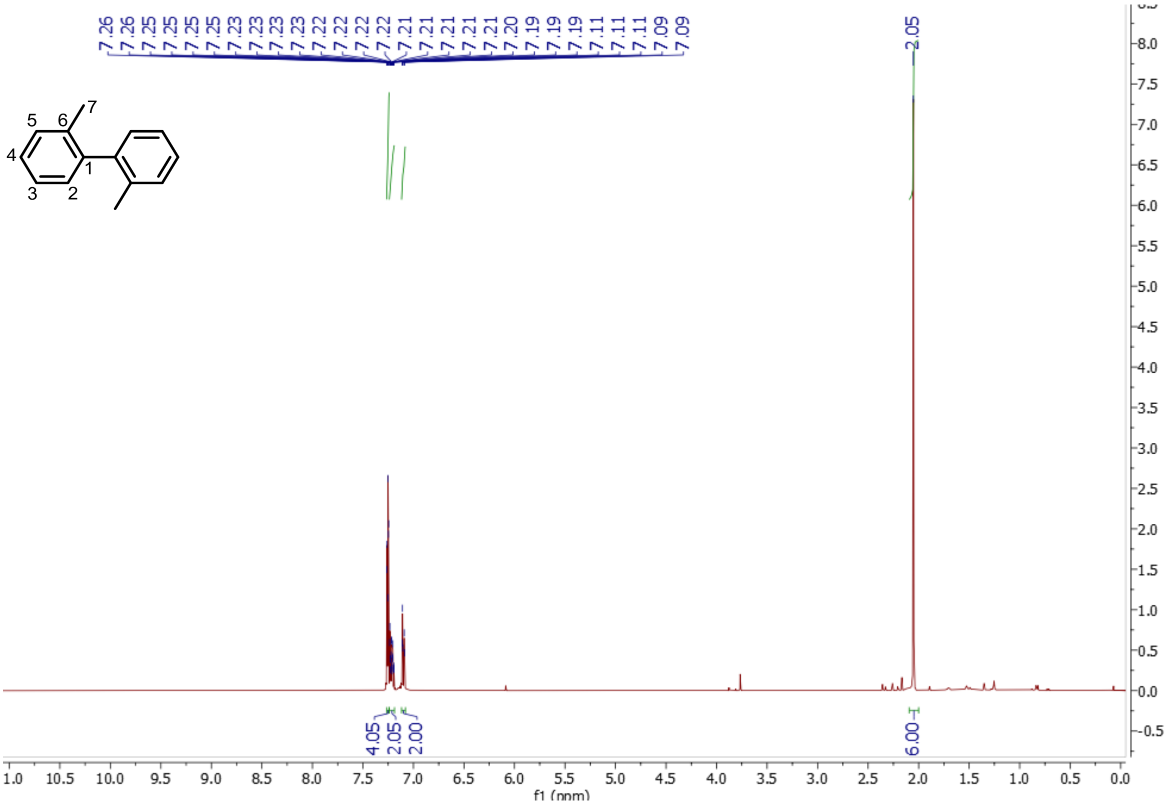

Supplementary Figure 146. ^{1}H NMR of 2,2'-dimethyl-1,1'-biphenyl (**2af**) (400 MHz, 20 °C, $CDCl_3$)

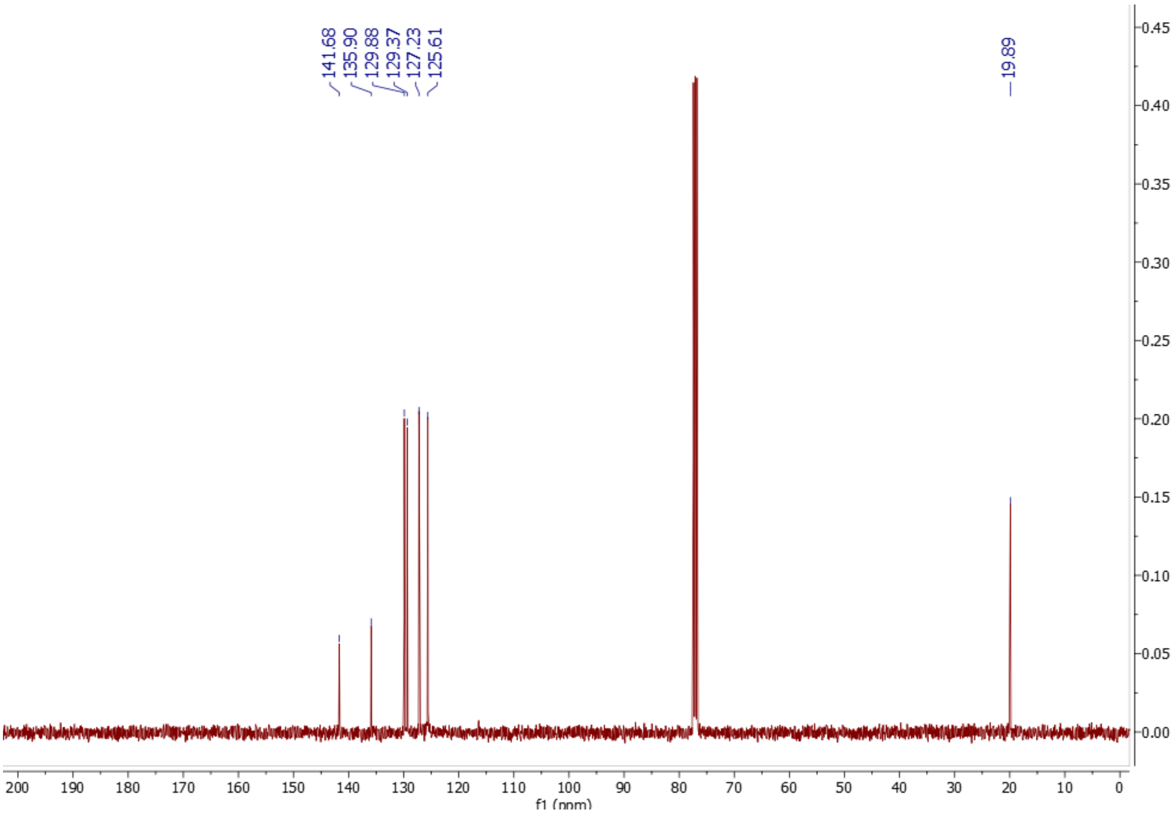

Supplementary Figure 147. ^{13}C NMR of 2,2'-dimethyl-1,1'-biphenyl (**2af**) (101 MHz, 20 °C, $CDCl_3$)

12.3 NMR spectra of other linkers

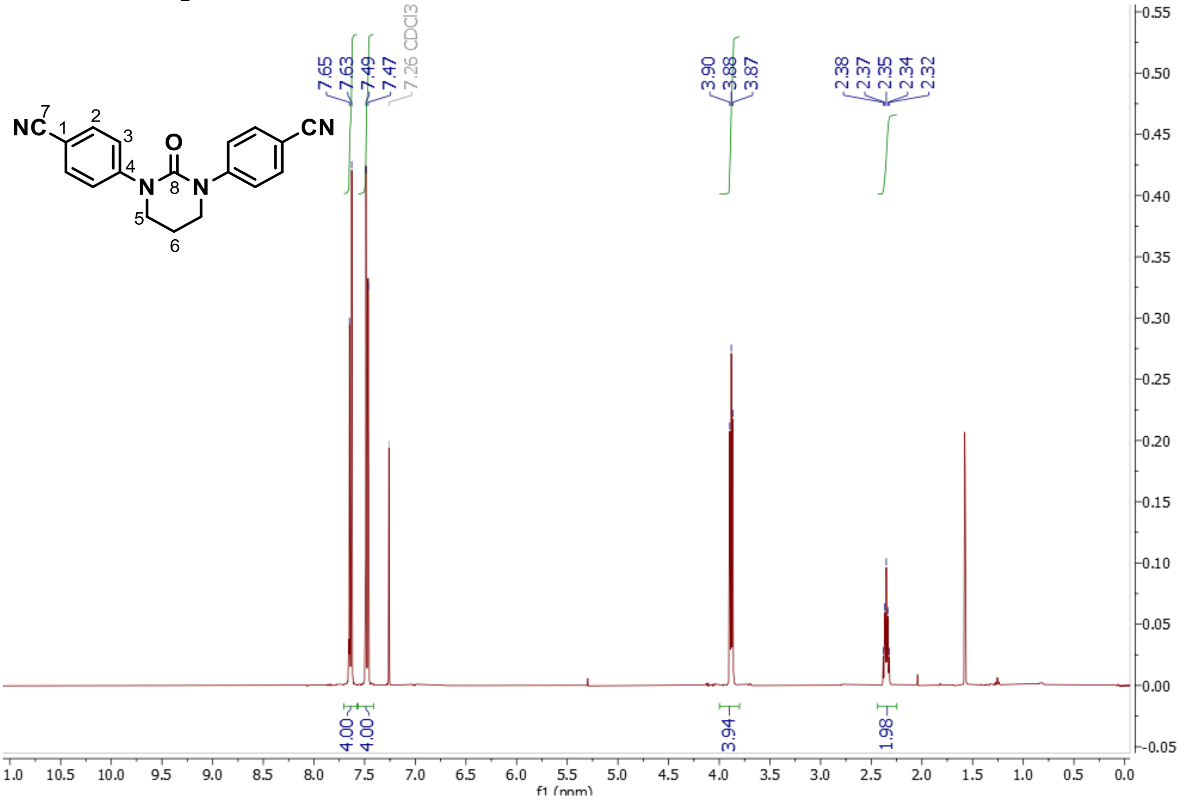

Supplementary Figure 148. *^{1}H NMR of 4,4'-(2-oxodihydropyrimidine-1,3(2H,4H)-diyl)dibenzonitrile (**4b**) (400 MHz, 20 °C, $CDCl_3$)*

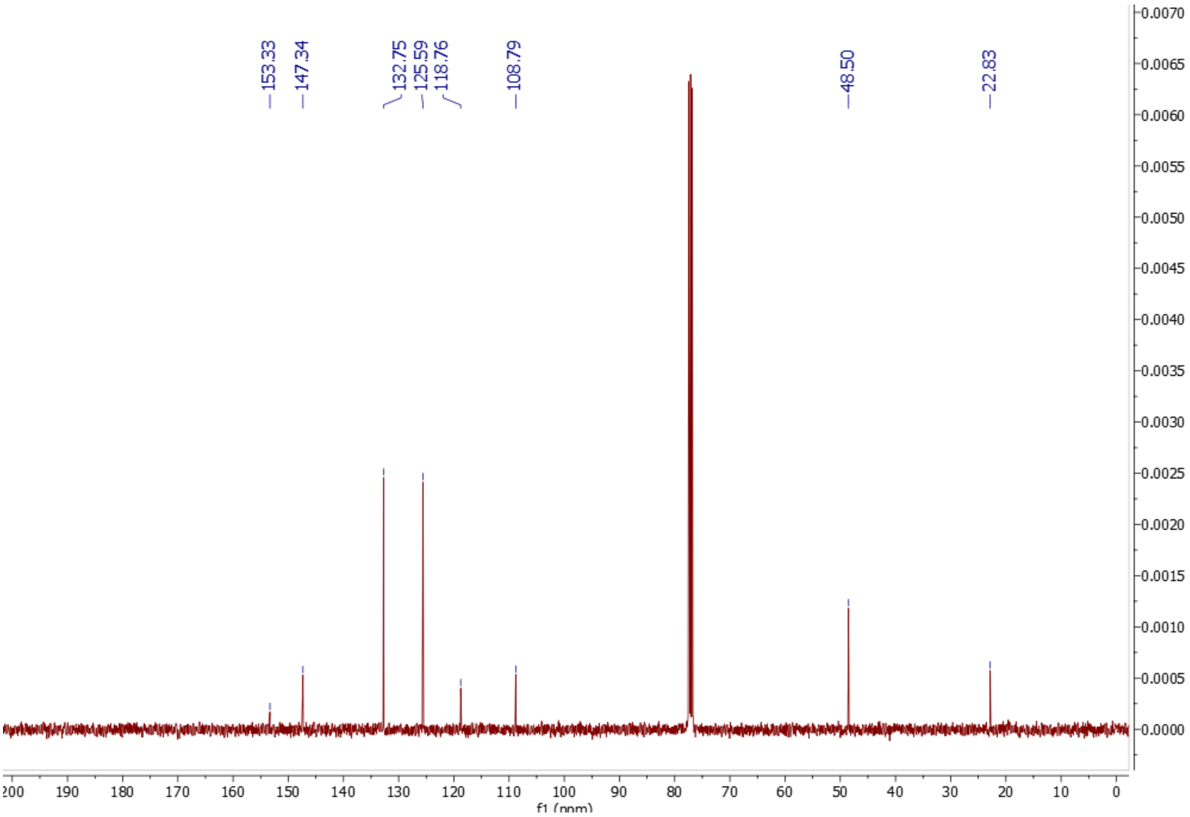

Supplementary Figure 149. *^{13}C NMR of 4,4'-(2-oxodihydropyrimidine-1,3(2H,4H)-diyl)dibenzonitrile (**4b**) (101 MHz, 20 °C, $CDCl_3$)*

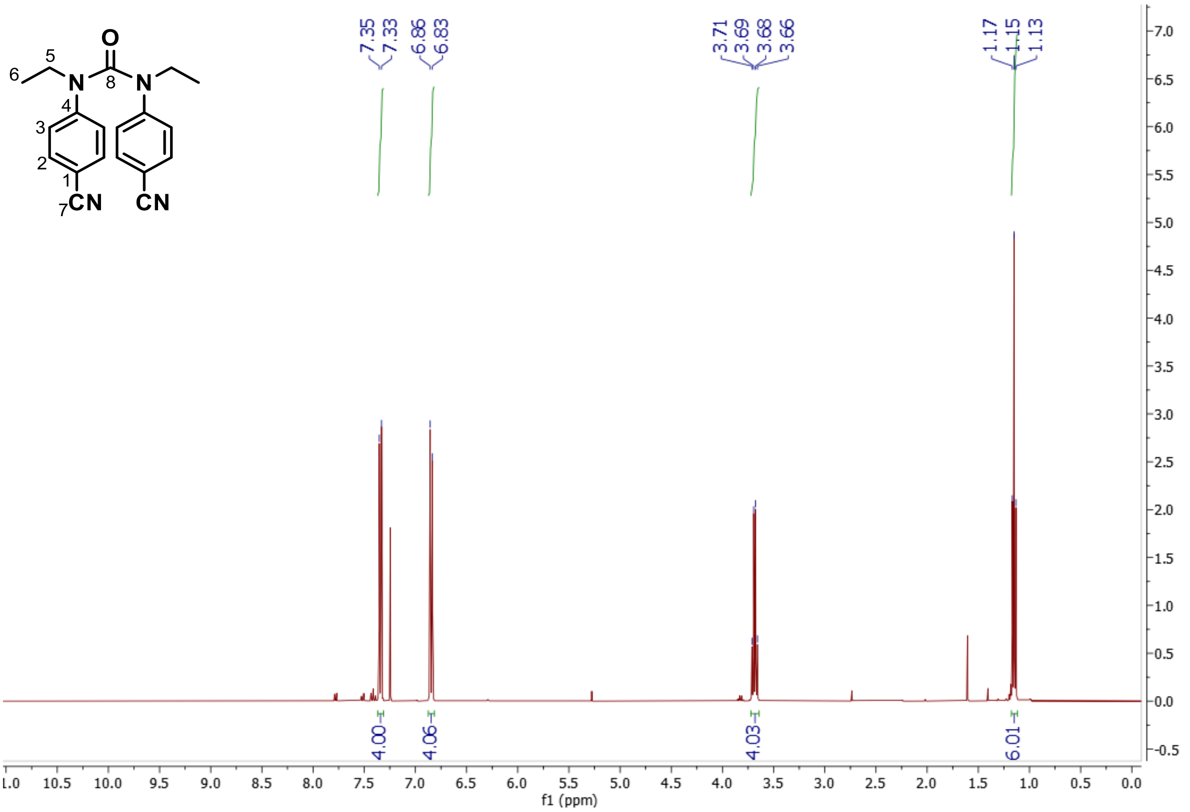

*Supplementary Figure 150. ¹H NMR of N,N'-bis(4-cyanophenyl)-N,N'-diethylurea (**5b**) (400 MHz, 20 °C, CDCl₃)*

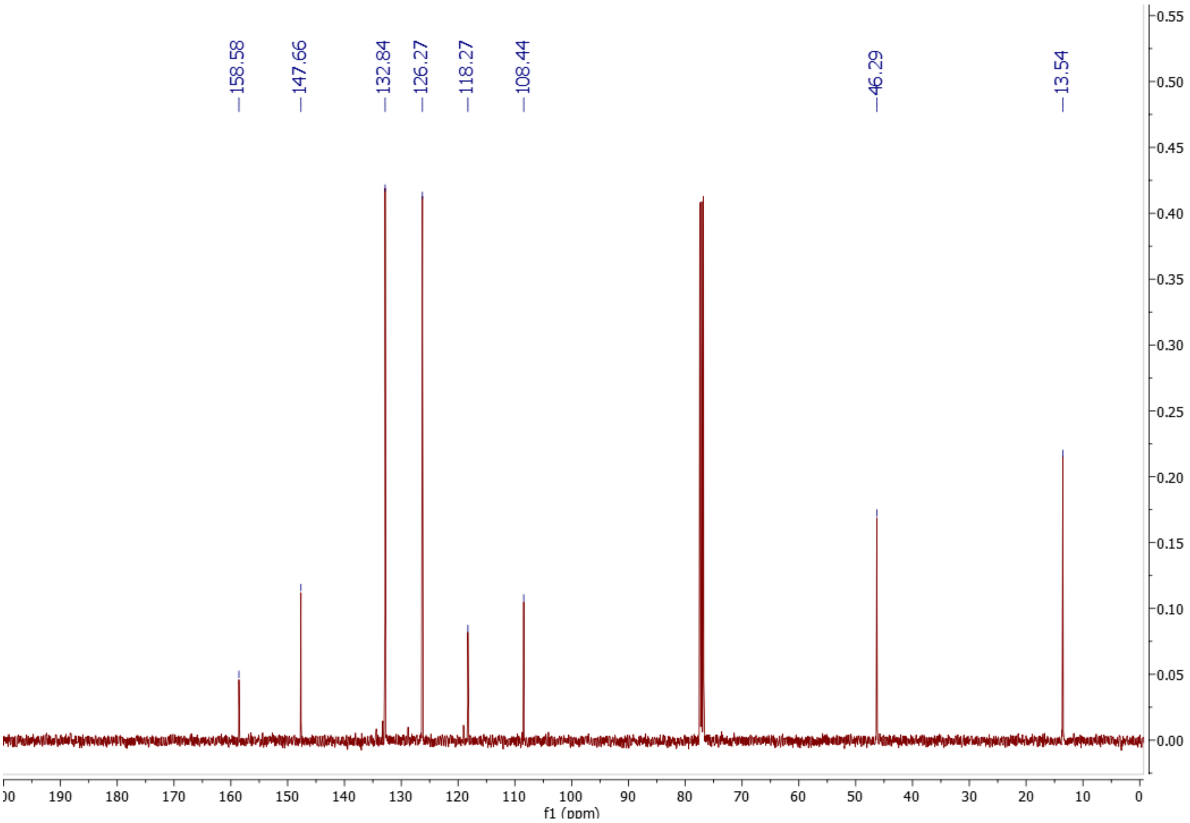

*Supplementary Figure 151. ¹³C NMR of N,N'-bis(4-cyanophenyl)-N,N'-diethylurea (**5b**) (101 MHz, 20 °C, CDCl₃)*

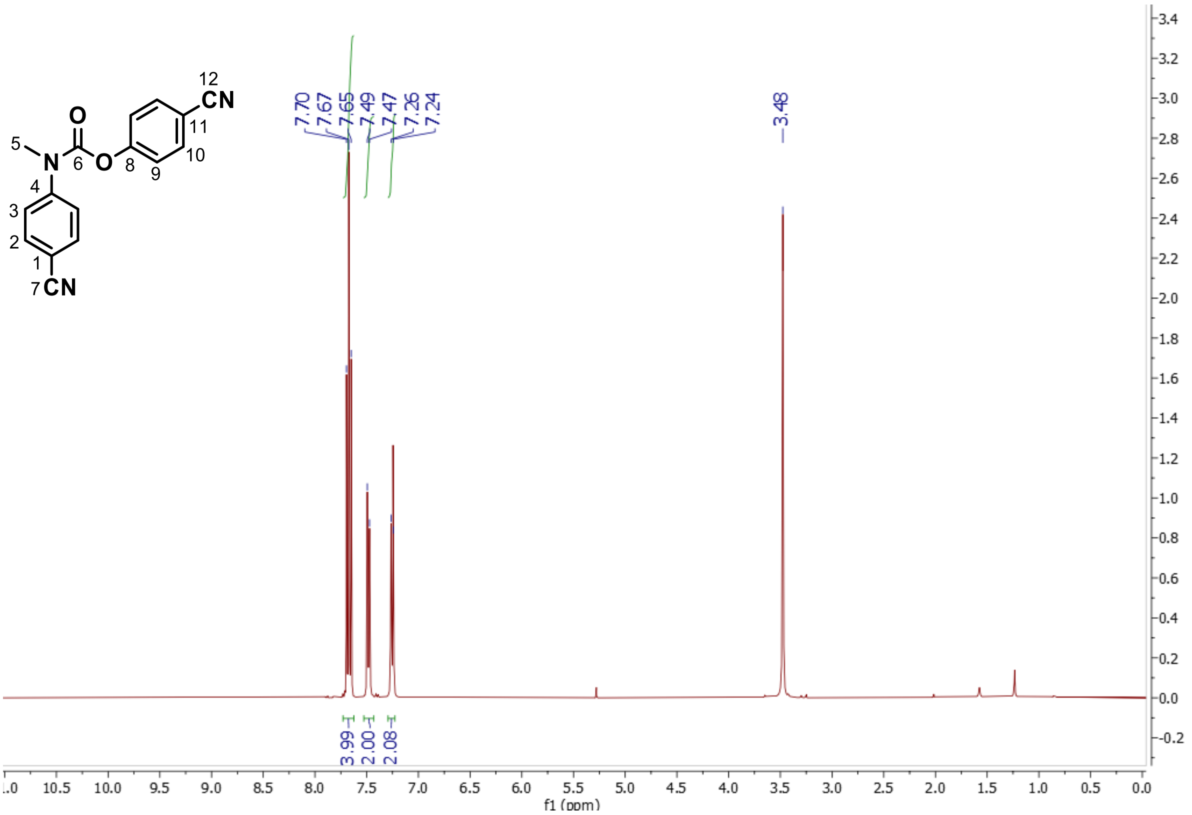

*Supplementary Figure 152. ^{1}H NMR of 4-cyanophenyl (4-cyanophenyl)(methyl)carbamate (**6b**) (400 MHz, 20 °C, $CDCl_3$)*

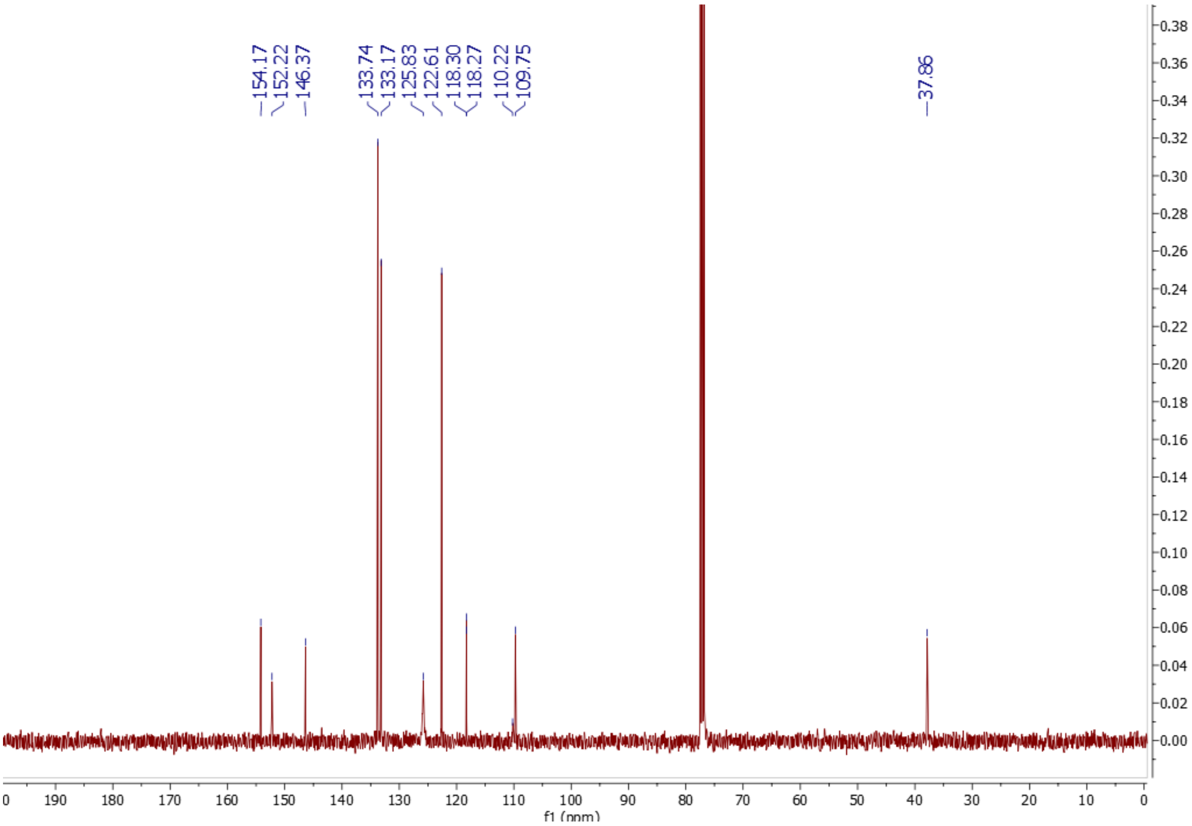

*Supplementary Figure 153. ^{13}C NMR of 4-cyanophenyl (4-cyanophenyl)(methyl)carbamate (**6b**) (101 MHz, 20 °C, $CDCl_3$)*

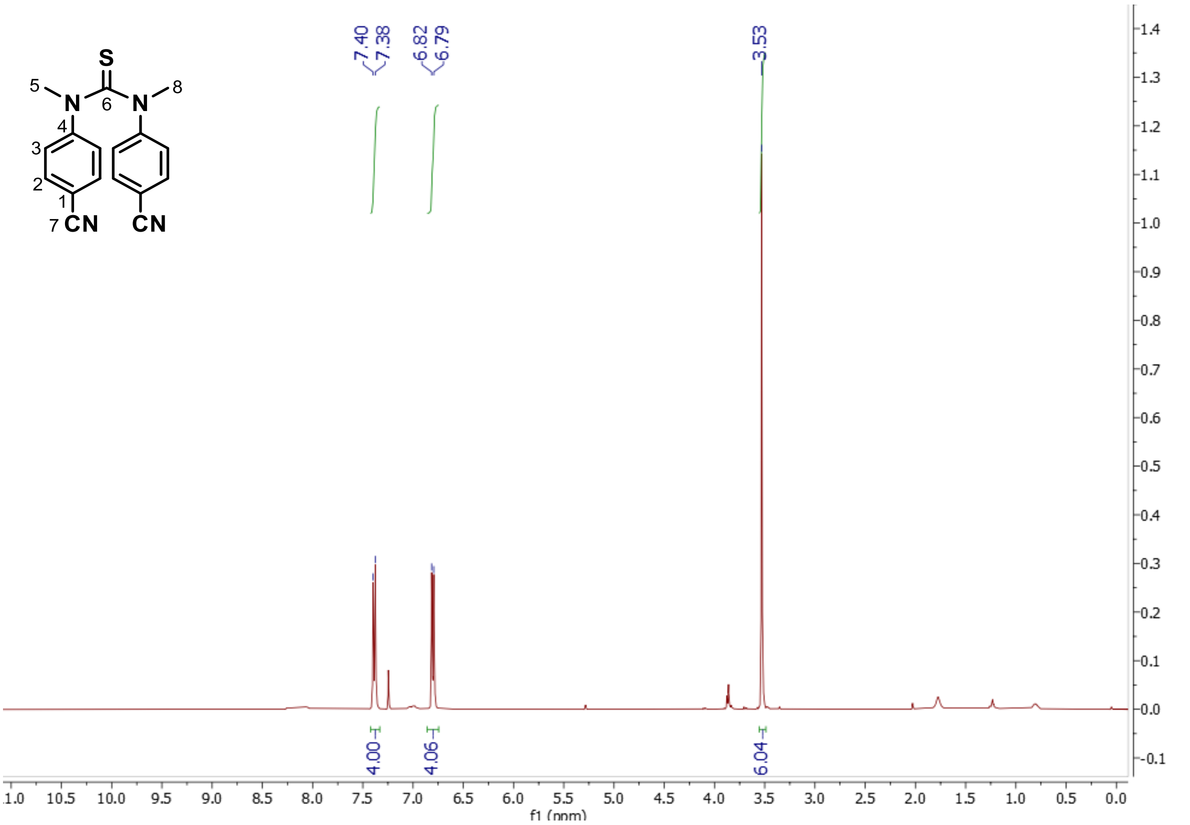

*Supplementary Figure 154. ¹H NMR of N,N'-bis(4-cyanophenyl)-N,N'-dimethylthiourea (**7b**) (400 MHz, 20 °C, $CDCl_3$)*

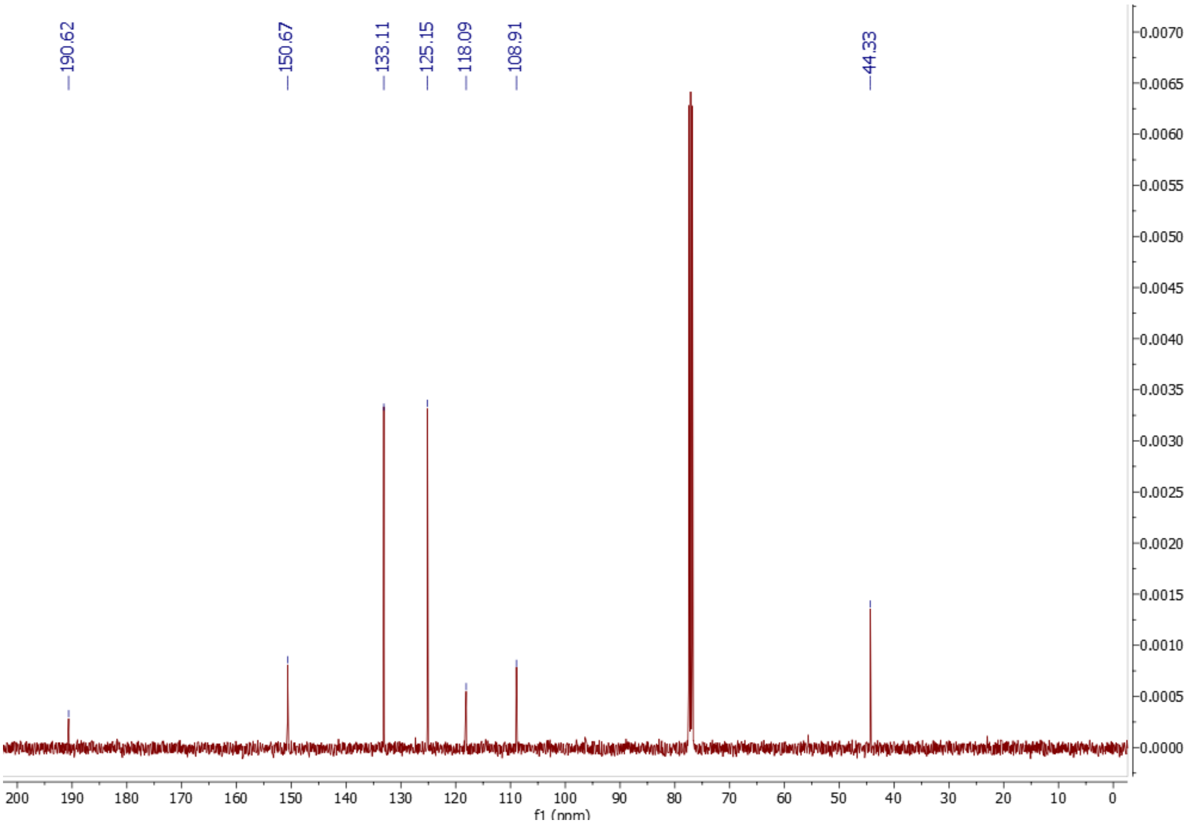

*Supplementary Figure 155. ¹³C NMR of N,N'-bis(4-cyanophenyl)-N,N'-dimethylthiourea (**7b**) (101 MHz, 20 °C, $CDCl_3$)*

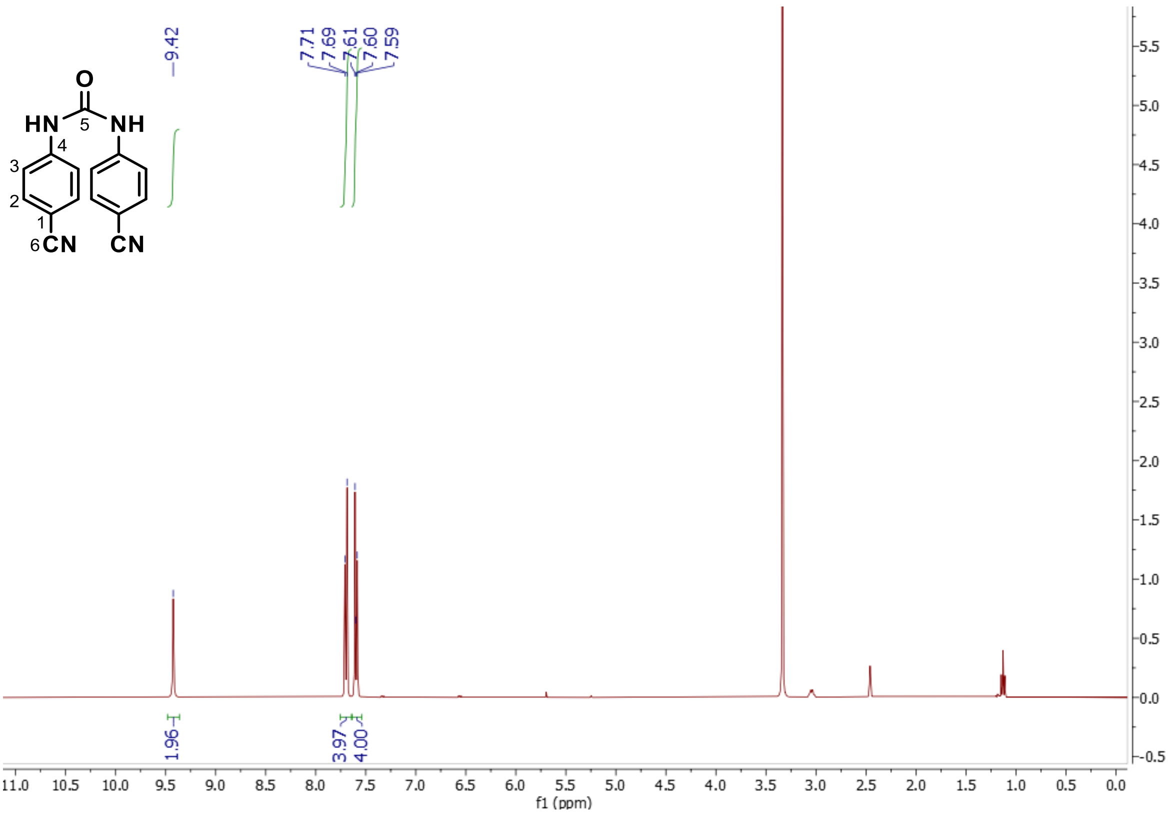

*Supplementary Figure 156. ^{1}H NMR of N,N'-bis(4-cyanophenyl)urea (**8b**) (400 MHz, 20 °C, DMSO-d_6)*

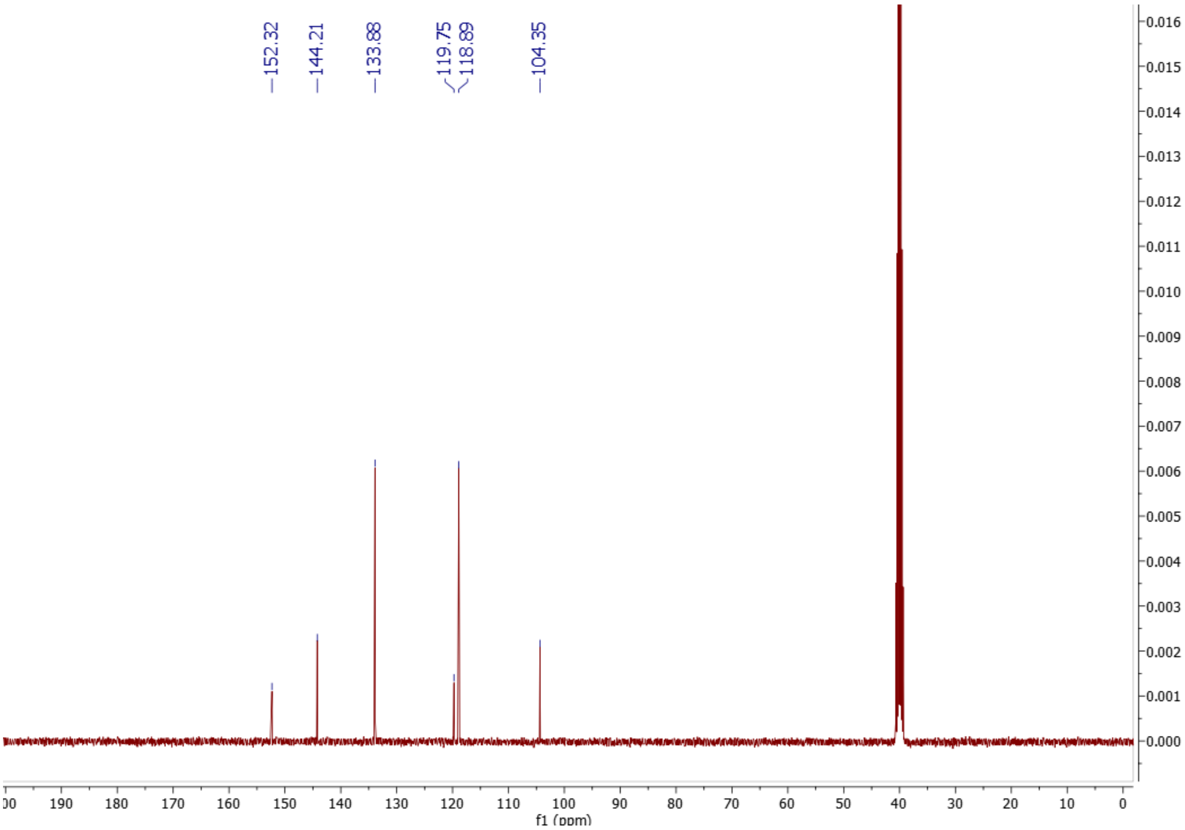

*Supplementary Figure 157. ^{13}C NMR of N,N'-bis(4-cyanophenyl)urea (**8b**) (101 MHz, 20 °C, DMSO-d_6)*

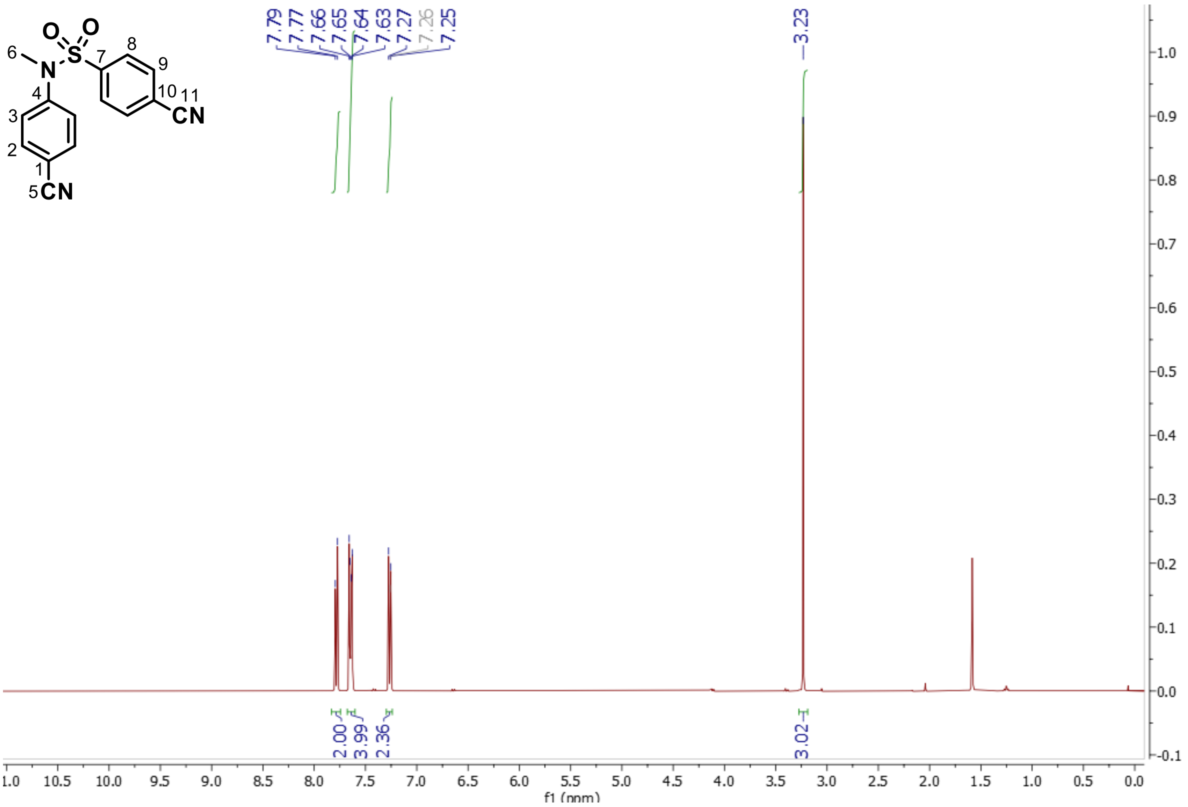

Supplementary Figure 158. ^{1}H NMR of 4-cyano-N-(4-cyanophenyl)-N-methylbenzenesulfonamide (**9b**) (400 MHz, 20 °C, $CDCl_3$)

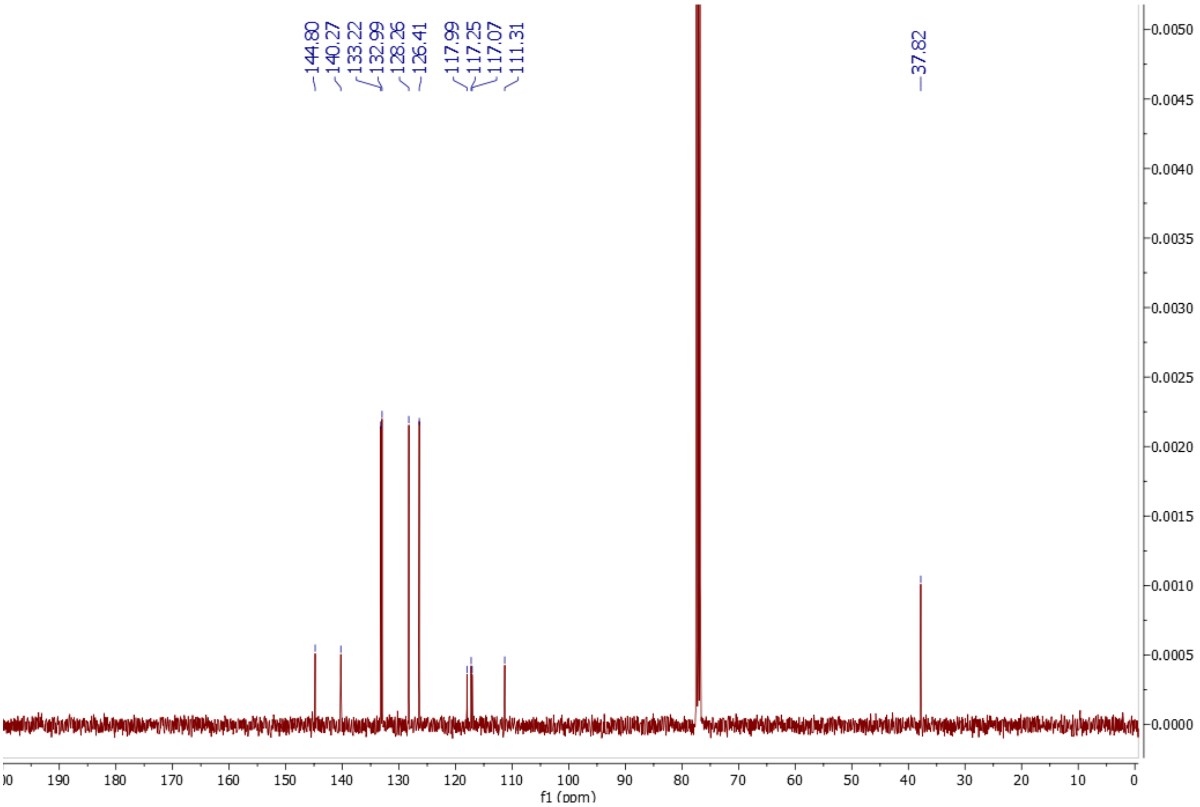

Supplementary Figure 159. ^{13}C NMR of 4-cyano-N-(4-cyanophenyl)-N-methylbenzenesulfonamide (**9b**) (101 MHz, 20 °C, $CDCl_3$)

12.4 NMR spectra of synthetic intermediates

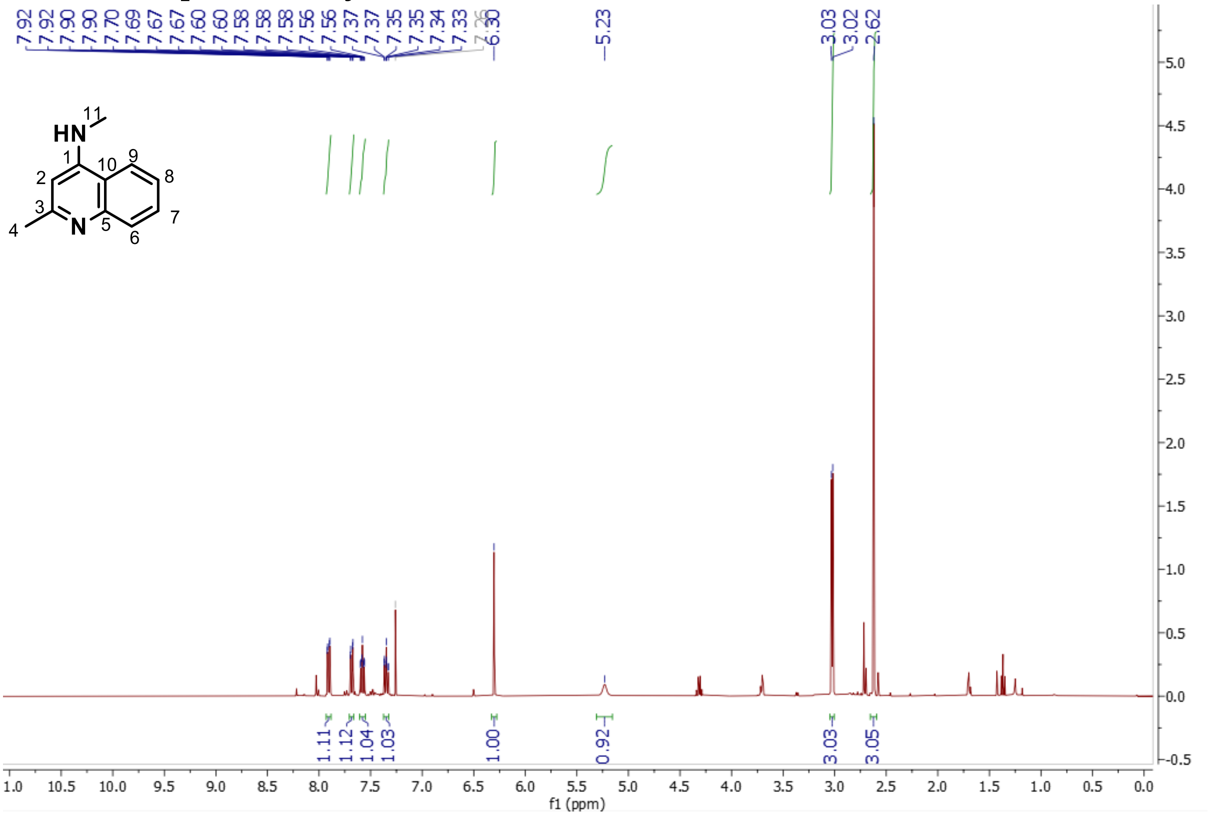

Supplementary Figure 160. ^{1}H NMR of N,2-dimethylquinolin-4-amine (**10**) (400 MHz, 20 °C, $CDCl_3$)

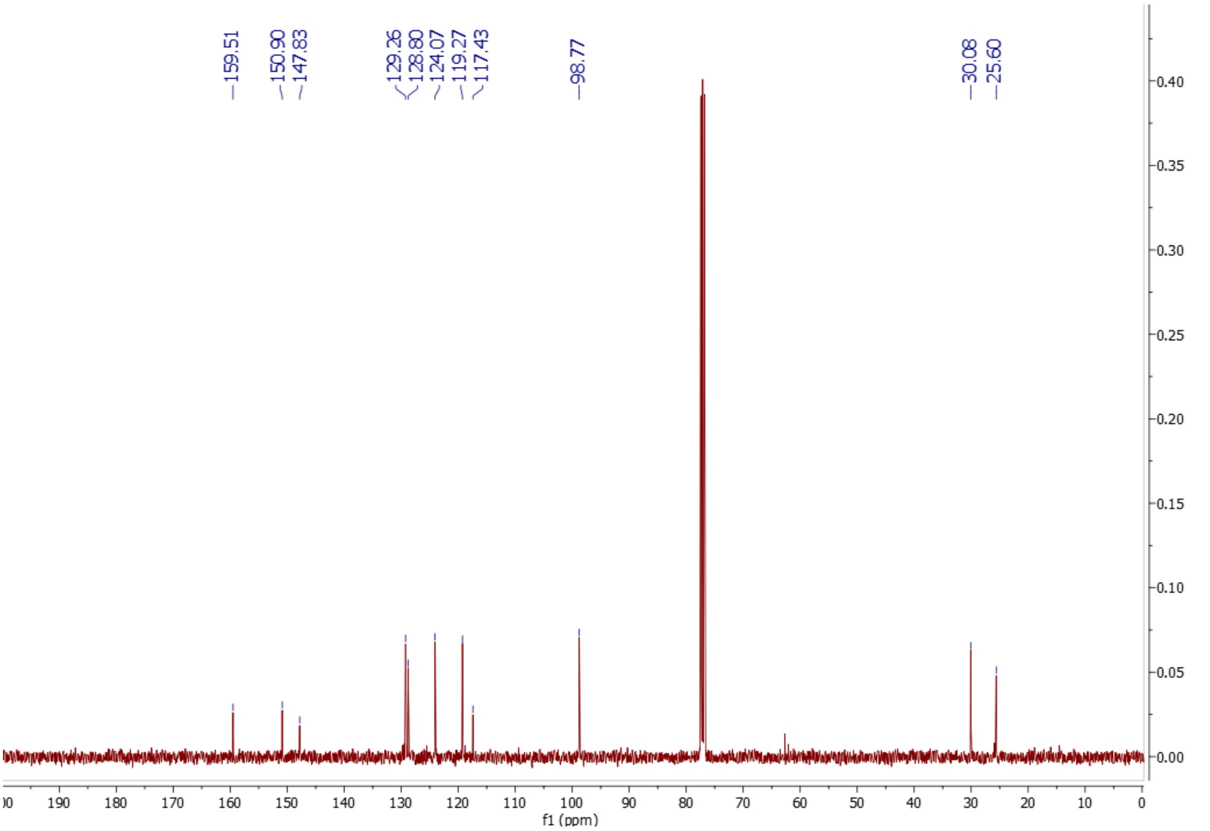

Supplementary Figure 161. ^{13}C NMR of N,2-dimethylquinolin-4-amine (**10**) (101 MHz, 20 °C, $CDCl_3$)

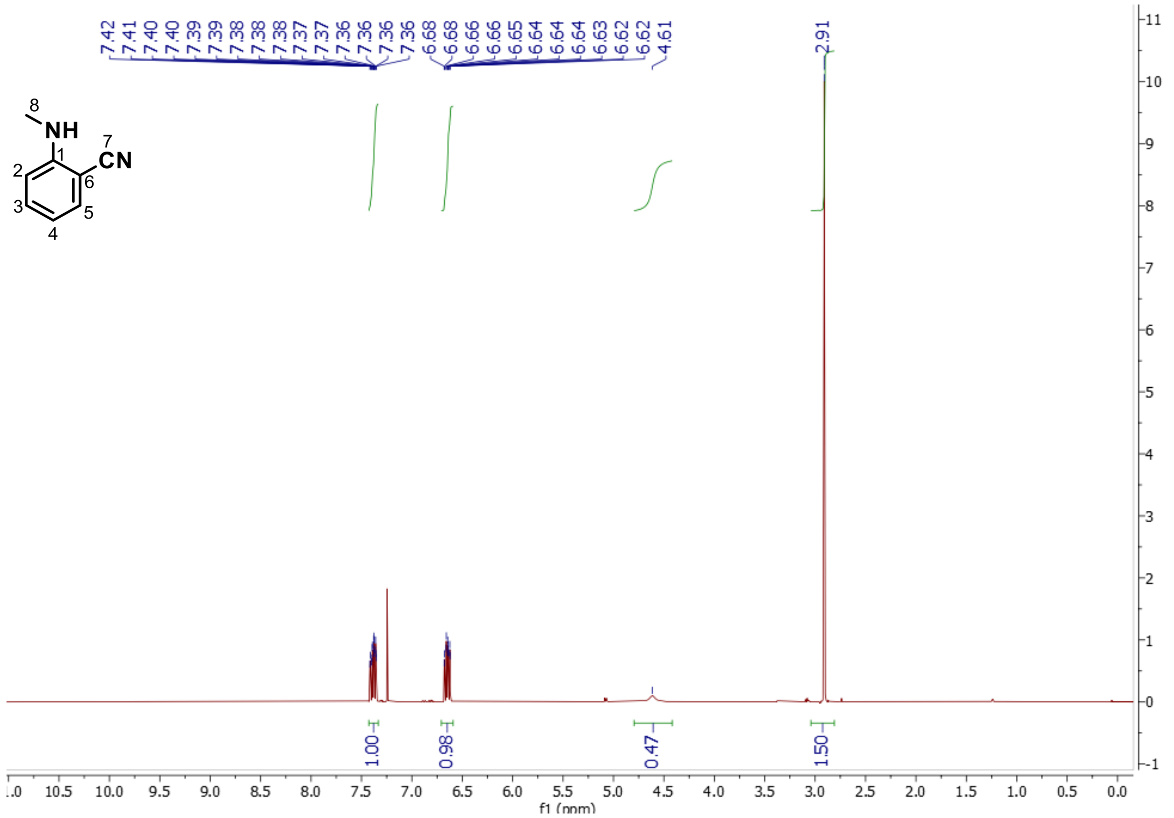

*Supplementary Figure 162. ^{1}H NMR of 2-(methylamino)benzonitrile (**11**) (400 MHz, 20 °C, $CDCl_3$)*

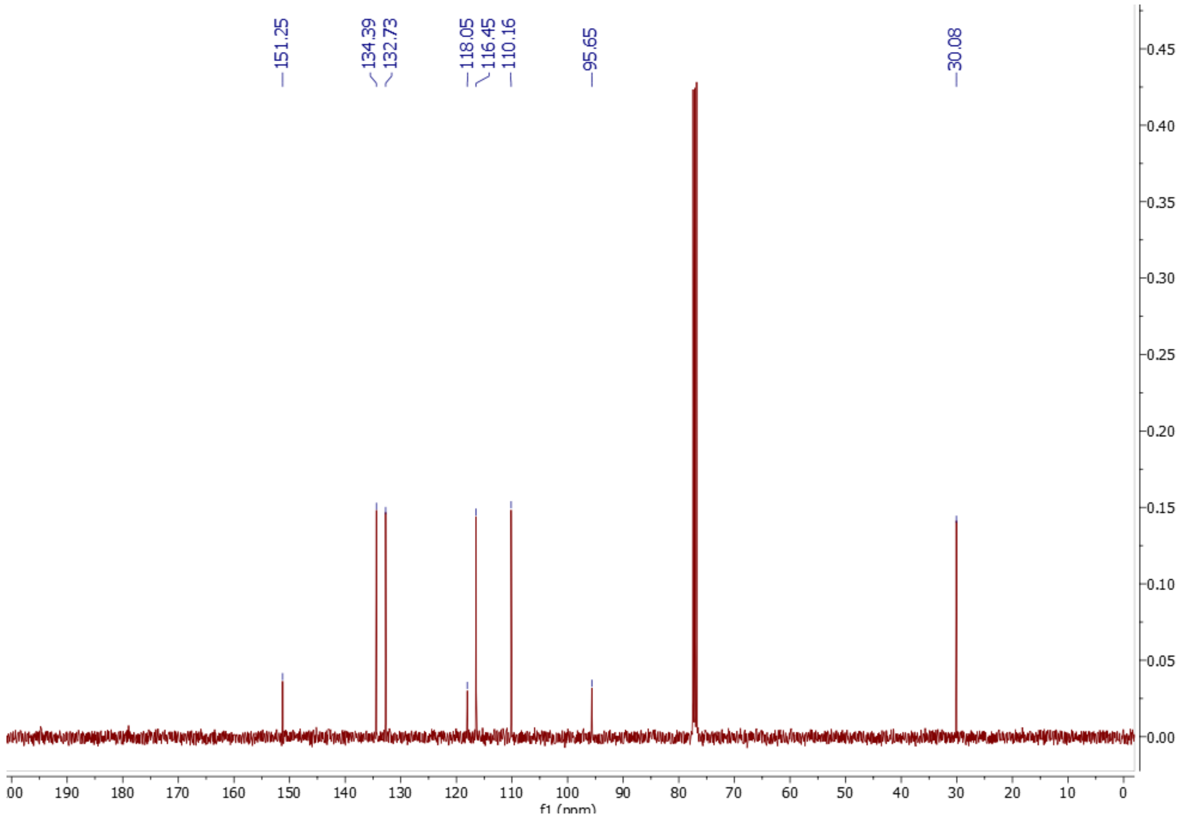

*Supplementary Figure 163. ^{13}C NMR of 2-(methylamino)benzonitrile (**11**) (101 MHz, 20 °C, $CDCl_3$)*

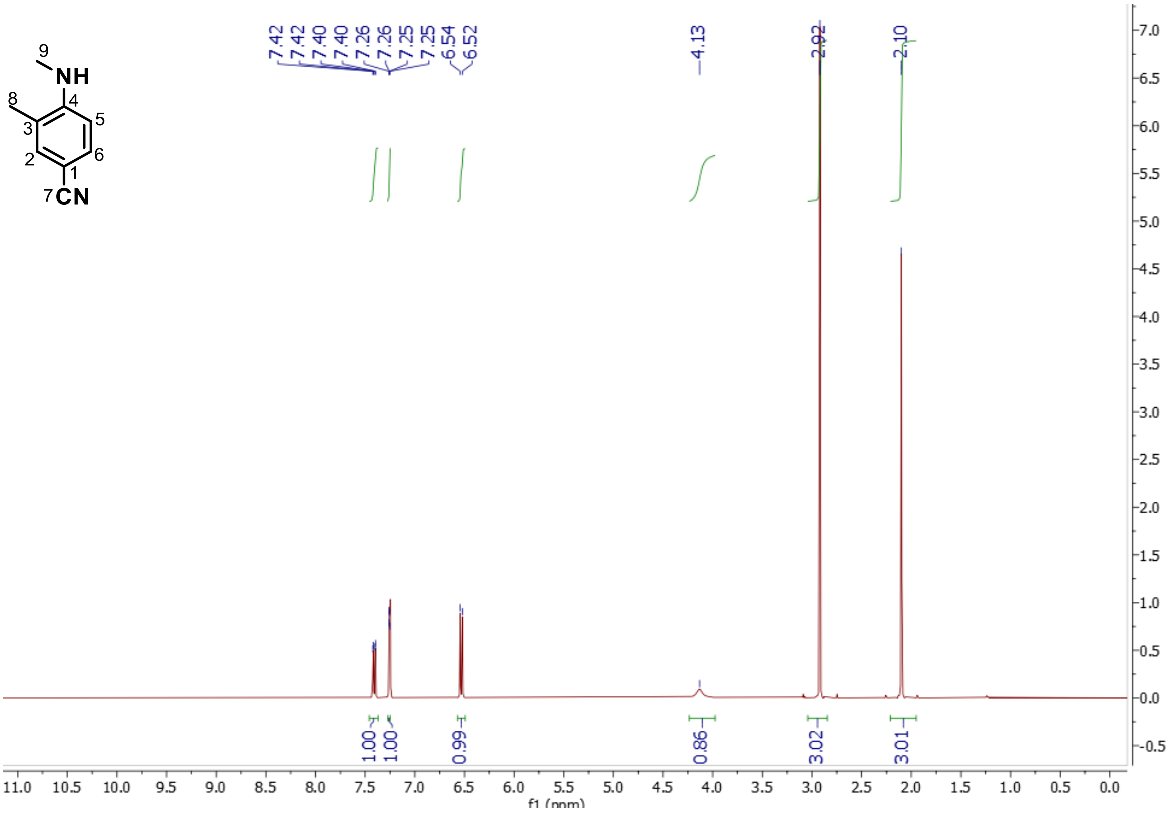

*Supplementary Figure 164. ^{1}H NMR of 3-methyl-4-(methylamino)benzonitrile (**12**) (400 MHz, 20 °C, $CDCl_3$)*

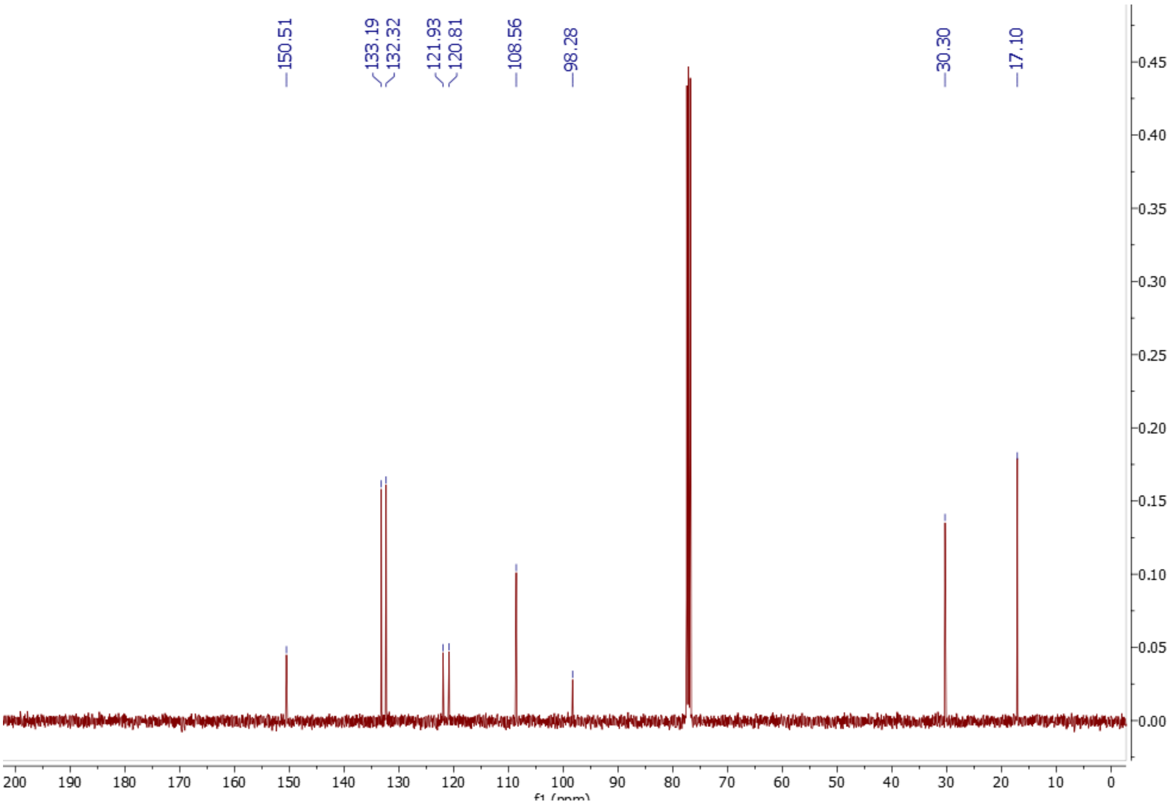

*Supplementary Figure 165. ^{13}C NMR of 3-methyl-4-(methylamino)benzonitrile (**12**) (101 MHz, 20 °C, $CDCl_3$)*

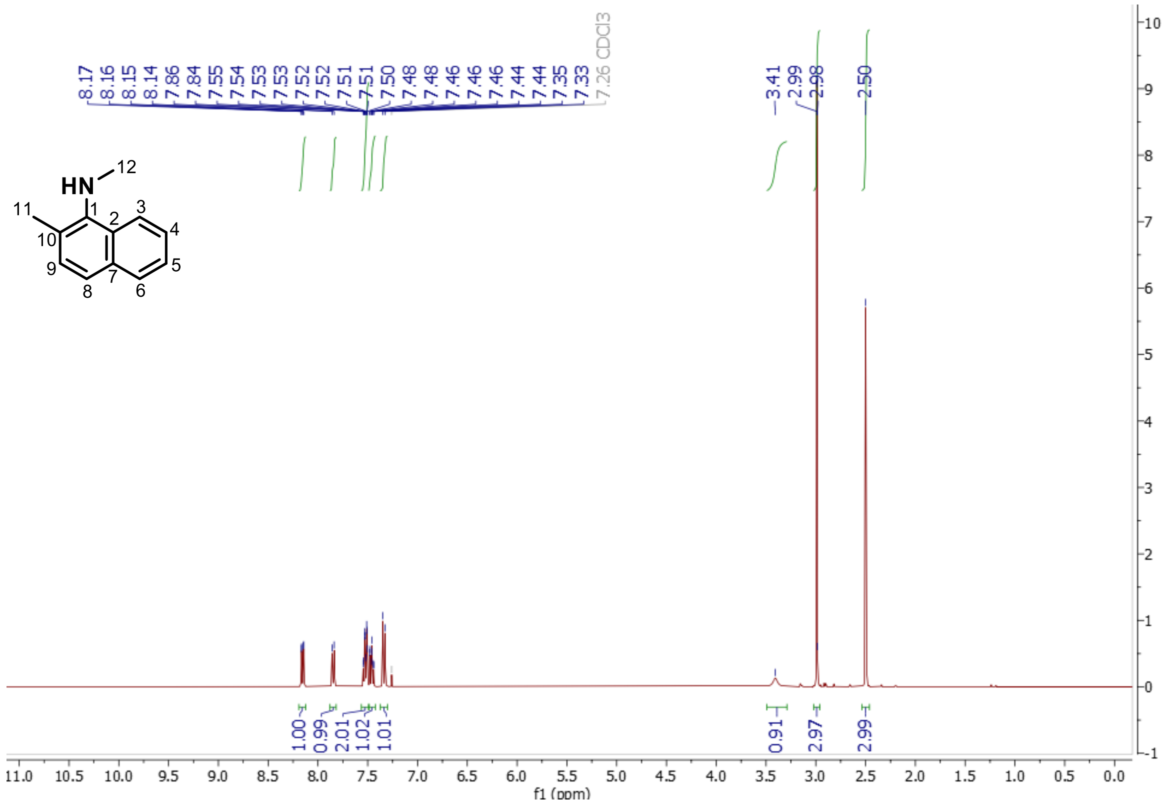

*Supplementary Figure 166. ^{1}H NMR of N,2-dimethylnaphthalen-1-amine (**13**) (400 MHz, 20 °C, $CDCl_3$)*

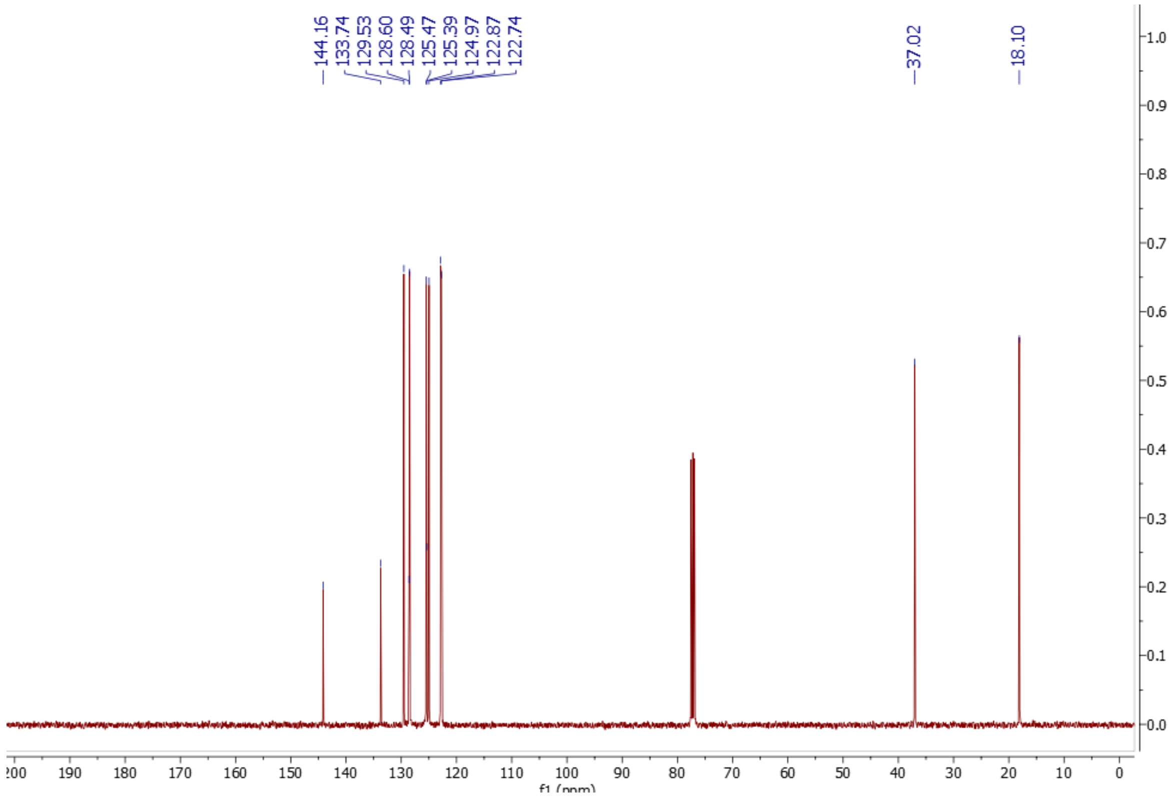

*Supplementary Figure 167. ^{13}C NMR of N,2-dimethylnaphthalen-1-amine (**13**) (101 MHz, 20 °C, $CDCl_3$)*

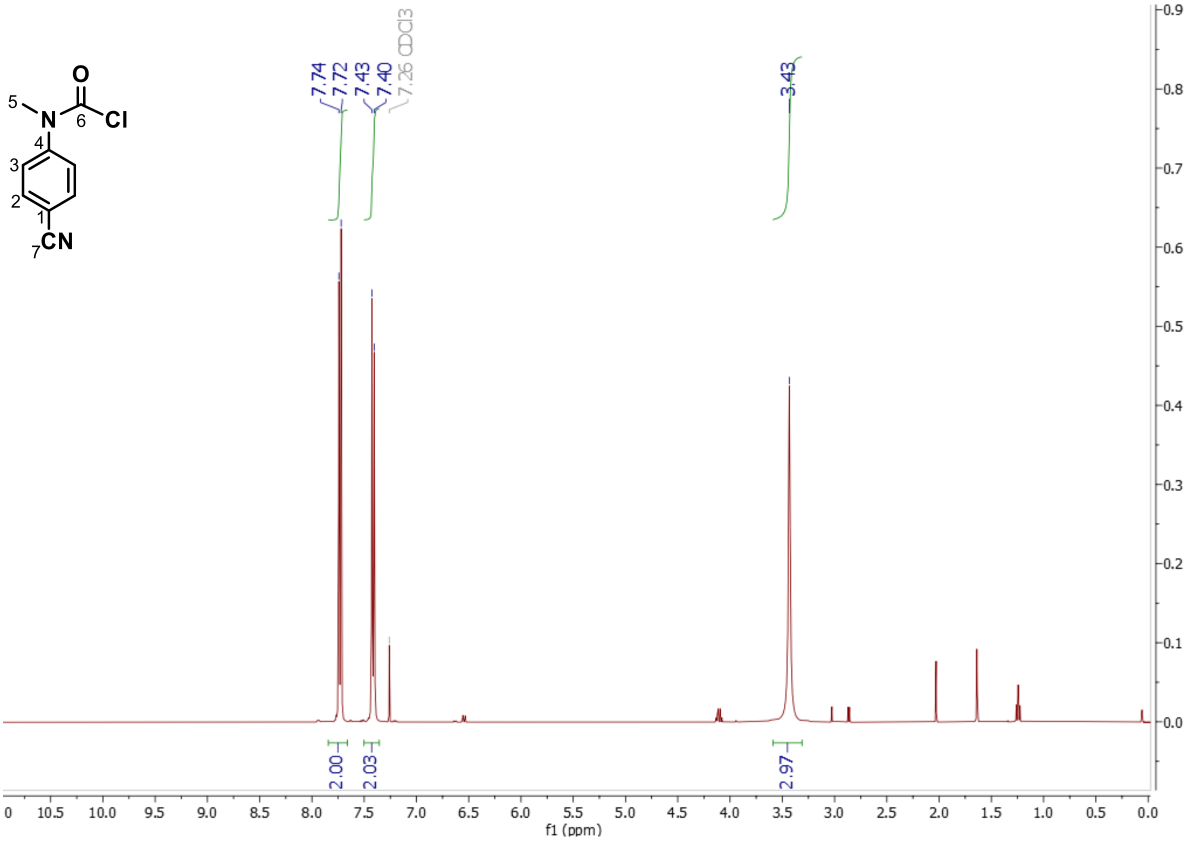

*Supplementary Figure 168. ^{1}H NMR of (4-cyanophenyl)(methyl)carbamic chloride (**14**) (400 MHz, 20 °C, $CDCl_3$)*

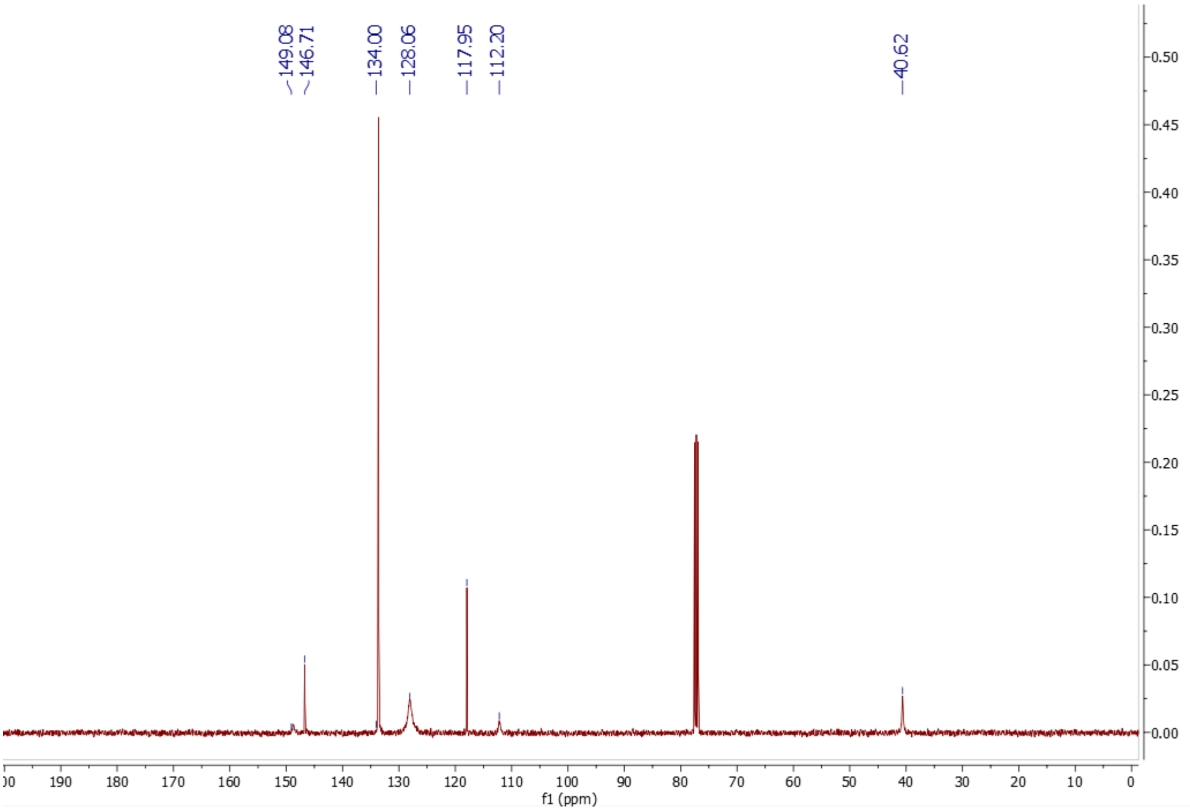

*Figure 169. ^{13}C NMR of (4-cyanophenyl)(methyl)carbamic chloride (**14**) (101 MHz, 20 °C, $CDCl_3$)*

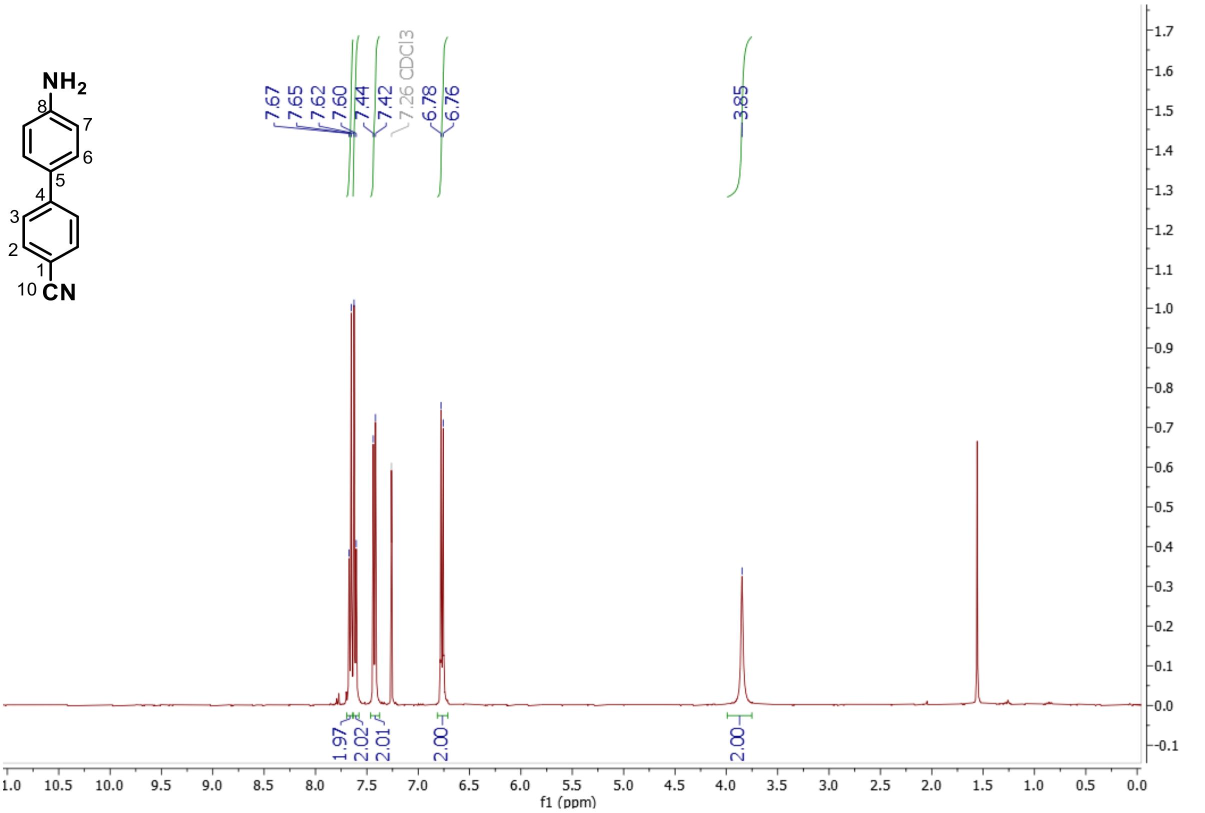

*Supplementary Figure 170. ^{1}H NMR of 4'-Amino-[1,1'-biphenyl]-4-carbonitrile (**15**) (400 MHz, 20 °C, $CDCl_3$)*

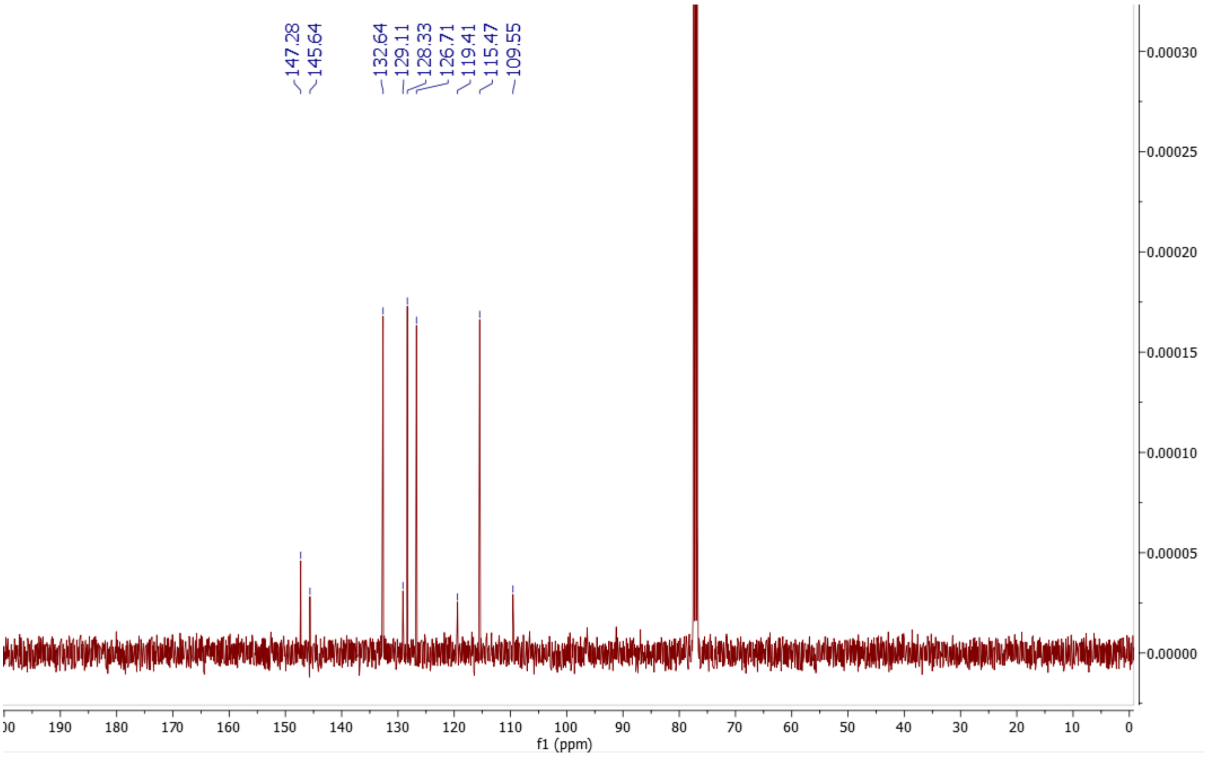

*Supplementary Figure 171. ^{13}C NMR of 4'-Amino-[1,1'-biphenyl]-4-carbonitrile (**15**) (101 MHz, 20 °C, $CDCl_3$)*

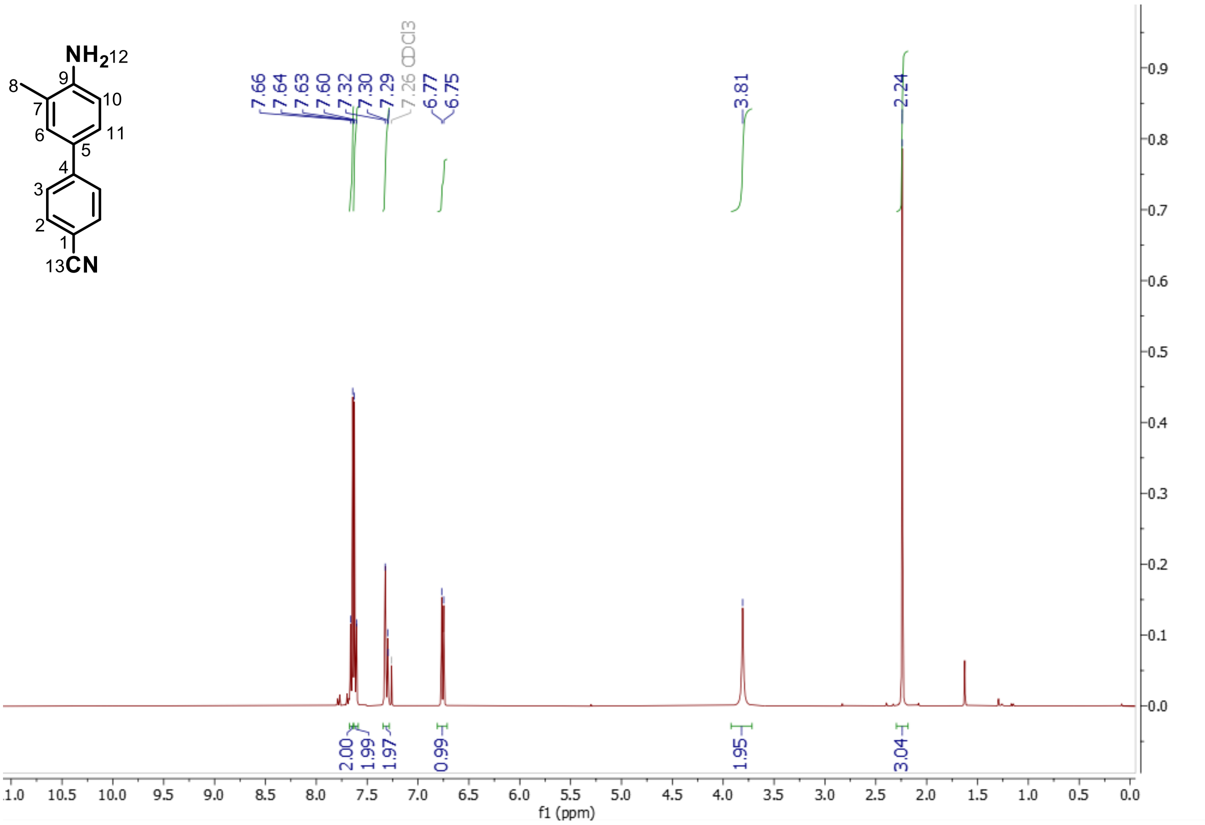

*Supplementary Figure 172. ^{1}H NMR of 4'-amino-3'-methyl-[1,1'-biphenyl]-4-carbonitrile (**16**) (400 MHz, 20 °C, $CDCl_3$)*

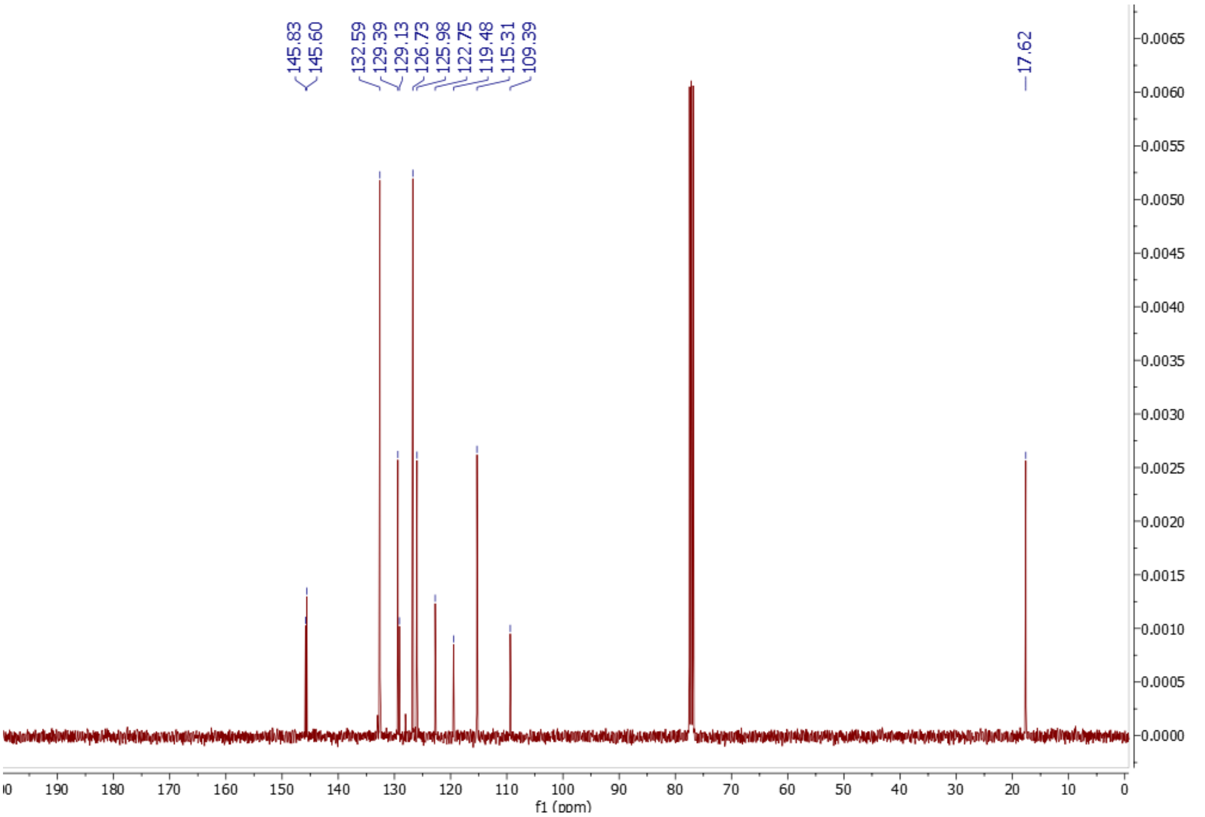

*Supplementary Figure 173. ^{13}C NMR of 4'-amino-3'-methyl-[1,1'-biphenyl]-4-carbonitrile (**16**) (101 MHz, 20 °C, $CDCl_3$)*

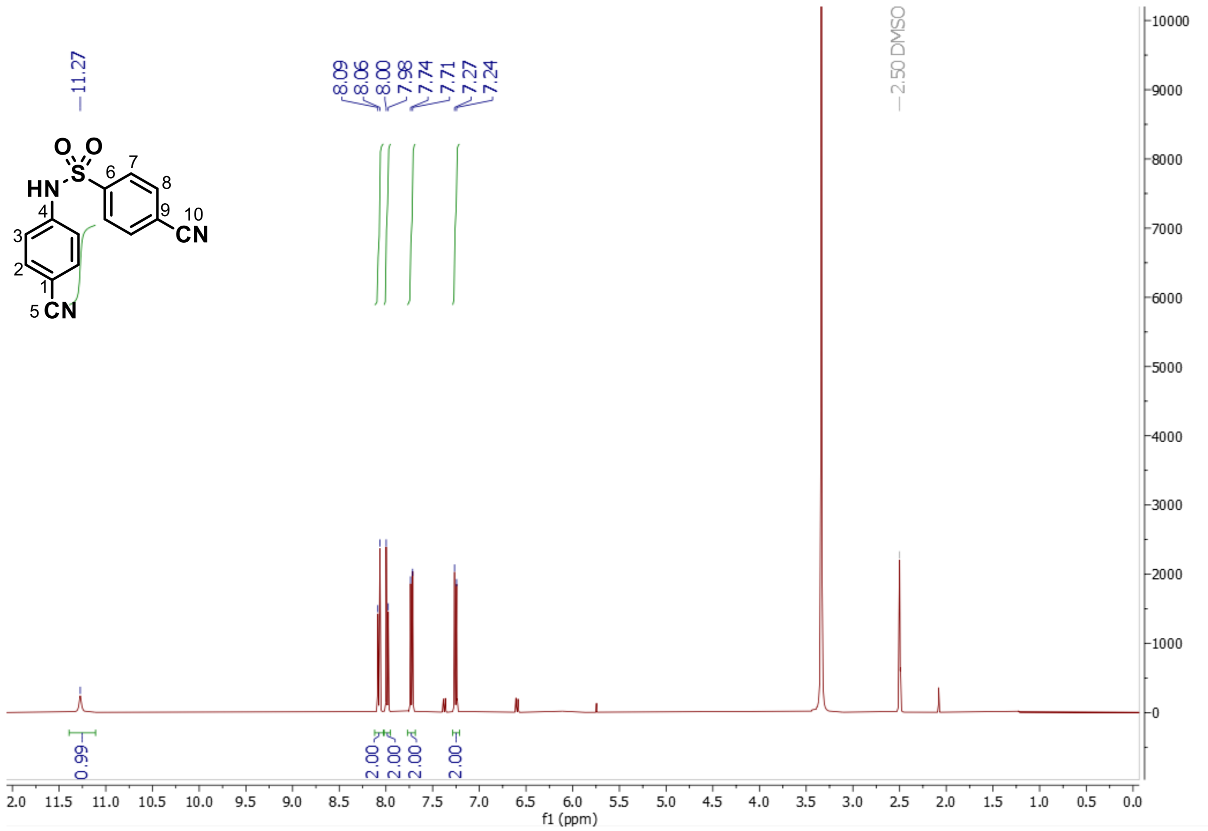

*Supplementary Figure 174. ¹H NMR of 4-cyano-N-(4-cyanophenyl)benzenesulfonamide (**17**) (400 MHz, 20 °C, CDCl₃)*

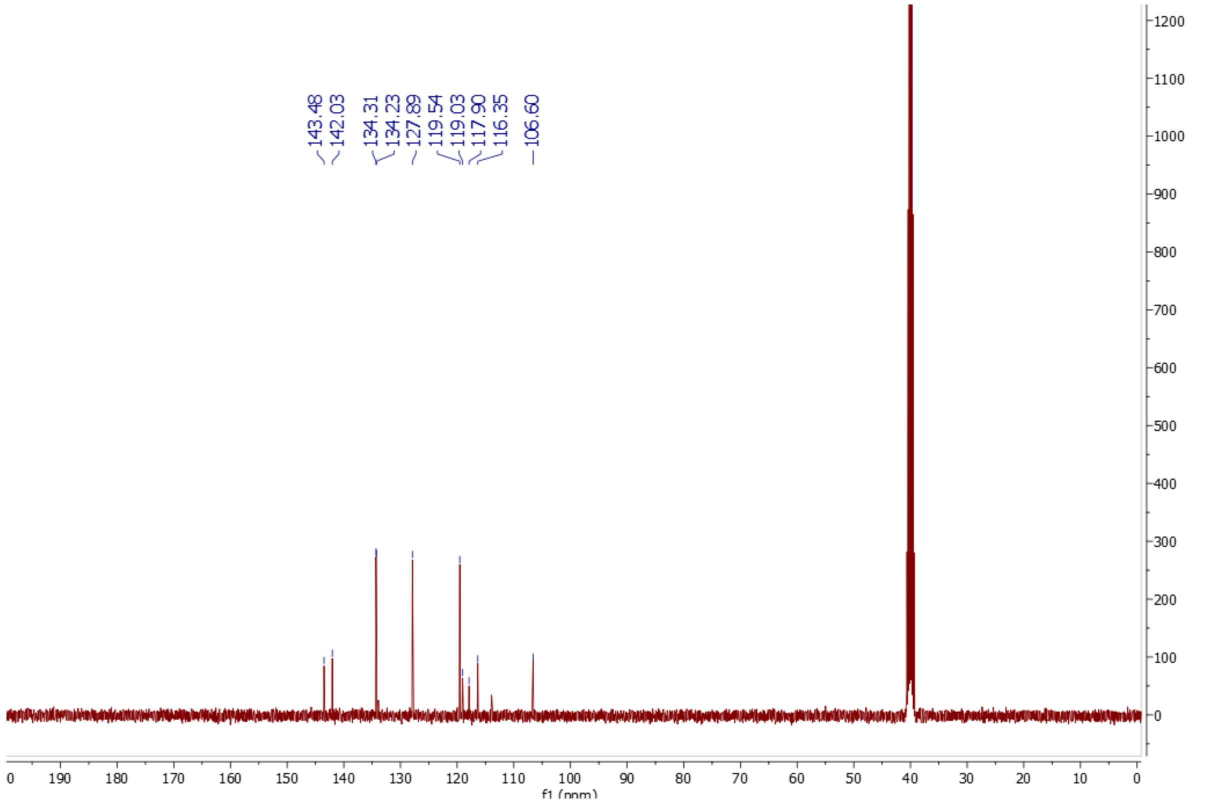

*Supplementary Figure 175. ¹³C NMR of 4-cyano-N-(4-cyanophenyl)benzenesulfonamide (**17**) (101 MHz, 20 °C, CDCl₃)*

12.5 NMR spectra of compounds synthesised for mechanistic investigation

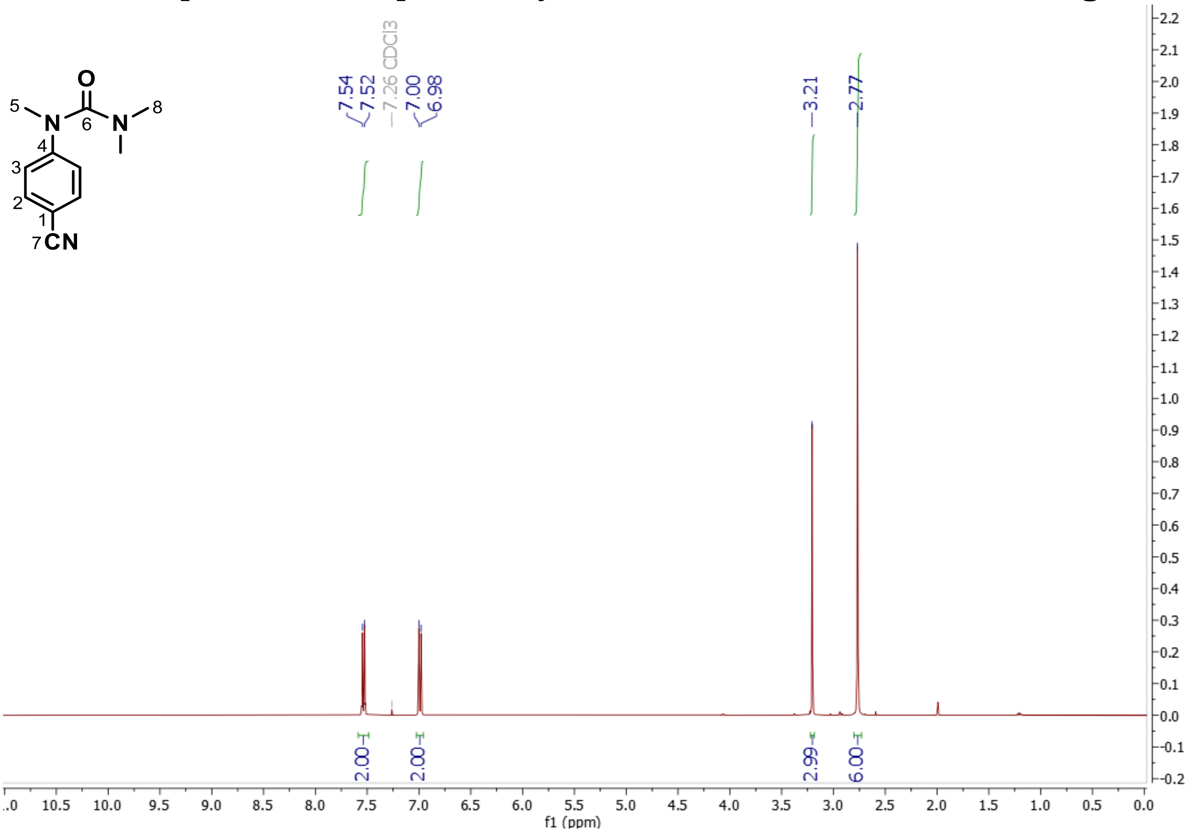

*Supplementary Figure 176. ^{1}H NMR of N-(4-cyanophenyl)-N,N',N'-trimethylurea (**3b**) (400 MHz, 20 °C, $CDCl_3$)*

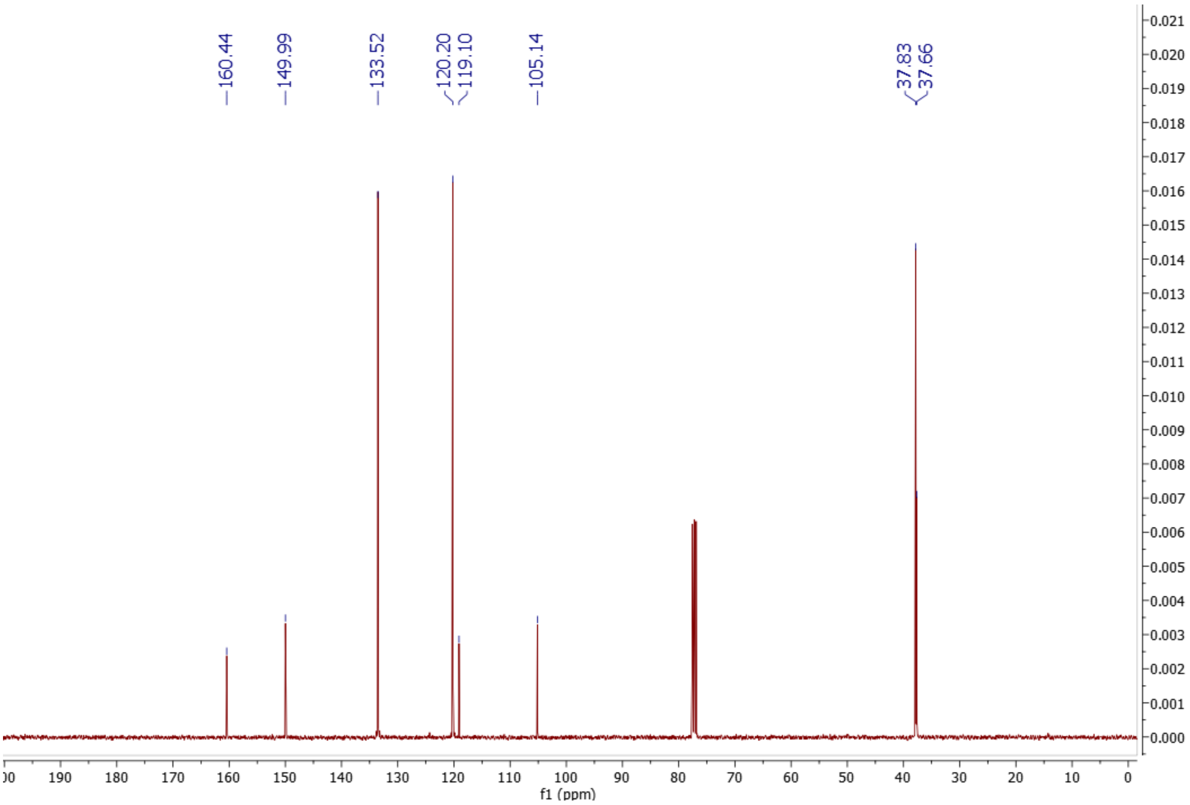

*Supplementary Figure 177. ^{13}C NMR of N-(4-cyanophenyl)-N,N',N'-trimethylurea (**3b**) (100 MHz, 20 °C, $CDCl_3$)*

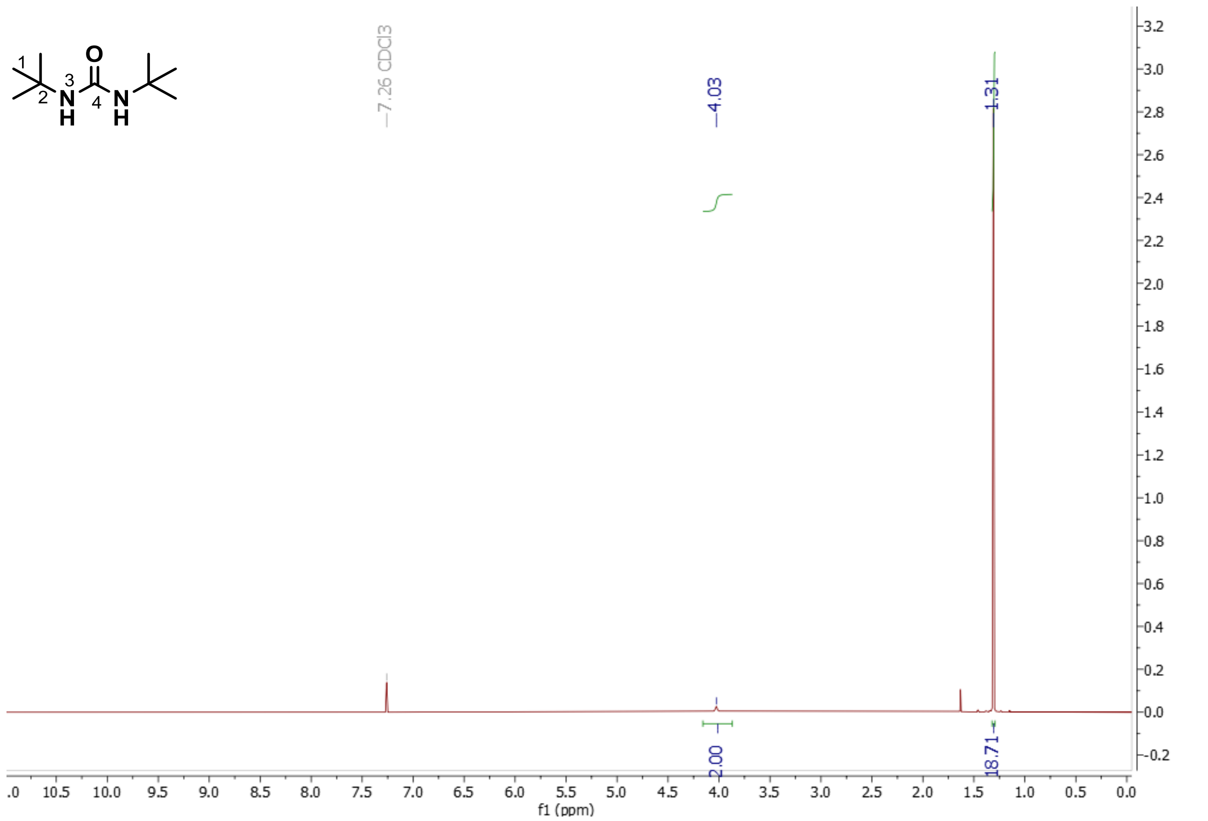

Supplementary Figure 178. ^{1}H NMR of N,N’-di-tert-butylurea (400 MHz, 20 °C, $CDCl_3$)

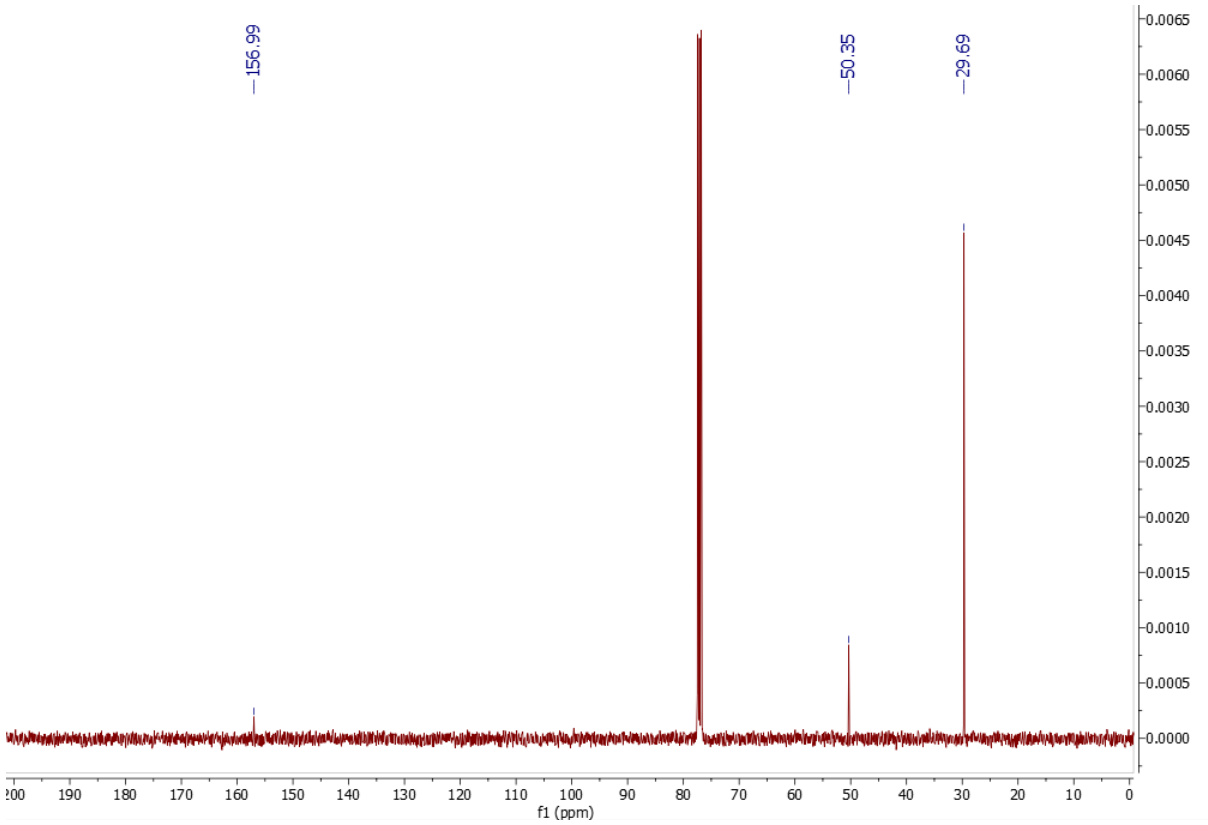

Supplementary Figure 179. ^{13}C NMR of N,N’-di-tert-butylurea (101 MHz, 20 °C, $CDCl_3$)

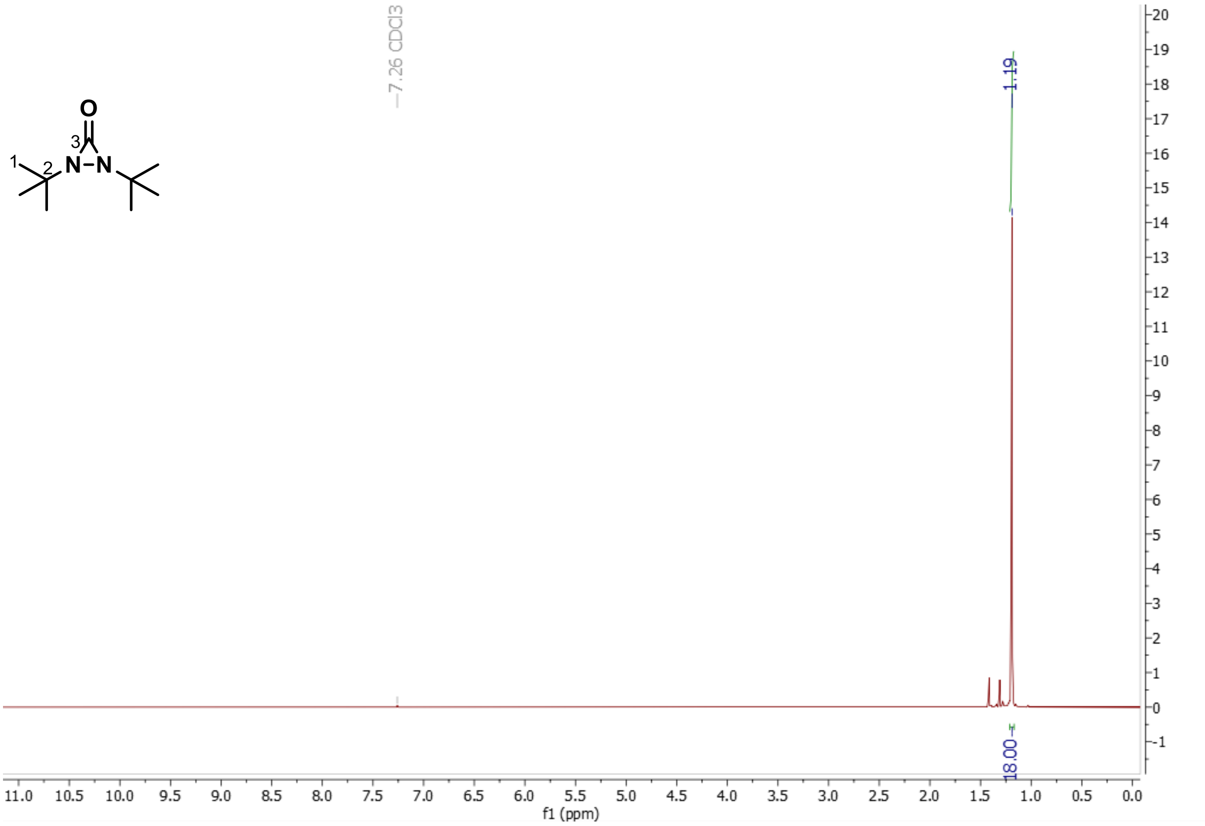

Supplementary Figure 180. ¹H NMR of 1,2-di-tert-butyldiaziridin-3-one (400 MHz, 20 °C, CDCl₃)

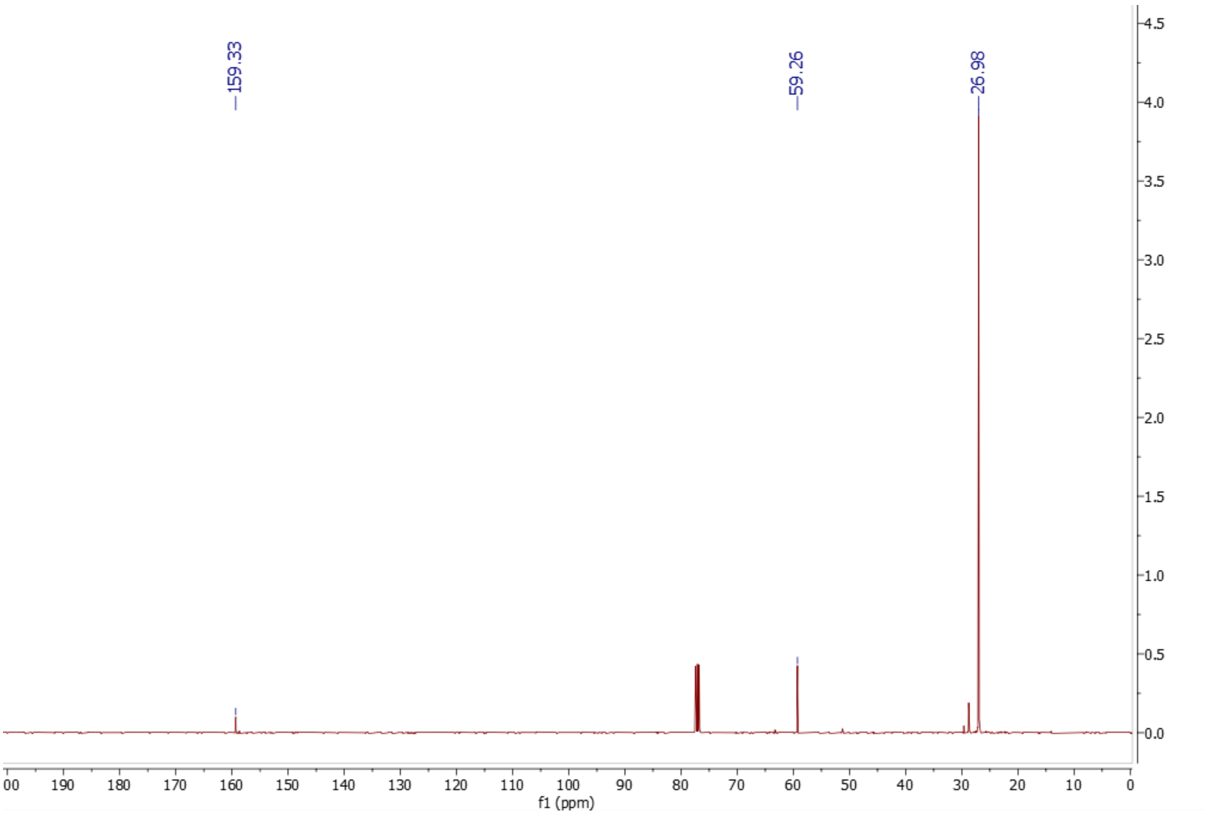

Supplementary Figure 181. ¹³C NMR of 1,2-di-tert-butyldiaziridin-3-one (101 MHz, 20 °C, CDCl₃)

13. Supplementary References

(1) Clayden, J.; Lemiègre, L.; Pickworth, M.; Jones, L. Conformation and Stereodynamics of 2,2′-Disubstituted N,N′-Diaryl Ureas. *Org. Biomol. Chem.* **2008**, *6* (16), 2908–2913.

(2) Lefranc, J.; Tetlow, D. J.; Donnard, M.; Minassi, A.; Gálvez, E.; Clayden, J. Geometry-Selective Synthesis of E or Z N-Vinyl Ureas (N -Carbamoyl Enamines). *Org. Lett.* **2011**, *13* (2), 296–299.

(3) Watson, R. B.; Butler, T. W.; Deforest, J. C. Preparation of Carbamates, Esters, Amides, and Unsymmetrical Ureas via Brønsted Acid-Activated N-Acyl Imidazoliums. *Org. Process Res. Dev.* **2021**, *25* (3), 500–506.

(4) Velavan, A.; Sumathi, S.; Balasubramanian, K. Biomolecular Chemistry Unsymmetrical Tetrasubstituted Ureas from Tertiary Carbamoylimidazole: Activation by AlMe3. *Org. Biomol. Chem.* **2012**, No. 10, 6420–6431.

(5) Kurth, T. L.; Lewis, F. D. Ground-State Conformational Equilibrium and Photochemical Behavior of Syn and Anti N,N′-Dimethyl-N,N′-Di-1-Naphthylurea Protophanes. *J. Am. Chem. Soc* **2003**, *125*, 13760–13767.

(6) Lepore, G.; Migdal, S.; Blagdon, D. E.; Goodman, M. Conformations of Substituted Arylureas in Solution. *J. Org. Chem.* **1973**, *38* (15), 2590–2594.

(7) Youn, S. W.; Yoo, H. J.; Lee, E. M.; Lee, S. Y. Metal-Free One-Pot Synthesis of (Tetrahydro)Quinolines through Three-Component Assembly of Arenediazonium Salts, Nitriles, and Styrenes. *Adv. Synth. Cat* **2018**, *360* (2), 278–283.

(8) Bhat, A. P. I.; Inam, F.; Bhat, B. R. One-Step Synthesis of Biaryls under Mild Conditions. *Eur. J. Chem* **2013**, *31* (31), 7139–7144.

(9) Moneo, Á.; Fernanda, M.; Carvalho, N.; Telo, J. Dicyanoaromatic Radical Anions as Mixed Valence Species. *J. Phys. Org. Chem.* **2012**, *25* (7), 559–565.

(10) Peshkov, R. Y.; Panteleeva, E. v.; Chunyan, W.; Tretyakov, E. v.; Shteingarts, V. D. One-Pot Synthesis of 4-Alkyl-4-Cyanobiaryls on the Basis of the Terephthalonitrile Dianion and Neutral Aromatic Nitrile Cross-Coupling. *Beilstein J. Org. Chem.* **2016**, *12*, 1577–1584.

(11) Chen, C. X.; Fan, Y. Z.; Cao, C. C.; Wang, H. P.; Fan, Y. N.; Jiang, J. J.; Wei, Z. W.; Maurin, G.; Su, C. Y. Dynamic Coordination Chemistry of Fluorinated Zr-MOFs: Synthetic Control and Reassembly/Disassembly Beyond de Novo Synthesis to Tune the Structure and Property. *Eur. J. Chem.* **2020**, *26* (37), 8254–8261.

(12) Murugesan, K.; Donabauer, K.; Konig, B. Visible-Light-Promoted Metal-Free Synthesis of (Hetero)Aromatic Nitriles from C(Sp3)-H Bonds. *Angew. Chem. Int. Ed* **2021**, *60*, 2439–2445.

(13) Fuentes-Rivera, J. J.; Zick, M. E.; Düfert, M. A.; Milner, P. J. Overcoming Halide Inhibition of Suzuki-Miyaura Couplings with Biaryl Monophosphine-Based Catalysts. *Org. Process Res. Dev.* **2019**, *23* (8), 1631–1637.

(14) Balkenhohl, M.; Ziegler, D. S.; Desaintjean, A.; Bole, L. J.; Kennedy, A. R.; Hevia, E.; Knochel, P. Preparation of Polyfunctional Arylzinc Organometallics in Toluene by Halogen/Zinc Exchange Reactions. *Angew. Chem. Int. Ed* **2019**, *131* (37), 13030–13034.

(15) Che, Y. Y.; Yue, Y.; Lin, L. Z.; Pei, B.; Deng, X.; Feng, C. Palladium-Catalyzed Electrophilic Functionalization of Pyridine Derivatives through Phosphonium Salts. *Angew. Chem. Int. Ed* **2020**, *59* (38), 16414–16419.

(16) Qiu, D.; Meng, H.; Jin, L.; Wang, S.; Tang, S.; Wang, X.; Mo, F.; Zhang, Y.; Wang, J. Synthesis of Aryl Trimethylstannanes from Aryl Amines: A Sandmeyer-Type Stannylation Reaction. *Angew. Chem. Int. Ed.* **2013**, *52* (44), 11581–11584.

(17) Chen, L.; Lang, H.; Fang, L.; Yu, J.; Wang, L. Nickel-Catalyzed Desulfitative Suzuki-Miyaura Cross-Coupling of N,N-Disulfonylmethylamines and Arylboronic Acids. *Eur. J. Chem.* **2014**, *2014* (29), 6385–6389. https://doi.org/10.1002/ejoc.201402919.

(18) Nagaki, A.; Ichinari, D.; Yoshida, J. I. Three-Component Coupling Based on Flash Chemistry. Carbolithiation of Benzyne with Functionalized Aryllithiums Followed by Reactions with Electrophiles. *J. Am. Chem. Soc* **2014**, *136* (35), 12245–12248.

(19) Lim, T.; Ryoo, J. Y.; Jang, M.; Han, M. S. Ligand-Free Suzuki-Miyaura Cross-Coupling with Low Pd Content: Rapid Development by a Fluorescence-Based High-Throughput Screening Method. *Org. Biomol. Chem.* **2021**, *19* (5), 1009–1016.

(20) Gan, Y.; Wang, G.; Xie, X.; Liu, Y. Nickel-Catalyzed Cyanation of Phenol Derivatives with Zn(CN)2 Involving C-O Bond Cleavage. *J. Org. Chem.* **2018**, *83* (22), 14036–14048.

(21) Fort, Y.; Becker, S.; Caubere, P. A Convenient Synthetic Route to His-Heteroaromatic and Es-Heterocyclic Compounds Promoted by Liganded Nickel Complex Reducing Agents. *Tetrahedron* **1994**, *50*, 11893–11902.

(22) Zhu, D.; Shi, L. Ni-Catalyzed Cross-Coupling of Aryl Thioethers with Alkyl Grignard Reagents via C-S Bond Cleavage. *Chem. Comm.* **2018**, *54* (67), 9313–9316.

(23) Guillén, E.; Hierrezuelo, J.; Martínez-Mallorquín, R.; López-Romero, J. M.; Rico, R. Suzuki-Miyaura Monocouplings of p-Dibromobiphenyl and Substituted p-Dibromo(Penta-p-Phenylenes). *Tetrahedron* **2011**, *67* (14), 2555–2561.

(24) Budén, M. E.; Guastavino, J. F.; Rossi, R. A. Room-Temperature Photoinduced Direct C-H-Arylation via Base-Promoted Homolytic Aromatic Substitution. *Org. Lett.* **2013**, *15* (6), 1174–1177.

(25) Yan, H.; Chellan, P.; Li, T.; Mao, J.; Chibale, K.; Smith, G. S. Cyclometallated Pd(II) Thiosemicarbazone Complexes: New Catalyst Precursors for Suzuki-Coupling Reactions. *Tetrahedron Lett.* **2013**, *54* (2), 154–157.

(26) Samanta, J.; Natarajan, R. Cofacial Organic Click Cage to Intercalate Polycyclic Aromatic Hydrocarbons. *Org Lett* **2016**, *18* (14), 3394–3397.

(27) Glettner, B.; Hein, S.; Reddy, R. A.; Baumeister, U.; Tschierske, C. Cyclic Ureas as Novel Building Blocks for Bent-Core Liquid Crystals. *Chem. Comm.* **2007**, No. 25, 2596–2598.

(28) Ameen, D.; Snape, T. J. A Baker-Venkataraman Retro-Claisen Cascade Delivers a Novel Alkyl Migration Process for the Synthesis of Amides. *Tetrahedron Lett.* **2015**, *56* (14), 1816–1819.

(29) Tripathi, C. B.; Mukherjee, S. Lewis Base Catalysis by Thiourea: N -Bromosuccinimide-Mediated Oxidation of Alcohols. *J. Org. Chem* **2012**, *77* (3), 1592–1598.

(30) Flygare, J. A.; Medina, J. C.; Shan, B.; Clark, D. L.; Rosen, T. J. Pentafluorobenzenesulfonamides and Analogs. US 6482860, **2002**.

(31) González, I.; Mosquera, J.; Guerrero, C.; Rodríguez, R.; Cruces, J. Selective Monomethylation of Anilines by Cu(OAc)2-Promoted Cross-Coupling with MeB(OH)2. *Organic Lett.* **2009**, *11* (8), 1677–1680.

(32) Dong, J.; Wu, Z.; Liu, Z.; Liu, P.; Sun, P. Rhodium(III)-Catalyzed Direct Cyanation of Aromatic C-H Bond to Form 2-(Alkylamino)Benzonitriles Using N-Nitroso As Directing Group. *J. Org. Chem.* **2015**, *80* (24), 12588–12593.

(33) Meng, J.; Xia, H. M.; Xu, A. Q.; Wang, Y. F.; Wang, Z.; Zhang, F. L. Selective N - Monomethylation of Primary Anilines with the Controllable Installation of N -CH2D, N - CHD2, and N -CD3units. *Org. Biomol. Chem.* **2020**, *18* (26), 4922–4926.

(34) Hayashi, K.; Matubayasi, N.; Jiang, C.; Yoshimura, T.; Majumdar, S.; Sasamori, T.; Tokitoh, N.; Kawabata, T. Insights into the Origins of Configurational Stability of Axially Chiral Biaryl Amines with an Intramolecular N-H-N Hydrogen Bond. *J. Org. Chem.* **2010**, *75* (15), 5031–5036.

(35) Iwai, T.; Fujihara, T.; Terao, J.; Tsuji, Y. Iridium-Catalyzed Annulation of N -Arylcarbamoyl Chlorides with Internal Alkynes. *J. Am. Chem. Soc* **2010**, *132* (28), 9602–9603.

(36) Mou, J.; Park, A.; Cai, Y.; Yuan, J.; Yuan, C. Structure-Activity Relationship Study of E6 as a Novel Necroptosis Inducer. *Bioorganic Med. Chem. Lett.* **2015**, *25* (15), 3057–3061.

(37) Youn, S. W.; Ko, T. Y.; Jang, Y. H. Palladium-Catalyzed Regioselective Synthesis of 3-Arylindoles from N -Ts-Anilines and Styrenes . *Angew. Chem. Int. Ed.* **2017**, *129* (23), 6736–6740.

(38) Smirnova, E. S.; Muñoz Molina, J. M.; Johnson, A.; Bandeira, N. A. G.; Bo, C.; Echavarren, A. M. Polynuclear Gold [Au I]4 , [Au I]8 , and Bimetallic [AuI4AgI] Complexes: C–H Functionalization of Carbonyl Compounds and Homogeneous Carbonylation of Amines. *Angew. Chem. Int. Ed* **2016**, *128* (26), 7613–7617.

(39) Du, H.; Zhao, B.; Shi, Y. Catalytic Asymmetric Allylic and Homoallylic Diamination of Terminal Olefins via Formal C-H Activation Supporting Information. *J. Am. Chem. Soc* **2008**, *130*, 8590–8591.
